# Critical Evaluation of Imprinted Gene Expression by RNA–Seq: A New Perspective

**DOI:** 10.1371/journal.pgen.1002600

**Published:** 2012-03-29

**Authors:** Brian DeVeale, Derek van der Kooy, Tomas Babak

**Affiliations:** 1 Department of Molecular Genetics, University of Toronto, Toronto, Canada; 2 Terrence Donnelly Centre for Cellular and Biomolecular Research, University of Toronto, Toronto, Canada; 3 Department of Biology, Stanford University, Stanford, California, United States of America; University of Cambridge, United Kingdom

## Abstract

In contrast to existing estimates of approximately 200 murine imprinted genes, recent work based on transcriptome sequencing uncovered parent-of-origin allelic effects at more than 1,300 loci in the developing brain and two adult brain regions, including hundreds present in only males or females. Our independent replication of the embryonic brain stage, where the majority of novel imprinted genes were discovered and the majority of previously known imprinted genes confirmed, resulted in only 12.9% concordance among the novel imprinted loci. Further analysis and pyrosequencing-based validation revealed that the vast majority of the novel reported imprinted loci are false-positives explained by technical and biological variation of the experimental approach. We show that allele-specific expression (ASE) measured with RNA–Seq is not accurately modeled with statistical methods that assume random independent sampling and that systematic error must be accounted for to enable accurate identification of imprinted expression. Application of a robust approach that accounts for these effects revealed 50 candidate genes where allelic bias was predicted to be parent-of-origin–dependent. However, 11 independent validation attempts through a range of allelic expression biases confirmed only 6 of these novel cases. The results emphasize the importance of independent validation and suggest that the number of imprinted genes is much closer to the initial estimates.

## Author Summary

Typically both copies of mammalian genes are expressed, but in some cases, “imprinting” restricts expression to the maternal or paternal copy. Having two copies of each gene is considered advantageous since in enables compensation when one does not function properly. Why imprinting evolved and its utility to each sex is widely debated, and having a complete catalog of imprinted genes and their functions is essential for fully characterizing this phenomenon. 25 years of screening has revealed about 130 imprinted genes, and the slowing rate of discovery suggests that we are reaching saturation. Two recent studies based on high-throughput sequencing of RNA reported more than 1,300 imprinted genes. To understand the basis of this paradigm shift, we first attempted to reproduce these results. Unable to do so, we performed additional analyses that show that most of these discoveries are due to noise in the experimental approach and assay. We remedy this with new methods that account for this noise and applied them to identify 50 novel putative imprinted genes. These methods will be useful for identifying genuine novel cases of imprinted expression as this type of screening approach becomes broadly utilized.

## Introduction

Why diploid organisms would forgo the safety-net of a redundant genome and preferentially express one allele in a parent-of-origin dependent manner has been a matter of debate since the discovery of imprinted transcription. Our understanding of this issue as well as the range of processes affected by imprinting is dependent on our catalog of the identity, function, and spatial-temporal specificity of imprinted genes.

Imprinting was initially characterized with genetics. Regions where uniparental disomy is not tolerated were mapped by intercrossing reciprocal translocations and comparing viability when both copies of a genomic segment were inherited from one parent to viability when inherited from the other [1]. Continued use of complementation tests in function-based screens provided the most dramatic examples of parent-of-origin effects on development, implicating imprinted transcription in neonatal behavior as well as pre-natal and post-natal growth [2]. Imprinting was then formally demonstrated using nuclear transfer experiments, where aberrant development was observed following transfer of two pronuclei from parents of the same sex [3]. Subsequently, the first individual imprinted transcripts were mapped [4]–[7]. After screening many translocations, genome-wide estimates stood at 100–200 imprinted transcripts [8]. Although based on what we now know was an overestimate for the total number of genes (60,000–100,000) and an underestimate of the number of known imprinted clusters [8], [9], the ∼20 year-old estimate has endured all screening methods applied, including those that do not depend on overt phenotypes. Some of these screens revealed that imprinting occurs outside of the growth axis and affects transcription unrelated to viability or growth [10]–[13]. By Dec 2008, genetic and molecular screening efforts combined yielded 128 confirmed imprinted genes in the mouse.

Transcriptome sequencing of F1 mouse hybrids provides an unbiased alternative for discovering imprinted transcription in wild-type animals [14], [15]. The approach is based on detecting allelic expression with RNA sequencing reads that map over heterozygous SNPs, where the identity of the base is used to distinguish allelic origin and a reciprocal cross is used to discriminate parent-of-origin from strain-specific biases. The first two applications of this approach yielded a small number of novel imprinted transcripts each [14], [15]. However, two recent studies used this approach to identify more than 1,300 imprinted loci, including 484 noncoding RNAs and 347 genes that were sex-specific [16], [17]. These 1,300 loci are an aggregate of the discoveries from E15 brains, adult medial prefrontal cortex (PFC) and adult preoptic area (POA), and represent a ten-fold increase over previously known imprinted genes. The authors suggest that improved sensitivity from increased sequencing depth and improved resolution from sequencing the parents for *de novo* identification of SNPs enabled these advances.

To investigate the biological robustness of these novel imprinted loci, we repeated the embryonic brain screen. Despite faithful technical reproduction of the experimental design, library construction, sequencing, and analysis, we could not reproduce the majority of novel imprinted genes. In this study we demonstrate that biological variation in the approach and technical variation of the assay introduce considerably more noise than was appreciated previously. We develop methods to account for this variation and demonstrate their utility through reanalysis of the published data mentioned above as well as new embryonic brain data.

## Results

### Novel imprinted loci in embryonic brain do not replicate

To distinguish known from novel imprinted loci we compiled a catalog of genomic coordinates for all 128 known mouse imprinted genes that we were able to recover from the literature [13]–[15], [18]–[23] (accessible from GEO [24] under accession GSE27016). All 128 were published prior to the recent papers reporting many more imprinted loci [16], [17].

E15 brains yielded considerably more novel imprinted genes than either the adult PFC or POA (553 vs 153 and 256 respectively), providing the richest opportunity to test reproducibility [17]. Aside from an inexact match in developmental time points (E17.5 vs E15), our experiment was a faithful reproduction of the approach used by Gregg et al. We both used brains from reciprocally crossed C57Bl/6J (B) and CAST/EiJ (C) F1s (from here on BxC will be used to describe F1s derived from B mother and C father; CxB will denote the reciprocal). We both constructed sequencing libraries using the standard Illumina RNA-Seq protocol and sequenced them to 36 bp (single-end) on an Illumina platform. We both used Novoalign (www.novocraft.com) to map reads to UCSC mouse transcripts and noncoding RNAs from the functional RNA database [25]. We used the same set of SNPs [17] and the same criteria for identifying imprinted transcripts (i.e. containing at least one SNP with 10 or more reads with reciprocally biased expression, *p*<0.05; chi-square test). We observed 100% agreement on known imprinted gene calls in E15 brain, POA, and PFC [17], confirming that our analyses were consistent.

**Figure 1:**
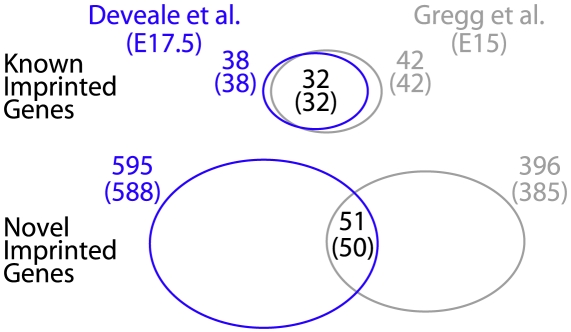
Independent replication with E17.5 brains recapitulates 76.2% (32/42, 0.1 expected by chance) known and 12.9% (51/396, 24 expected by chance) novel imprinted genes reported previously [17]. 12,171 (E15) and 10,418 (E17.5) genes were sufficiently powered for detection (≥1 SNP with ≥10 reads), of which 10,222 were in both samples. Reanalysis excluding SNPs detected in parental transcriptomes while relying exclusively on public SNPs marginally changes the outcome of imprint detection (numbers shown in brackets).

We detected 38 and 42 known imprinted genes in E17.5 and E15 data respectively. 32/42 (76.2%) were detected in both samples (0.1 transcripts expected by chance; Figure 1). This was in sharp contrast to 51/396 (12.9%, 24 expected by chance) novel imprinted genes that confirmed in our screen (Figure 1). This discrepancy is not inconsistent with the experimental validation carried out on novel imprinted genes by Gregg et al.: included in these 51 replicating genes were 2/2 with no previous evidence of imprinting (*Eif2c2* and *DOKist*) that were discovered and further validated in E15 brain [17].

To investigate the benefits of sequencing parents to identify heterozygous SNPs, we repeated this analysis using publicly available SNPs. Perlegen [26] used microarrays to resequence the CAST genome in 2007 and the Wellcome Trust Sanger Institute sequenced 17 mouse strains and released ∼19 million C57Bl/CAST SNPs in 2009 [27]. We converted SNP transcript coordinates published by Gregg et al. [17] to genomic coordinates (July 2007 NCBI 37/mm9) using coordinates of UCSC Known Genes [28]. 99.96% SNPs [17] mapped successfully to 136,532 unique positions. 88.9% of these were among the 19.6 million SNPs identified by Perlegen [26] and/or Sanger [27], of which 98.9% agreed on base identity. The transition∶transversion ratio of SNPs that agree with [26], [27] was 3.00, and 2.06 for the remaining 11% novel SNPs, suggesting that the novel SNPs reported by Gregg et al. [17] have a higher proportion of false-positives. False-positive SNPs cannot lead to an imprinting call since there would be no reads supporting the non-reference CAST allele. Reanalysis of the data using only the 88.9% SNPs that also exist in the public domain yielded nearly identical results (Figure 1; see numbers in parentheses) with no reduction in sensitivity for known imprinted genes and less than 3% sensitivity reduction in novel regions. This demonstrates that sequencing biological parents when SNPs are publicly available from sequenced parental strains provides little added benefit [26], [27].

### Allele-specific expression (ASE) measured with RNA–Seq is exceedingly more noisy than accounted for by basic counting statistics

Statistical modeling of allele-specific expression measured by transcriptome sequencing is an unresolved challenge [29], [30]. Gregg et al. [16], [17] used a chi-square metric that assumes no experimental biases are introduced during library construction, sequencing, genomic alignment, and that each sequencing read is independent of all other reads. Unfortunately these assumptions are often violated [30]–[34], and systematic error in quantifying allele-specific expression by RNA-Seq is just now becoming apparent [29], [35]–[37].

To begin to understand the underlying cause of inconsistent imprinting calls we investigated the accuracy of ASE quantification with RNA-Seq. It has previously been shown that ASE measured at the same SNP is highly reproducible across technical and biological replicates [17], [38]. However, this comparison is immune to systematic error such as priming, fragmentation, and PCR biases that arise during library construction, sequencing chemistry [36], and read alignment [31]. Since most sources of systematic error are sequence dependent, a more informative test would compare concordance in ASE within the same sample, but between independently sampled sites where the level of ASE is the same. We reasoned that SNPs within the same coding exon should satisfy this requirement since there are few known biological phenomena that could disrupt this relationship. Allele-specific premature transcriptional termination is possible, but has to our knowledge not been documented. Furthermore, premature termination codons are relatively infrequent [39] and those introduced by alternative splicing lead to immediate degradation by nonsense-mediated decay [40]. We did not use exons containing UTRs since transcription start sites vary significantly [41] and 3′UTRs are extensively processed [42]. We also restricted our analysis to RefSeq coding exons, which are based on experimentally validated transcripts to maximize the accuracy of same-exon SNP associations.

**Figure 2:**
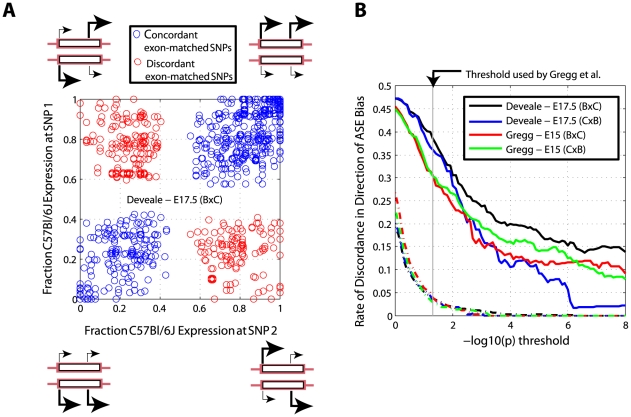
Direction of allele-specific expression bias measured at SNPs in the same exon disagrees more frequently than expected by chance. (A) Expression bias at 1,388 pairs of SNPs under ASE (chi-square *p*<1e-4 for both SNPs) disagrees in direction for 20.0% of pairs in E17.5 BxC data. (B) Discordance improves with increasing *p*-value thresholds but is higher than simulated data where allelic counts for one SNP in each pair are randomly sampled from the *χ* ^2^ distribution (broken lines; see Materials and Methods).

We estimated the accuracy of ASE quantification as the frequency of SNP pairs within the same exon that agree on direction of bias. We considered all SNP pairs separated by more than 40 bp, which is the longest used sequencing read length [17], to ensure independent sampling. At a *p*-value threshold of 0.0001 (i.e. both *p*-values in each SNP pair are less than 0.0001) we observed 1,388 SNP pairs in our E17.5 BxC data, of which 278 (20.03%) disagreed on direction of bias (Figure 2A). If ASE measured with RNA-Seq was adequately modeled by random sampling, we would expect less than 0.1% to be discordant at this level of significance (Figure 2B and see Materials and Methods). We observed exceedingly higher levels of discordance than expected across all levels of significance (Figure 2B). Nonetheless, *p*-values computed from a basic chi-square test are predictive since higher thresholds result in lower rates of discordance and do not reach an asymptote (within extent of available data). This indicates that: 1) our test is valid and not confounded by biological differences in ASE between SNPs in the same coding exon, and 2) counting statistics can be a reliable approach for identifying ASE, although at face-value lead to a major over-estimate of significance.

### Technical and biological variation explain the majority of novel imprinted genes

**Figure 3:**
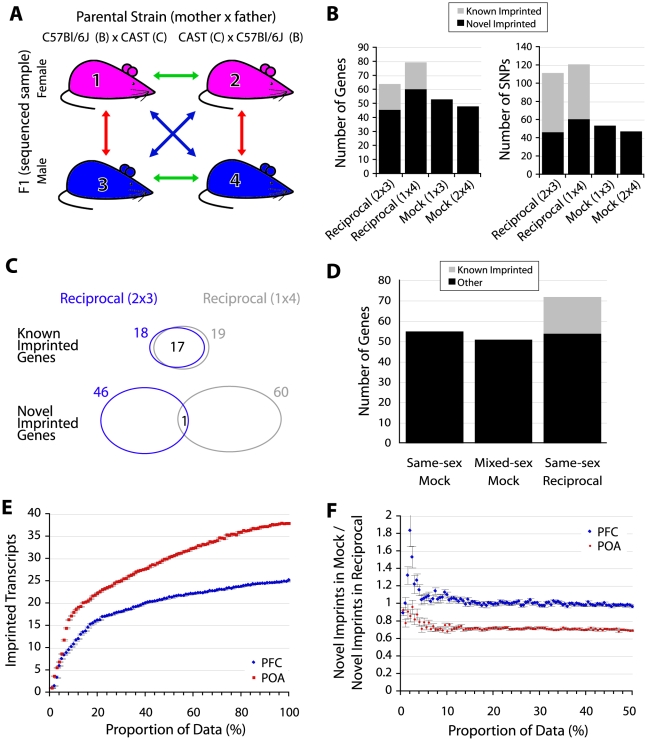
False discoveries explain the majority of novel imprinted genes. (A) Schematic of experimental design and mock (negative control) comparisons. The red arrows indicate mock comparisons, green arrows indicate same-sex reciprocal comparisons, while blue arrows indicate mixed-sex reciprocal comparisons. (B) Number of SNPs/genes that exceed threshold used by [16], [17] in reciprocally crossed and mock PFC samples. (C) Agreement on known and novel imprinted genes detected in the two reciprocal comparisons shown in 2B. (D) Number of genes exceeding the threshold used in [16], [17] in a mock cross between E17.5 brains from embryos of the same sex (males) is not significantly different from mock comparison of opposite sexes. (E) Relationship between read depth and sensitivity (number of transcripts reaching significance). Aligned reads from the same cross (1+3 and 2+4) were selected at random in equal proportions for each sample (see Materials and Methods). Average values for 100 sampling iterations (+/− standard error) are shown. (F) Proportion of novel imprinted genes estimated to be false-positives as a function of input read depth adjusted by random sampling (see Materials and Methods). Average values for 100 sampling iterations (+/− standard error) are shown.

For discovery of imprinted genes, a simple negative control that accounts for systematic error, technical variation, and biological variation is to ask how many SNPs/genes exceed significance in a mock reciprocal cross (i.e. comparing samples with the same parental background as though they were from reciprocal crosses). In such a comparison any reciprocally biased expression cannot be caused by genomic imprinting and is a measure of the technical and biological variation of the experimental approach. Data from two animals of opposite sex was available for PFC and POA and enabled two mock-cross analyses (Figure 3A). Strikingly, in the mock reciprocal cross, nearly as many imprinted gene calls exceeded the significance threshold used by Gregg et al. as in the reciprocal cross (Figure 3B). Similar to our comparison in embryonic brains, the majority of the known imprinted gene calls from reciprocal crosses were the same, but novel calls were not (Figure 3C). Comparing males to females in the reciprocal analysis controlled for sex-specific expression biases. We confirmed that mock-reciprocal hits are not caused by differences in sex by generating additional sequencing data from a male E17.5 brain sample. This enabled a comparison based on true (sex-matched) biological replicates and revealed that male vs male and male vs female BxC mock comparisons produced equivalent numbers of false positive measurements (Figure 3D). While we used approximately half of the data to generate calls (Figure 3B), randomly removing mapped reads from aggregated data revealed that sensitivity is not markedly different at 50% vs 100% of input reads (Figure 3E). Furthermore, the estimated proportion of novel imprinted genes that are false-positives is not impacted by further down-sampling (the slope of the line is consistent when sufficient data exists to overcome noise; Figure 3F) and an overestimate on account of reduced sequencing depth is thus unlikely. We note that an aggregate mock comparison (1+4 vs 2+3) is not an informative negative control since potential sex-specific imprinted genes would not be balanced in this scheme and the output would be a mixture of true sex-specific imprinted genes and false-positives where the contribution of each is not clear.

**Figure 4:**
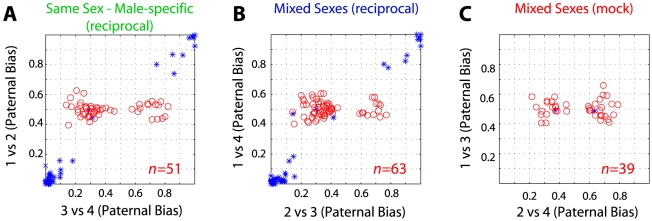
False-discoveries explain the majority of sex-specific imprinted genes. Frequency of SNPs meeting the criteria used in [16], [17] to report sex-specific imprinted genes when comparing male PFCs from reciprocal crosses (A) as in [16], [17] as well as mixed-sex reciprocal (B) and mixed-sex mock comparisons (C). Red points (o) are SNPs that are biased toward the same parent-of-origin (exceed 10 read counts, *p*<0.05) in the comparison indicated on the x-axis but not in the comparison indicated on the y-axis (the criteria in [16], [17] for sex-specific imprinted gene calls). Blue points are SNPs exceeding significance in comparison of animals on both axes.

A high false-discovery rate may also explain the large number of sex-specific imprinted genes reported by Gregg et al. [16] since these, by definition [16], only reach significance in a comparison of one reciprocal cross (e.g. between males) and not the other (e.g. between females). If this were a reliable assay for identifying sex-specific imprinted genes then nothing should meet significance by comparing opposite sexes within each cross, since expression at a sex-specifically imprinted locus would always be biallelic in one animal. 51 imprinted genes in reciprocally crossed male PFC samples reached significance that did not reach significance in reciprocally crossed females at the threshold used by Gregg et al. (but had sufficient coverage to make a call; 36 agreed with Gregg et al. [16], *p*<1e-63). However, a similar number reached significance in negative controls (63 genes in mixed-sex and 39 in mock reciprocals; Figure 4). We obtained similar ratios for female-specific PFC imprinted genes and all POA comparisons (Figure S1), demonstrating that this approach is not sufficiently powered at the selected threshold of statistical significance.

### Discovery of novel imprinted genes

**Figure 5:**
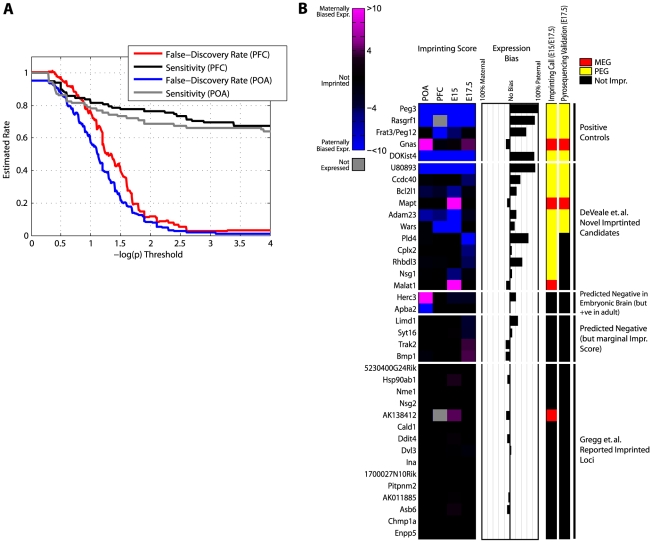
Validating features predictive of genomic imprinting. (A) Estimated false-discovery rate as a function of *p*-value threshold. Allele-specific expression counts were summed over all SNPs within each gene. False-discovery was estimated as the number of significant genes in a mock cross (1 versus 3+2 vs 4; see Figure 3A) divided by the number of significant genes in a reciprocal cross (1 versus 4+2 versus 3). Sensitivity was computed as the number of known imprinted genes meeting significance in the reciprocal cross (1 versus 4+2 versus 3) as a fraction of imprinted genes powered for detection (at least one SNP with ≥10 reads in both animals). (B) Imprinting scores (left panel), allelic bias (middle panel), our imprinting call and pyrosequencing call (right panel) for 37 loci tested by pyrosequencing. Imprinting Scores are log_10_(*p*), where *p* is the less significant *p*-value from chi-square tests performed on the two reciprocal crosses; negatives arbitrarily represent paternal bias. Expression bias is the allelic bias measured in RNA-Seq data from the average of embryonic brain data (E15/E17.5). Imprinting call is based on an imprinting score threshold of 4 (*p*<1e-4) in E15 and/or E17.5 brains. Pyrosequencing validation was done on total RNA from E17.5 brains and calibrated on biological replicates (see Materials and Methods for details). Imprinted Loci reported by Gregg et al. represent 17 randomly selected SNPs reported imprinted in E15 [17].

To estimate the total number of imprinted genes we first asked how many are detected in the four available datasets (E15 brain, E17.5 brain, adult PFC and POA). Aggregating allele-specific reads across SNPs in the same gene improved our sensitivity in known imprinted regions (data not shown) and we thus applied this approach genome-wide. We also took advantage of all publicly available SNPs [17], [26], [27] and expanded our alignment reference to include the whole genome (see Materials and Methods). Using the mock/reciprocal approach to estimate false-discovery (Figure 5A), we proceeded with *p* = 1e-4 (FDR<0.05) as a threshold of significance for calling a gene imprinted (in addition to the standard reciprocal bias toward sex of parent and ≥10 reads in each cross). We selected *p* = 1e-4 as a threshold (e.g. as opposed to 1e-3) since candidates with scores between 1e-3 and 1e-4 did not validate by pyrosequencing (see below) and sensitivity is negligibly impacted (Figure 5A). We identified a total of 53 putative imprinted genes in at least one sample (Figure S2). 5/53 occurred in all 4 samples and 3 (*Eif2c2*, *Cdh15*, and *DOKist4*) were validated by Gregg et al [17]. We also detected 56 genes that were previously known to be imprinted (27 in all 4 samples). Of the putative novel genes, 4 are probable extensions of known imprinted genes based on EST or transcription evidence derived from this data (3 of the 5 putative novel imprinted genes which recur in all 4 samples), 7 others are associated with known imprinted clusters (within 1 Mb) and 42 are completely novel (Table S1).

### Pyrosequencing validation of novel imprinted genes in E17.5 brain

From manual inspection we identified three distinguishing features among the known imprinted genes that we detected and reasoned these may be useful for predicting novel imprinted regions. These include: 1) reciprocal allelic bias and high sequencing depth (reflected in conjunction as the imprinting score), 2) agreement on imprinted expression among neighboring SNPs, and 3) recurrence of signal across biological replicates and/or tissues. To further investigate the predictive potential of these features we identified 37 candidate imprinted loci that represent a range of values for each feature and tested these by pyrosequencing. In addition to 5 positive controls, we tested 17 candidate loci selected at random from the list of imprinted SNPs reported by Gregg et al [17] in E15, 4 candidates with marginal imprinting scores (between 1e-3 and 1e-4), 2 candidates detected in adult but not embryonic brain, 7 candidates detected only in embryonic brain, and 5 candidates detected in at least two samples. All 17 candidates reported Gregg et al. are from the ‘complex’ category where the imprint does not agree with other SNPs in the same gene (this category accounts for 94.8% of the novel imprinted genes reported [17]).

**Figure 6:**
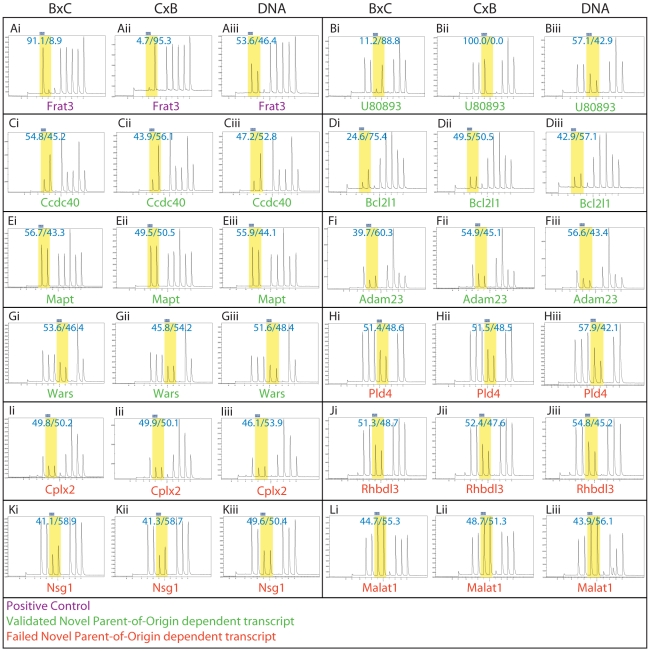
Pyrosequencing traces confirmed 6 of the 11 predicted novel parent-of-origin effects in E17.5 brains. For each gene (A–L), (i) are traces from E17.5 brains from BxC parents, (ii) are traces of E17.5 brains from CxB parents and (iii) are traces on genomic DNA from BxC parents. (A) *Frat3* is a positive control. (B–G) Confirmed novel parent-of-origin effects. The *Bcl2l1* traces illustrate the utility of sequencing 50∶50 hybrid BxC DNA to calibrate each assay. In this particular assay an unknown effect caused a slight allelic bias (57∶43) even in the case where input was 50∶50 B∶C DNA. If assay bias is not accounted for, the *Bcl2l1* reciprocal F1 traces suggest a genetic background effect on allelic bias, but support a parent-of-origin effect once assay bias is accounted for by normalization to the 50∶50 hybrid DNA. Please see Materials and Methods for details on estimating significance of bias from technical replicate runs. (H–L) Predicted novel parent-of-origin effects that did not validate. In all cases, SNPs are highlighted in yellow with the percent contribution of each nucleotide superimposed on each. Note that the sequencing chemistry is such that adenosine produces 16% more signal than other nucleotides in the trace (Qiagen, personal communication) and the Pyromark software accounts for this when calculating the allelic ratio.

Pyrosequencing validation suggests that all three features are predictive (Figure 5B; Table S2; Figure 6). To establish a level of technical and biological variance in our pyrosequencing assays, we first measured ASE for the 37 loci in biological replicates (two BxC E17.5 brain samples) and observed excellent agreement (Figure S3). This also enabled us to establish a meaningful threshold for detecting differences in ASE (see Materials and Methods). In agreement with our results suggesting that the majority of the Gregg et al imprinting calls are false-positives as well our own imprinting calls on these 17 loci (16/17 predicted to be negative), none validated by pyrosequencing (Figure 5B; Table S2). Of 11/16 predicted parent-of-origin effects in embryonic brain (incl. 5 positive controls) that validated, all contain more than one SNP where parental bias was observed, suggesting that consensus SNP calls may be a valuable predictor. The imprinting score was also predictive; the average (absolute) value of the 6/11 novel parent-of-origin effects that validated was 28.4 vs 6.7 for the 5/11 predictions that did not validate and none of the four tested predictions in the 3–4 range validated (Figure 5B; Table S2). 5/7 predictions detected in only one of the embryonic samples as well as the two detected only in POA did not validate, suggesting that recurring detection in more than one related sample is also informative.

## Discussion

### How many imprinted genes are there?

A recurring conclusion throughout this study is that there is no evidence for mammals having an order of magnitude more imprinted loci than was previously appreciated, as two recent papers claim [16], [17]. Independent replication of this work, reanalysis that included negative controls to estimate false-discovery, and follow-up validation using an independent assay unilaterally suggest that the vast majority of the reported imprinted genes are false-positives explained by variation in the assay and experimental approach. This is in agreement with long-standing genetic estimates and other high-throughput screens, some of which used the same global approach [14], [15], that reported at most a few novel imprinted genes each. Furthermore, novel loci that typically validate (this study incl.) are close to known imprinted regions and are likely uncharacterized extensions of known imprinted transcripts and/or rely on an imprinting mechanism of an established region. Nearly all the novel candidates emerging from RNA-Seq screens thus far (this study incl.) are marginally significant relative to known expressed imprinted genes that typically have a strong signal.

Is it possible then that hundreds to thousands of undiscovered imprinted genes exist? Yes, but there is no evidence for it. These would need to occur in developmental stages or tissues not yet assayed or represent transcripts that are invisible to standard RNA-Seq. Examples of imprinted expression that evade RNA-Seq are non-polyadenylated transcripts, antisense transcripts imprinted/expressed to similar degrees and in opposite directions such that the signal cancels out, and biases that occur to such a minor extent that they are detected below the threshold of noise. Because RNA-Seq cannot be modeled with counting statistics that assume each read is randomly and independently sampled and free of systematic bias, it is imperative that this threshold is firmly defined. Without accounting for background, SNPs with very small parent-of-origin biases resulting from assay variance may appear imprinted with high statistical significance, particularly if the SNP is highly expressed. Even if some of these were real, our pyrosequencing efforts did not validate any. One could still argue that the effect is below the threshold of pyrosequencing detection (e.g. less than a 5% difference in this study), but if this were the case then the effect on transcriptional load would be small and likely without functional consequence. In any case, the current state of RNA-Seq and analysis cannot detect these minor effects, even if they do exist.

A more fruitful application of RNA-Seq to imprinting discovery in the near term may involve screening additional developmental time-points, tissues, and species. RNA-Seq methods that do not require polyA+ selection and that retain strand-specificity may also uncover novel transcripts of the noncoding/antisense variety, some of which have already been shown to be important for establishing and maintaining imprinting states [43]. Finally, integration with complementary global datasets, such as genome-wide allele-specific methylation maps, will improve both specificity of imprinting discovery as well as insight into the underlying mechanisms.

Overall, 6/11 novel parent-of-origin effects validated by pyrosequencing, with the average allelic bias measured by pyrosequencing indicated in parentheses: *U80893* (44.4%), *Ccdc40* (5.5%), *Bcl2l1* (12.5%), *Mapt* (3.6%), *Adam23* (9.3%) and *Wars* (3.9%). As with *U80893*, *Adam23* and *Wars*, *Bcl2l1* is located in close proximity to known imprinted transcript (all <150 kB from a known imprinted transcript). *U80893* is a trinucleotide-repeat (CAG) containing gene of unknown function [44]. *Ccdc40* is essential for left-right patterning in both mice and humans, full range cilia motility and a causal mutation for a variant of primary ciliary dyskinesia in humans [45]. *Bcl2l1* inhibits apoptosis [46]. *Mapt* is primarily known as a major component of the prevalent neurofibrillary tangle pathophysiology in Alzheimer's disease [47], suggesting that imprinted expression of *Mapt* in humans may have relevance to the inheritance and progression of Alzheimer's. *Adam23* mutants are smaller than wild type littermates and exhibit tremors and ataxia [48] and *Wars* is uncharacterized. Three of these genes, *Wars*, *Ccdc40*, and *Mapt*, were marginally biased in their parent-of-origin expression (Figure 5B, Figure 6, Dataset S1). This may be due to a mechanism that causes incomplete silencing or due to allele-specific expression in only a subset of cell types/tissues comprising the organ assayed. Using more homogeneous samples provides an obvious path forward, but revealing the functional consequences to these types of imprinting cases is the real challenge since many novel imprinted genes awaiting discovery will likely fall into this category.

An estimate for the total number of imprinted genes must account for several variables, including the increasing difficulty in validating novel candidates. Of the 6/11 candidates that validated in this study, only 2 of the 6 represent parent-of-origin effects that are clearly not associated with previously known imprinted regions (Figure 5B and Figure S2). This severely limits our power to establish a firm number and an estimate should be interpreted with caution. Nonetheless, assuming a confirmation rate of 37.5% (3/8) for the remaining untested embryonic brain candidates and a detection sensitivity of 56/128 (known imprinted genes) extrapolates to an estimate of 37 imprinted genes awaiting discovery and validation, yielding an estimate for a total number of ∼175 imprinted genes.

### Lessons learned: Implications for future imprinting discovery with RNA–Seq

Although the statistically-derived imprinting score is the strongest predictor of imprinting, it cannot be interpreted as a direct measure of probability. As others have noted, RNA-Seq is not free of systematic error [29], [31], [35], [36] which may impact measurement of ASE. We show that allele-specific read counts are substantially less accurate for quantifying ASE than expected from a random sampling approach. Although our metric for quantifying error in measuring ASE is concordance in direction of bias, this measure becomes synonymous with false-positive rate in identifying ASE as sampling iterations become large and the full range of significance thresholds is represented. Our model could thus be extended to serve as a correction for the significance of ASE to yield an empirical measure of false-discovery when identifying regions with ASE. Since detection of imprinting requires demonstration of reciprocal bias, most of the effect introduced by systematic error (which is sequence-dependent) is likely eliminated. Our analysis confirms this expectation: FDR estimated from a mock-reciprocal analysis (Figure 5A) reaches manageable levels at more relaxed thresholds of significance than modeling ASE (Figure 2B).

Second, recurrence of imprinted expression across related samples increases the likelihood that novel candidates validate. Although exceptions exist [13], if expression of a given gene is imprinted in adulthood, its expression is generally also imprinted in the developmental precursor of that tissue. Data in Wamidex [13], for example, suggests ∼90% concordance between imprinted expression of an adult tissue with its precursor tissue. 20.0% and 75.0% of the putative novel imprinted genes in the POA and PFC respectively were also imprinted in the E15 and/or E17.5 brain (Figure S4). These numbers increase to 68.5% and 93.3% respectively, if known imprinted genes are included. The rate of independent confirmation amongst putative imprinted transcripts was higher for those reaching the threshold in multiple samples, suggesting that these figures are likely an underestimate. Other high probability candidates that were not assayed independently but fit this profile include: *AK039650/AK044369*, *DQ715667*, *Klhdc10*, and *C230091D08Rik*, while *Herc3* is likely to confirm in the adult POA.

Third, consensus in parent-of-origin allelic bias among neighboring SNPs provides additional predictive potential. Read aggregation across SNPs in the same gene enabled detection of concordance (the same gene exhibiting imprinted expression in more than one sample) in several genes that appeared biallelic using *p*<0.05 without read aggregation [17]. Examples include *Wars* (POA), *Adam23* (POA), *Klhdc10* (E15, POA), and *Cdh15* (E15, PFC).

Fourth, proximity of novel candidates to known imprinted regions or to each other is also predictive. Most known imprinted genes occur in clusters that can span hundreds of kilobases [14] and typically share regulatory mechanisms. 7 (of 8) putative novel imprinted genes that were identified in at least two samples are associated with known imprinted regions (<1 Mb), as well as three detected in only one sample (Figure S2 and Table S1). *Adam23* is ∼100 kB from *Zdbf2*, *C230091D08Rik* is ∼1 kB from *Ube3a*, *U80893* may be part of the contiguous transcription extending from *Zfp127* [14], Aceview [49] suggests *DQ715667* is part of *Eif2c2 which* is 500 kB from *Kcnk9*, *AK039650/AK044369* appears to be an extension of *Kcnk9*, *Klhdc10* is ∼250 kB from *Mest*, and *Wars* is ∼150 kB from *Begain*. Furthermore, 6 putative novel imprinted genes form 3 clusters of 2 genes within 200 kB of each other (Table S1). Two of these, *Wars* and *Slc25a29*, are <600 kB from the *Dlk1* locus.

In conclusion, until we can accurately model ASE measured with RNA-Seq, estimating FDR of imprinted gene discovery will ideally be done empirically. The additional criteria mentioned above can be used to further rank novel candidates, but since no combination of criteria was absolutely predictive among the novel imprinted genes identified in this study, we assert that independent validation is essential for making definitive claims about imprinting of any gene.

## Materials and Methods

### Calling imprinted genes

We downloaded sequencing data and SNP calls from GEO (GSE22131) and used the same strategy as Gregg et al. [16], [17] to score imprinted expression. We aligned the data to UCSC known genes (transcripts) and noncoding RNAs downloaded from the Functional RNA Database [50]. We called a SNP ‘imprinted’ if greater than 50% of the reads in each sample mapped to alleles from the same parental sex (*p*<0.05, chi-square test) in both samples. We compiled a non-redundant set of gene models from UCSC known genes [28] by taking the union of transcribed bases when multiple isoforms overlap (i.e. collapse isoforms into one gene model that contains all exons), yielding 26,214 models, of which 19,867 were coding (available under GSE27016). 13,604 gene models had at least one SNP identified by Gregg et al [17]. We called a gene imprinted if it contained one or more imprinted SNP(s). Sex-specific calls were made as described [16].

### Concordance in ASE direction of bias

The raw fastq data was realigned against RefSeq transcripts and the mouse genome (mm9) using Novoalign. 19.6 million C57Bl/CAST SNPs, representing the union of Perlegen [26] and Sanger [27], were masked prior to alignment to reduce alignment biases caused by more sequence mismatches between CAST and C57Bl reference genome than C57Bl reads [31]. Uniquely mapping reads (in genomic space; ∼80% of all reads on average) were retained for further analysis. Non-redundant RefSeq coding exons were identified to avoid multiple sampling bias arising from the same SNP present in multiple isoforms. In cases where more than one coding exon overlapped in the genome, the longest coding exon was selected. All pairs of SNPs within each exon were used for comparisons (e.g. three comparisons were done if three SNPs were present), but SNP pairs separated by 40 bp or less (the longest sequencing read length [17]) were not considered to ensure independent sampling. Significance of ASE was computed using a chi-square test as described above, and the less significant (i.e. higher) p-value was used to set the significance threshold for that comparison (x-axis in Figure 2B). For the simulated analysis, the more significant SNP in each pair was replaced with expected counts and adjusted for variance with random sampling from the chi-square distribution with one degree of freedom. For example, if one SNP had 30 and 70 C57 and CAST reads respectively (expect 50 and 50, chi-square *p* = 6.3e-5, df = 1) and the other had 30 and 20 (expect 25 and 25, chi-square *p* = 0.157, df = 1), the allelic counts of the first SNP would be replaced by 60 and 40 (±variance). If a random sampling of the chi-square distribution yields, for example, *χ* ^2^ = 1, rearranging Pearson's formula for the *χ* ^2^ statistic and solving the quadratic reveals simulated counts of 65 or 55 and 35 or 45 for C57 and CAST respectively. A schematic summary of the approach is provided (Figure S5). Replacing the more significant SNP (as opposed to the less significant SNP) was done to ensure direct comparison with the observed data where the less significant *p*-value defines the threshold of significance for both SNPs. Replacing the less significant pair led to a further reduction in expected discordance (data not shown). This process was repeated for each SNP pair and concordance was computed as described above.

### Mock comparisons

Imprinting calls were made using criteria described above. To minimize sampling bias reads aligned to chromosome X and the mitochondrial chromosome were not considered in this analysis. Furthermore, all samples were normalized to have the same number of aligned input reads. For example, for the PFC comparison, the BxC male sample had the fewest aligned reads so reads were randomly removed from the three remaining samples (BxC female, CxB male, and CxB female) to exactly match BxC male.

### E17.5 brain data

All animals were housed and treated in accordance with the Institutional and Governmental Animal Care Committee guidelines of the University of Toronto. Whole brains were dissected from 2 male BxC E17.5 brains, 1 female BxC E17.5 brain and 1 male CxB E17.5 brain, snap chilled in liquid nitrogen and immediately homogenized in Trizol. Total RNA was extracted with Trizol according to the manufacturer's recommendations (Invitrogen) and integrity was confirmed on an Agilent Bioanalyzer (RIN>9 for both samples). PolyA+ RNA was isolated using a Dynabead mRNA Purification Kit according to the manufacturer's instructions (Invitrogen). Double stranded cDNA libraries were made using the Illumina mRNA-Seq kit according to the manufacturer's recommendations (Illumina) and converted into libraries (adapter ligation, PCR, cleanup) using a NEBNext kit according to the manufacturer's recommendations (NEB). We retained strand-specificity by using dUTP during second-strand synthesis and an UNG treatment prior to the final amplification as described previously [51]. Libraries were verified on an Agilent Bioanalyzer and by qRT-PCR, and each sequenced to 36 bp on an Illumina HiSeq 2000 platform, yielding on average 80.1 million reads. Raw data and alignments are available at GEO under accession GSE27016. Brains were processed independently such that each library is derived from a unique sample without pooling.

### Discovery of novel imprinted genes

For global discovery of novel imprinted genes we aligned to gene models as described above, all possible splice-junction sequences (100 bases; 50 bases from each flanking exon) representing up to two exon-skipping events in these gene models, as well as the genome. We converted all alignments to genomic coordinates and retained uniquely mapping reads for further analysis. 79.9% of reads aligned uniquely (on average 80.1% per sample), of which 7.7% spanned splice-junctions (17.6% of reads that overlapped coding exons also spanned splice junctions) and 0.12% represented exon-skipping events. Imprinting was assessed at each SNP as described above.

### Pyrosequencing validation

We identified SNPs amenable to pyrosequencing (non-consecutive nucleotides separated from other SNPs) within transcripts of interest and designed assays using Pyromark Assay Design 2.0 requiring assay scores >87. The sequence ‘CGCCAGGGTTTTCCCAGTCACGAC’ was added to the 5′ end of all primers designated for biotinylation to enable biotin incorporation during PCR following an approach reported previously [52] with some modifications. Specifically, biotinylated amplicons were generated directly from RNA using the Pyromark OneStep RT-PCR kit (Qiagen) according to the manufacturer's instructions with the addition of a third HPLC purified universal biotinylated primer (biotin-CGCCAGGGTTTTCCCAGTCACGAC) added at 9/10 the recommended molarity to supplement the RT-PCR primer designated for biotinylation, which was added at 1/10 the recommended molarity. Other combinations ranging from 5∶5 to 9∶10 did not result in a noticeable difference in product yield and size (as assayed by an Agilent Bioanalyzer) or pyrosequencing performance. We performed sequencing on a Pyromark Q96 MD (Qiagen) according to the manufacturer's instructions and quantified allelic bias using the AE quantification software included with the instrument (all traces available in Dataset S1). To maximize sensitivity of detecting small allelic changes we defined the null ratio by sequencing DNA as described previously [53]. Transcripts were called imprinted if the difference in allelic bias between the reciprocal samples were >5.02% and where each ratio was reciprocally biased (i.e. in opposite directions) relative to ratio obtained with DNA. 5.02% represents 2 standard deviations of variance determined from comparing allelic ratios between biological replicates (Figure S3) and corresponds to a significance of *p*<0.022. Both biological replicates had to meet these criteria and DNA ratios were averaged between the two DNA samples prior to normalization. A negative pyrosequencing call includes cases that do not meet significance as well as cases where reciprocal bias was not observed. The majority of negatives fall into the latter category and relaxing the threshold of significance to *p*<0.2 did not change the outcome of any calls. 5/42 assays attempted were removed due to technical failure (two because of a high skew in the DNA ratio and three where the Pyromark AE quantification software could not accurately identify peaks) leaving 37 assays successfully executed across all 5 samples.

## Supporting Information

Dataset S1 Pyrosequencing traces analyzed to validate putative novel imprinted genes. Table of contents details page numbers for each assay. In brief, 5 traces were run for each assay that are grouped together. These are in the same order for each: BxC RNA biological replicate 1, CxB RNA biological replicate 1, BxC RNA biological replicate 2, BxC DNA biological replicate 1 and BxC DNA biological replicate 2.
(PDF)

Figure S1 The majority of sex-specific imprints reported [17] are due to technical and biological variance. Red points (o) are SNPs that are biased toward the same parent-of-origin (exceed 10 read counts, *p*<0.05) in the comparison indicated on the x-axis but not in the comparison indicated on the y-axis (the criteria in [16], [17] for sex-specific imprint calls). Blue points are SNPs exceeding significance in comparison of animals on both axes.
(EPS)

Figure S2 Imprinting scores of candidate imprinted genes discovered in E17.5 brain data and reanalysis of E15, PFC, and POA samples [17], clustered by genomic coordinates.
(EPS)

Figure S3 Expression bias measured by pyrosequencing is reproducible in biological replicates. Linear regression was calculated on the agreement in allelic bias for each of the 37 loci assayed by pyrosequencing. Each data point represents a single assay performed on two independent biological replicates of BxC E17.5 brains. Details on the assays are provided in Table S1.
(EPS)

Figure S4 Significance of detection for 53 novel imprinted genes at significance exceeding *p*<0.0001. Imprinting Score is log10(*p*), where p is the less significant *p*-value from chi-square tests performed on the two reciprocal crosses; negatives arbitrarily represents paternal bias.
(EPS)

Figure S5 Schematic illustrating how discordance rates plotted in Figure 2 were generated.
(EPS)

Table S1 Putative imprinted genes with Imprinting Scores <−4 or >4 in one of the four samples when aggregating read counts within a transcript. The table provides the following detail for each candidate: Gene ID, Genome Coordinates, Imprinting Scores (E15 Brains, E17.5 Brains, PFC, POA), Distance to Nearest Known Imprinted Gene, Nearest Known Imprinted Gene, Putative Cluster, Gene Name, Gene/Alternate Gene Symbol, Gene/Proteome/Description, Gene/SwissProt/Keywords, Gene/Gene Ontology via Proteome & Entrez Gene/Biological Process, Gene/Gene Ontology via Proteome & Entrez Gene/Molecular Function, Gene/Gene Ontology via Proteome & Entrez Gene/Cellular Component, Gene/KEGG Pathways, Transcript/Description, SwissProt ID, Proteome ID, Unigene ID.
(XLS)

Table S2 Putative imprinted loci validated or refuted by pyrosequencing. Table provides detail relevant to each assay: Gene/Transcript, Transcript Model, Reason for Testing, Additional Information, SNP Coordinate (mm9), RNA-Seq allelic counts, Imprinting Scores (POA, PFC, E15 brain, E17.5 brain), Imprinting Calls, Allelic Bias (POA, PFC, E15 Brain, E17.5 Brain), Pyrosequencing Calls, BxC # standard deviations from DNA, CxB # standard deviations from DNA, F1 primer sequence, R1 primer sequence, S1 primer sequence.
(XLS)

## References

[1] Searle AG, Beechey CV (1978). Complementation studies with mouse translocations.. Cytogenet Cell Genet.

[2] Beechey CVaC, B.M. (1993). Mouse' Genome.

[3] Surani MA, Barton SC, Norris ML (1984). Development of reconstituted mouse eggs suggests imprinting of the genome during gametogenesis.. Nature.

[4] Barlow DP, Stoger R, Herrmann BG, Saito K, Schweifer N (1991). The mouse insulin-like growth factor type-2 receptor is imprinted and closely linked to the Tme locus.. Nature.

[5] DeChiara TM, Robertson EJ, Efstratiadis A (1991). Parental imprinting of the mouse insulin-like growth factor II gene.. Cell.

[6] Bartolomei MS, Zemel S, Tilghman SM (1991). Parental imprinting of the mouse H19 gene.. Nature.

[7] Ferguson-Smith AC, Cattanach BM, Barton SC, Beechey CV, Surani MA (1991). Embryological and molecular investigations of parental imprinting on mouse chromosome 7.. Nature.

[8] Barlow DP (1995). Gametic imprinting in mammals.. Science.

[9] Morison IM, Ramsay JP, Spencer HG (2005). A census of mammalian imprinting.. Trends Genet.

[10] Renfree MB, Hore TA, Shaw G, Graves JA, Pask AJ (2009). Evolution of genomic imprinting: insights from marsupials and monotremes.. Annu Rev Genomics Hum Genet.

[11] Cowley M, Oakey RJ (2010). Retrotransposition and genomic imprinting.. Brief Funct Genomics.

[12] Barlow DP (1993). Methylation and imprinting: from host defense to gene regulation?. Science.

[13] Schulz R, Woodfine K, Menheniott TR, Bourc'his D, Bestor T (2008). WAMIDEX: a web atlas of murine genomic imprinting and differential expression.. Epigenetics.

[14] Babak T, Deveale B, Armour C, Raymond C, Cleary MA (2008). Global survey of genomic imprinting by transcriptome sequencing.. Curr Biol.

[15] Wang X, Sun Q, McGrath SD, Mardis ER, Soloway PD (2008). Transcriptome-wide identification of novel imprinted genes in neonatal mouse brain.. PLoS ONE.

[16] Gregg C, Zhang J, Butler JE, Haig D, Dulac C (2010). Sex-specific parent-of-origin allelic expression in the mouse brain.. Science.

[17] Gregg C, Zhang J, Weissbourd B, Luo S, Schroth GP (2010). High-resolution analysis of parent-of-origin allelic expression in the mouse brain.. Science.

[18] Menheniott TR, Woodfine K, Schulz R, Wood AJ, Monk D (2008). Genomic imprinting of Dopa decarboxylase in heart and reciprocal allelic expression with neighboring Grb10.. Mol Cell Biol.

[19] Ding F, Prints Y, Dhar MS, Johnson DK, Garnacho-Montero C (2005). Lack of Pwcr1/MBII-85 snoRNA is critical for neonatal lethality in Prader-Willi syndrome mouse models.. Mamm Genome.

[20] Wood AJ, Bourc'his D, Bestor TH, Oakey RJ (2007). Allele-specific demethylation at an imprinted mammalian promoter.. Nucleic Acids Res.

[21] Schulz R, Menheniott TR, Woodfine K, Wood AJ, Choi JD (2006). Chromosome-wide identification of novel imprinted genes using microarrays and uniparental disomies.. Nucleic Acids Res.

[22] Parker-Katiraee L, Carson AR, Yamada T, Arnaud P, Feil R (2007). Identification of the imprinted KLF14 transcription factor undergoing human-specific accelerated evolution.. PLoS Genet.

[23] Monk D, Wagschal A, Arnaud P, Muller PS, Parker-Katiraee L (2008). Comparative analysis of human chromosome 7q21 and mouse proximal chromosome 6 reveals a placental-specific imprinted gene, TFPI2/Tfpi2, which requires EHMT2 and EED for allelic-silencing.. Genome Res.

[24] Edgar R, Domrachev M, Lash AE (2002). Gene Expression Omnibus: NCBI gene expression and hybridization array data repository.. Nucleic Acids Res.

[25] Mituyama T, Yamada K, Hattori E, Okida H, Ono Y (2009). The Functional RNA Database 3.0: databases to support mining and annotation of functional RNAs.. Nucleic Acids Res.

[26] Frazer KA, Eskin E, Kang HM, Bogue MA, Hinds DA (2007). A sequence-based variation map of 8.27 million SNPs in inbred mouse strains.. Nature.

[27] Keane TM, Goodstadt L, Danecek P, White MA, Wong K (2011). Mouse genomic variation and its effect on phenotypes and gene regulation.. Nature.

[28] Fujita PA, Rhead B, Zweig AS, Hinrichs AS, Karolchik D (2011). The UCSC Genome Browser database: update 2011.. Nucleic Acids Res.

[29] Nothnagel M, Wolf A, Herrmann A, Szafranski K, Vater I (2011). Statistical inference of allelic imbalance from transcriptome data.. Hum Mutat.

[30] Robinson MD, Oshlack A (2010). A scaling normalization method for differential expression analysis of RNA-seq data.. Genome Biol.

[31] Degner JF, Marioni JC, Pai AA, Pickrell JK, Nkadori E (2009). Effect of read-mapping biases on detecting allele-specific expression from RNA-sequencing data.. Bioinformatics.

[32] Kozarewa I, Ning Z, Quail MA, Sanders MJ, Berriman M (2009). Amplification-free Illumina sequencing-library preparation facilitates improved mapping and assembly of (G+C)-biased genomes.. Nat Methods.

[33] Main BJ, Bickel RD, McIntyre LM, Graze RM, Calabrese PP (2009). Allele-specific expression assays using Solexa.. BMC Genomics.

[34] Mamanova L, Andrews RM, James KD, Sheridan EM, Ellis PD (2010). FRT-seq: amplification-free, strand-specific transcriptome sequencing.. Nat Methods.

[35] Fontanillas P, Landry CR, Wittkopp PJ, Russ C, Gruber JD (2010). Key considerations for measuring allelic expression on a genomic scale using high-throughput sequencing.. Mol Ecol.

[36] Heap GA, Yang JH, Downes K, Healy BC, Hunt KA (2010). Genome-wide analysis of allelic expression imbalance in human primary cells by high-throughput transcriptome resequencing.. Hum Mol Genet.

[37] Turro E, Su SY, Goncalves A, Coin LJ, Richardson S (2011). Haplotype and isoform specific expression estimation using multi-mapping RNA-seq reads.. Genome Biol.

[38] Babak T, Garrett-Engele P, Armour CD, Raymond CK, Keller MP (2010). Genetic validation of whole-transcriptome sequencing for mapping expression affected by cis-regulatory variation.. BMC Genomics.

[39] Ng SB, Turner EH, Robertson PD, Flygare SD, Bigham AW (2009). Targeted capture and massively parallel sequencing of 12 human exomes.. Nature.

[40] Matsuda D, Hosoda N, Kim YK, Maquat LE (2007). Failsafe nonsense-mediated mRNA decay does not detectably target eIF4E-bound mRNA.. Nat Struct Mol Biol.

[41] Yamashita R, Sathira NP, Kanai A, Tanimoto K, Arauchi T (2011). Genome-wide characterization of transcriptional start sites in humans by integrative transcriptome analysis.. Genome Res.

[42] Wilhelm BT, Marguerat S, Watt S, Schubert F, Wood V (2008). Dynamic repertoire of a eukaryotic transcriptome surveyed at single-nucleotide resolution.. Nature.

[43] Royo H, Cavaille J (2008). Non-coding RNAs in imprinted gene clusters.. Biol Cell.

[44] Kim SJ, Shon BH, Kang JH, Hahm KS, Yoo OJ (1997). Cloning of novel trinucleotide-repeat (CAG) containing genes in mouse brain.. Biochem Biophys Res Commun.

[45] Becker-Heck A, Zohn IE, Okabe N, Pollock A, Lenhart KB (2011). The coiled-coil domain containing protein CCDC40 is essential for motile cilia function and left-right axis formation.. Nat Genet.

[46] Motoyama N, Wang F, Roth KA, Sawa H, Nakayama K (1995). Massive cell death of immature hematopoietic cells and neurons in Bcl-x-deficient mice.. Science.

[47] Nukina N, Ihara Y (1986). One of the antigenic determinants of paired helical filaments is related to tau protein.. J Biochem.

[48] Mitchell KJ, Pinson KI, Kelly OG, Brennan J, Zupicich J (2001). Functional analysis of secreted and transmembrane proteins critical to mouse development.. Nat Genet.

[49] Thierry-Mieg D, Thierry-Mieg J (2006). AceView: a comprehensive cDNA-supported gene and transcripts annotation.. Genome Biol.

[50] Kin T, Yamada K, Terai G, Okida H, Yoshinari Y (2007). fRNAdb: a platform for mining/annotating functional RNA candidates from non-coding RNA sequences.. Nucleic Acids Res.

[51] Parkhomchuk D, Borodina T, Amstislavskiy V, Banaru M, Hallen L (2009). Transcriptome analysis by strand-specific sequencing of complementary DNA.. Nucleic Acids Res.

[52] Royo JL, Hidalgo M, Ruiz A (2007). Pyrosequencing protocol using a universal biotinylated primer for mutation detection and SNP genotyping.. Nat Protoc.

[53] Wang H, Elbein SC (2007). Detection of allelic imbalance in gene expression using pyrosequencing.. Methods Mol Biol.

