# Supplementary material for: Critical Evaluation of Imprinted Gene Expression by RNA–Seq: A New Perspective
Source: PLoS Genet. 2012 Mar 29;8(3):e1002600. doi: 10.1371/journal.pgen.1002600 (PMC3315459; doi:10.1371/journal.pgen.1002600)

Table of contents: Pyrosequencing Traces

Table of contents: Pyrosequencing Traces	Pages
Peg3 (BxC 1, CxB 1, BxC 2, BxC DNA 1, BxC DNA 2)	1-5
Rasgrf1 (BxC 1, CxB 1, BxC 2, BxC DNA 1, BxC DNA 2)	6-10
Frat3 (BxC 1, CxB 1, BxC 2, BxC DNA 1, BxC DNA 2)	11-15
Gnas (BxC 1, CxB 1, BxC 2, BxC DNA 1, BxC DNA 2)	16-20
DOKist4 (BxC 1, CxB 1, BxC 2, BxC DNA 1, BxC DNA 2)	21-25
U80893 (BxC 1, CxB 1, BxC 2, BxC DNA 1, BxC DNA 2)	26-30
C230091D08Rik (BxC 1, CxB 1, BxC 2, BxC DNA 1, BxC DNA 2)	31-35
Limd1 (BxC 1, CxB 1, BxC 2, BxC DNA 1, BxC DNA 2)	36-40
Syt16 (BxC 1, CxB 1, BxC 2, BxC DNA 1, BxC DNA 2)	41-45
Trak2 (BxC 1, CxB 1, BxC 2, BxC DNA 1, BxC DNA 2)	46-50
Bmp1 (BxC 1, CxB 1, BxC 2, BxC DNA 1, BxC DNA 2)	51-55
Pld4 (BxC 1, CxB 1, BxC 2, BxC DNA 1, BxC DNA 2)	56-60
Cplx2 (BxC 1, CxB 1, BxC 2, BxC DNA 1, BxC DNA 2)	61-65
Ccdc40 (BxC 1, CxB 1, BxC 2, BxC DNA 1, BxC DNA 2)	66-70
Rhbd13 (BxC 1, CxB 1, BxC 2, BxC DNA 1, BxC DNA 2)	71-75
Bcl2l1 (BxC 1, CxB 1, BxC 2, BxC DNA 1, BxC DNA 2)	76-80
Nsg1 (BxC 1, CxB 1, BxC 2, BxC DNA 1, BxC DNA 2)	81-85
Malat1 (BxC 1, CxB 1, BxC 2, BxC DNA 1, BxC DNA 2)	86-90
Mapt (BxC 1, CxB 1, BxC 2, BxC DNA 1, BxC DNA 2)	91-95
Herc3 (BxC 1, CxB 1, BxC 2, BxC DNA 1, BxC DNA 2)	96-100
Apba2 (BxC 1, CxB 1, BxC 2, BxC DNA 1, BxC DNA 2)	101-105
Adam23 (BxC 1, CxB 1, BxC 2, BxC DNA 1, BxC DNA 2)	106-110
Wars (BxC 1, CxB 1, BxC 2, BxC DNA 1, BxC DNA 2)	111-115
5230400G24Rik (BxC 1, CxB 1, BxC 2, BxC DNA 1, BxC DNA 2)	116-120
Hsp90ab1 (BxC 1, CxB 1, BxC 2, BxC DNA 1, BxC DNA 2)	121-125
Nme1 (BxC 1, CxB 1, BxC 2, BxC DNA 1, BxC DNA 2)	126-130
Nsg2 (BxC 1, CxB 1, BxC 2, BxC DNA 1, BxC DNA 2)	131-135
AK138412 (BxC 1, CxB 1, BxC 2, BxC DNA 1, BxC DNA 2)	136-140
Cald1 (BxC 1, CxB 1, BxC 2, BxC DNA 1, BxC DNA 2)	141-145
Ddit4 (BxC 1, CxB 1, BxC 2, BxC DNA 1, BxC DNA 2)	146-150
Dvl3 (BxC 1, CxB 1, BxC 2, BxC DNA 1, BxC DNA 2)	151-155
Ina (BxC 1, CxB 1, BxC 2, BxC DNA 1, BxC DNA 2)	156-160
1700027N10Rik (BxC 1, CxB 1, BxC 2, BxC DNA 1, BxC DNA 2)	161-165
Pitpnm2 (BxC 1, CxB 1, BxC 2, BxC DNA 1, BxC DNA 2)	166-170
AK011885 (BxC 1, CxB 1, BxC 2, BxC DNA 1, BxC DNA 2)	171-175
Asb6 (BxC 1, CxB 1, BxC 2, BxC DNA 1, BxC DNA 2)	176-180
Chmp1a (BxC 1, CxB 1, BxC 2, BxC DNA 1, BxC DNA 2)	181-185
Enpp5 (BxC 1, CxB 1, BxC 2, BxC DNA 1, BxC DNA 2)	186-190

10 uL universal (141+157) - Well D5
Entry: Peg3
1: C: 0.0% / T: 100.0%
(Passed)

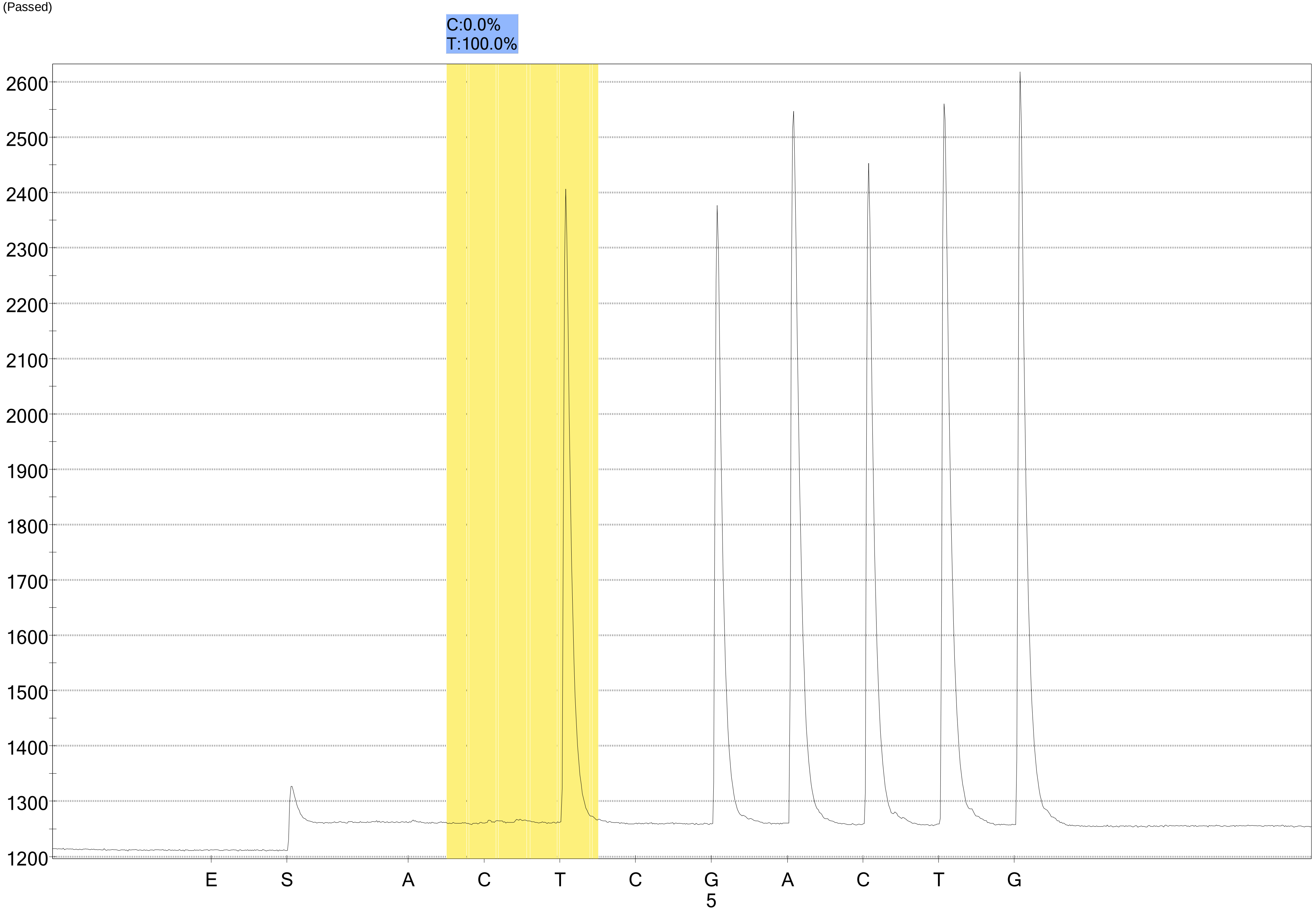

10 uL universal (141+157) - Well D11
Entry: Peg3
1: C: 92.7% / T: 7.3%
(Passed)

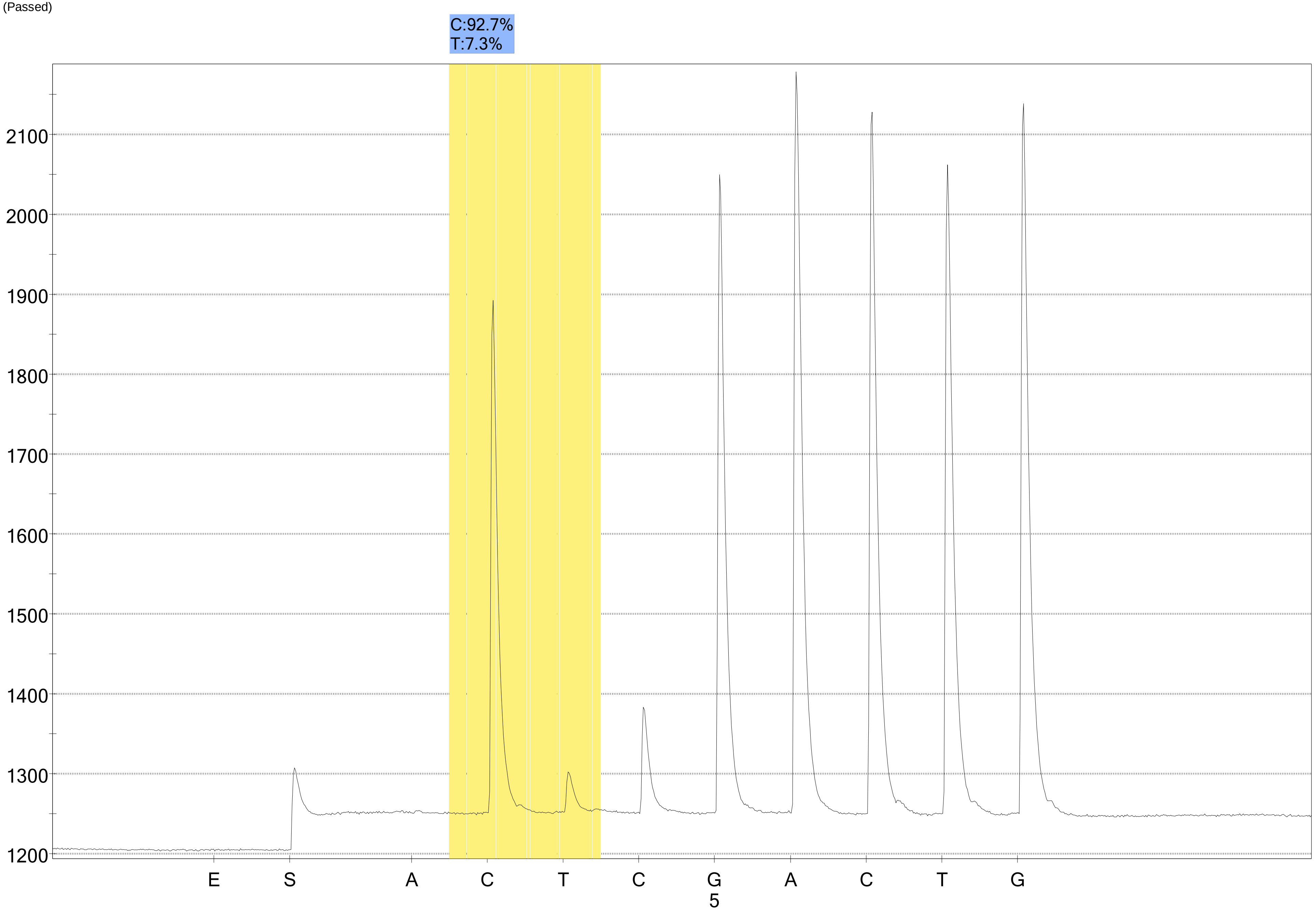

145 - Well D5
Entry: Peg3
1: C: 0.8% / T: 99.2%
(Passed)

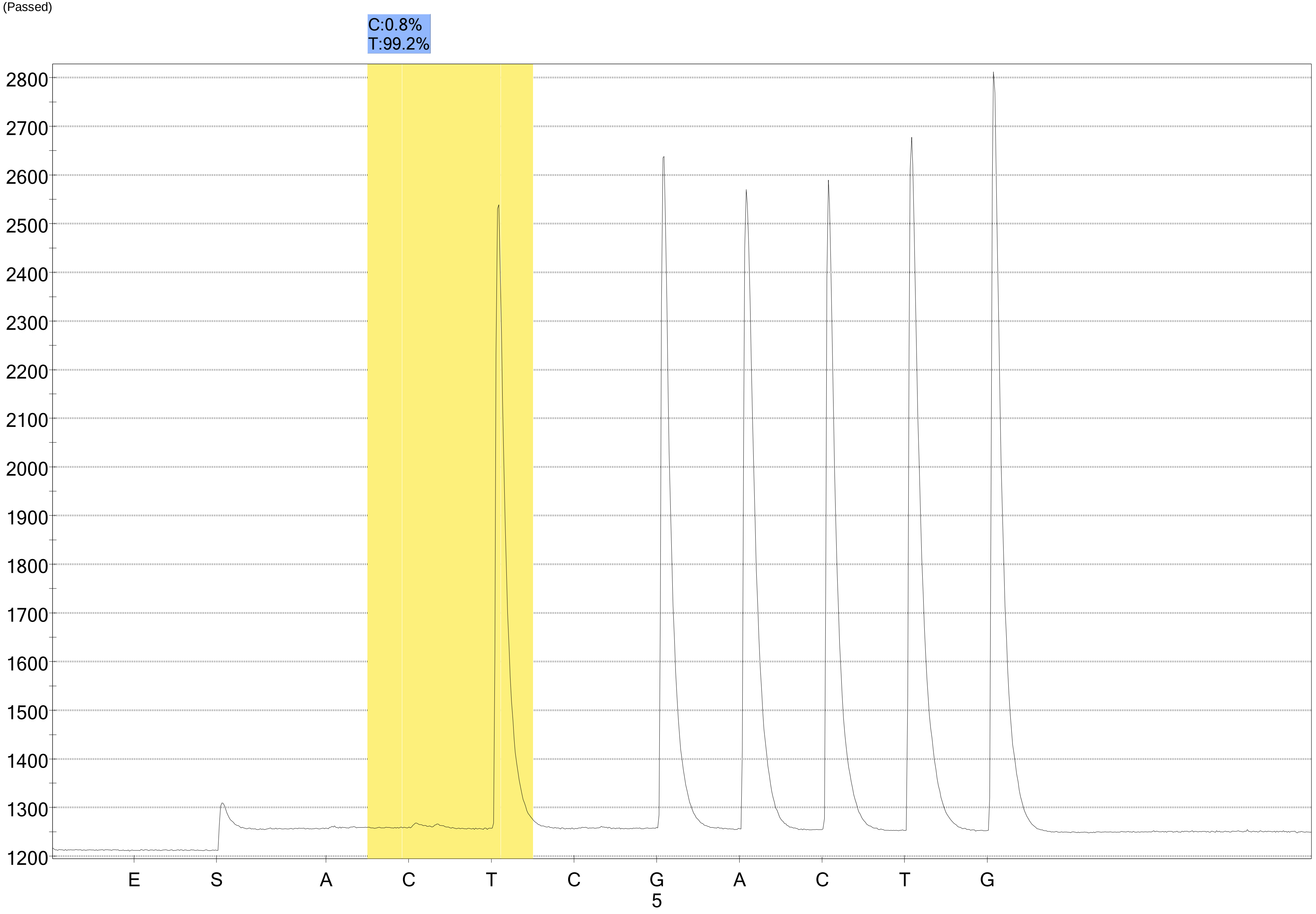

dna - Well D5
Entry: Peg3
1: C: 36.8% / T: 63.2%
(Passed)

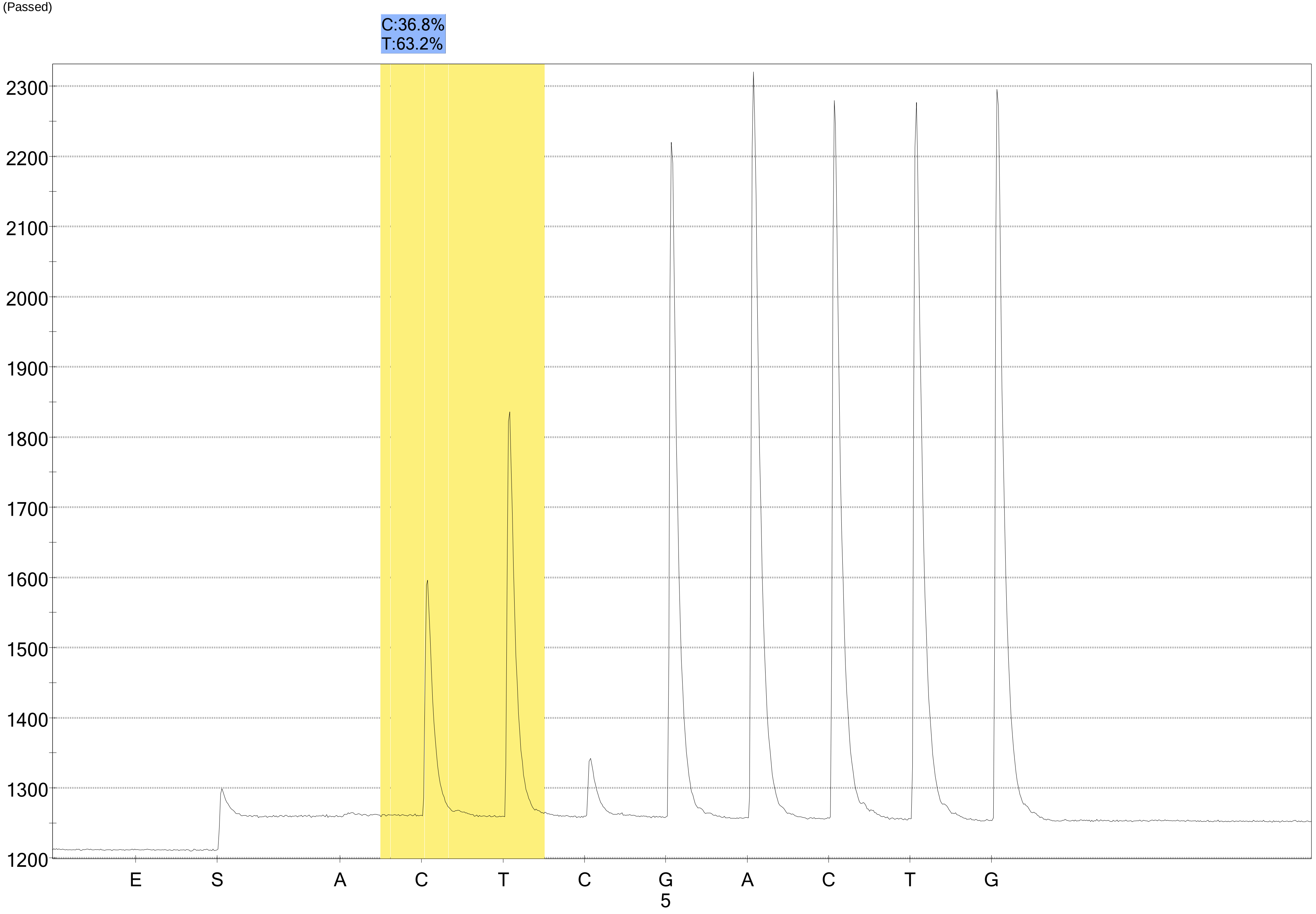

dna - Well D11
Entry: Peg3
1: C: 40.0% / T: 60.0%
(Passed)

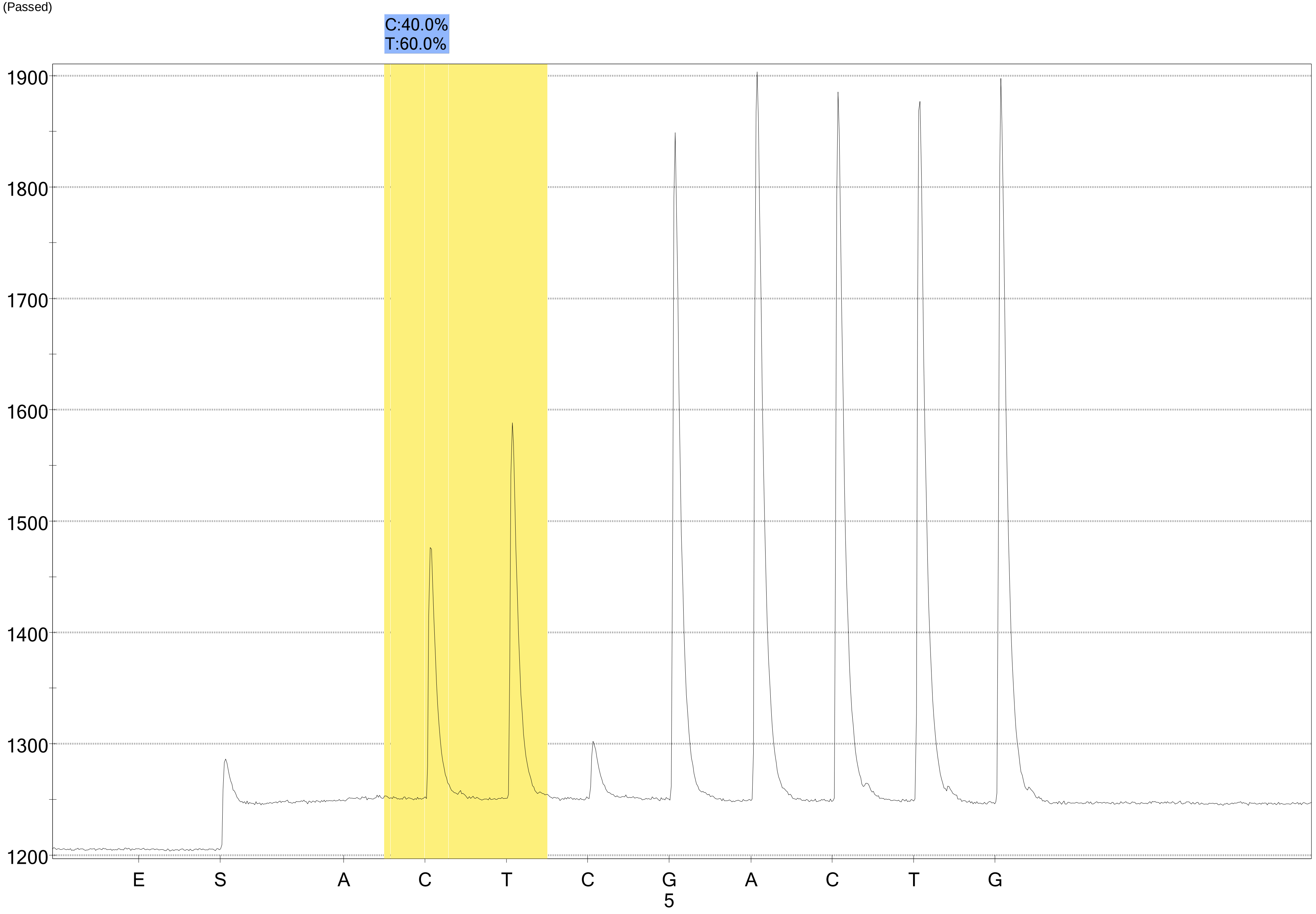

10 uL universal (141+157) - Well G5
Entry: Rasgrf1
2: A: 89.7% / T: 10.3%
(Passed)

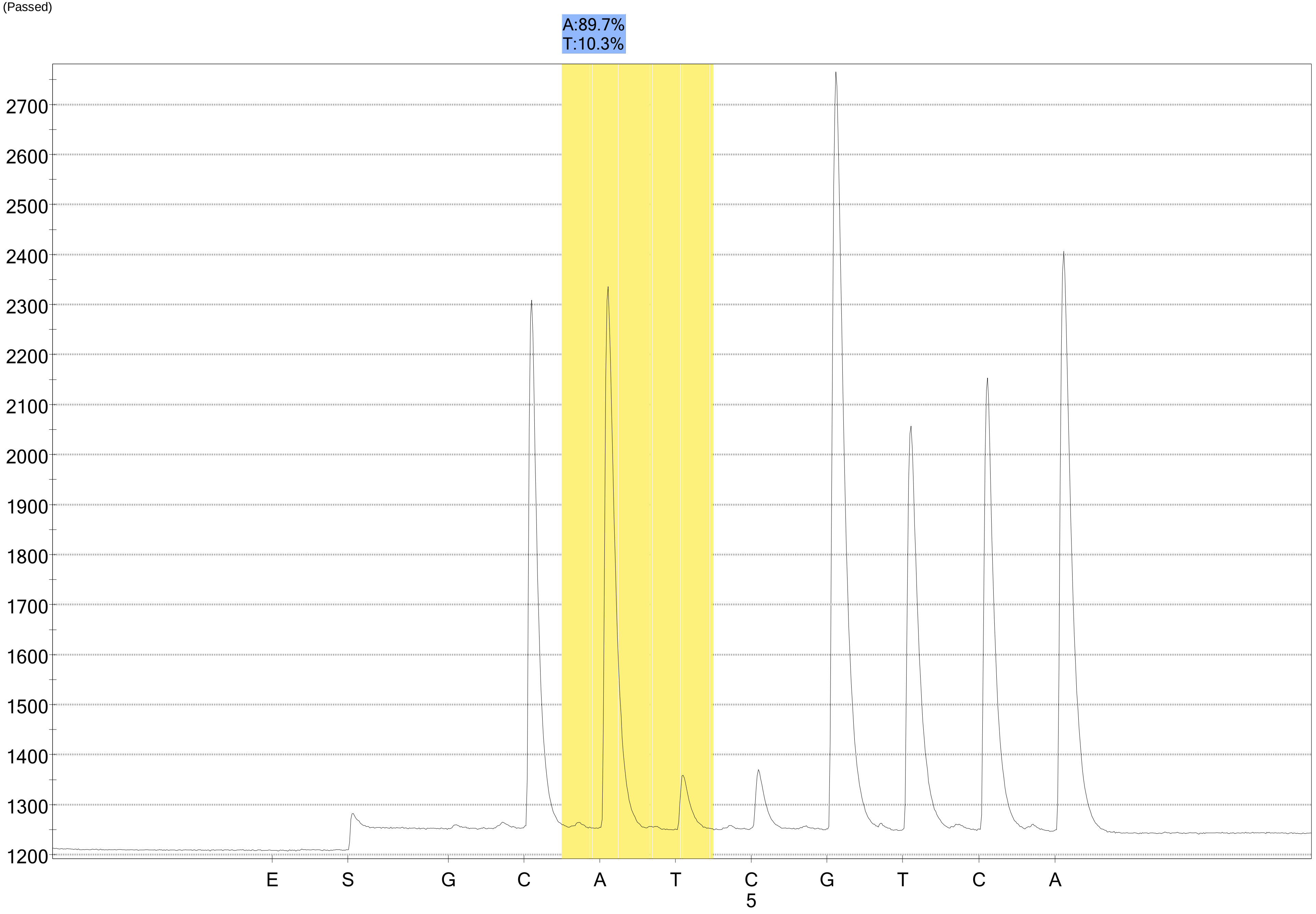

10 uL universal (141+157) - Well G11
Entry: Rasgrf1
2: A: 2.9% / T: 97.1%
(Passed)

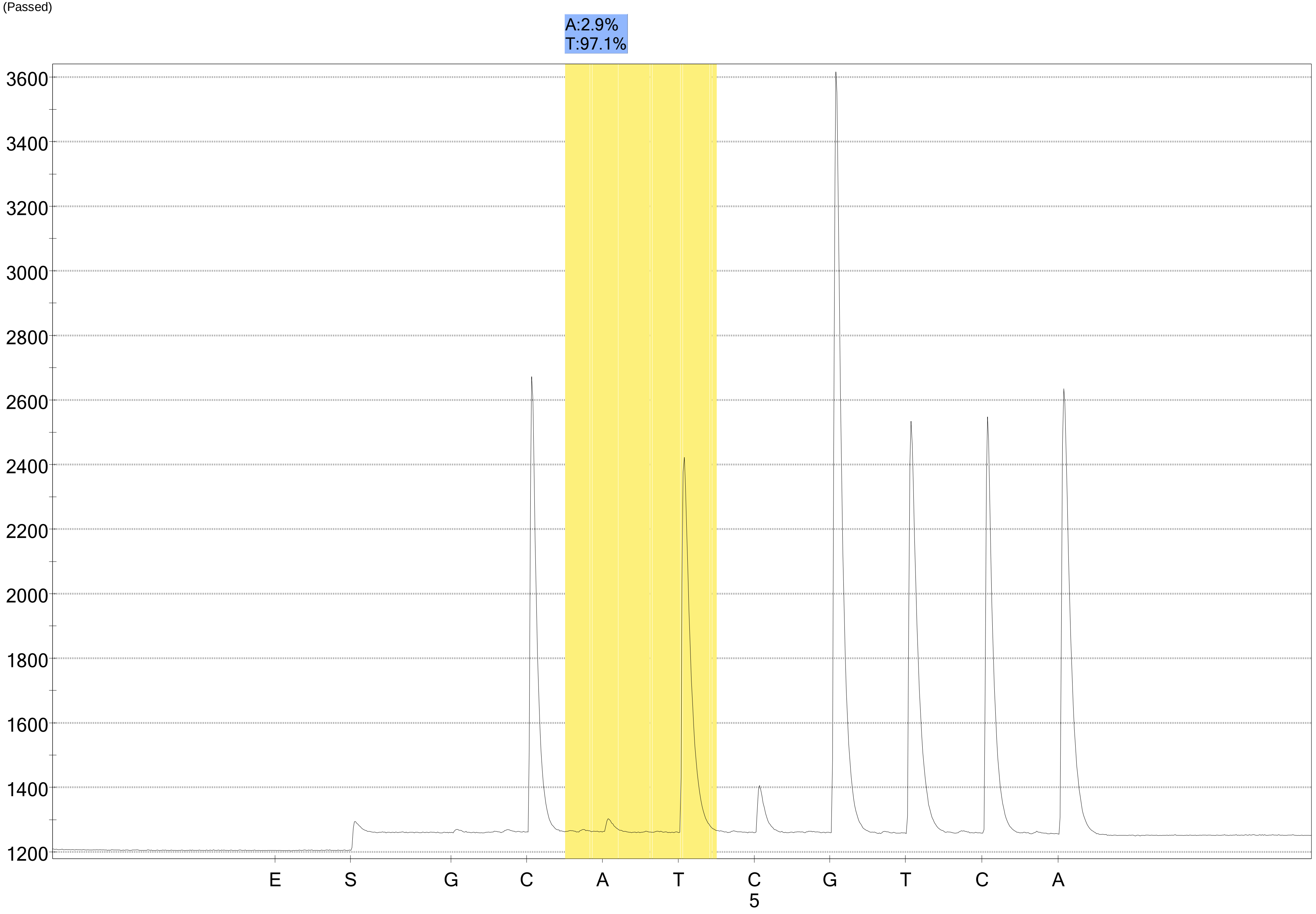

145 - Well G5
Entry: Rasgrf1
2: A: 87.7% / T: 12.3%
(Passed)

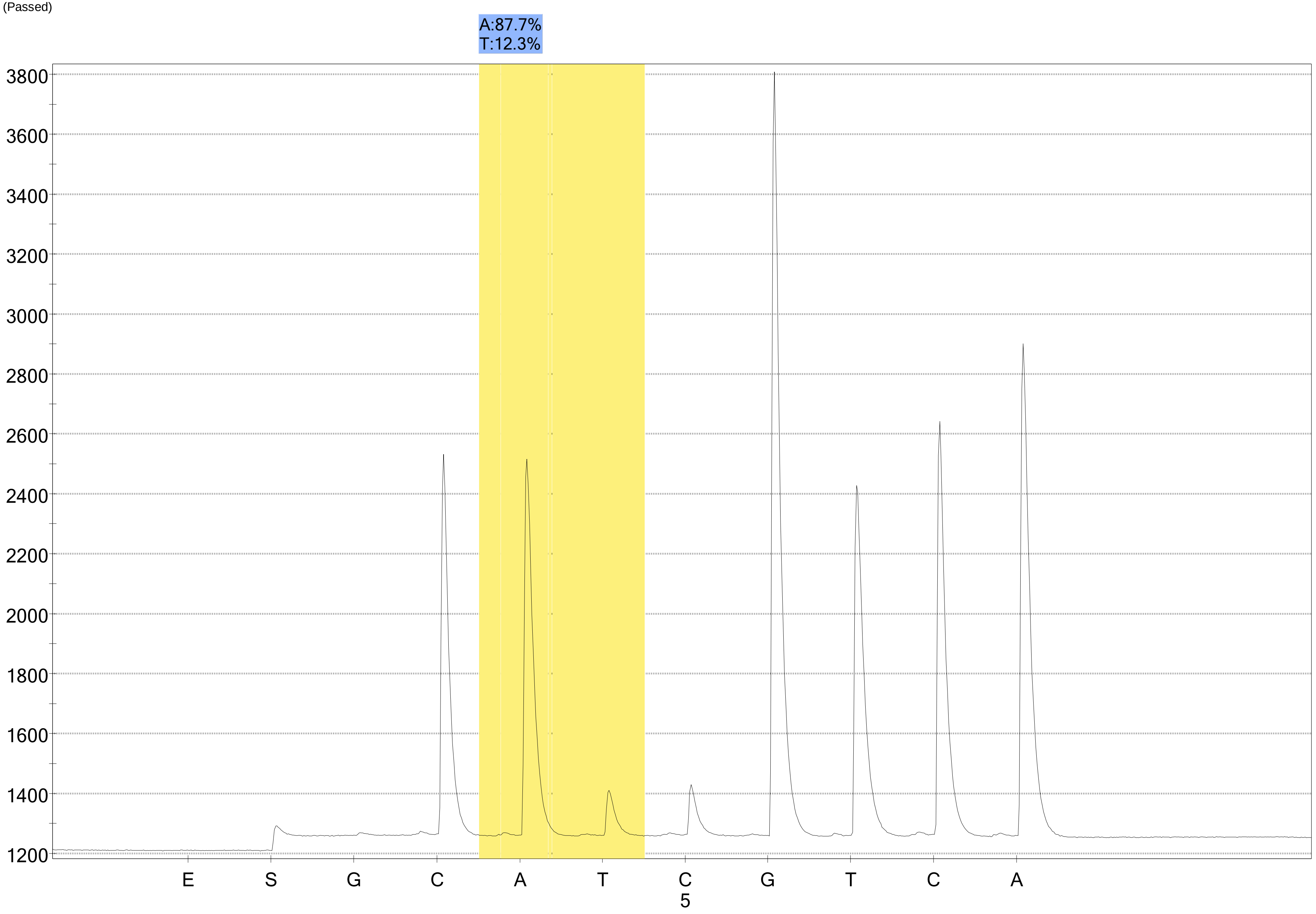

dna - Well G5
Entry: Rasgrf1
2: A: 20.3% / T: 79.7%
(Passed)

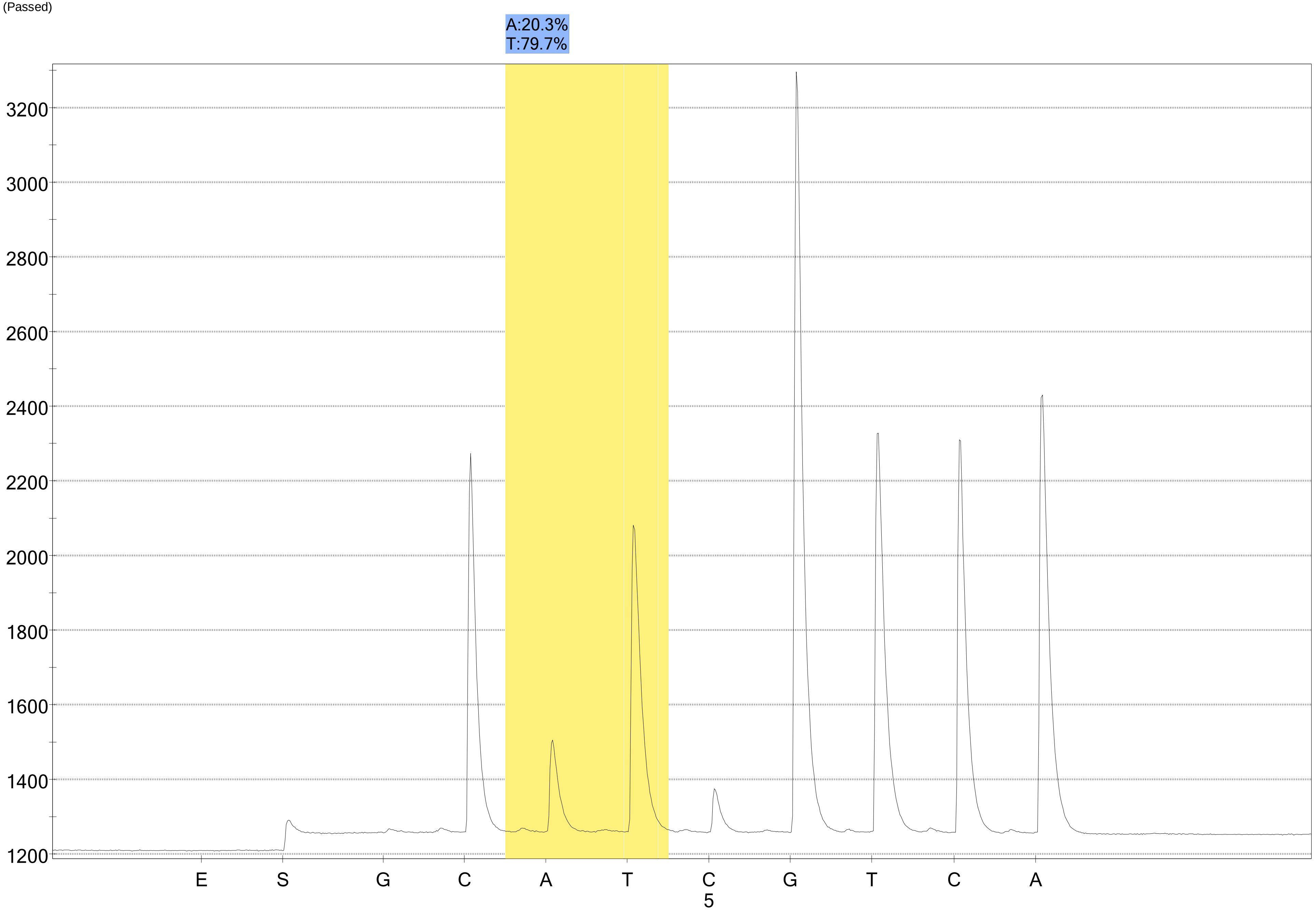

dna - Well G11
Entry: Rasgrf1
2: A: 20.4% / T: 79.6%
(Passed)

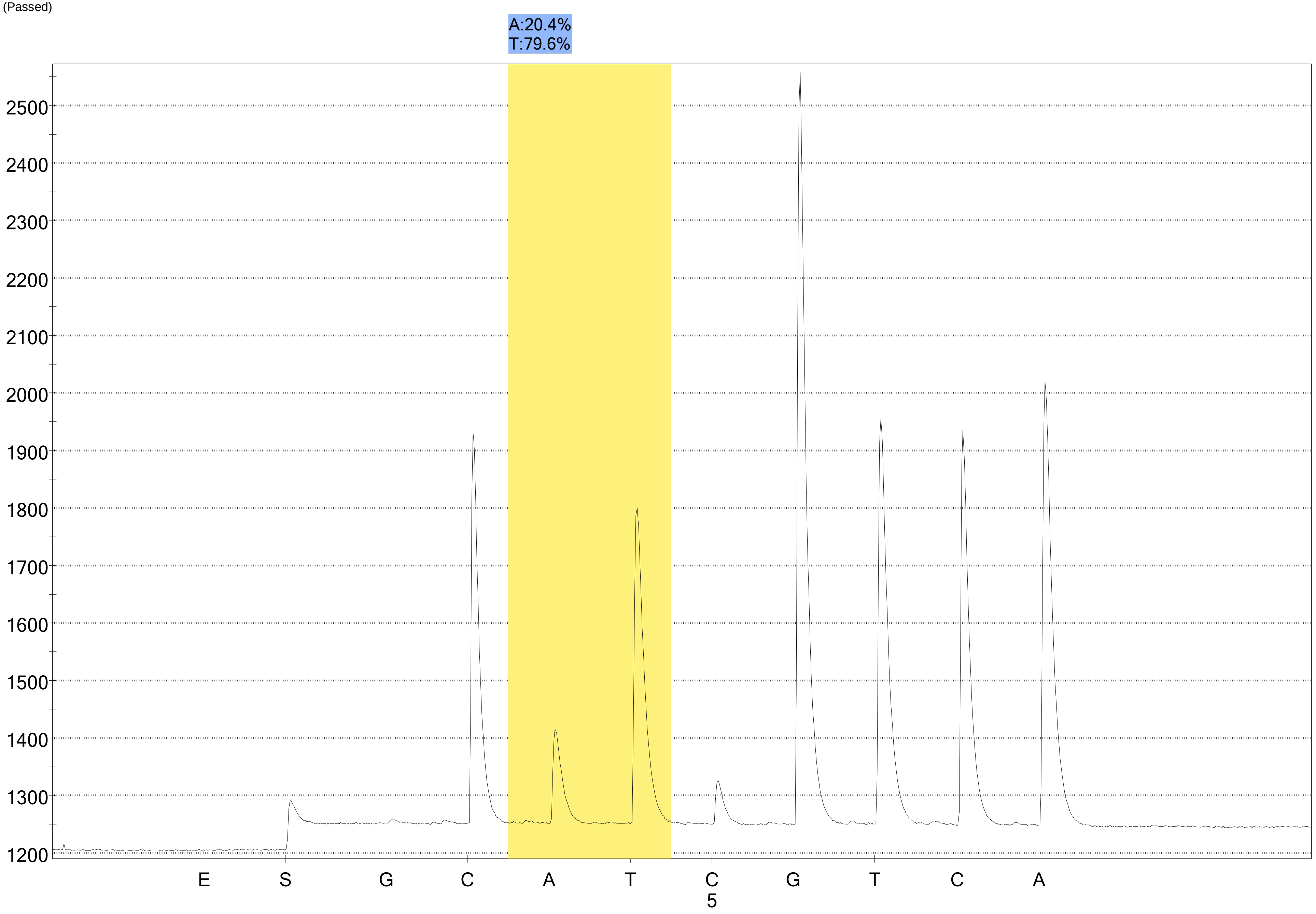

10 uL universal (141+157) - Well F3
Entry: Frat3
1: A: 91.1% / C: 8.9%
(Passed)

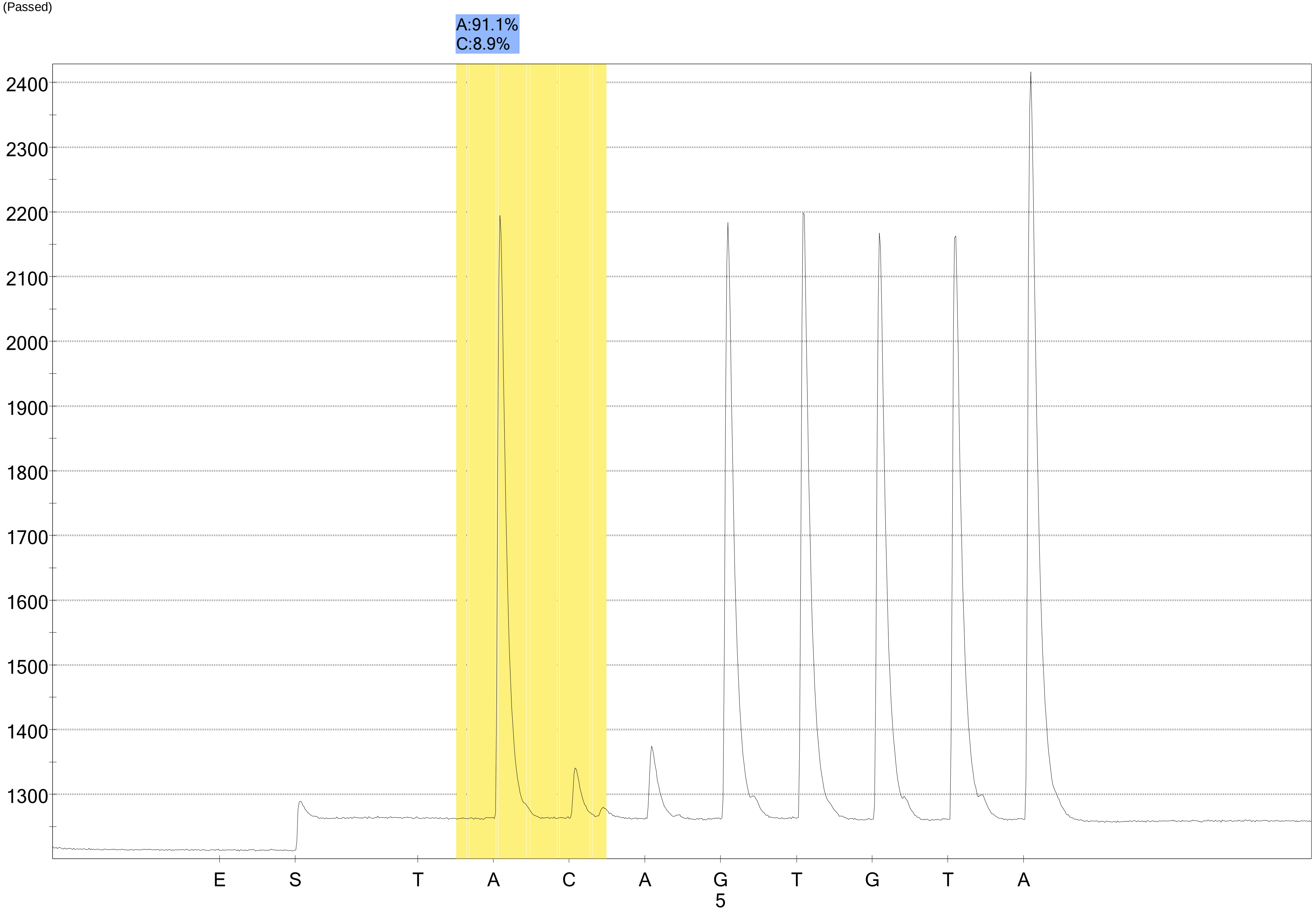

10 uL universal (141+157) - Well F9
Entry: Frat3
1: A: 4.7% / C: 95.3%
(Passed)

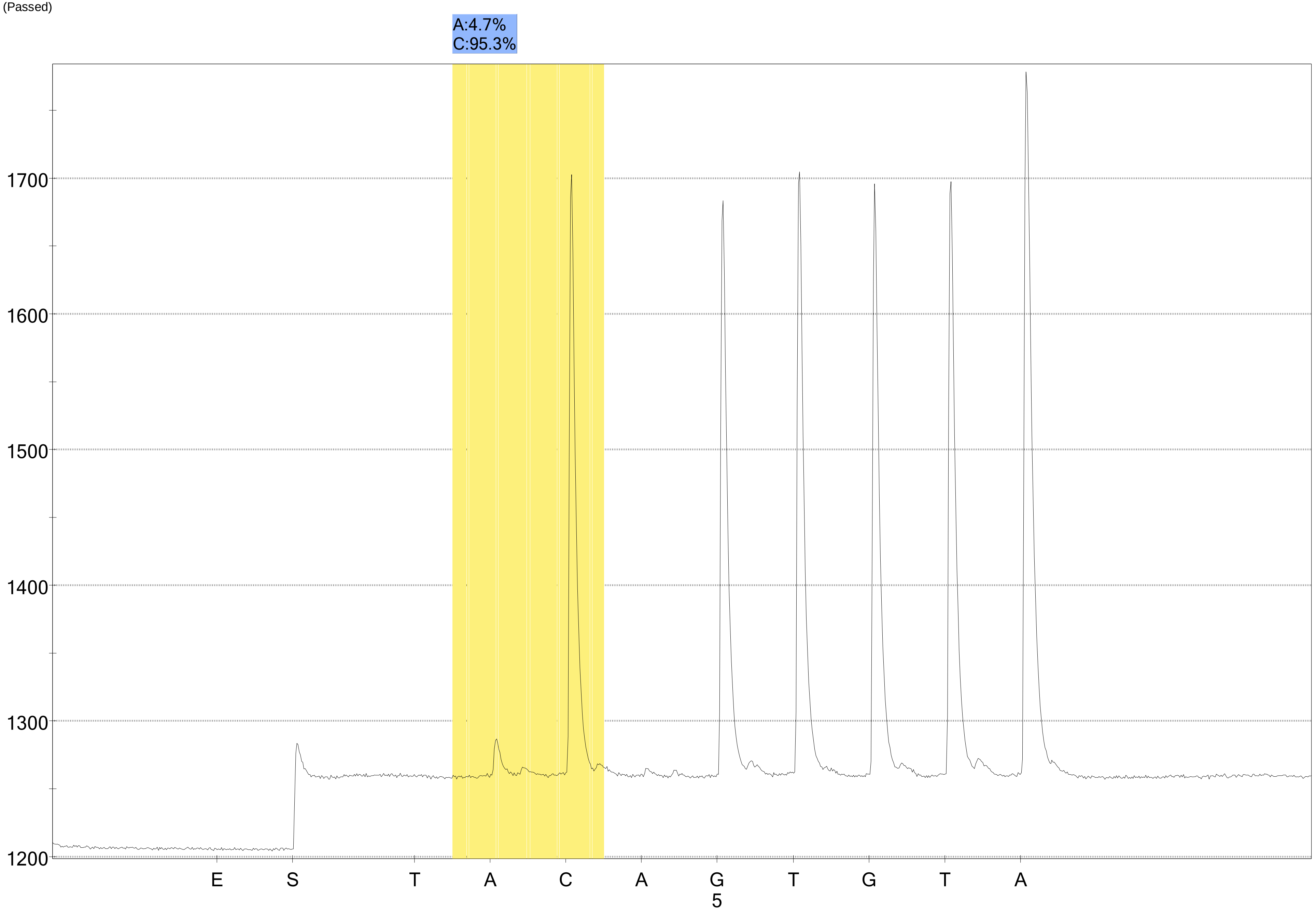

145 - Well F3
Entry: Frat3
1: A: 91.3% / C: 8.7%
(Passed)

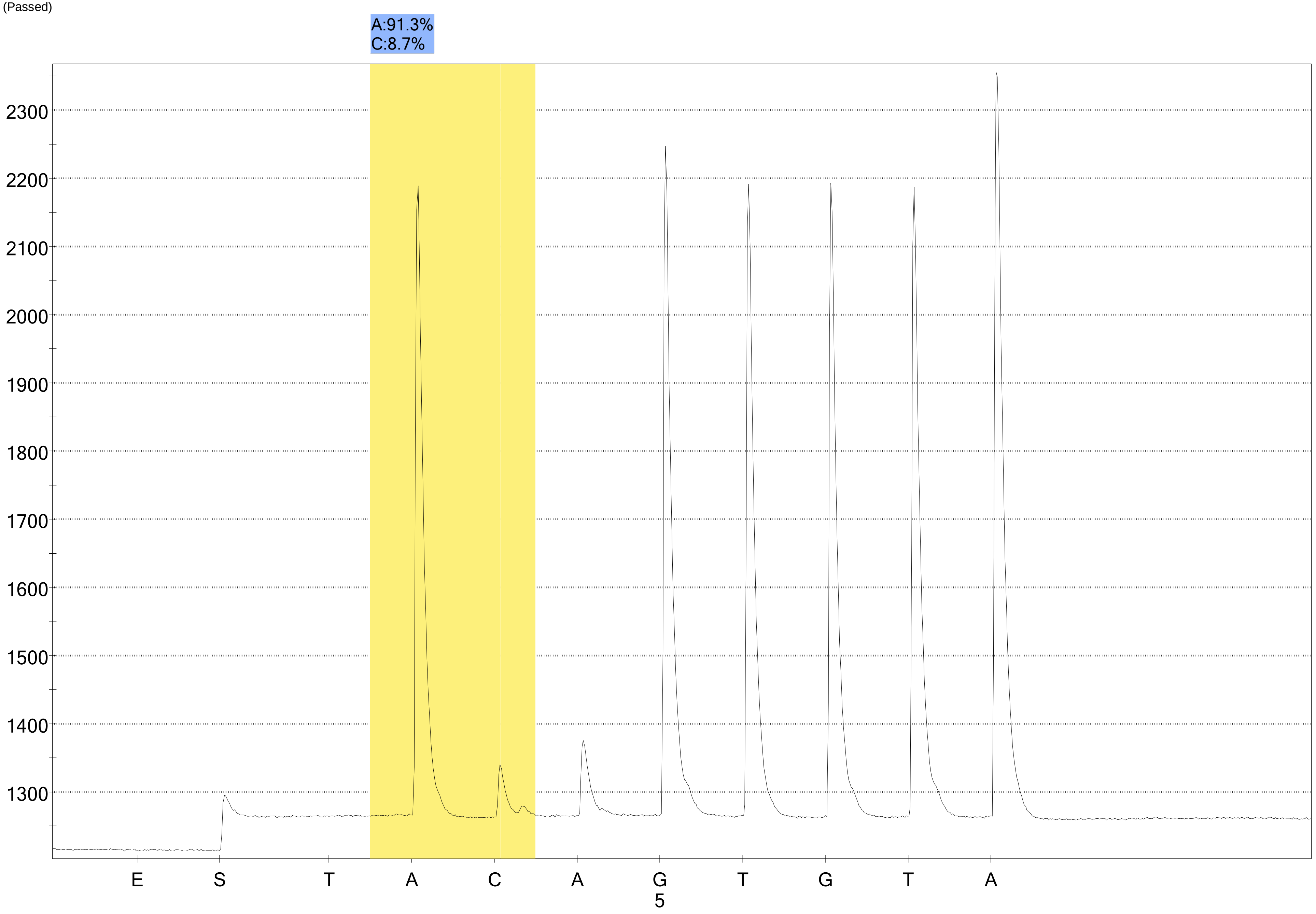

dna - Well F3
Entry: Frat3
1: A: 53.6% / C: 46.4%
(Passed)

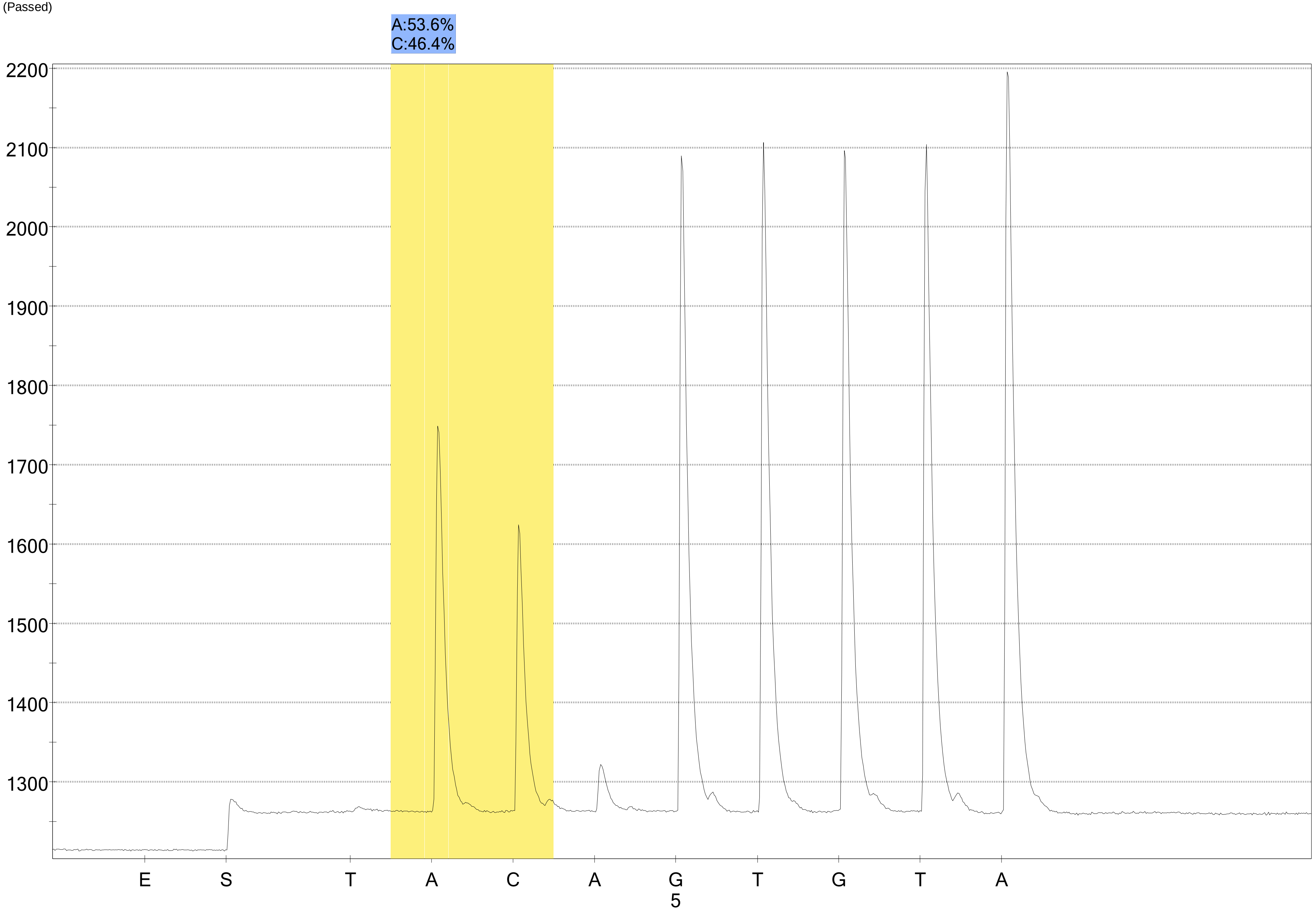

dna - Well F9
Entry: Frat3
1: A: 50.9% / C: 49.1%
(Passed)

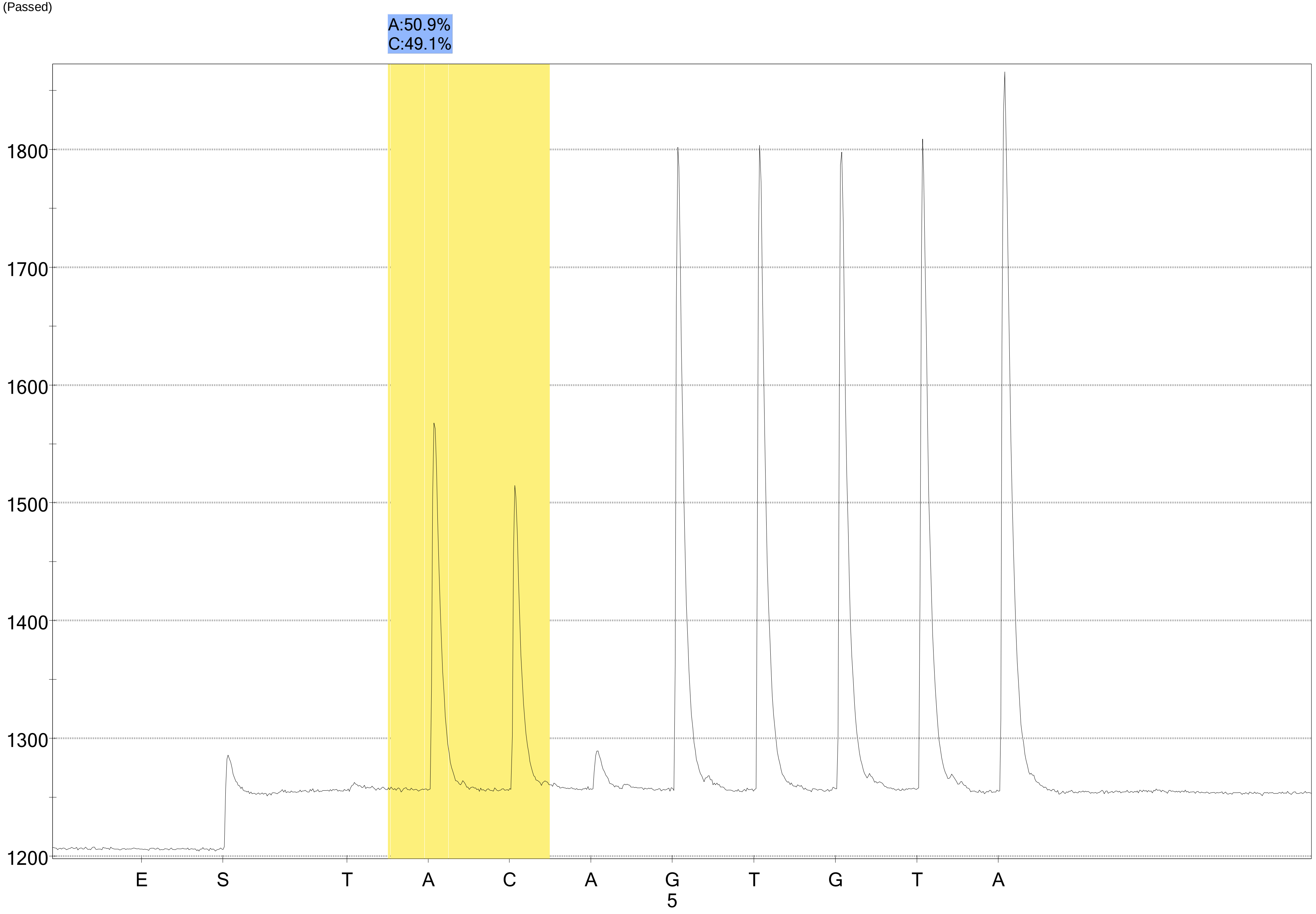

10 uL universal (141+157) - Well G3
Entry: Gnas
1: C: 1.7% / T: 98.3%
(Passed)

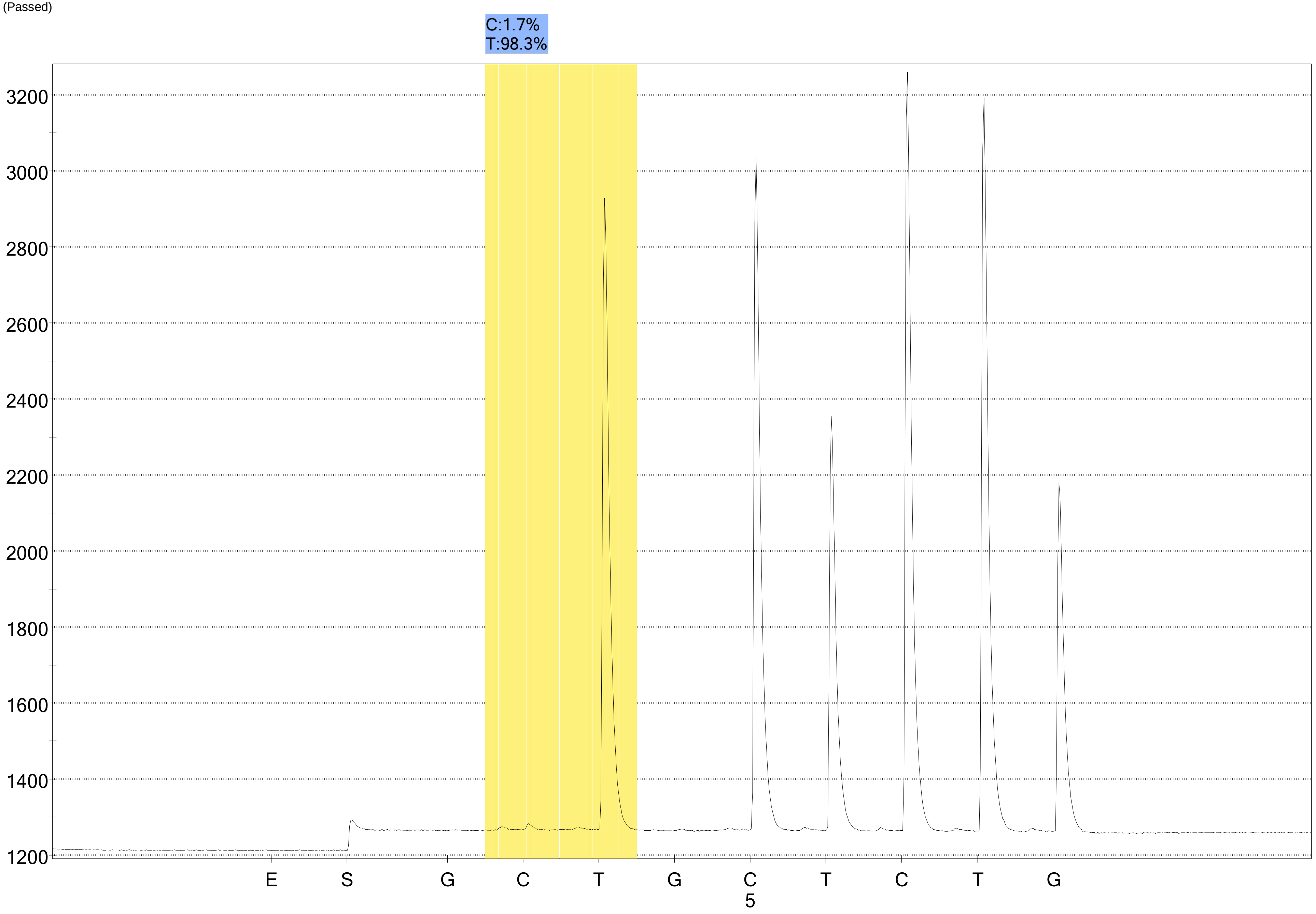

10 uL universal (141+157) - Well G9
Entry: Gnas
1: C: 95.9% / T: 4.1%
(Passed)

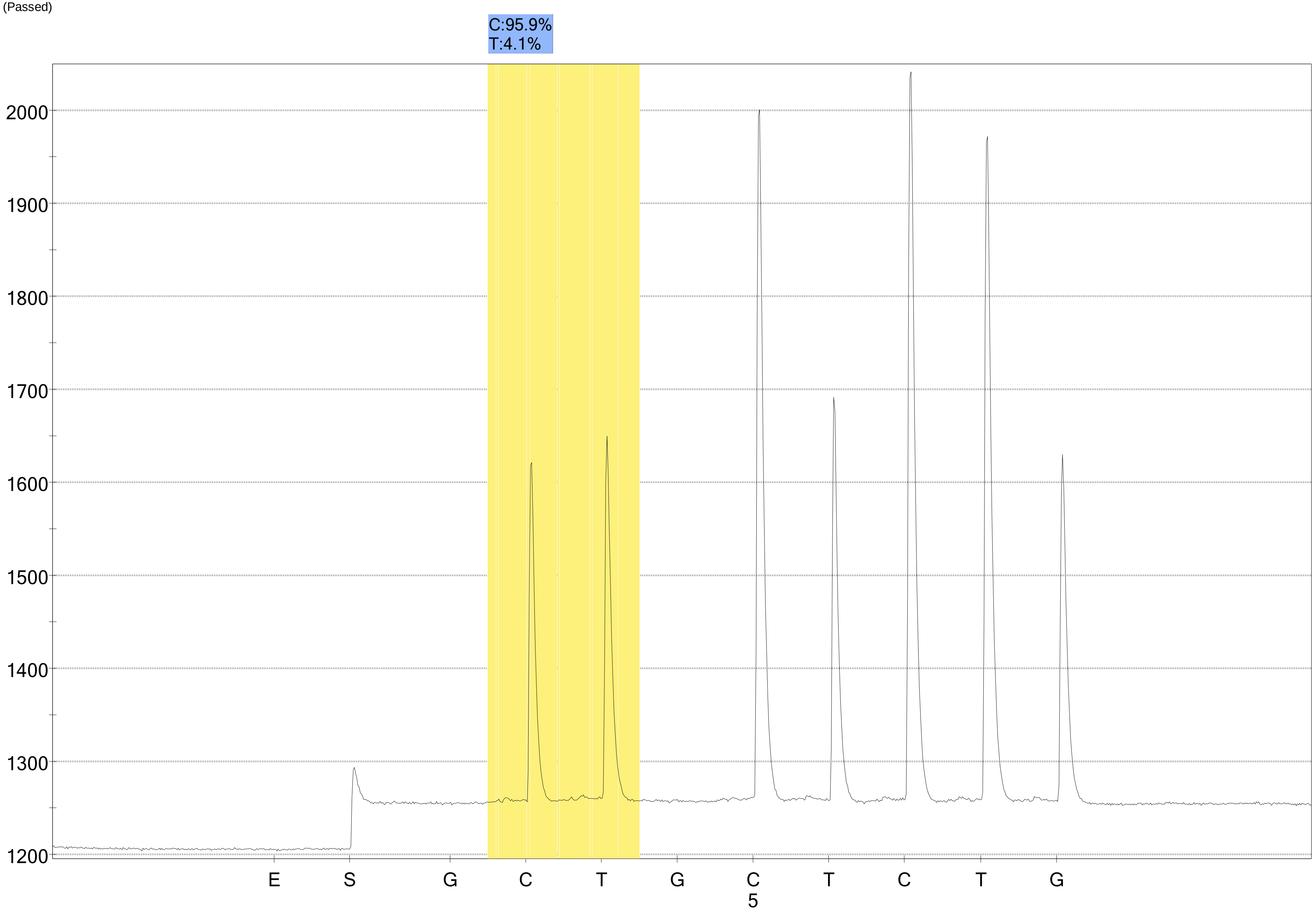

145 - Well G3
Entry: Gnas
1: C: 2.1% / T: 97.9%
(Passed)

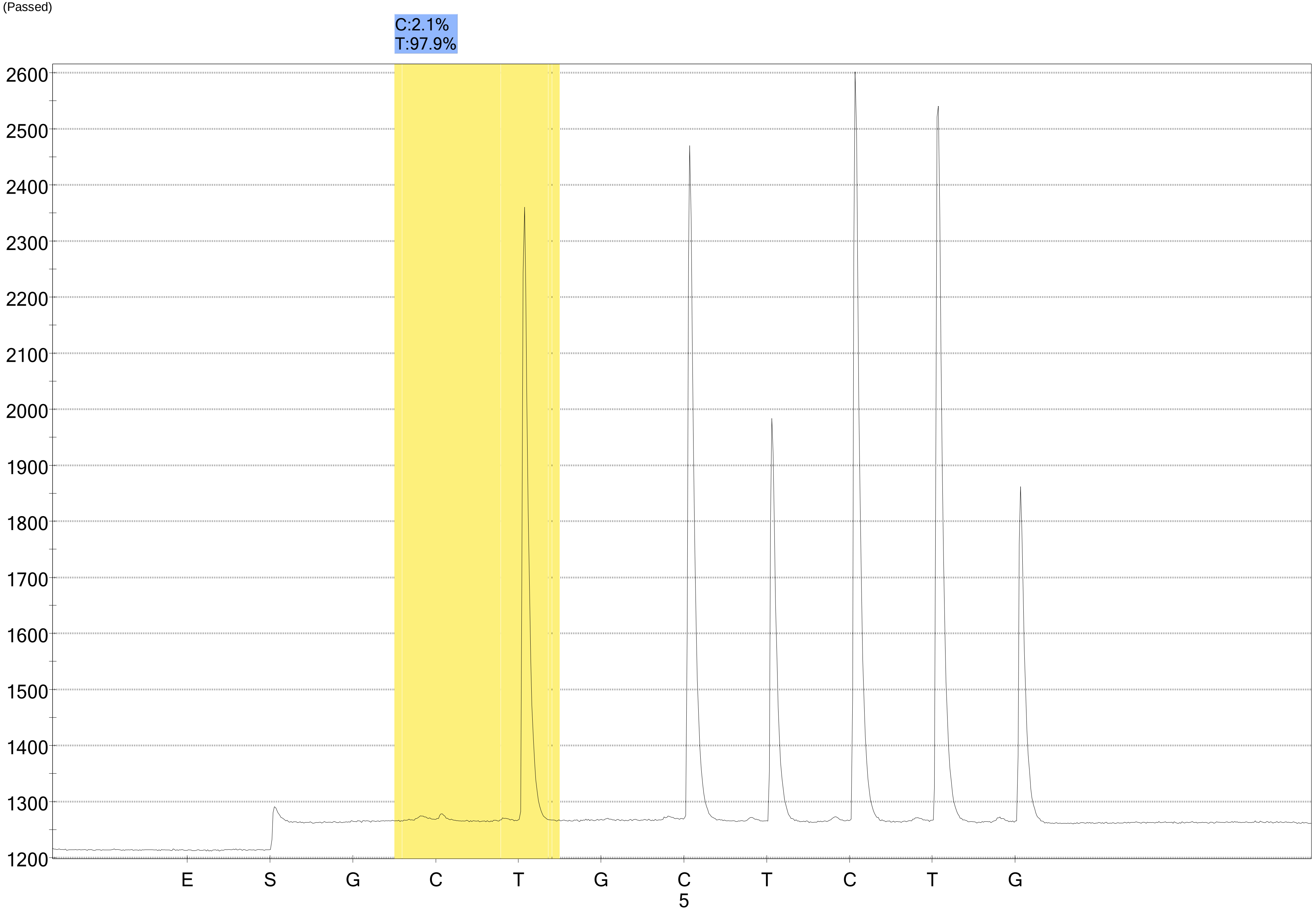

dna - Well G3
Entry: Gnas
1: C: 50.8% / T: 49.2%
(Passed)

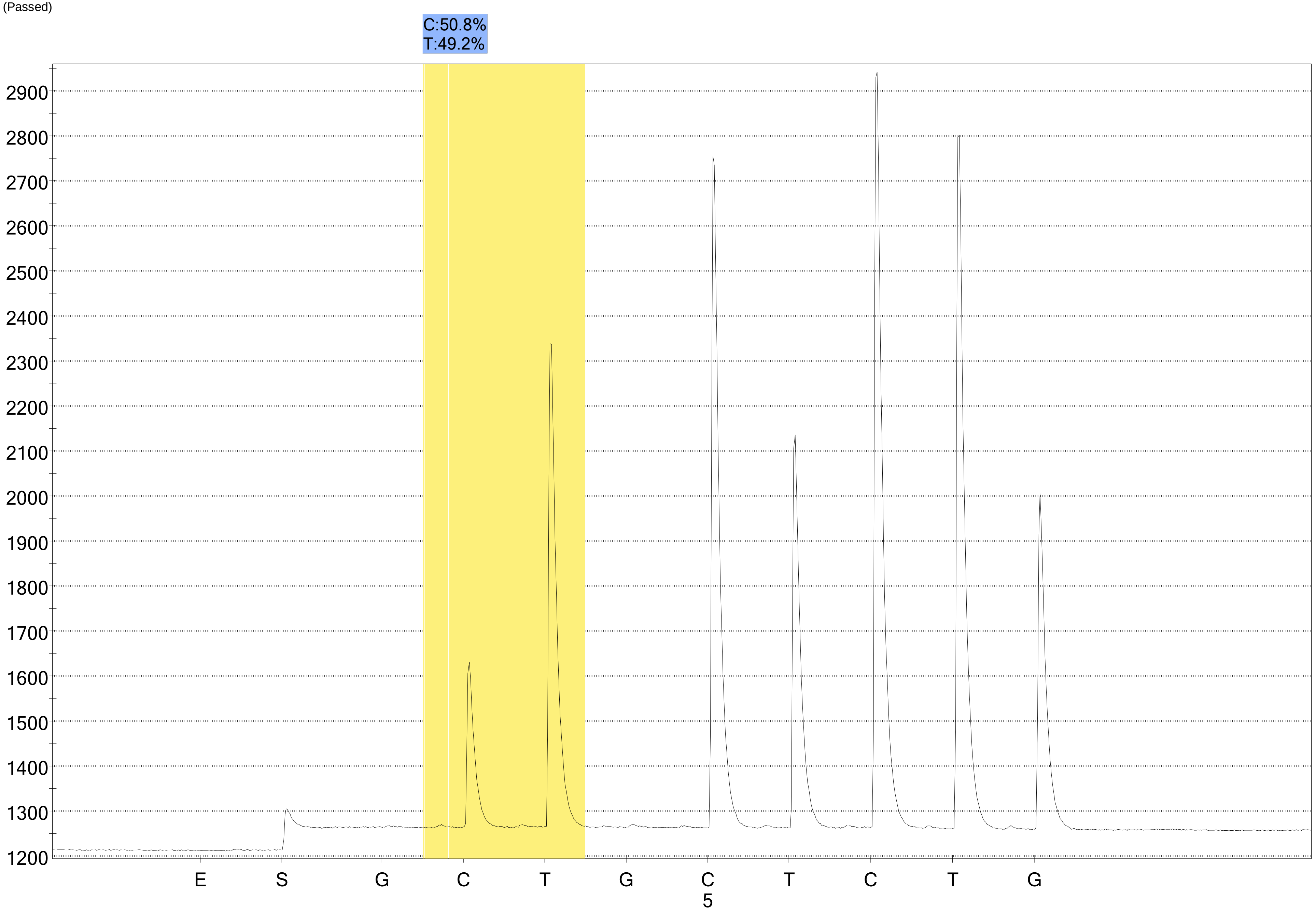

dna - Well G9
Entry: Gnas
1: C: 48.3% / T: 51.7%
(Passed)

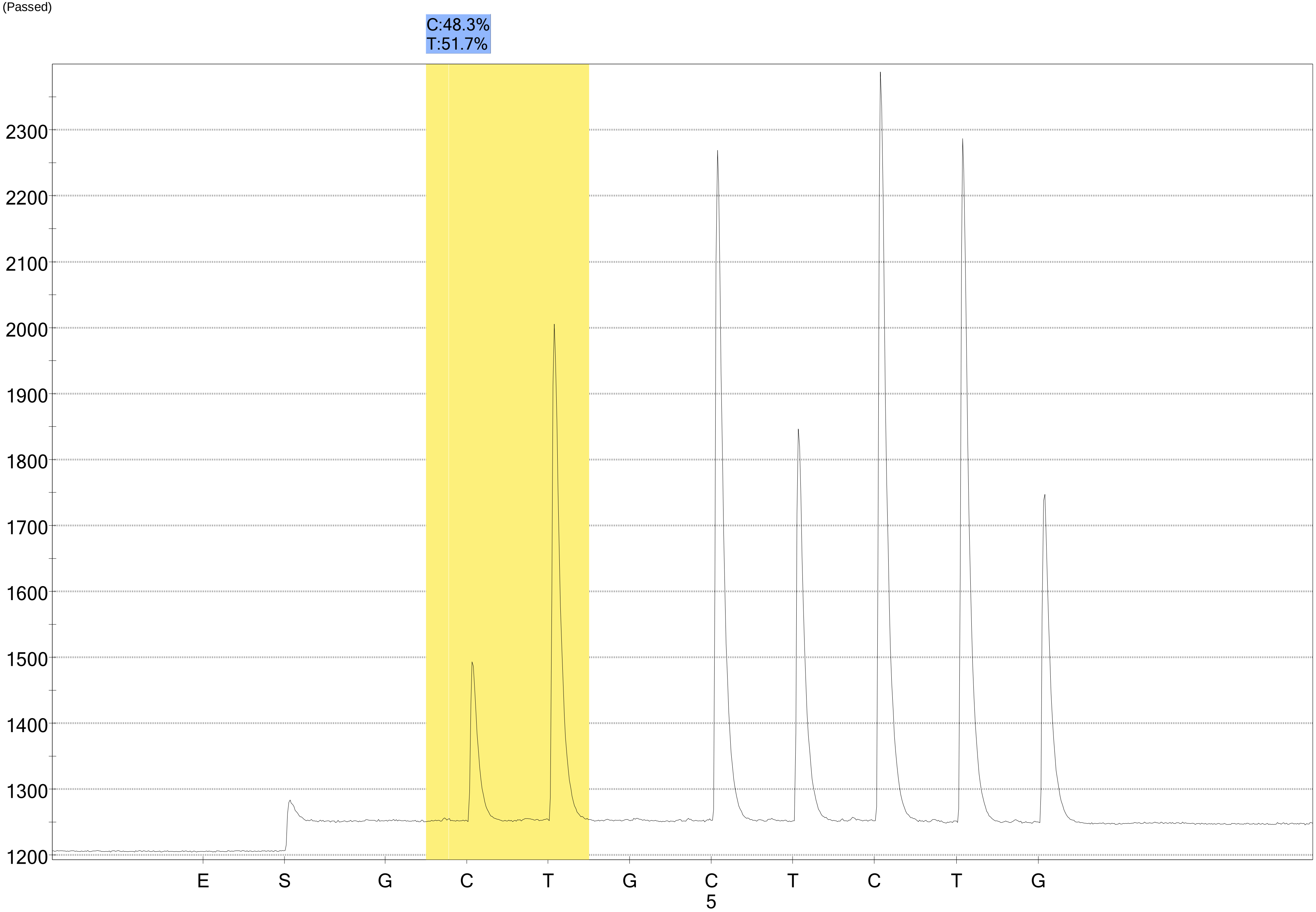

10 uL universal (141+157) - Well C3
Entry: DOKist4
1: T: 97.1% / C: 2.9%
(Passed)

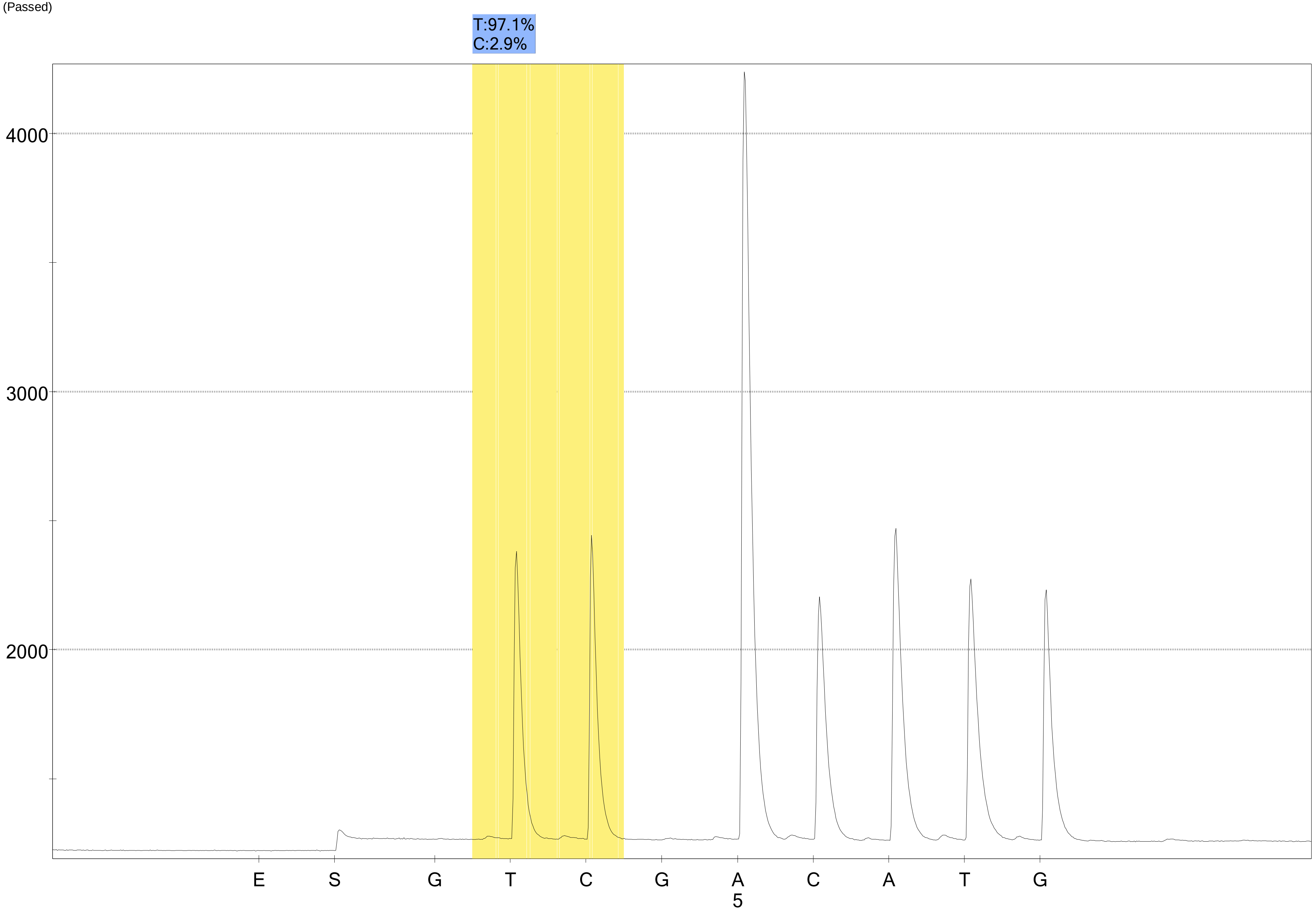

10 uL universal (141+157) - Well C9
Entry: DOKist4
1: T: 8.2% / C: 91.8%
(Passed)

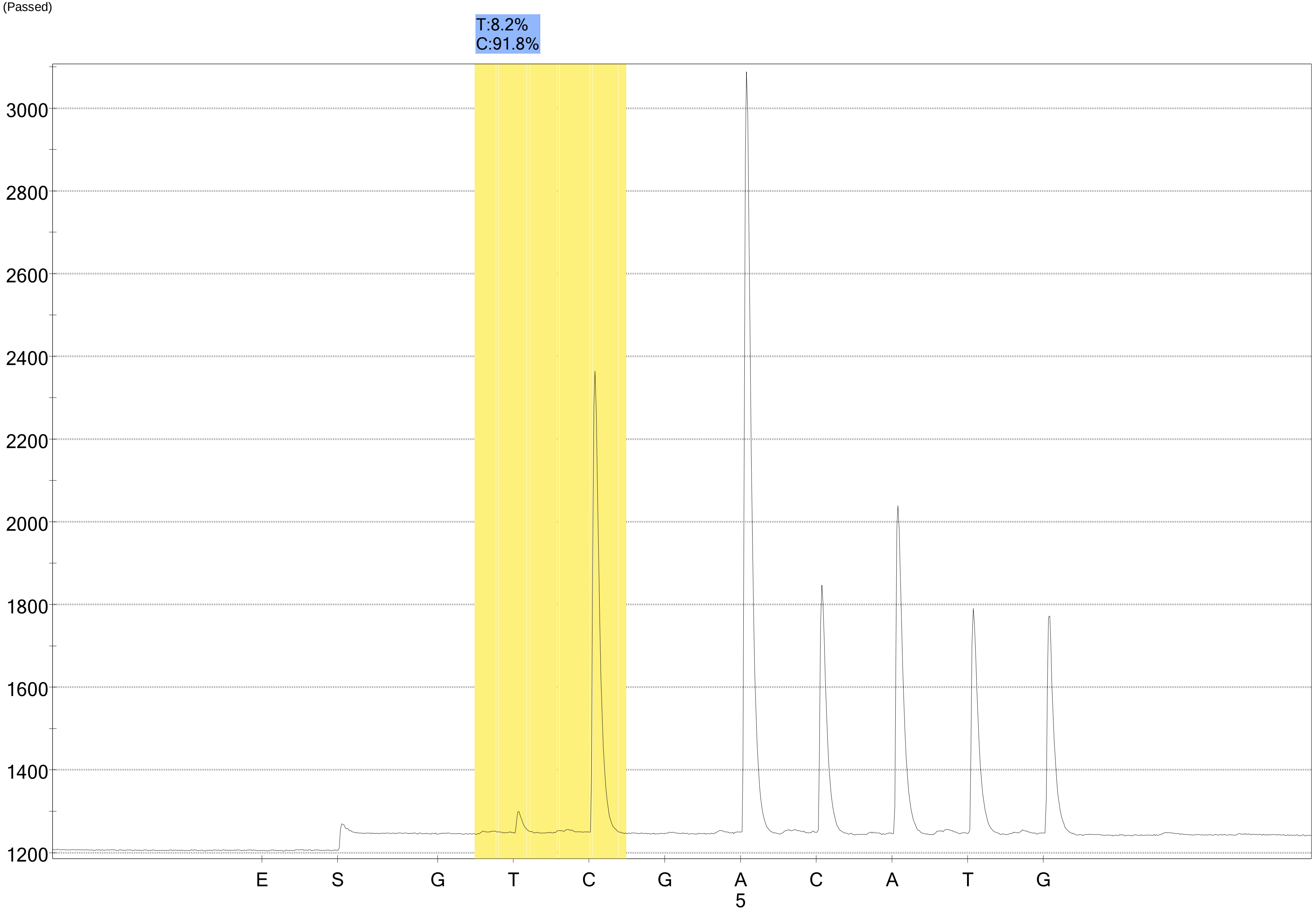

145 - Well C3
Entry: DOKist4
1: T: 98.8% / C: 1.2%
(Passed)

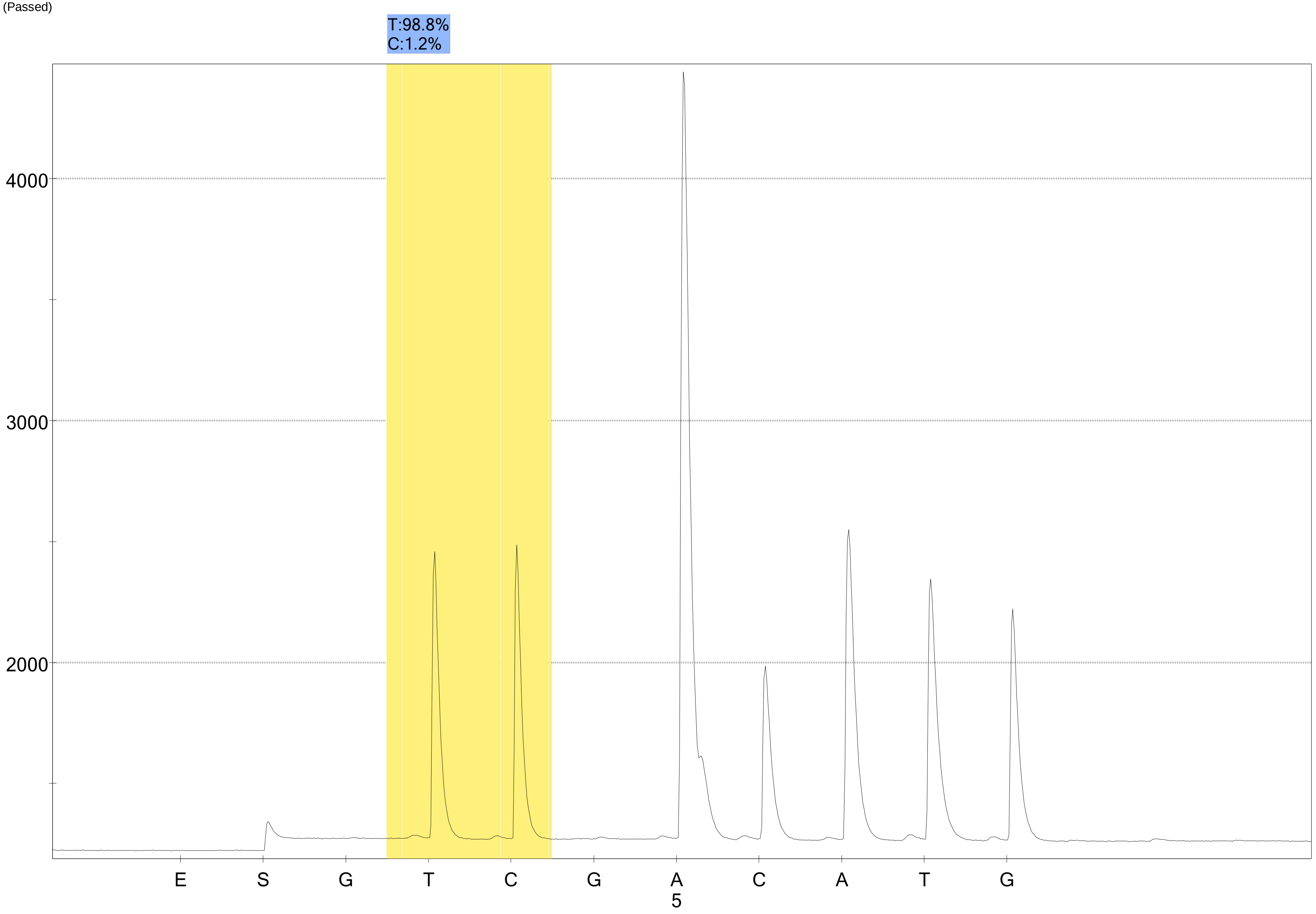

dna - Well C3
Entry: DOKist4
1: T: 44.1% / C: 55.9%
(Passed)

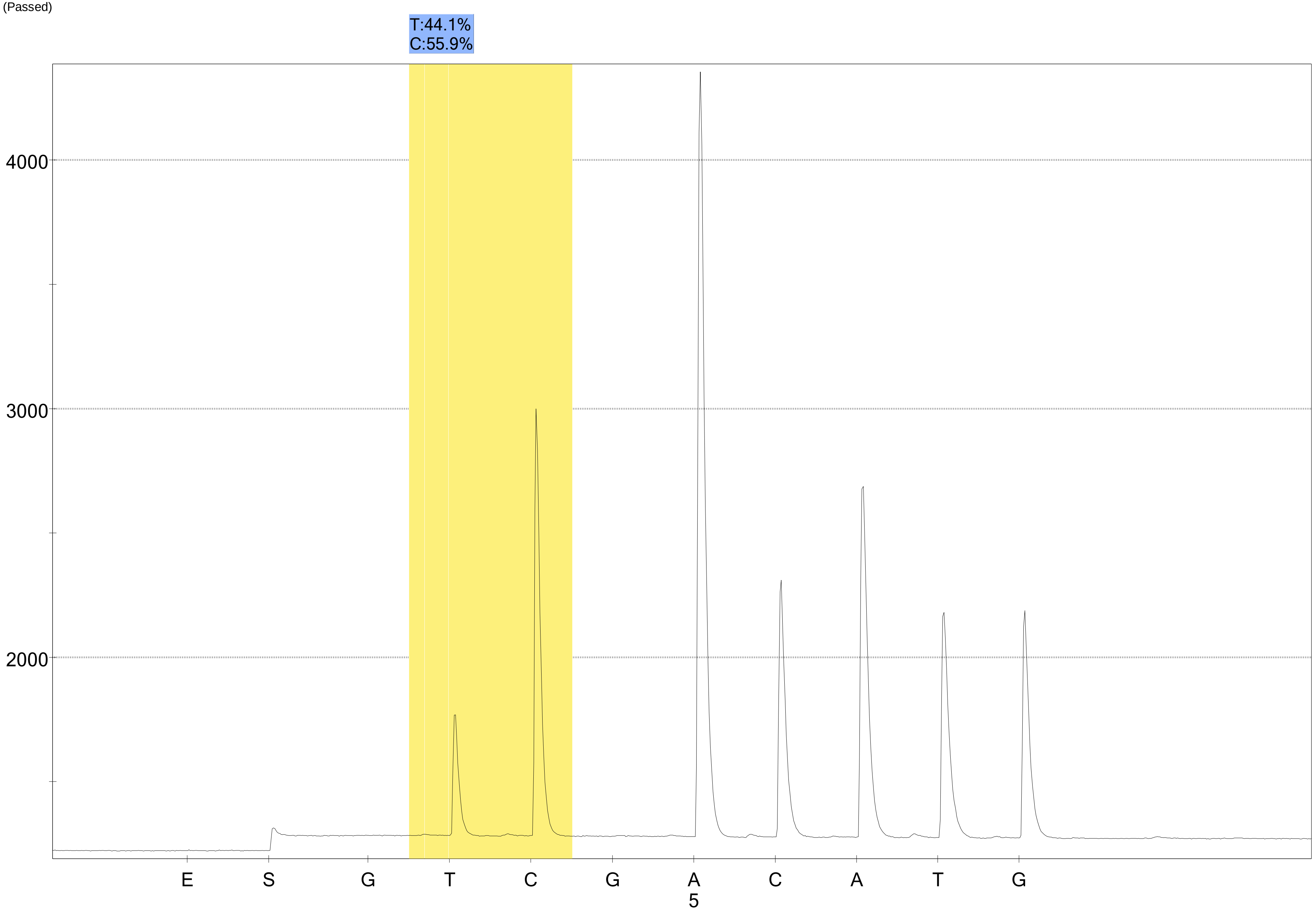

dna - Well C9
Entry: DOKist4
1: T: 45.2% / C: 54.8%
(Passed)

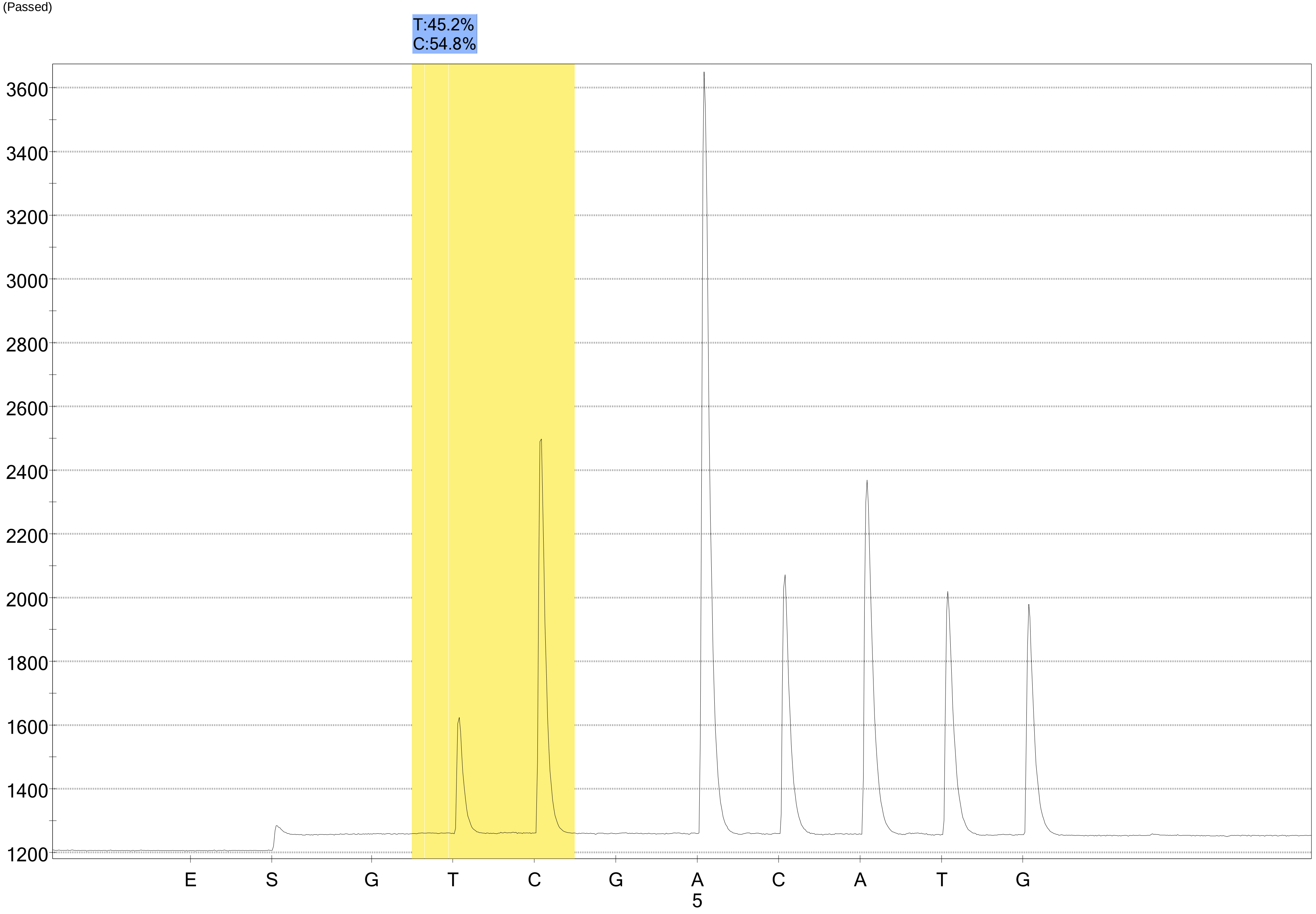

10 uL universal (141+157) - Well F6
Entry: U80893
3: A: 11.2% / G: 88.8%
(Passed)

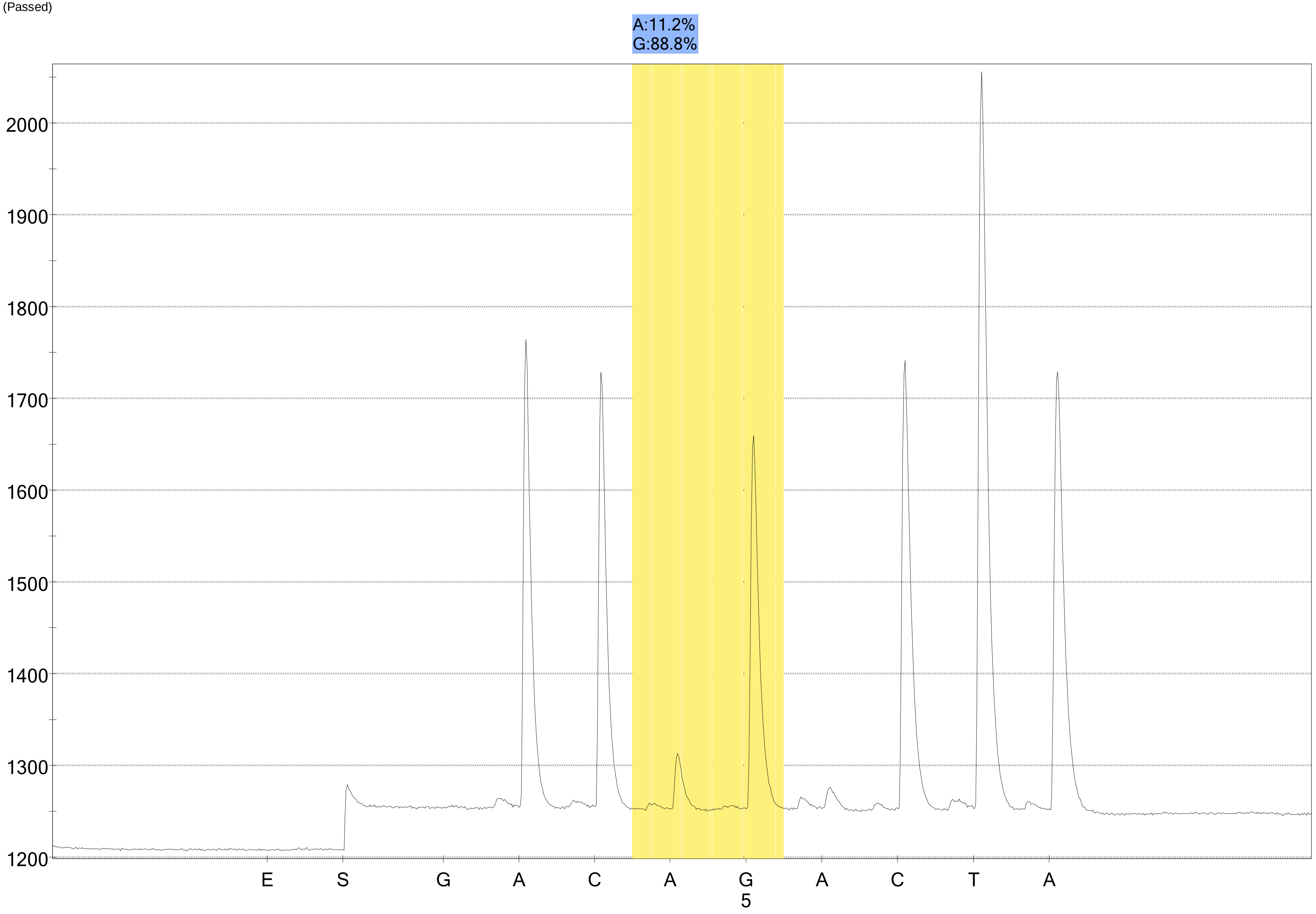

10 uL universal (141+157) - Well F12
Entry: U80893
3: A: 100.0% / G: 0.0%
(Passed)

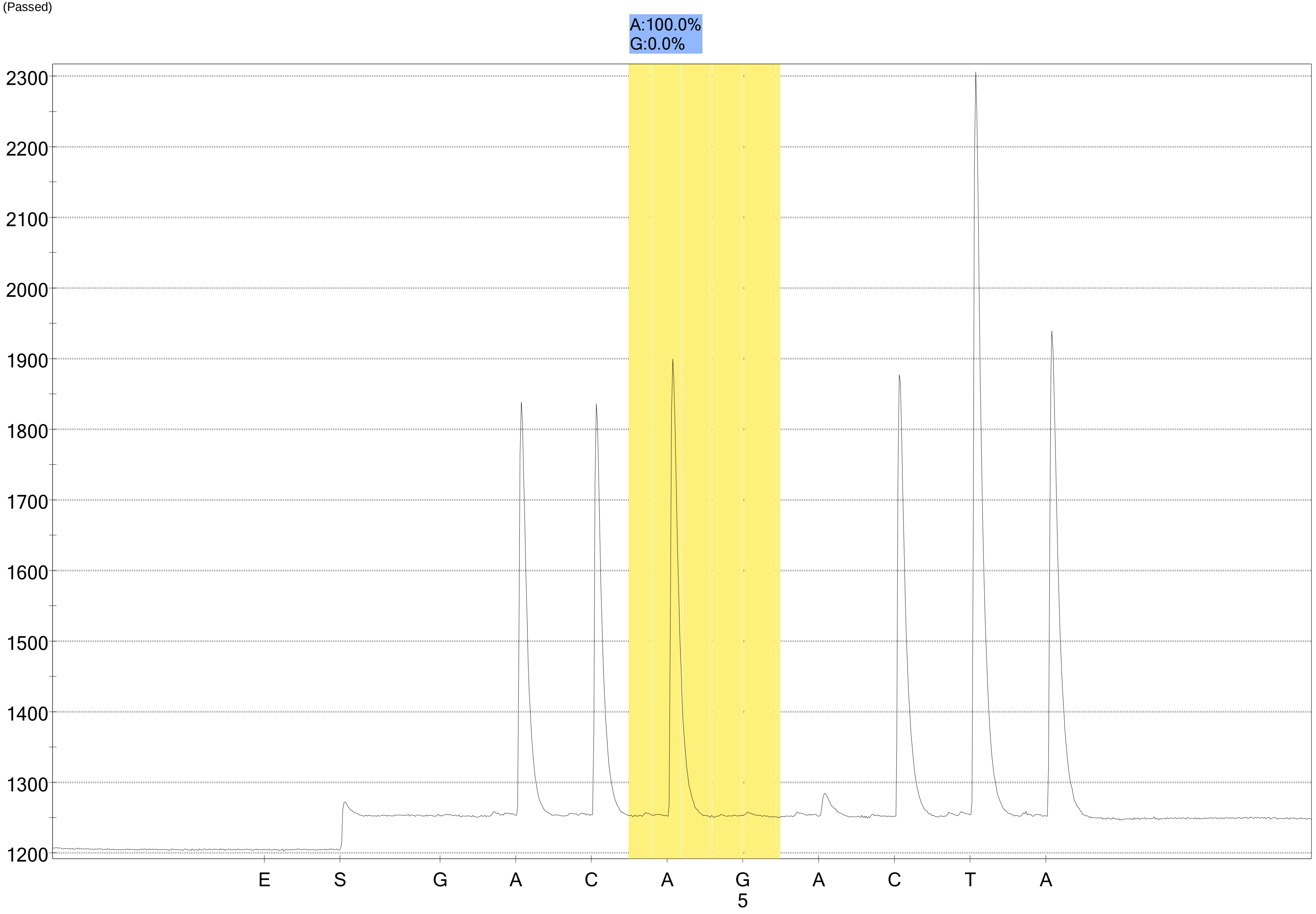

145 - Well F6
Entry: U80893
3: A: 11.3% / G: 88.7%
(Passed)

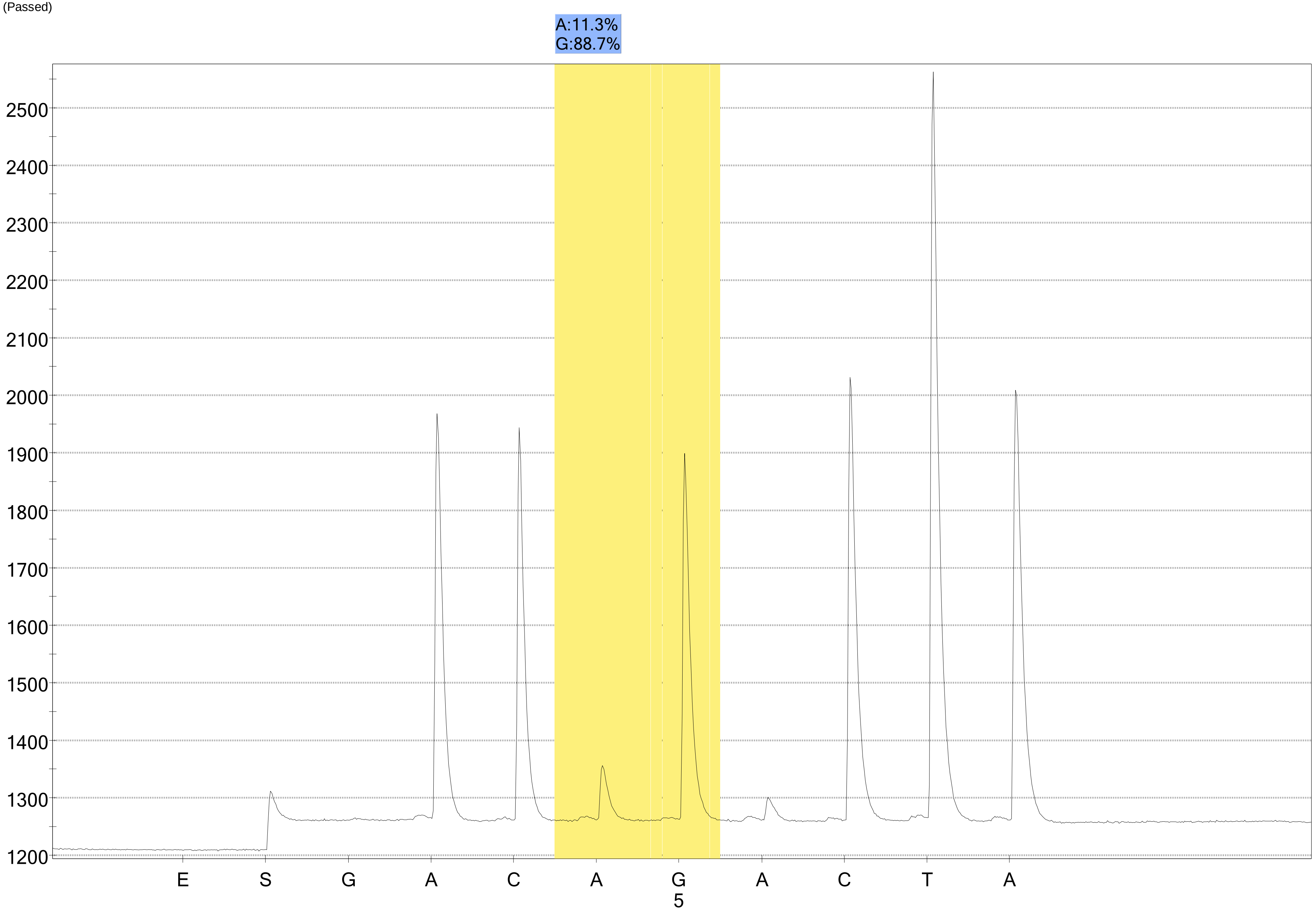

dna - Well F6
Entry: U80893
3: A: 57.1% / G: 42.9%
(Passed)

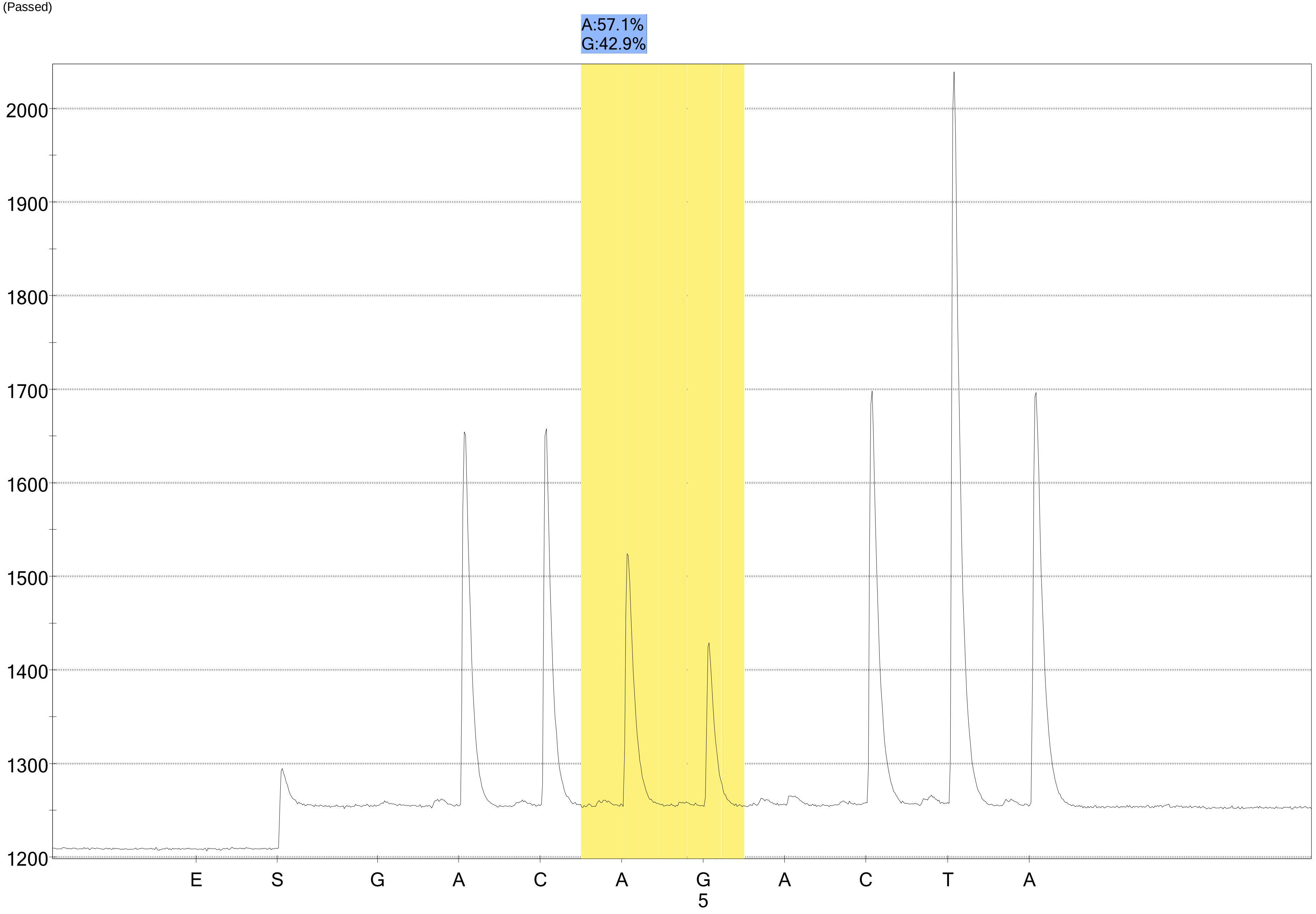

dna - Well F12
Entry: U80893
3: A: 53.6% / G: 46.4%
(Passed)

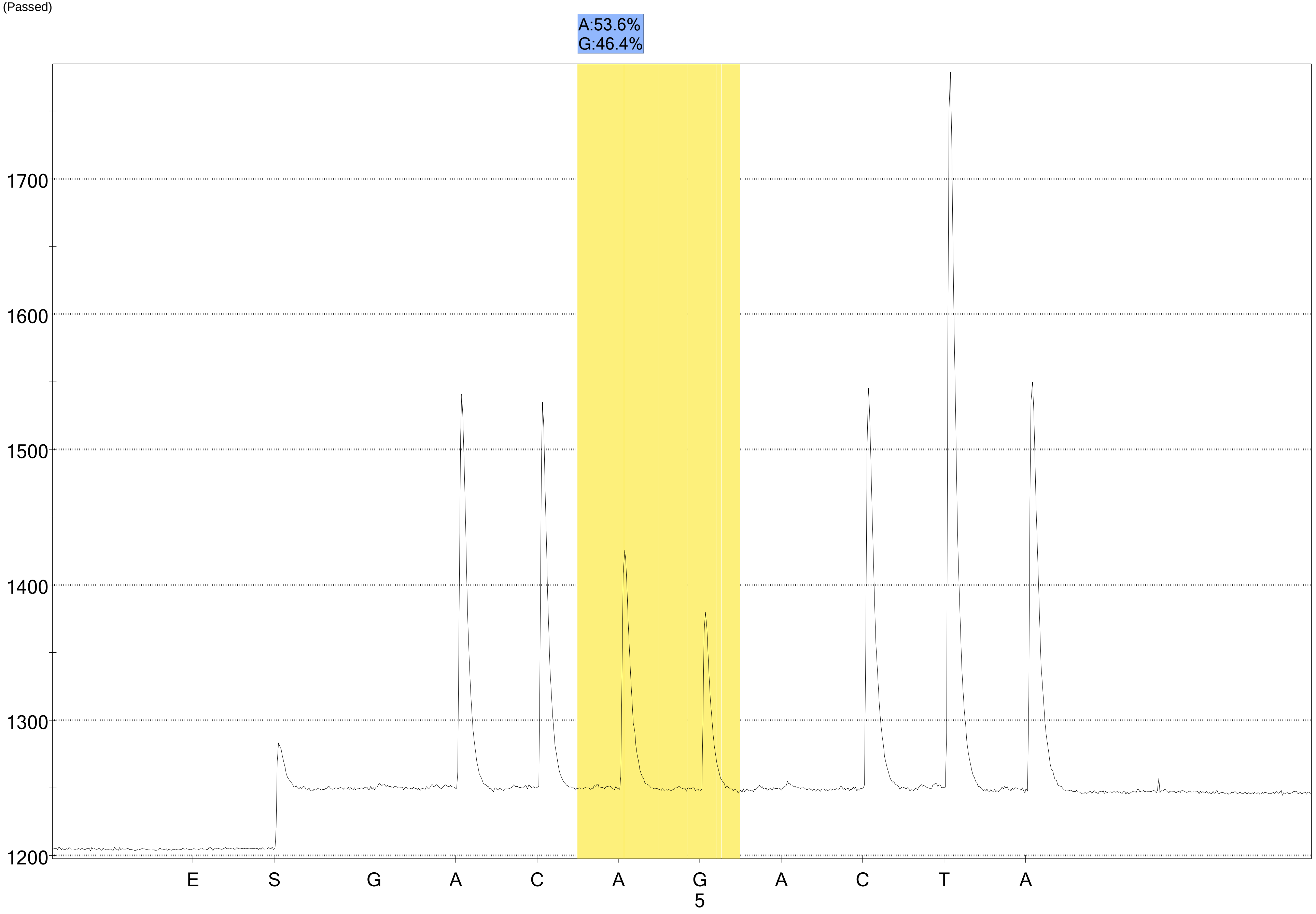

10 uL universal (141+157) - Well C2
Entry: C230091D08Rik
1: G: 42.4% / A: 57.6%
(Passed)

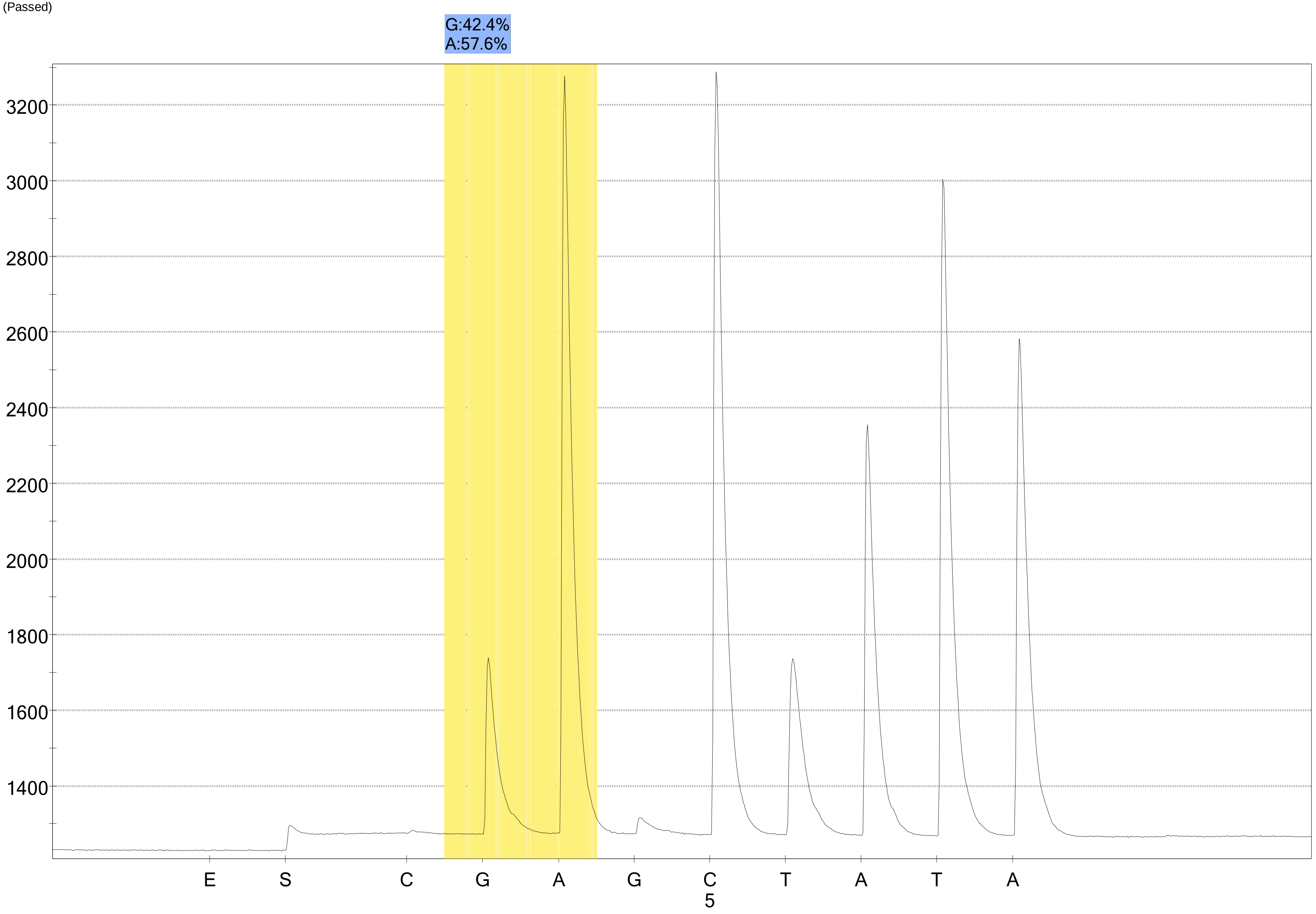

10 uL universal (141+157) - Well C8
Entry: C230091D08Rik
1: G: 43.2% / A: 56.8%
(Passed)

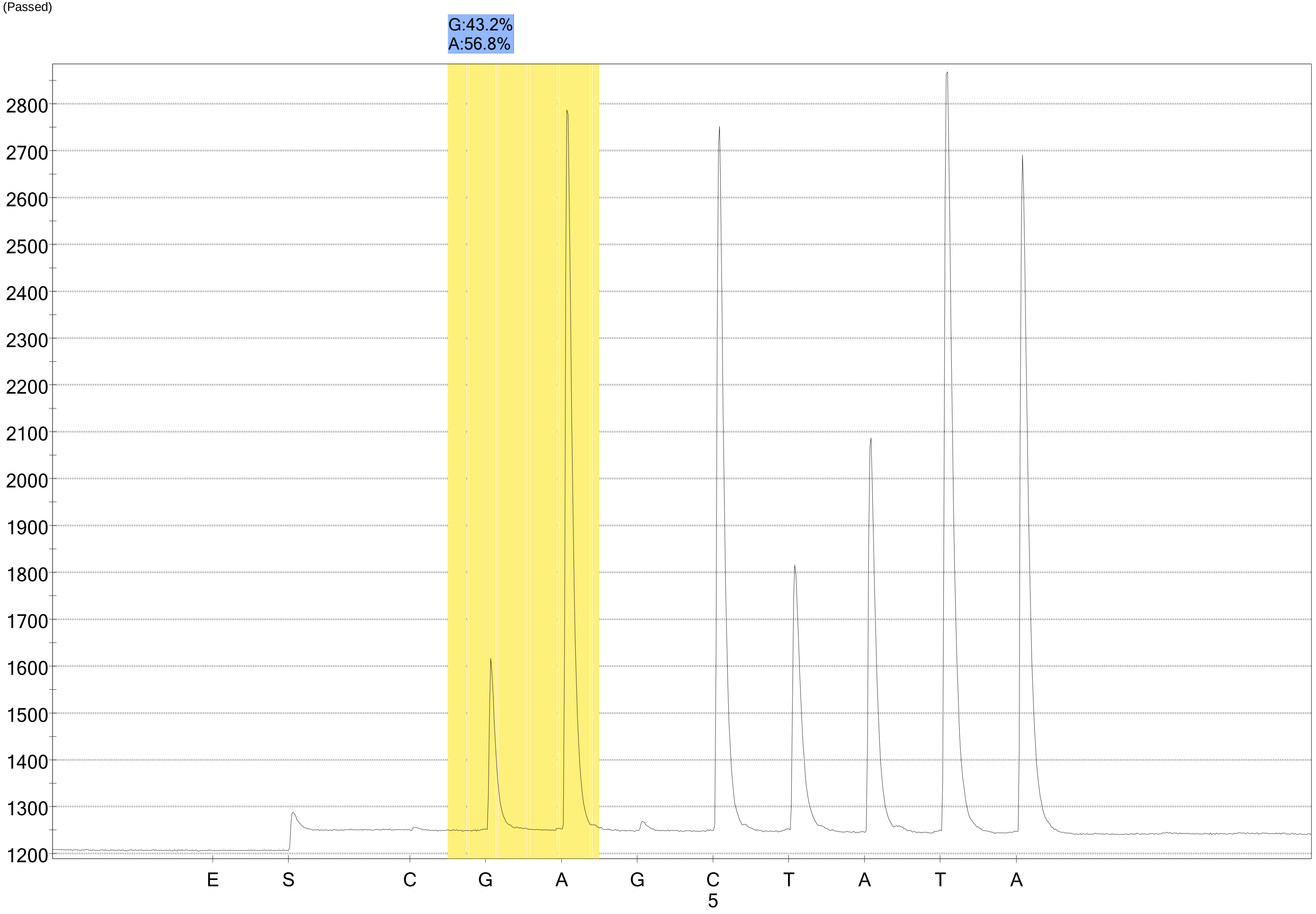

145 - Well C2
Entry: C230091D08Rik
1: G: 45.1% / A: 54.9%
(Passed)

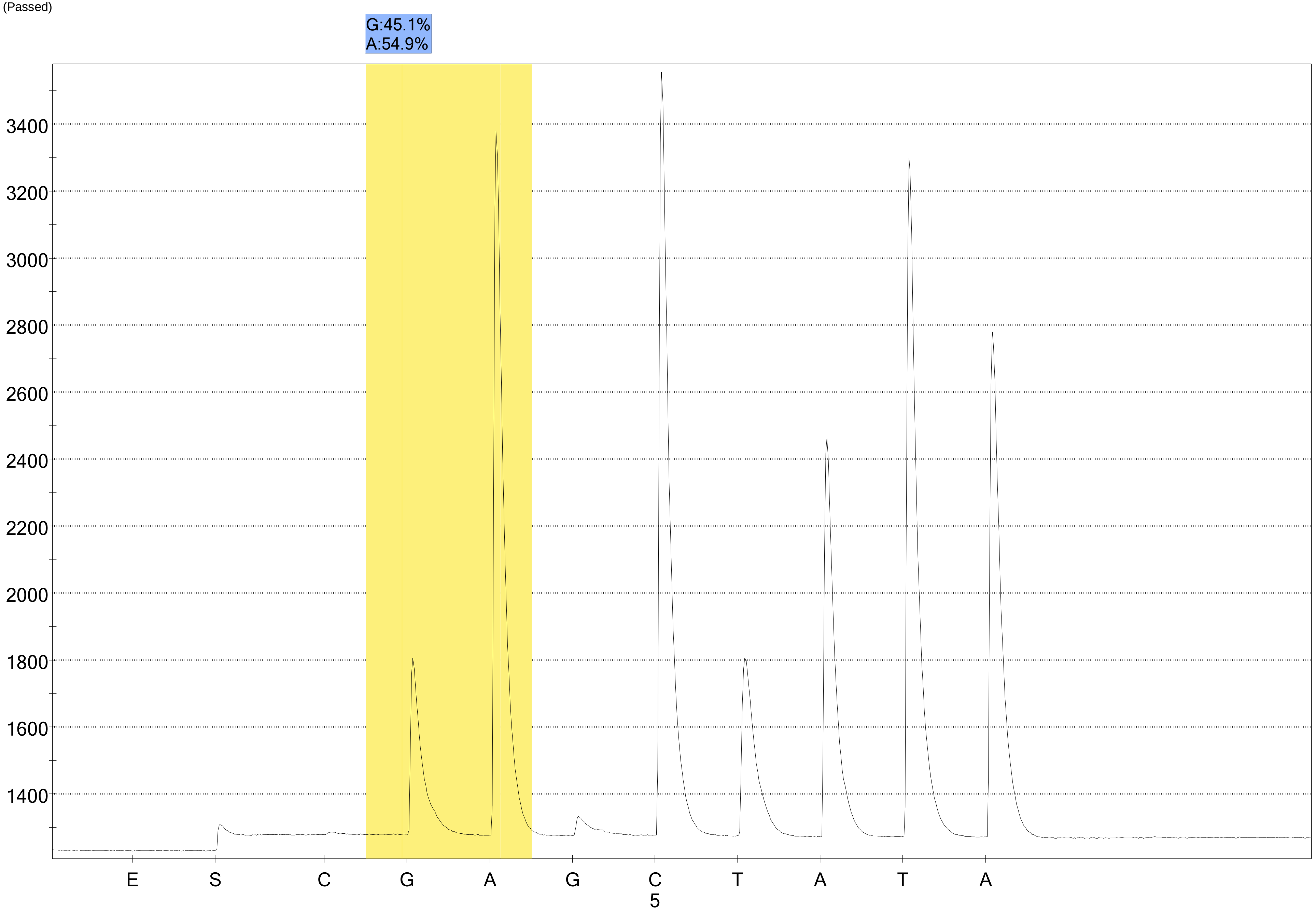

dna - Well C2
Entry: C230091D08Rik
1: G: 51.6% / A: 48.4%
(Passed)

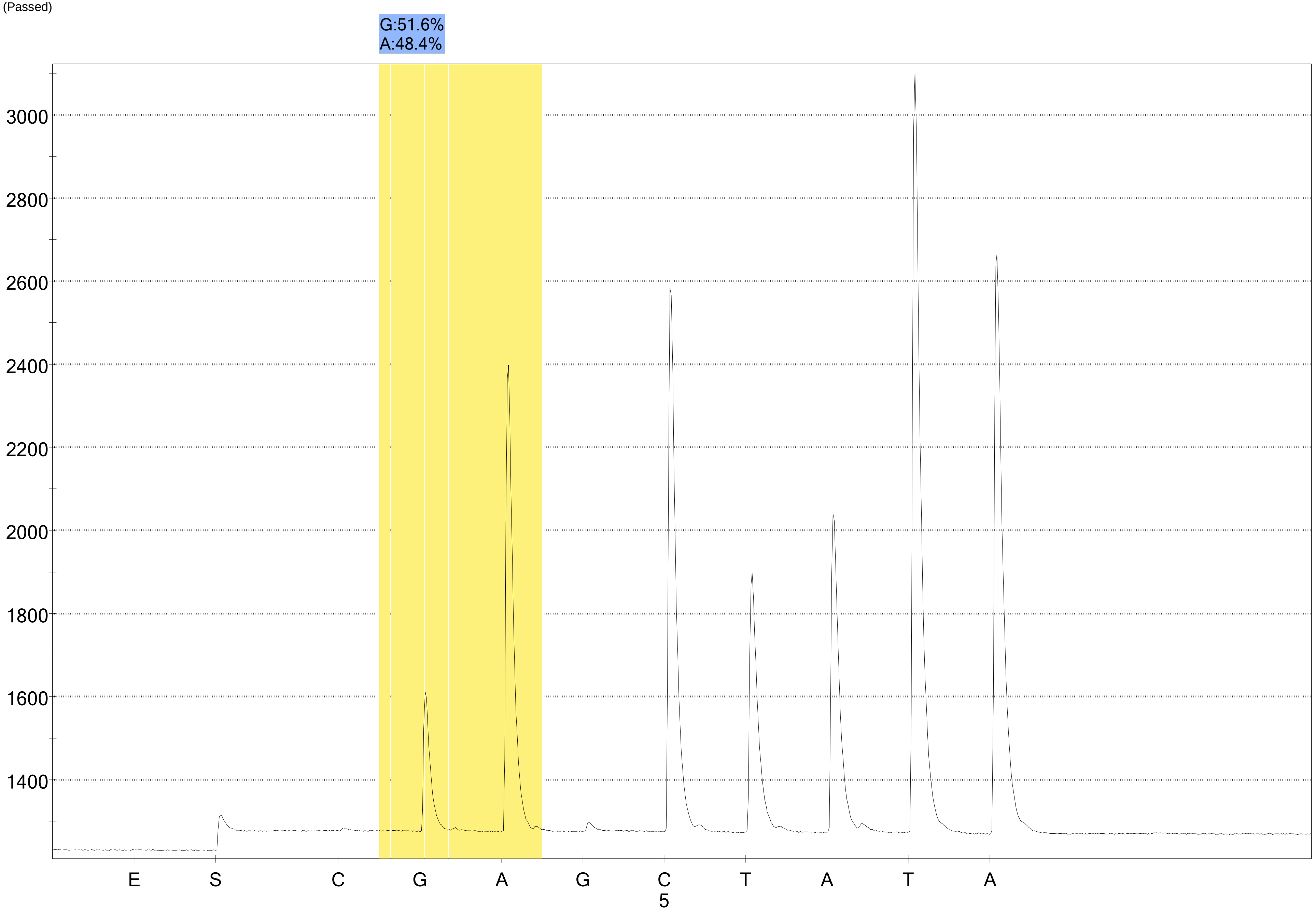

dna - Well C8
Entry: C230091D08Rik
1: G: 45.2% / A: 54.8%
(Passed)

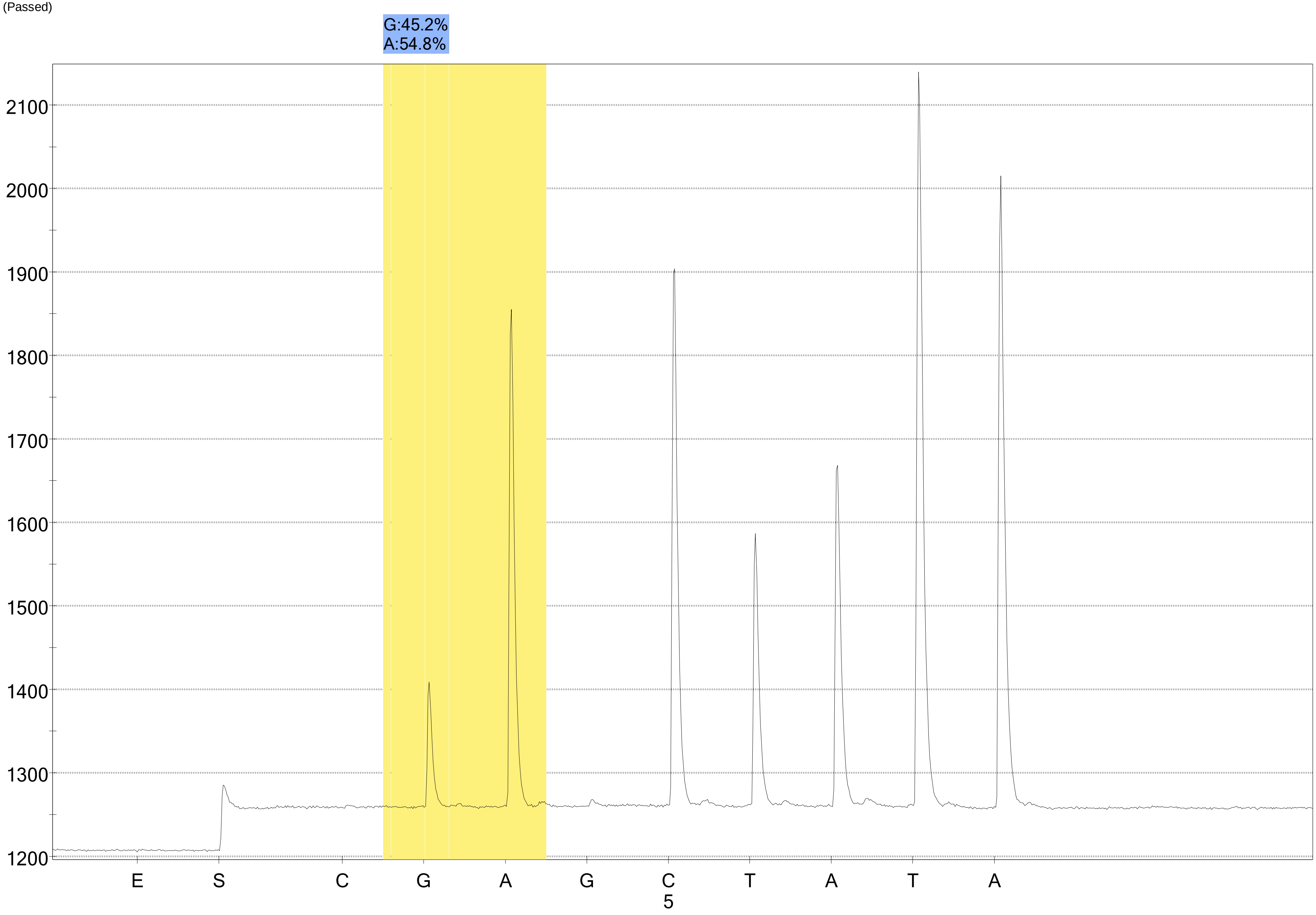

10 uL universal (141+157) - Well E4
Entry: Limd1
1: A: 67.0% / G: 33.0%
(Passed)

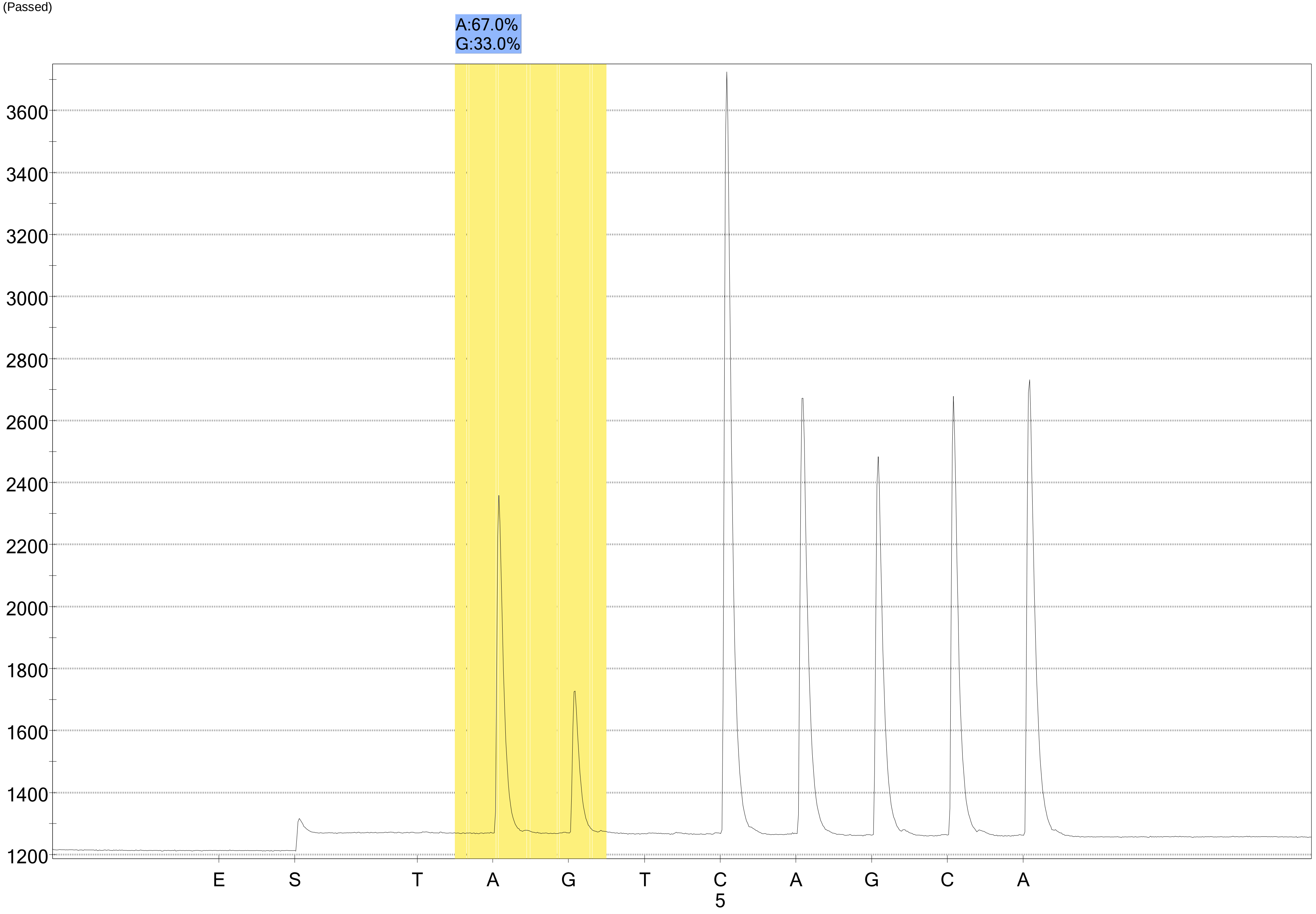

10 uL universal (141+157) - Well E10
Entry: Limd1
1: A: 63.8% / G: 36.2%
(Passed)

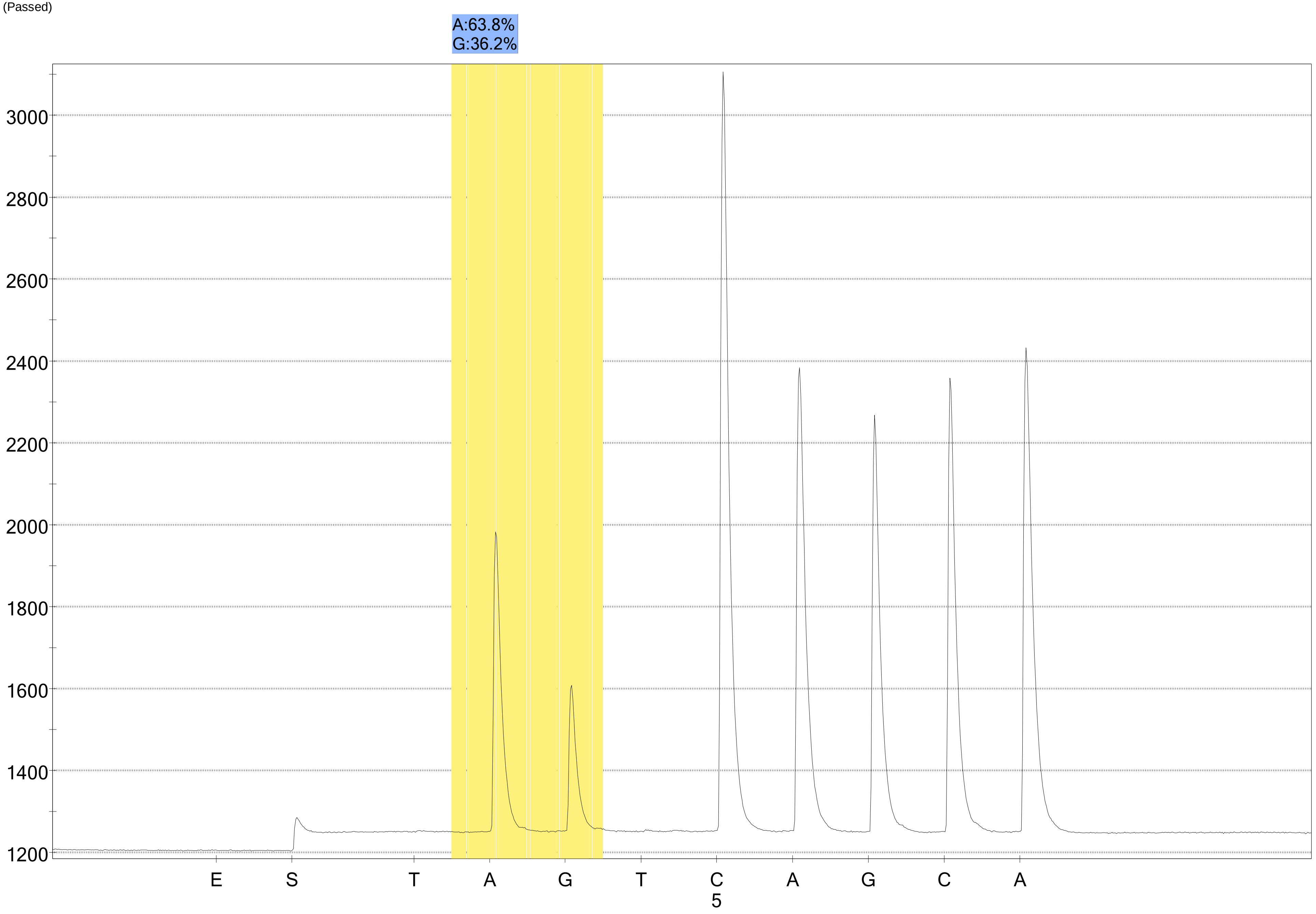

145 - Well E4
Entry: Limd1
1: A: 75.9% / G: 24.1%
(Passed)

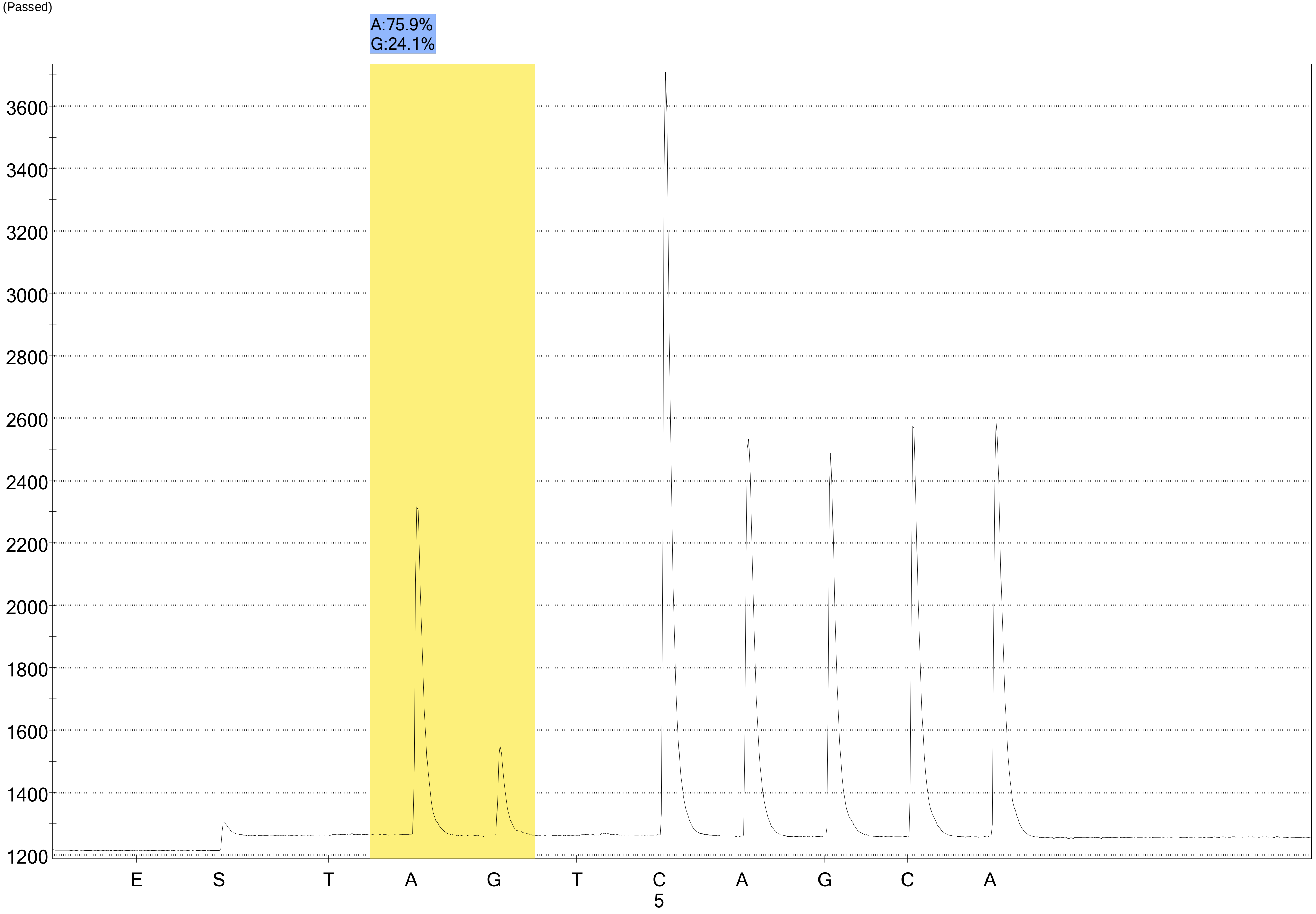

dna - Well E4
Entry: Limd1
1: A: 75.2% / G: 24.8%
(Passed)

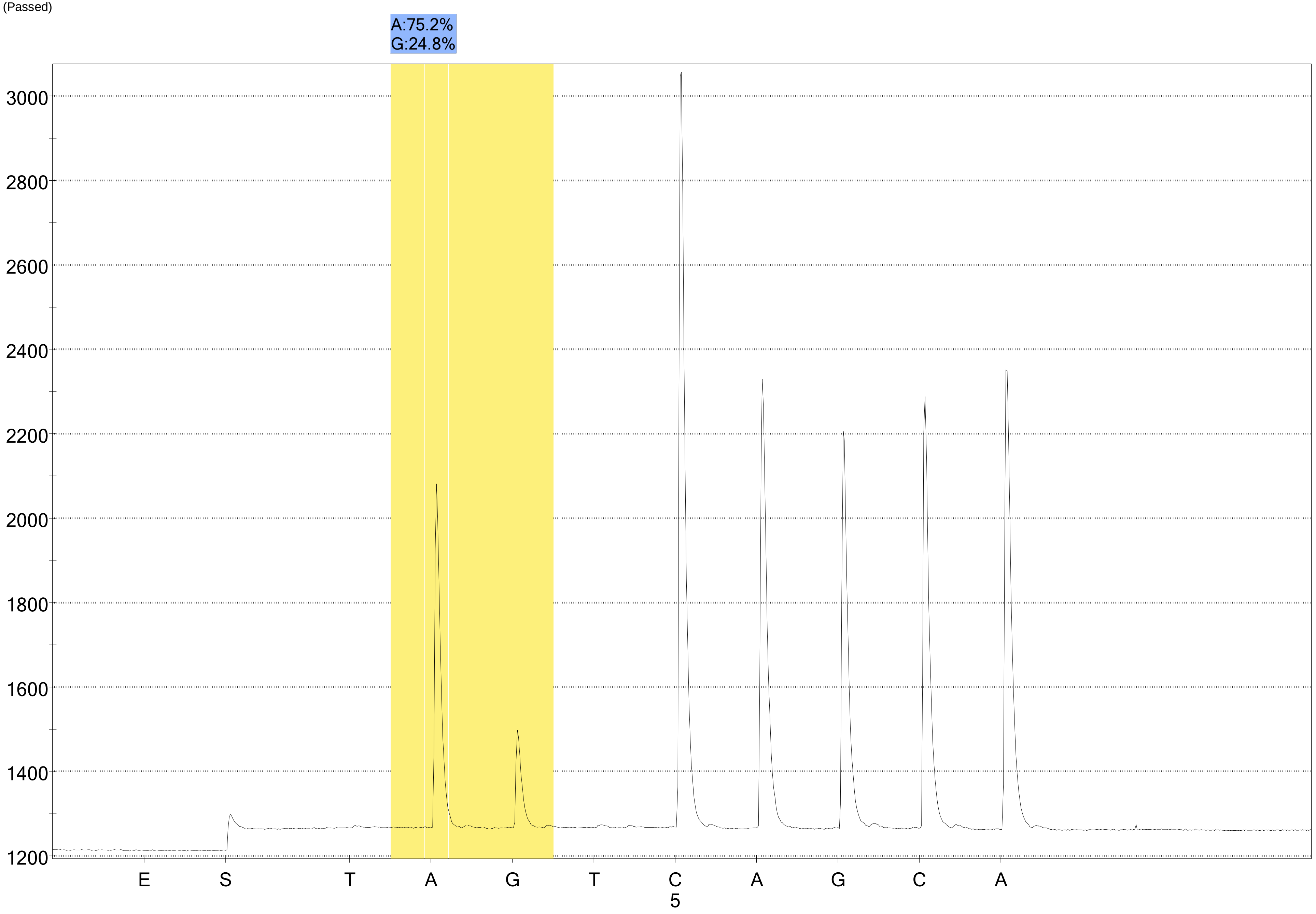

dna - Well E10
Entry: Limd1
1: A: 72.1% / G: 27.9%
(Passed)

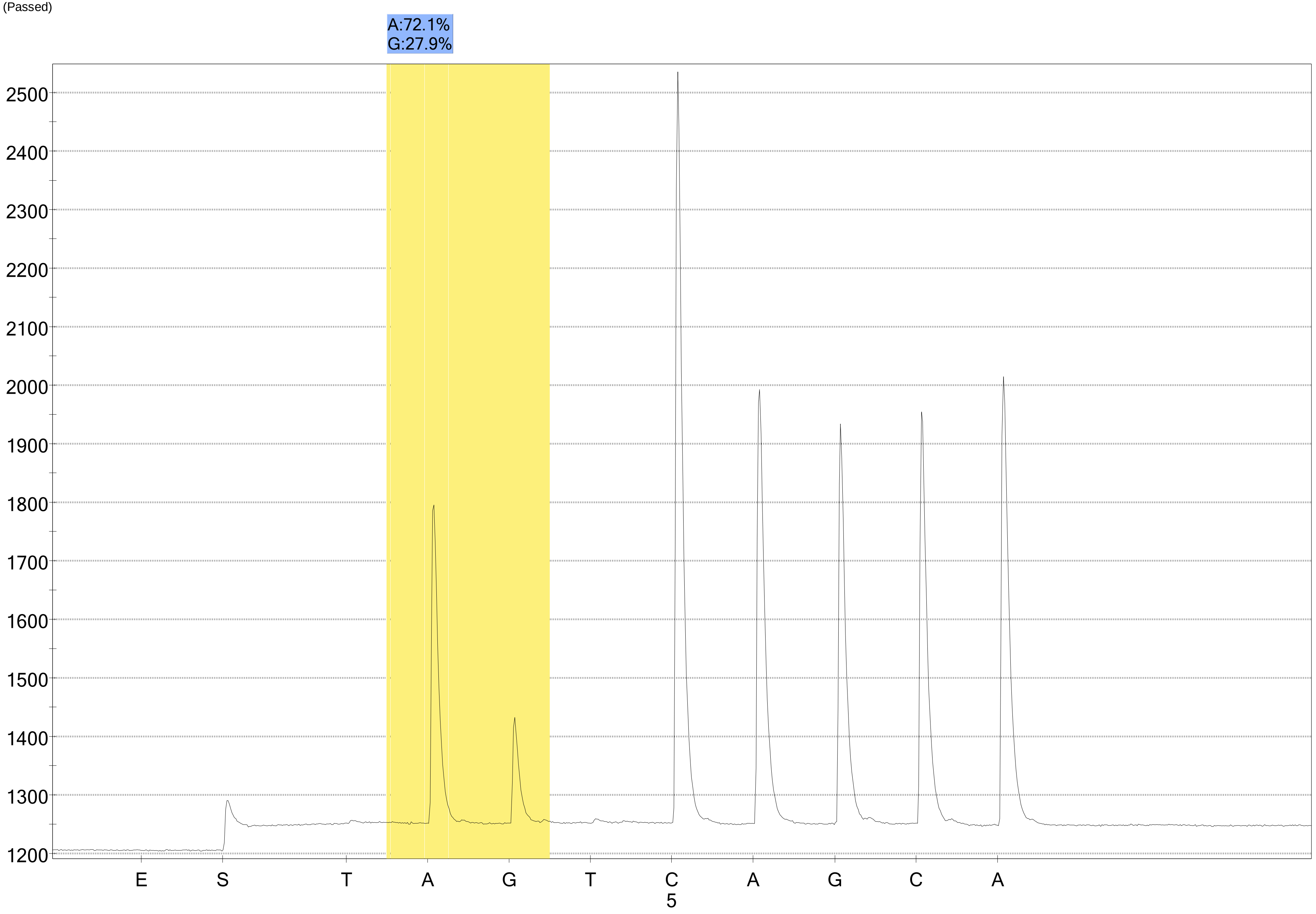

10 uL universal (141+157) - Well C6
Entry: Syt16
1: A: 45.3% / G: 54.7%
(Passed)

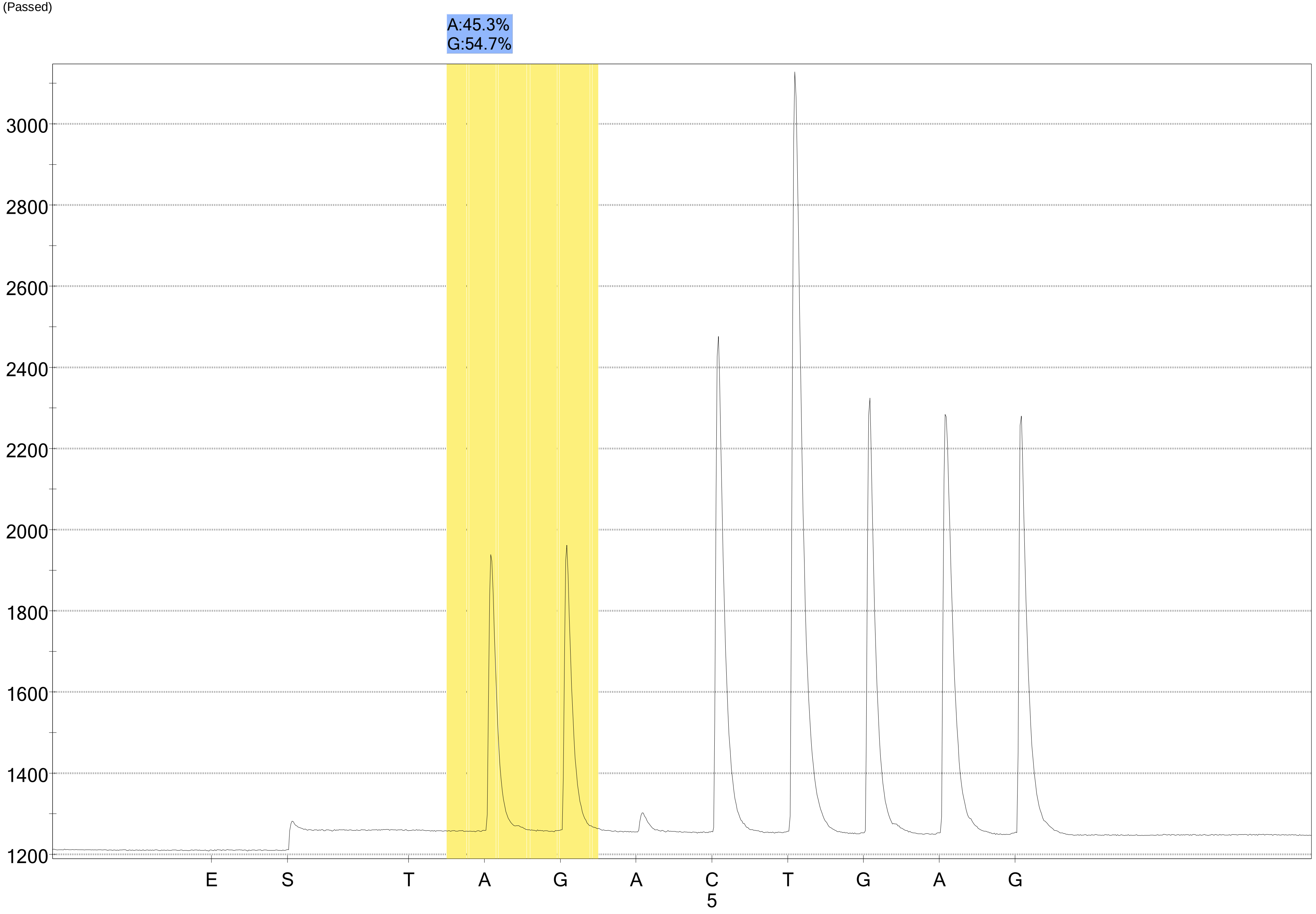

10 uL universal (141+157) - Well C12
Entry: Syt16
1: A: 43.5% / G: 56.5%
(Passed)

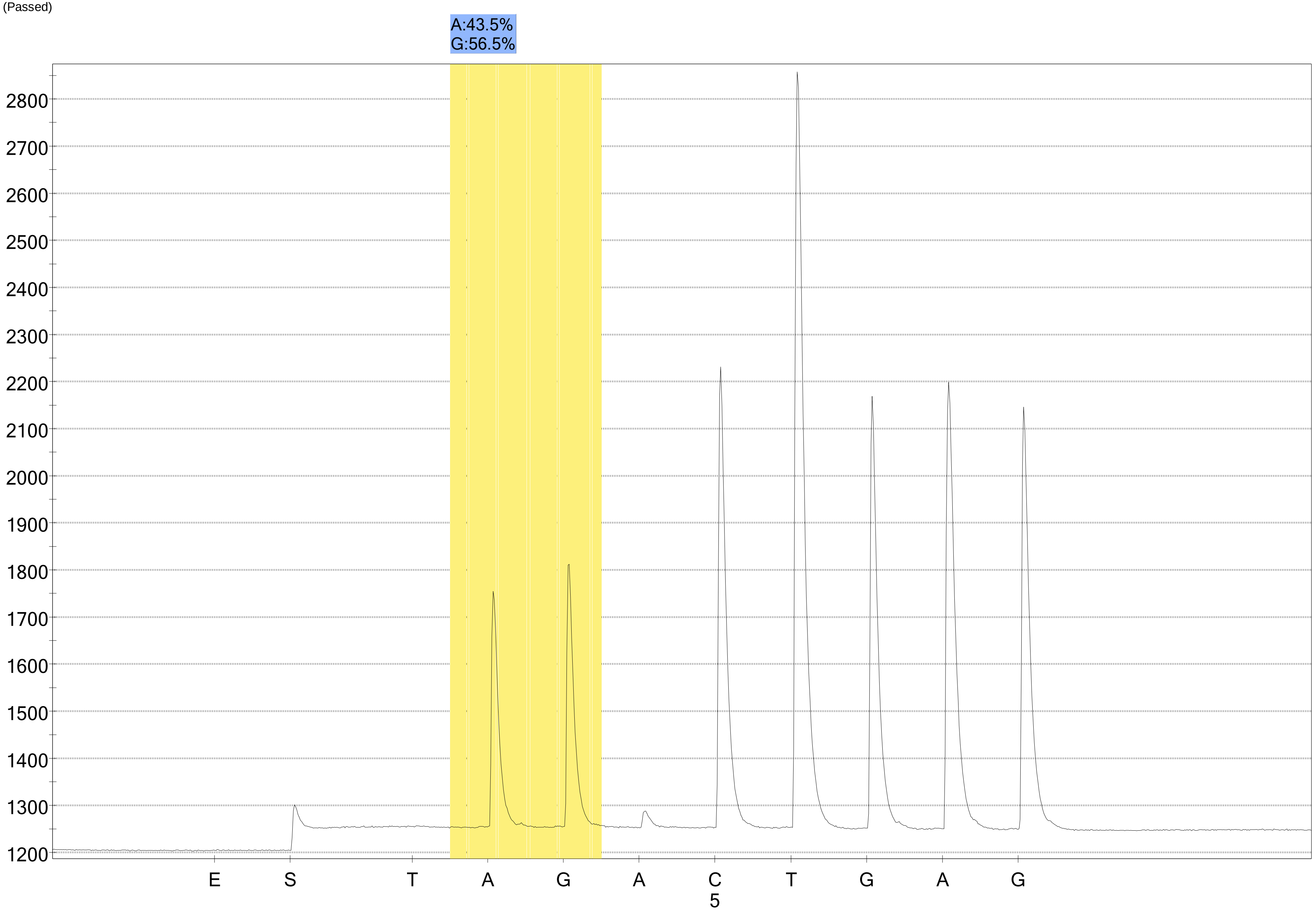

145 - Well C6
Entry: Syt16
1: A: 47.7% / G: 52.3%
(Passed)

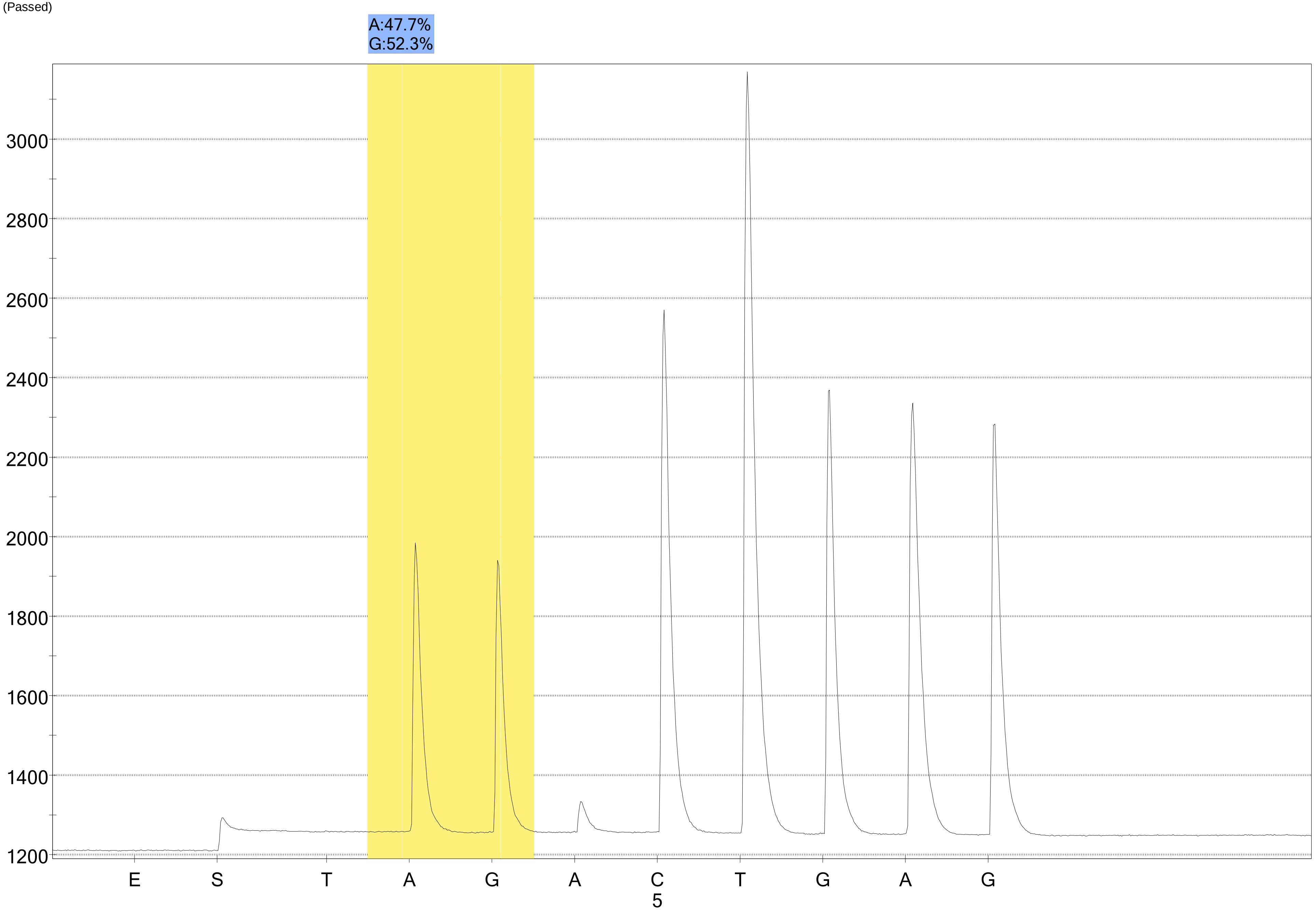

dna - Well C6
Entry: Syt16
1: A: 45.2% / G: 54.8%
(Passed)

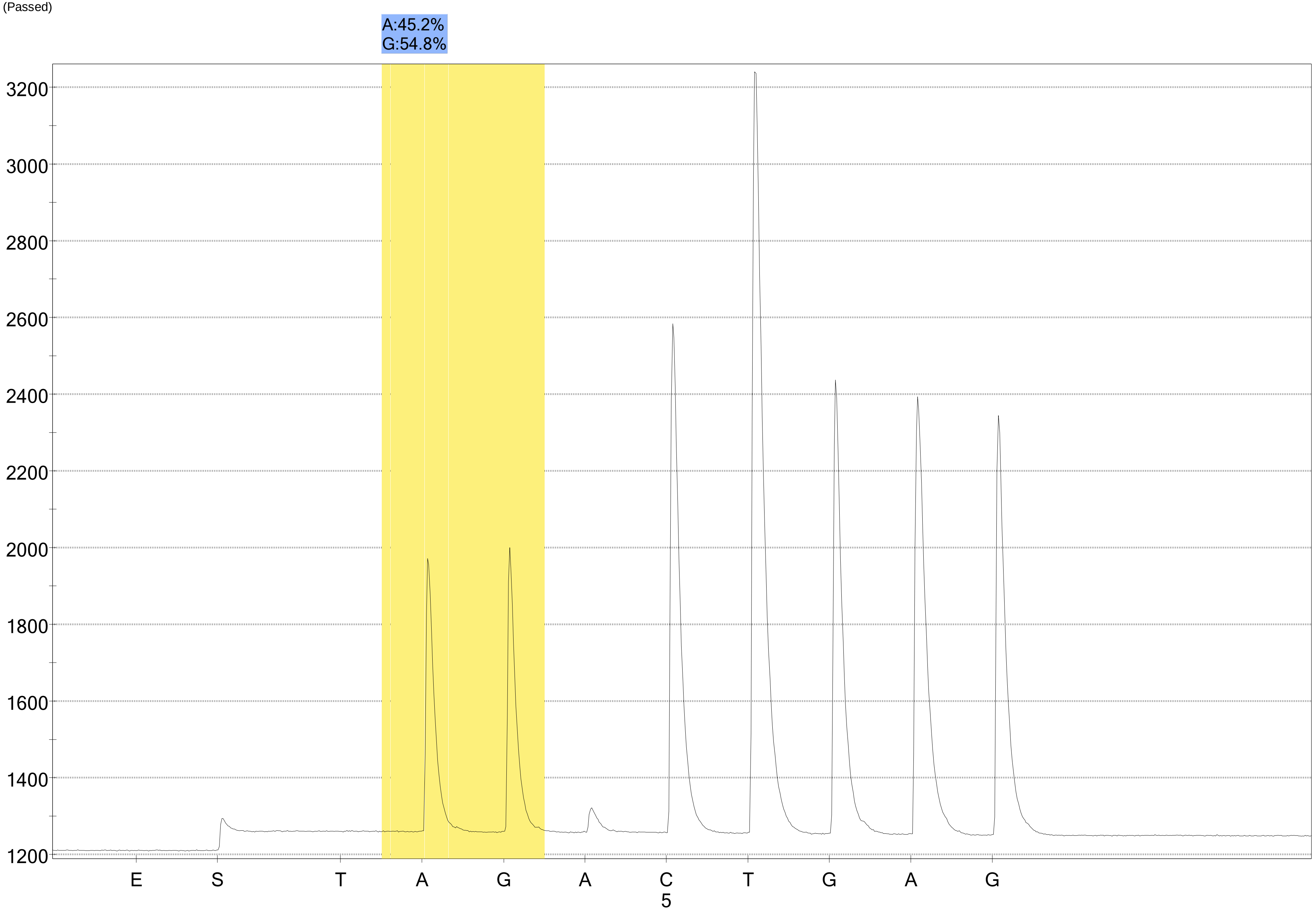

dna - Well C12
Entry: Syt16
1: A: 42.4% / G: 57.6%
(Passed)

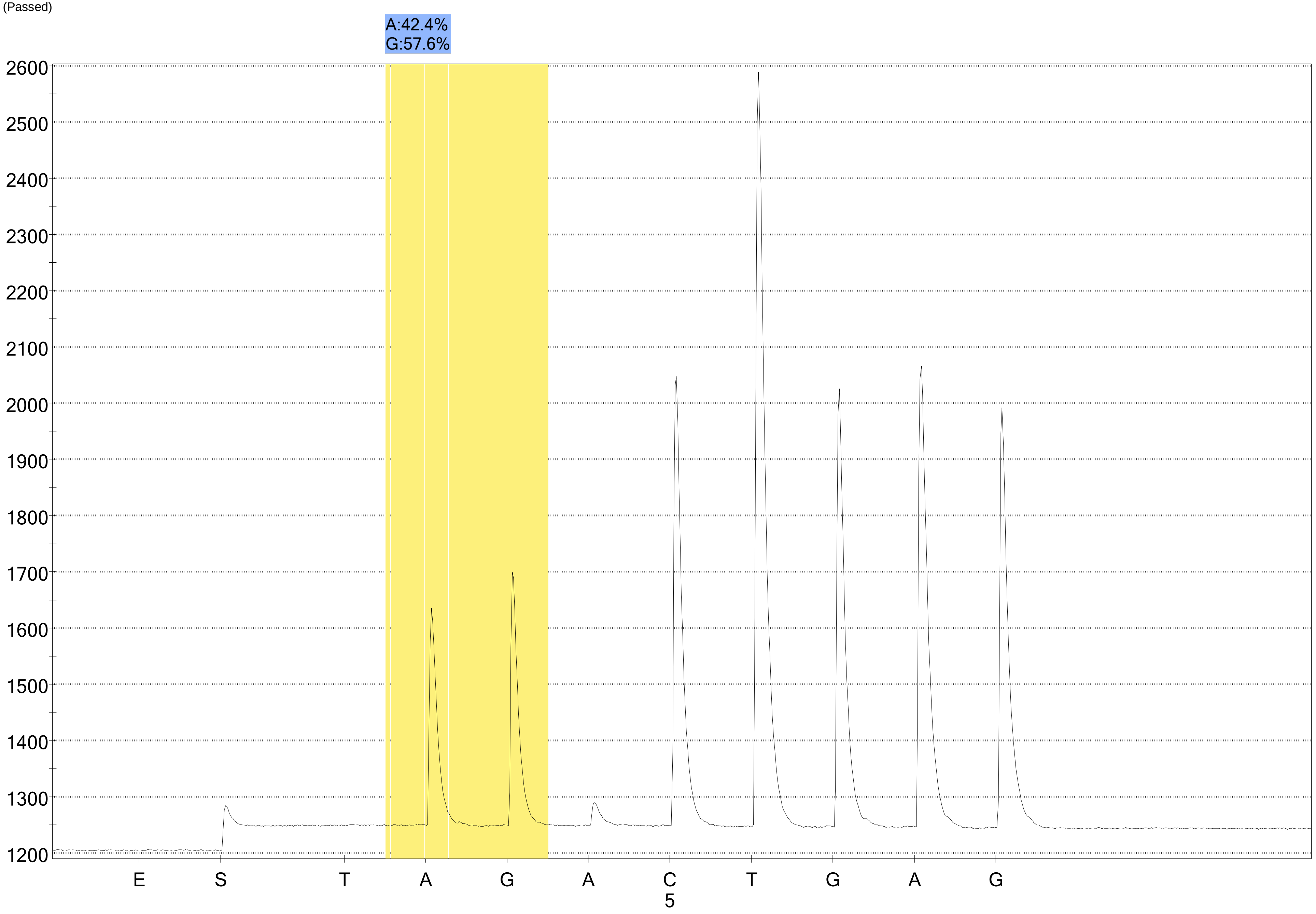

10 uL universal (141+157) - Well D6
Entry: Trak2
2: C: 42.4% / T: 57.6%
(Passed)

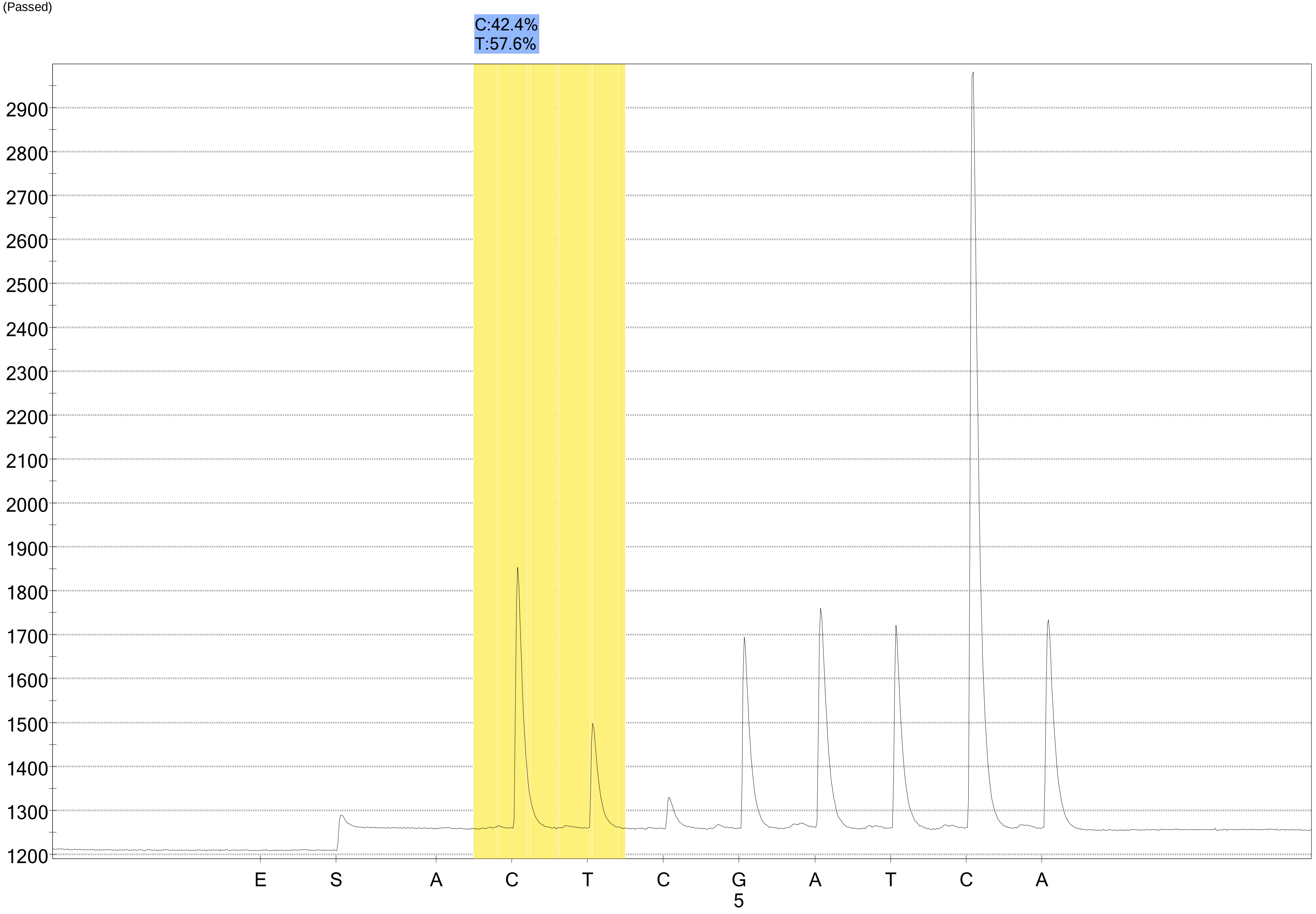

10 uL universal (141+157) - Well D12
Entry: Trak2
2: C: 42.6% / T: 57.4%
(Passed)

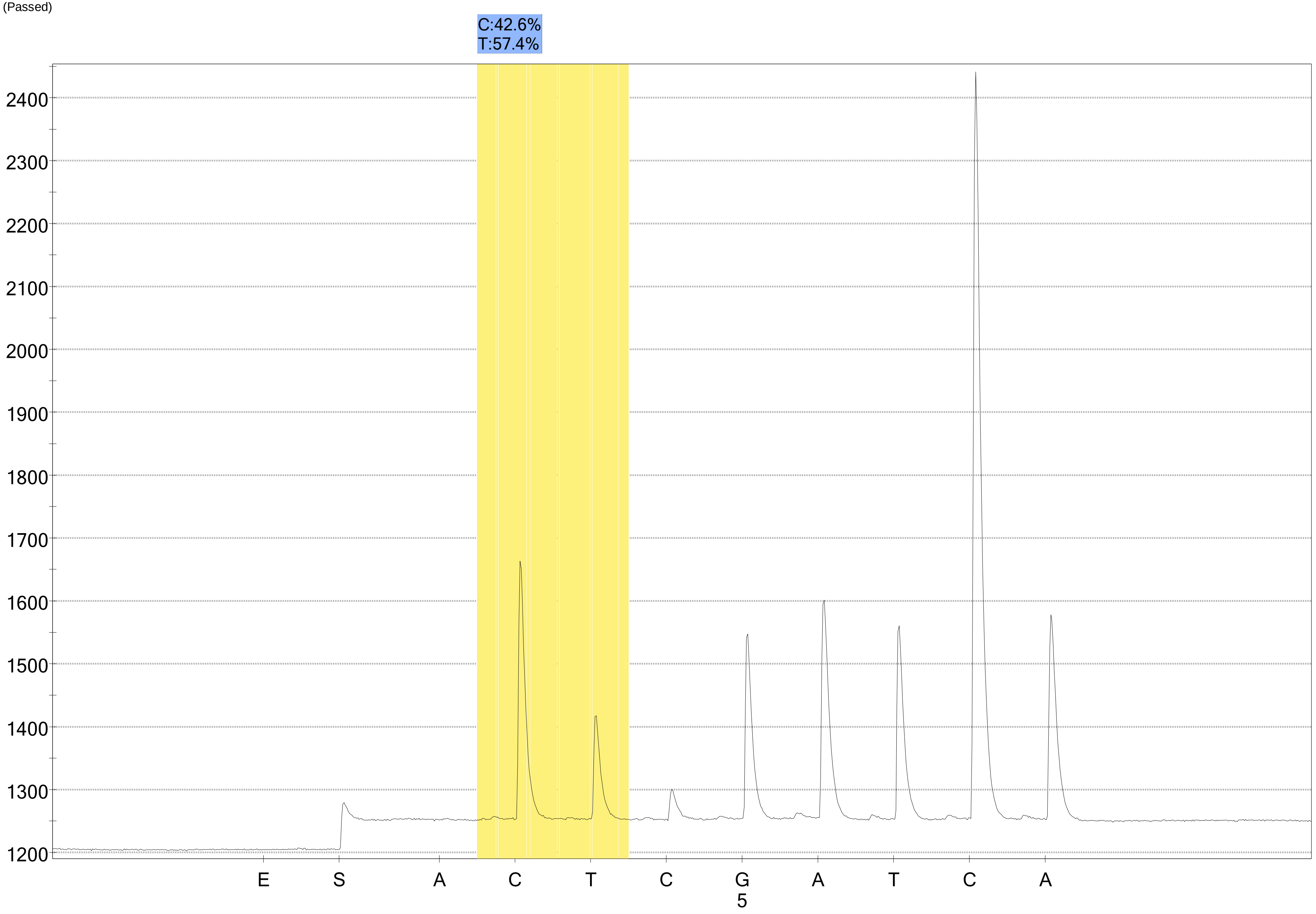

145 - Well D6
Entry: Trak2
2: C: 41.6% / T: 58.4%
(Passed)

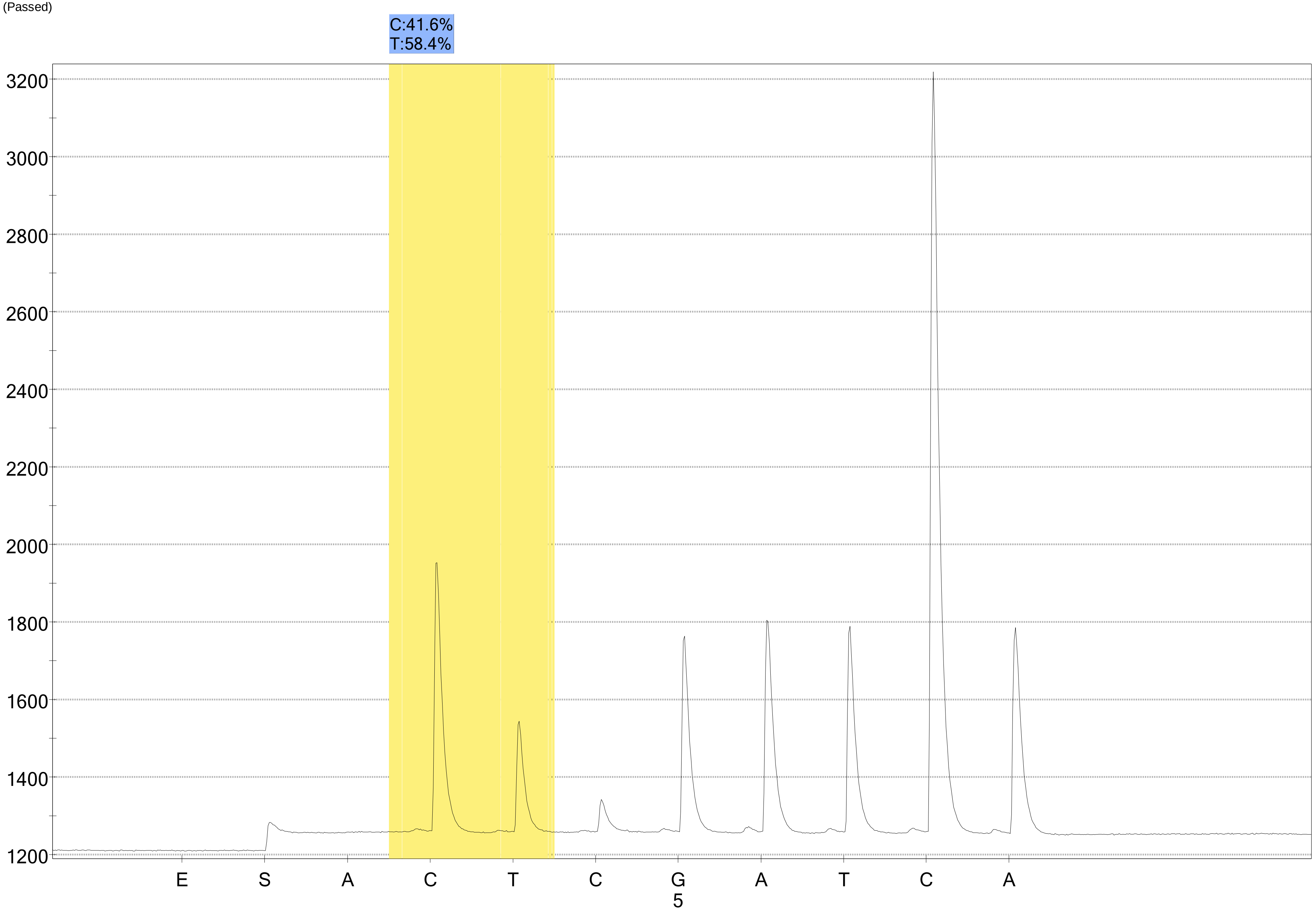

dna - Well D6
Entry: Trak2
2: C: 33.5% / T: 66.5%
(Passed)

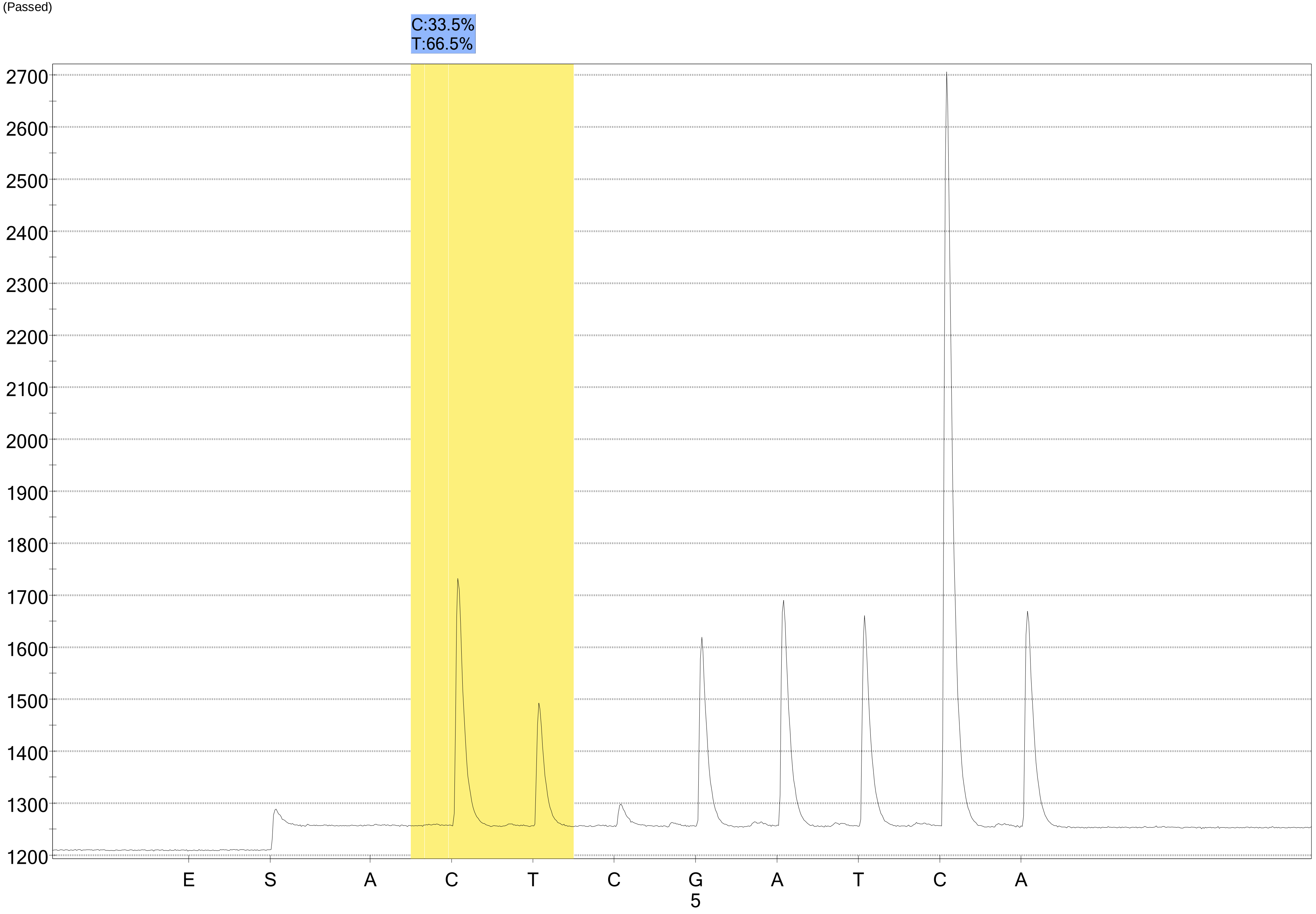

dna - Well D12
Entry: Trak2
2: C: 34.1% / T: 65.9%
(Passed)

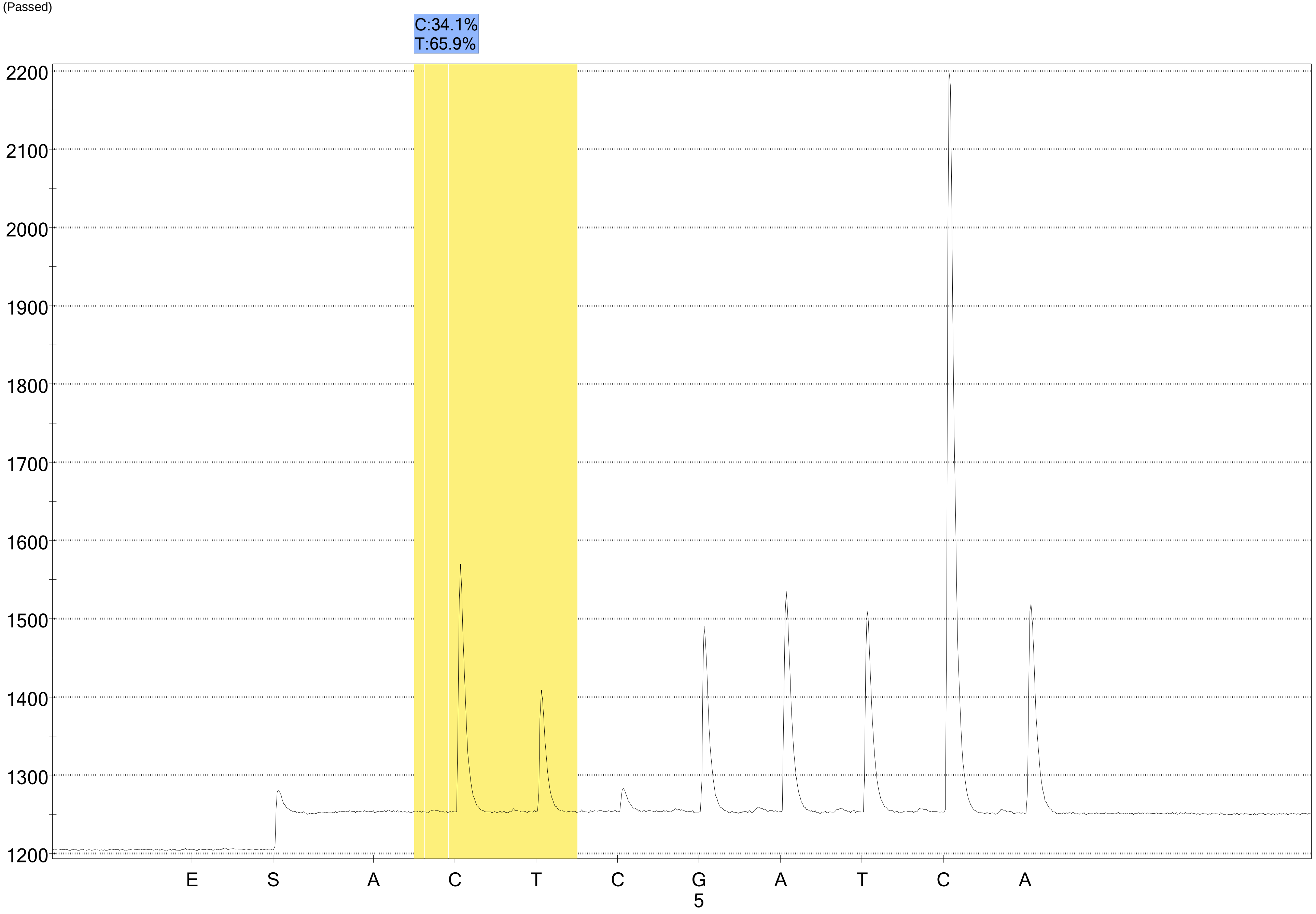

10 uL universal (141+157) - Well B2
Entry: Bmp1
1: C: 72.3% / T: 27.7%
(Passed)

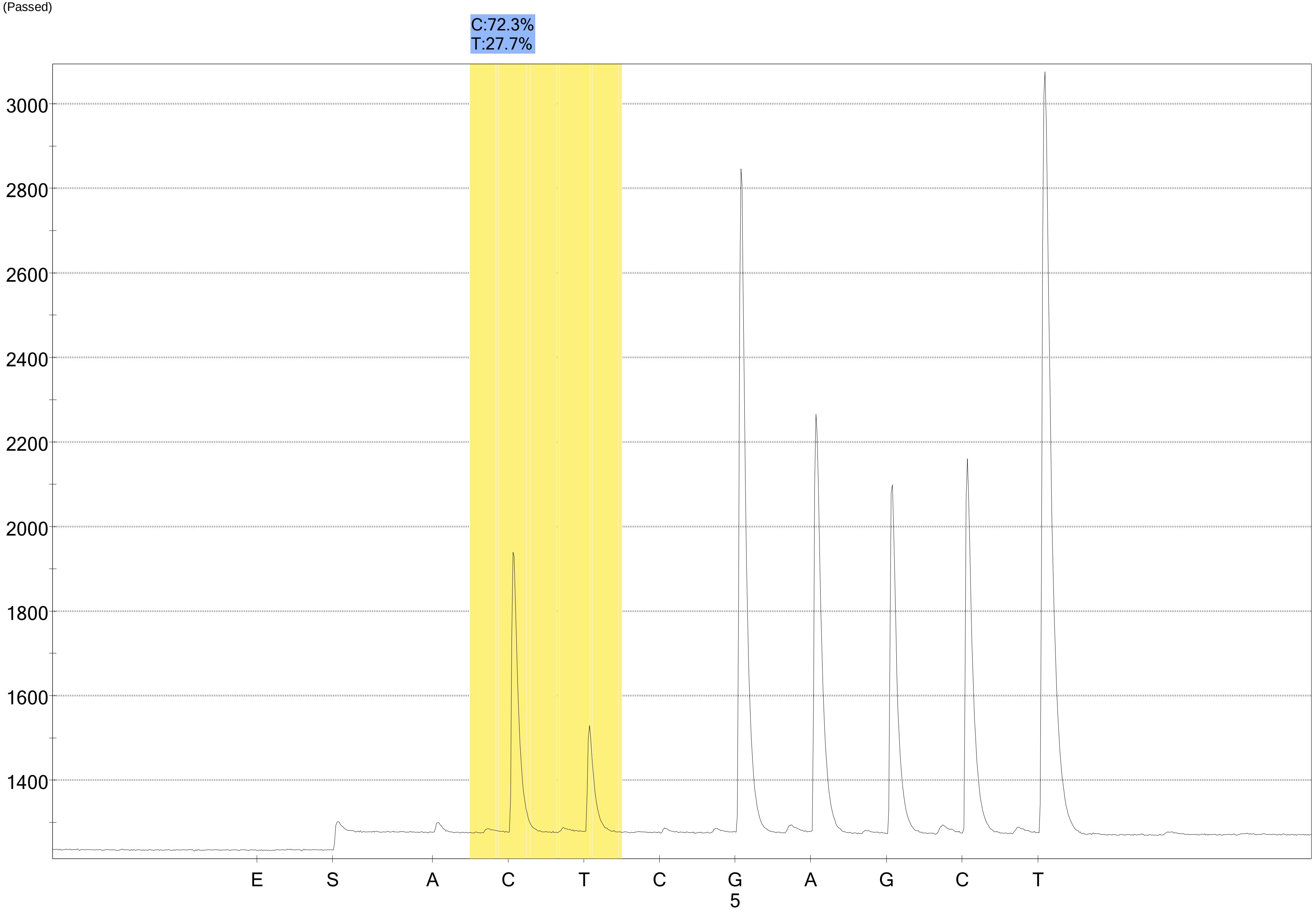

10 uL universal (141+157) - Well B8
Entry: Bmp1
1: C: 76.5% / T: 23.5%
(Passed)

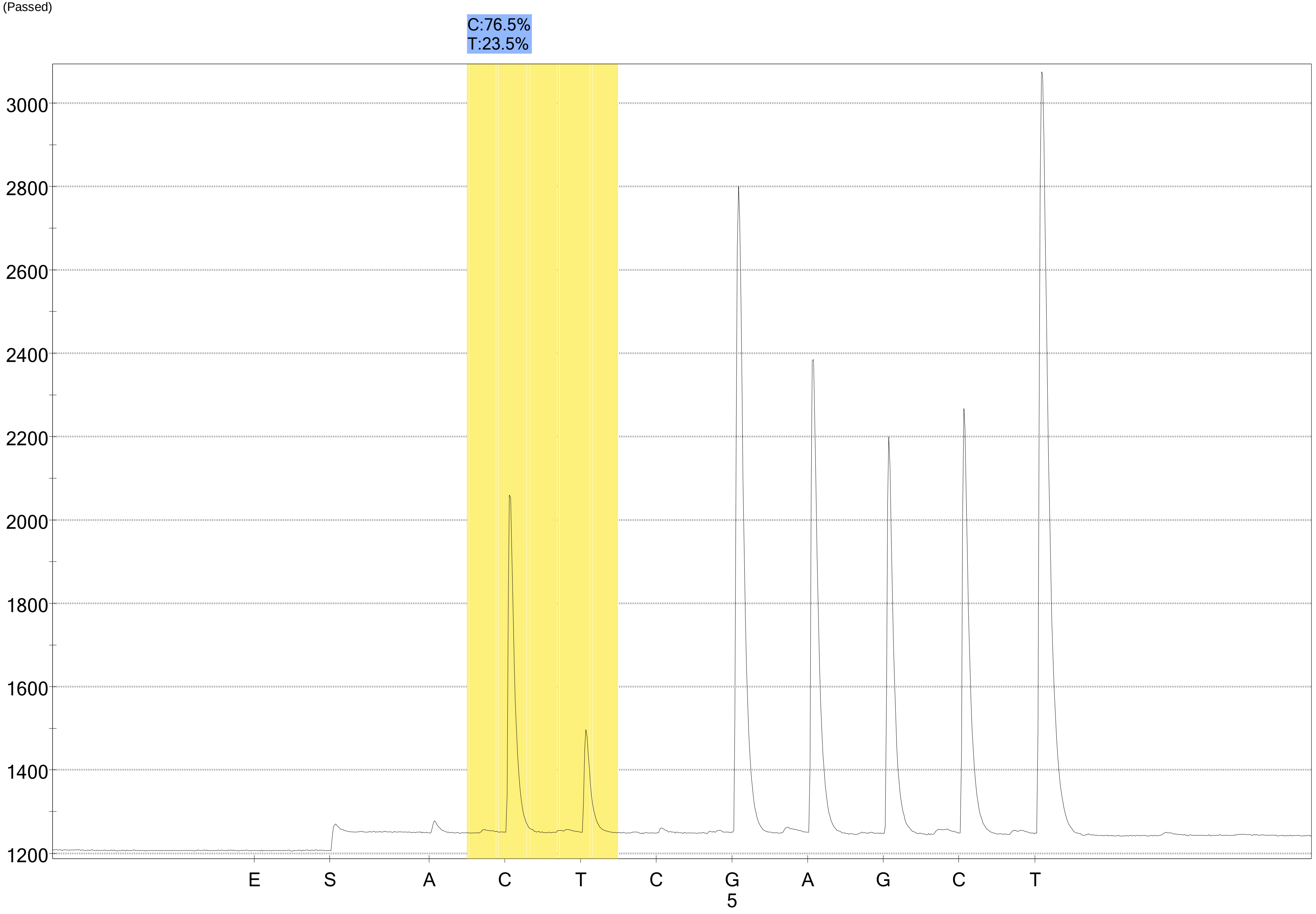

145 - Well B2
Entry: Bmp1
1: C: 73.8% / T: 26.2%
(Passed)

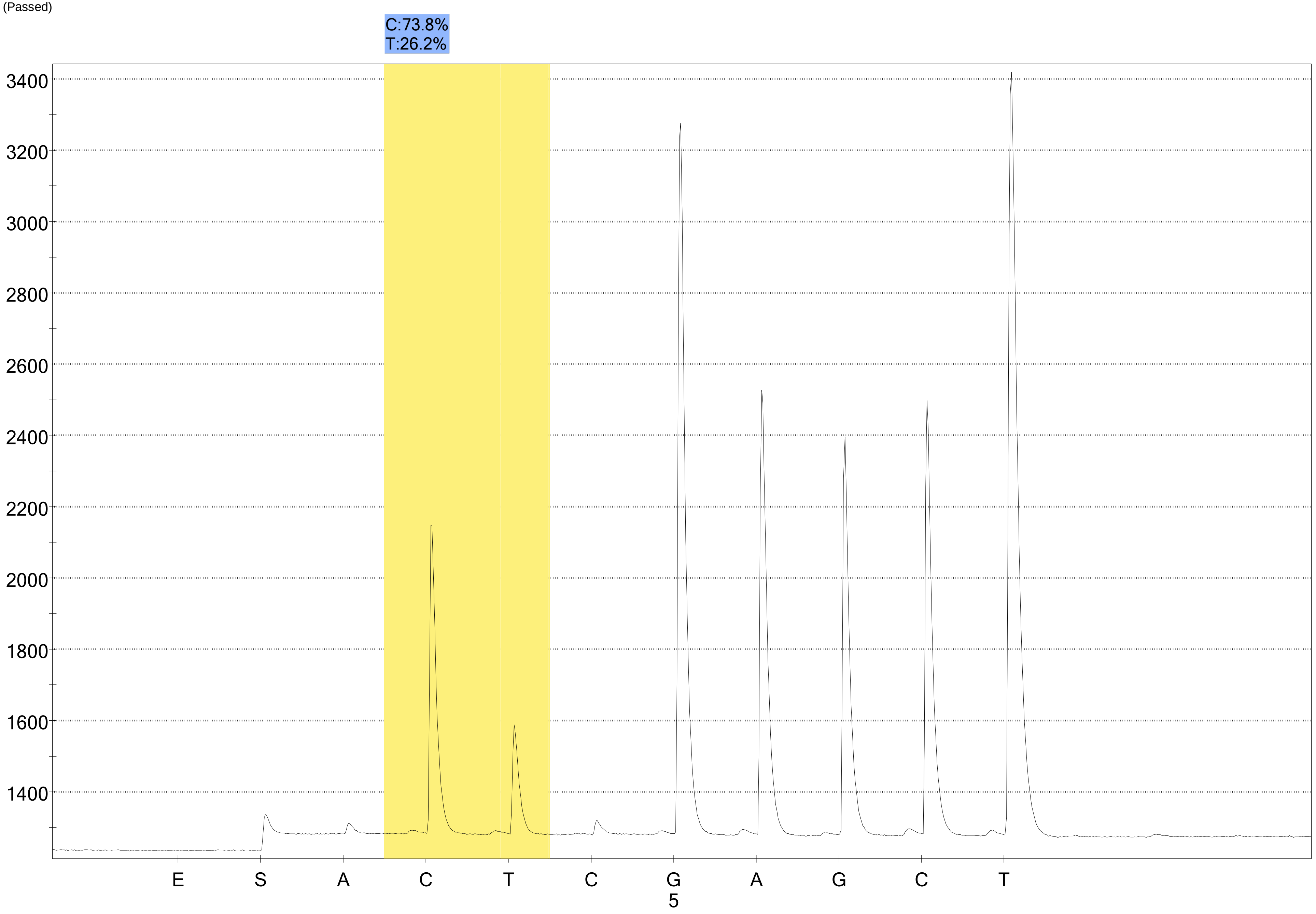

dna - Well B2
Entry: Bmp1
1: C: 79.6% / T: 20.4%
(Passed)

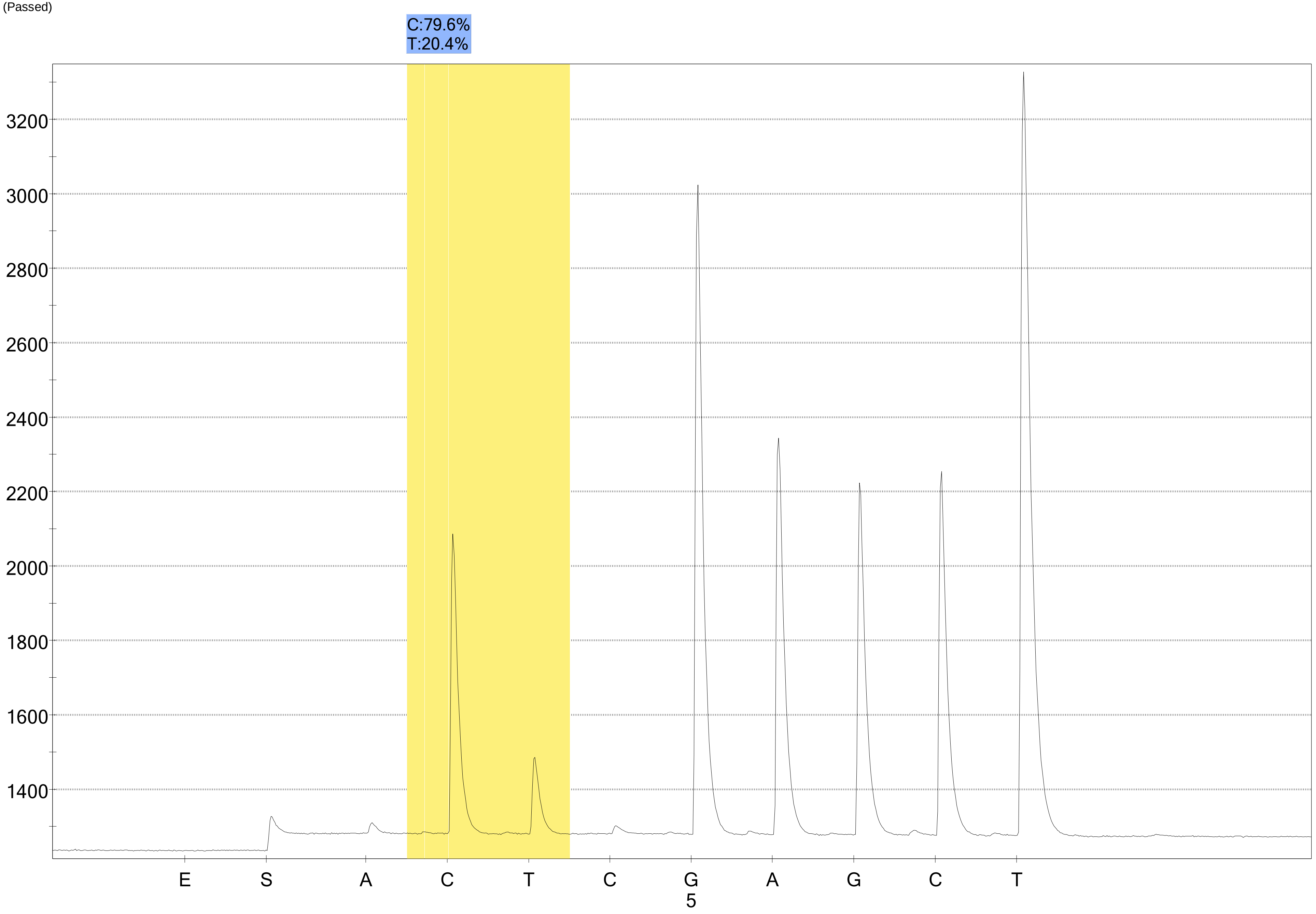

dna - Well B8
Entry: Bmp1
1: C: 82.2% / T: 17.8%
(Passed)

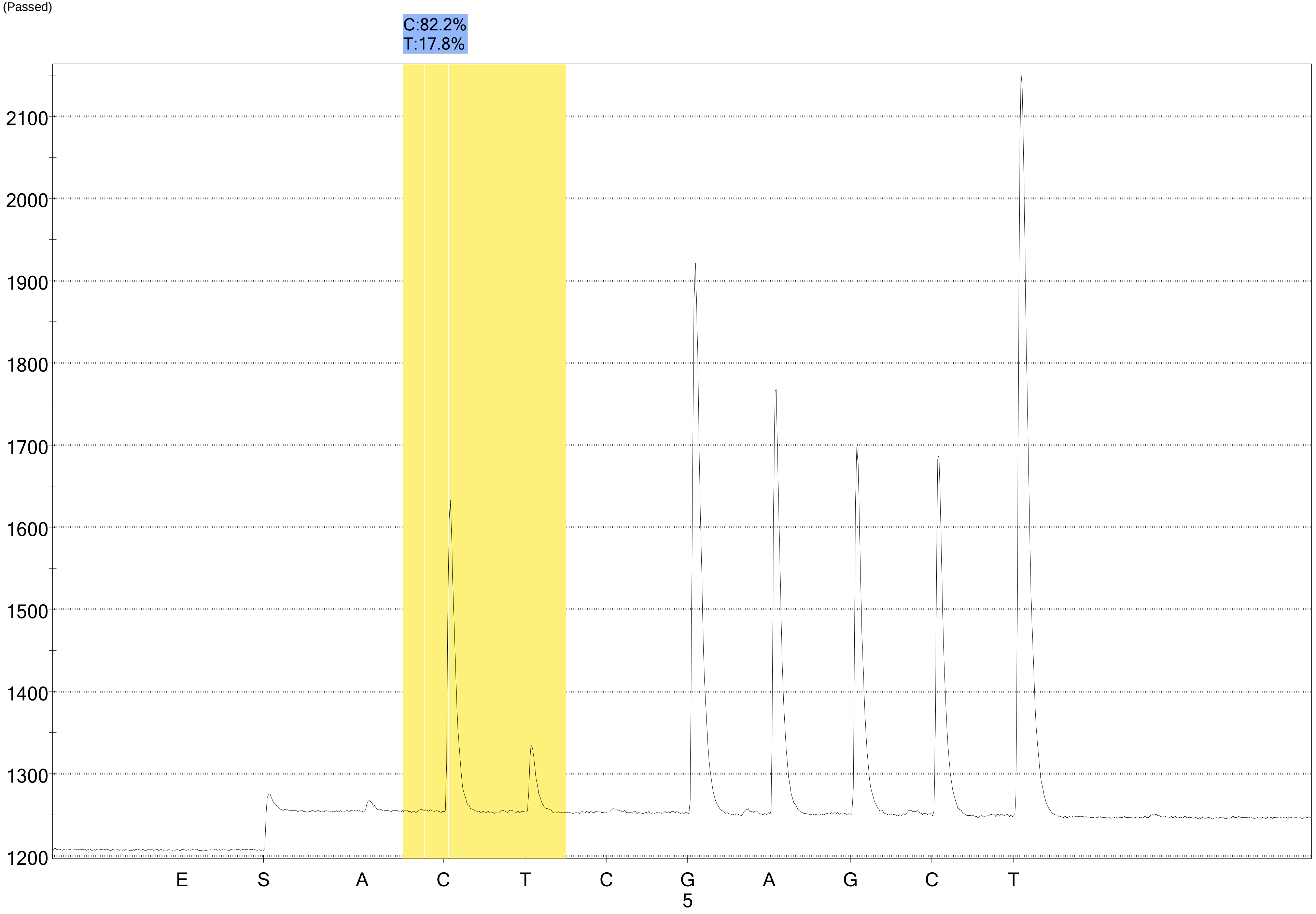

10 uL universal (141+157) - Well F5
Entry: Pld4
4: A: 51.4% / G: 48.6%
(Passed)

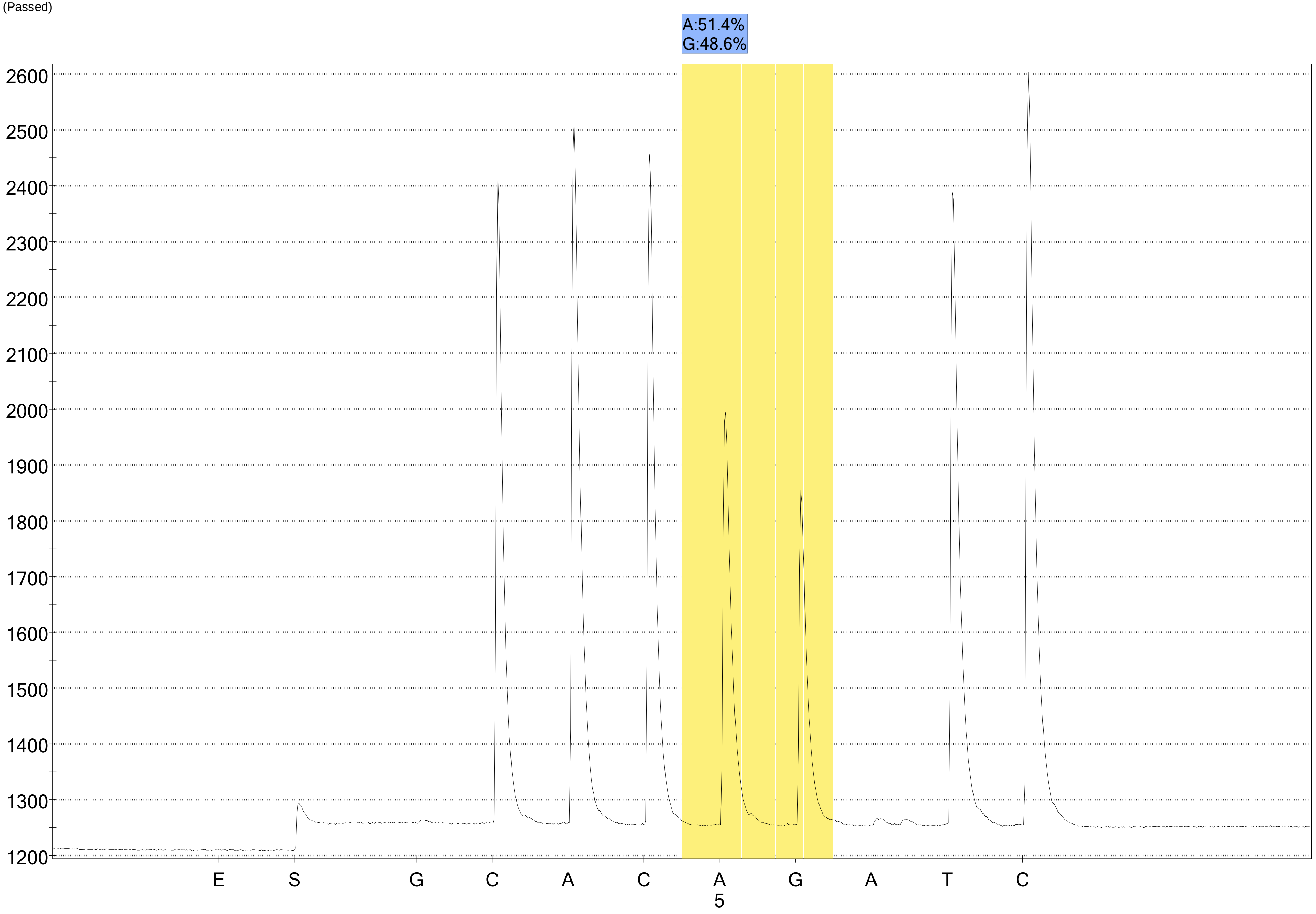

10 uL universal (141+157) - Well F11
Entry: Pld4
4: A: 51.5% / G: 48.5%
(Passed)

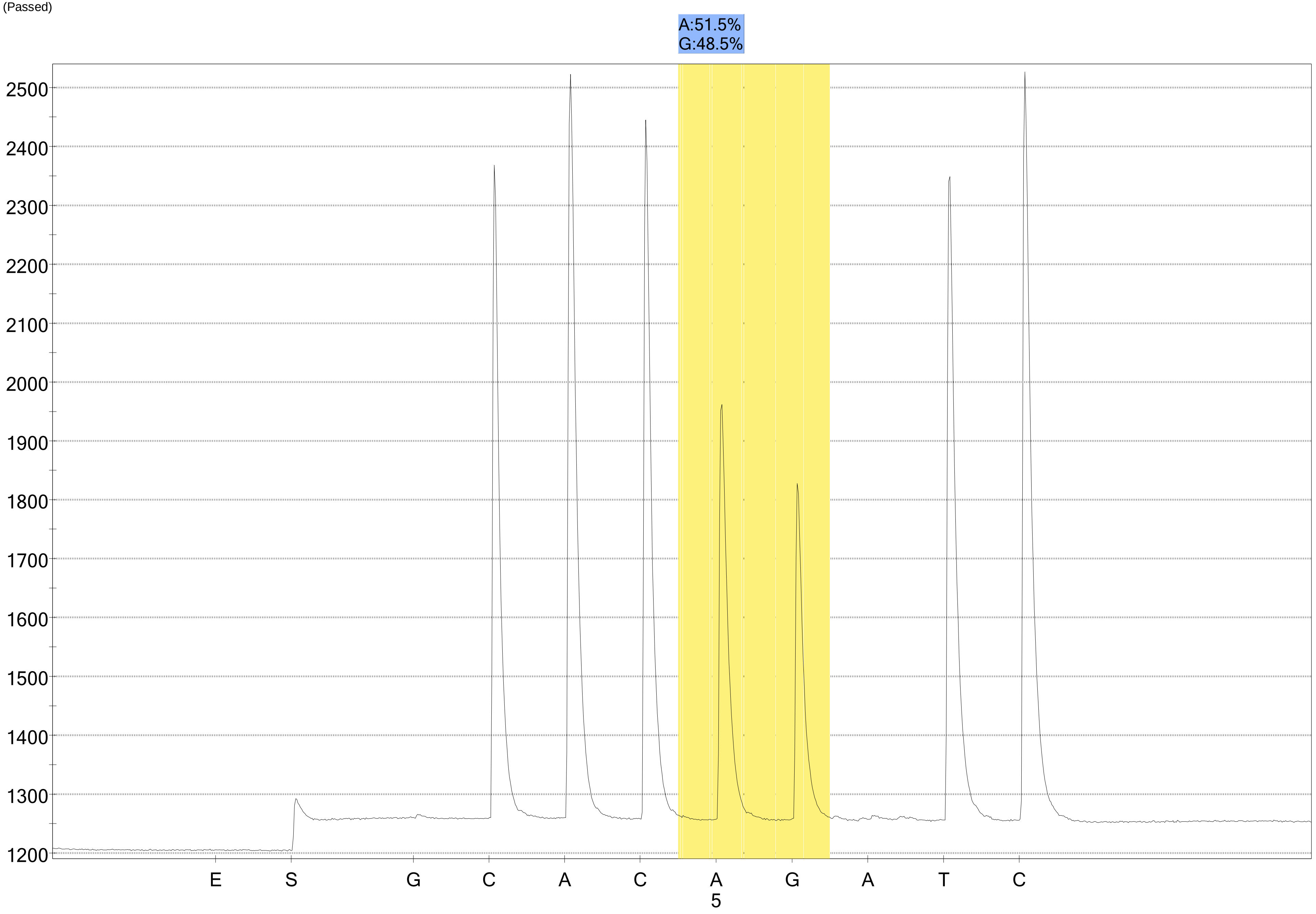

145 - Well F5
Entry: Pld4
4: A: 62.3% / G: 37.7%
(Passed)

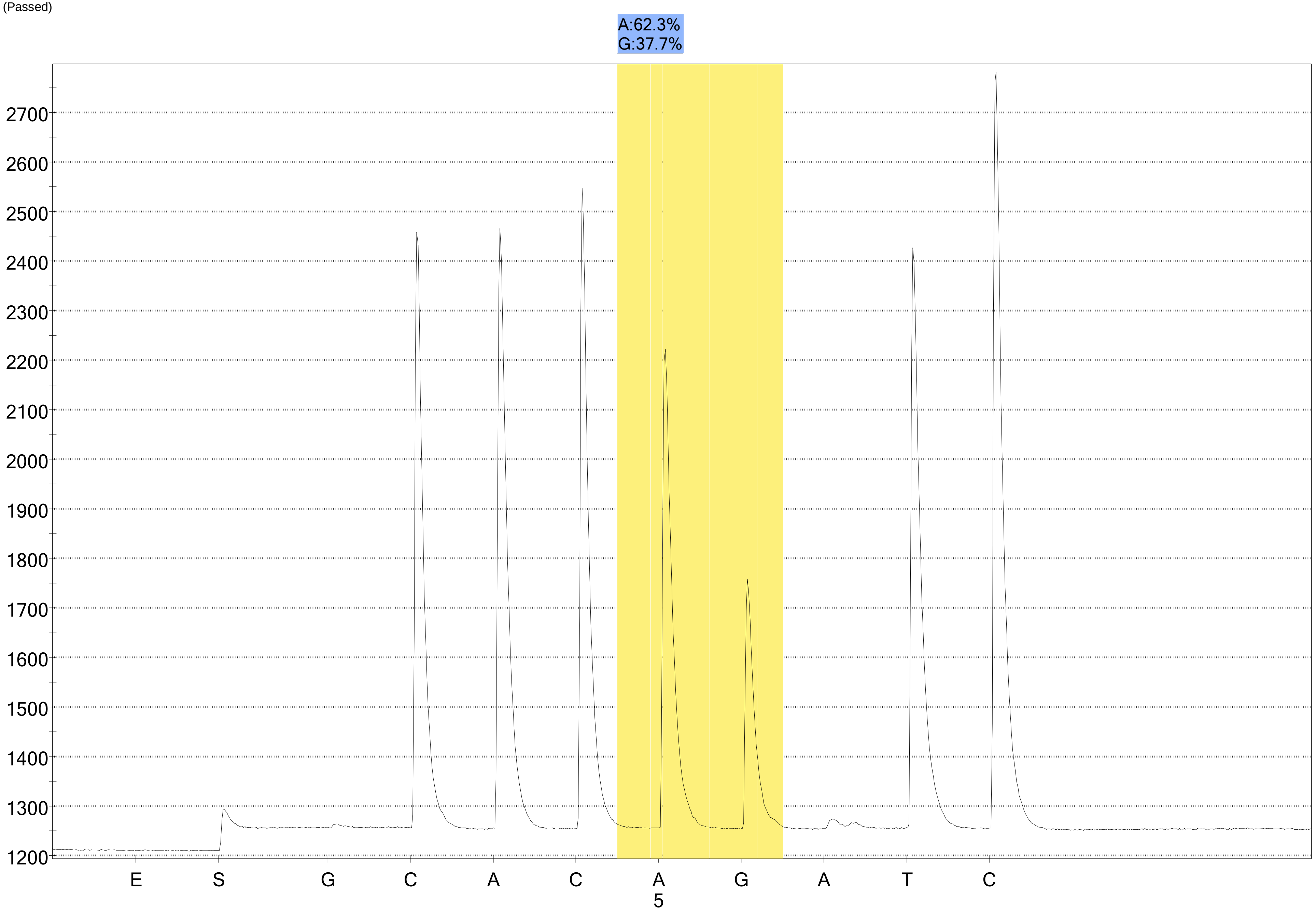

dna - Well F5
Entry: Pld4
4: A: 57.9% / G: 42.1%
(Passed)

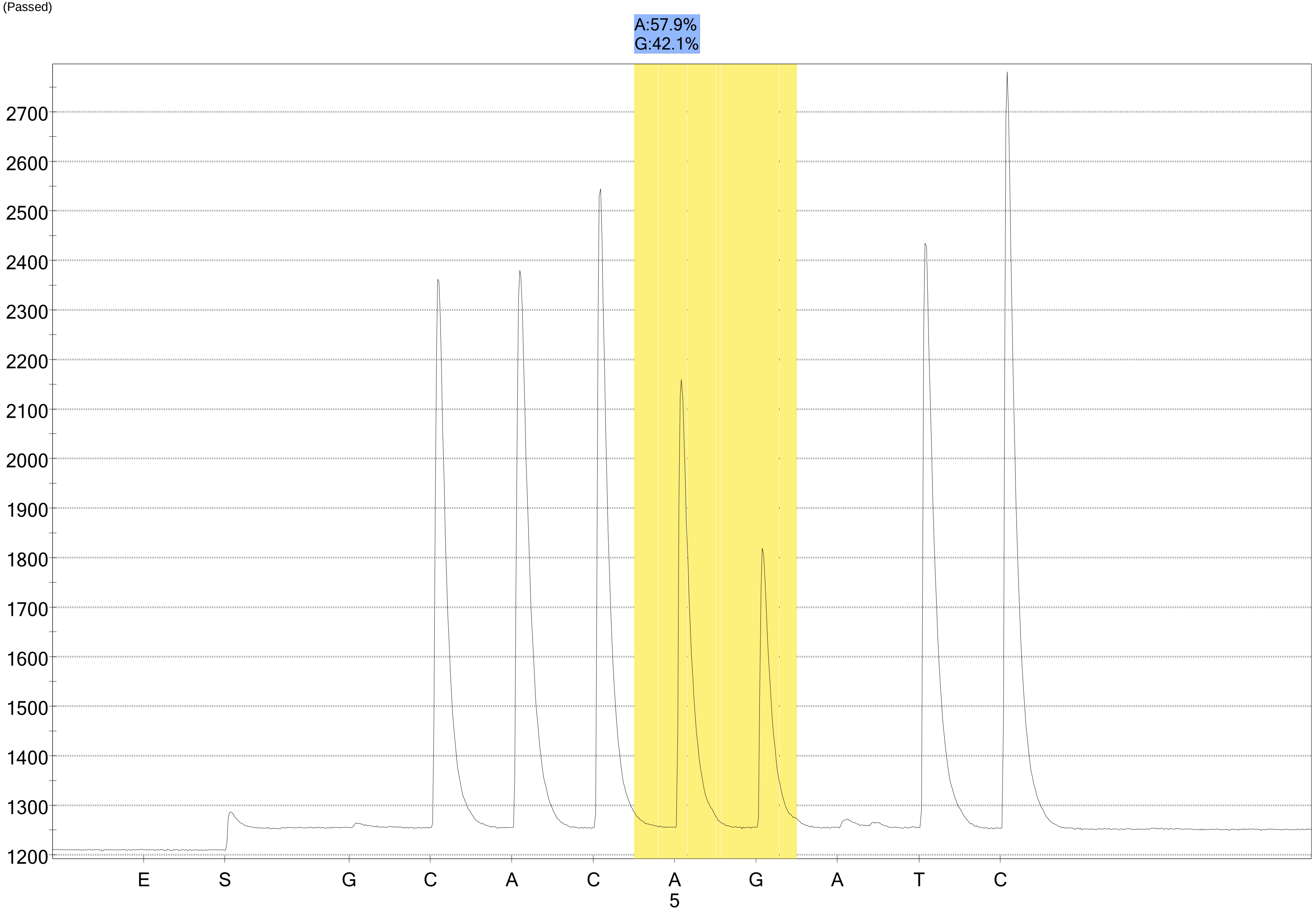

dna - Well F11
Entry: Pld4
4: A: 58.4% / G: 41.6%
(Passed)

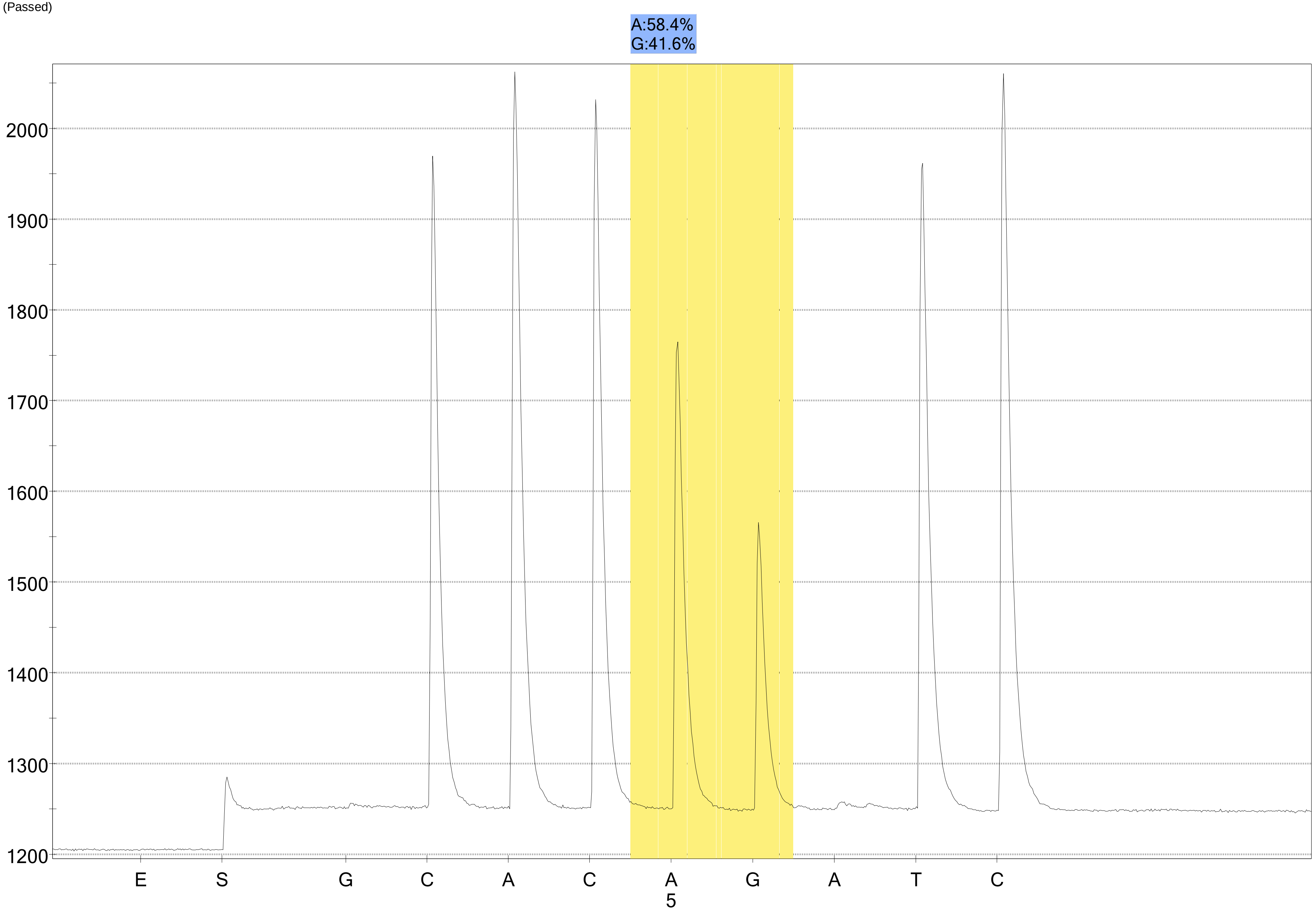

10 uL universal (141+157) - Well A3
Entry: Cplx2
2: C: 49.8% / T: 50.2%
(Passed)

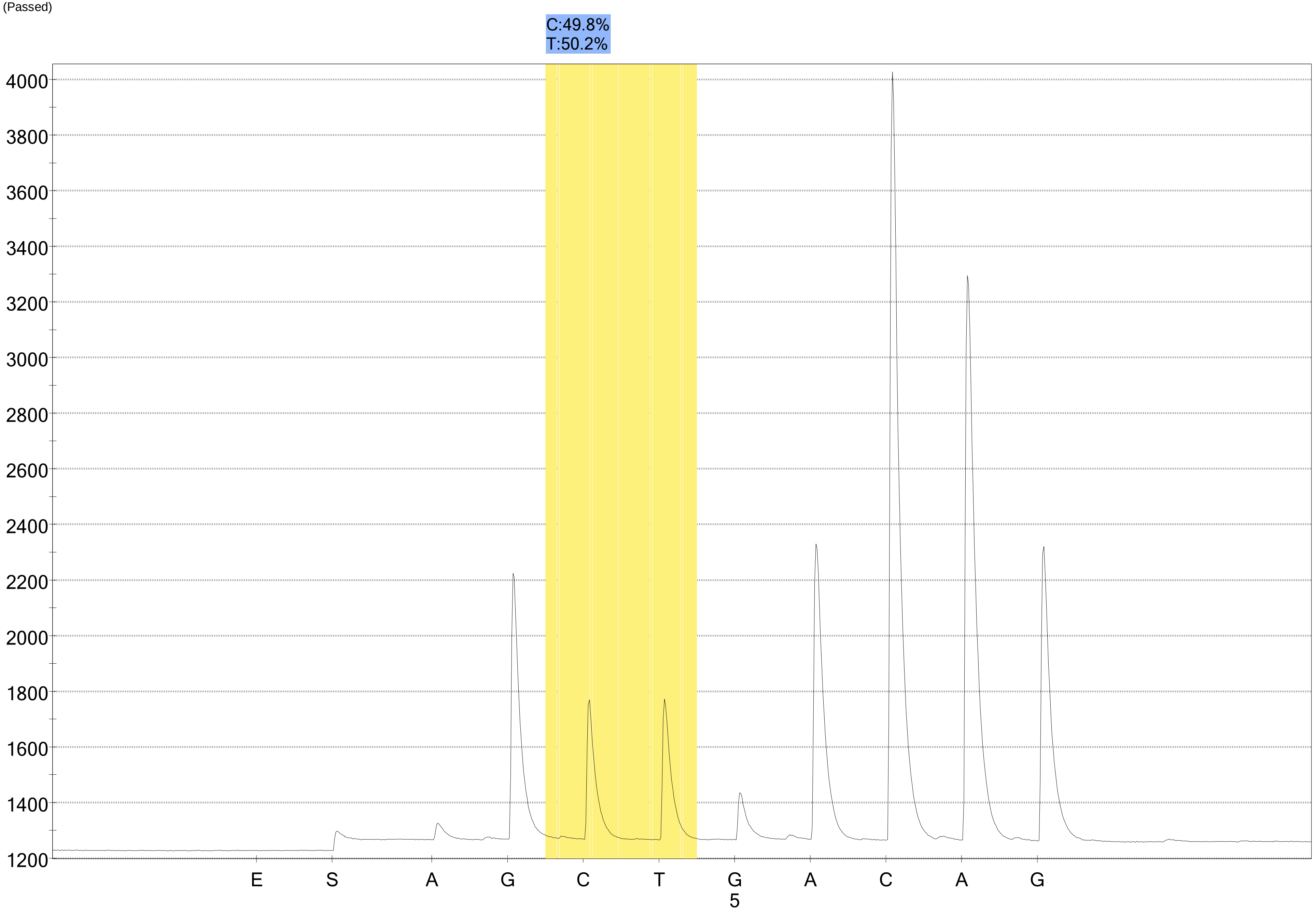

10 uL universal (141+157) - Well A9
Entry: Cplx2
2: C: 49.9% / T: 50.1%
(Passed)

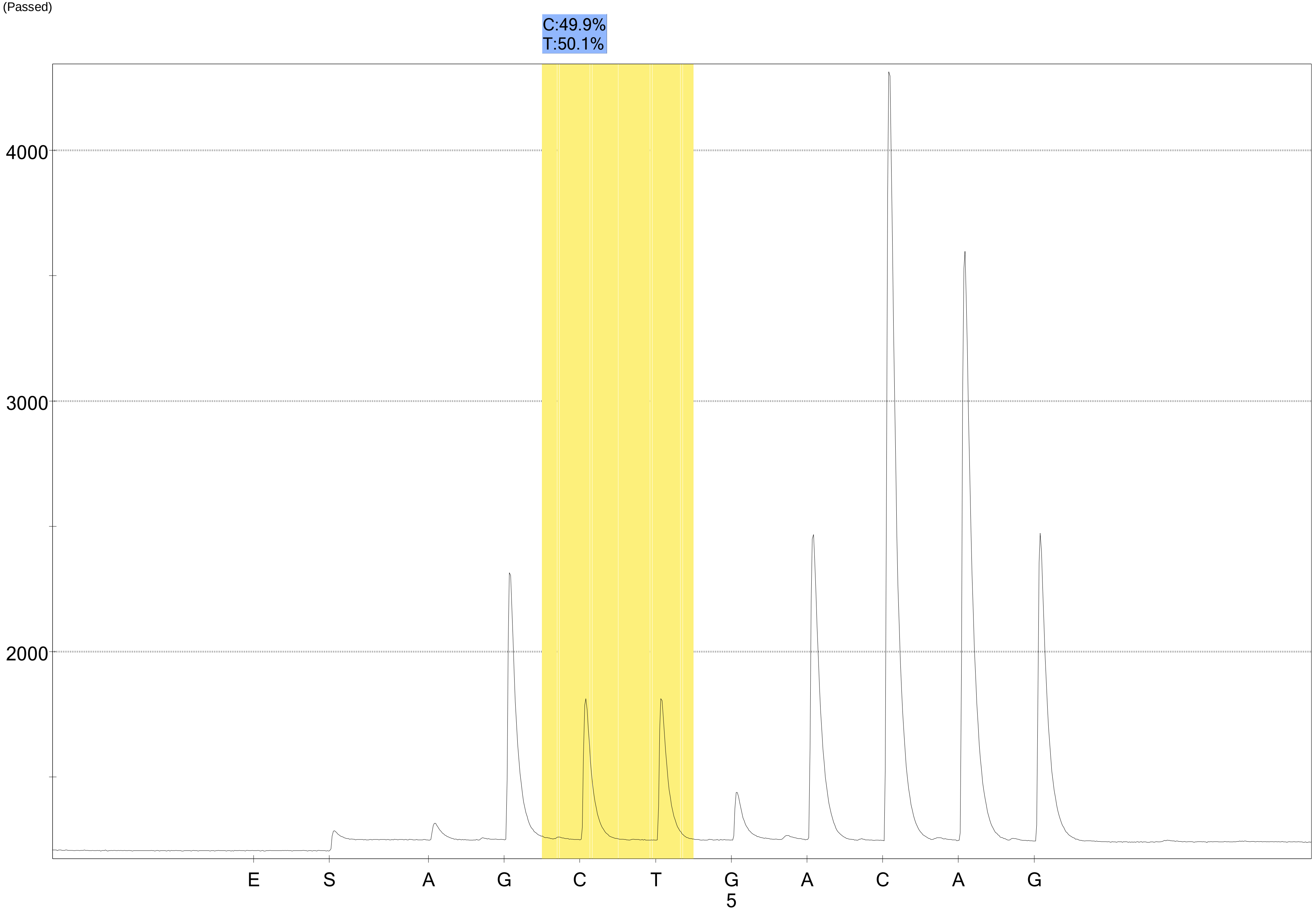

145 - Well A3
Entry: Cplx2
2: C: 50.5% / T: 49.5%
(Passed)

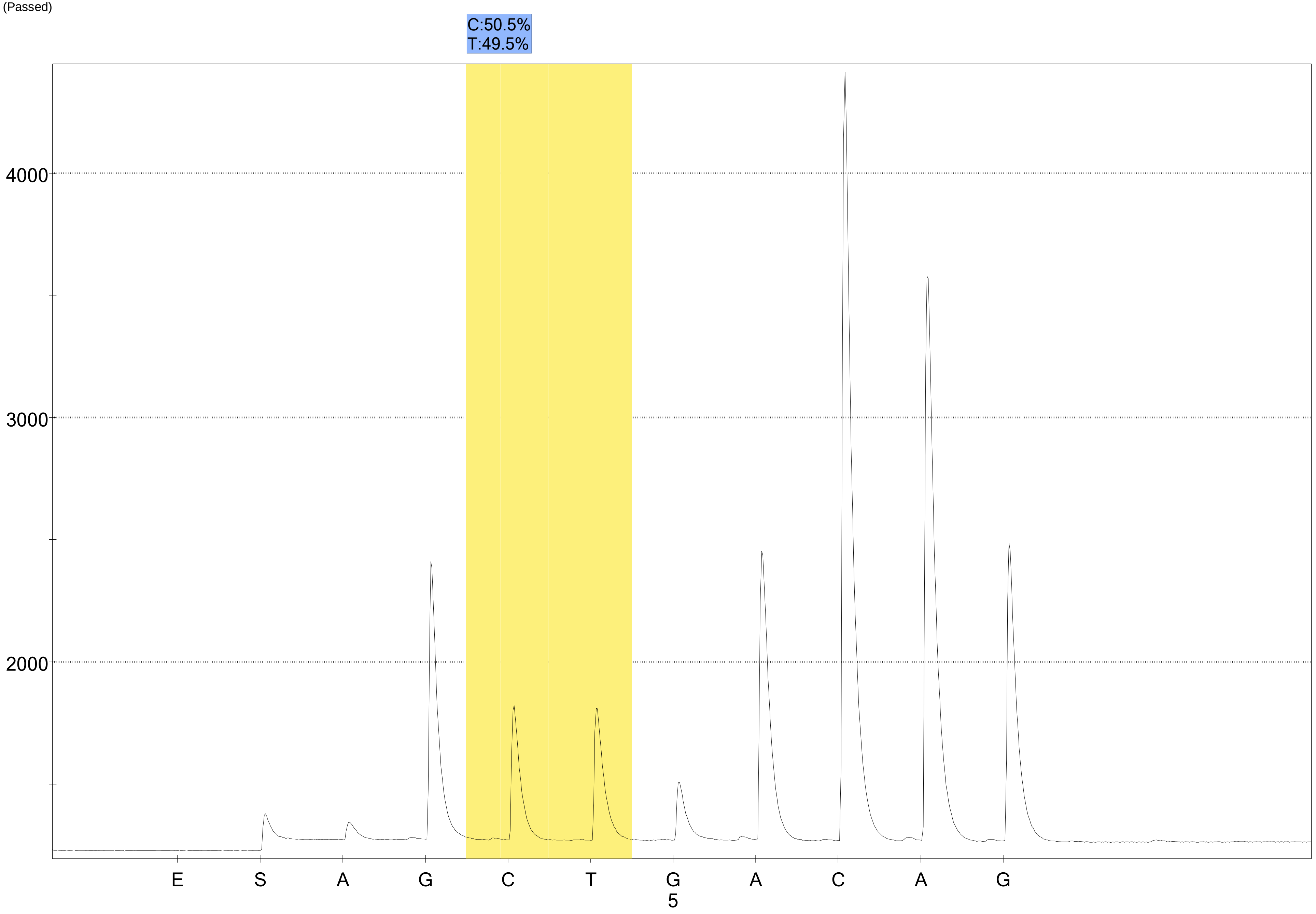

dna - Well A3
Entry: Cplx2
2: C: 46.1% / T: 53.9%
(Passed)

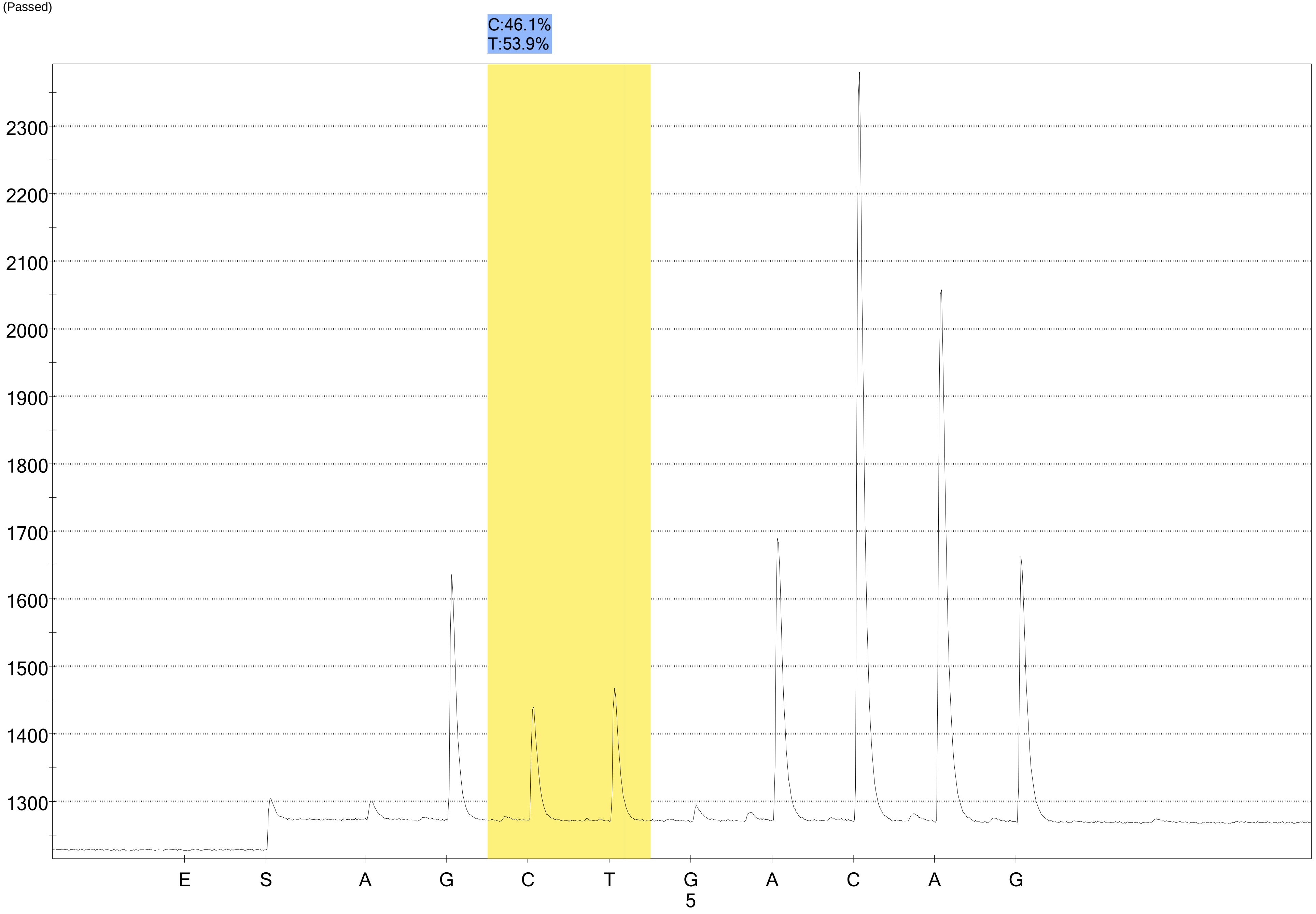

dna - Well A9
Entry: Cplx2
2: C: 48.7% / T: 51.3%
(Passed)

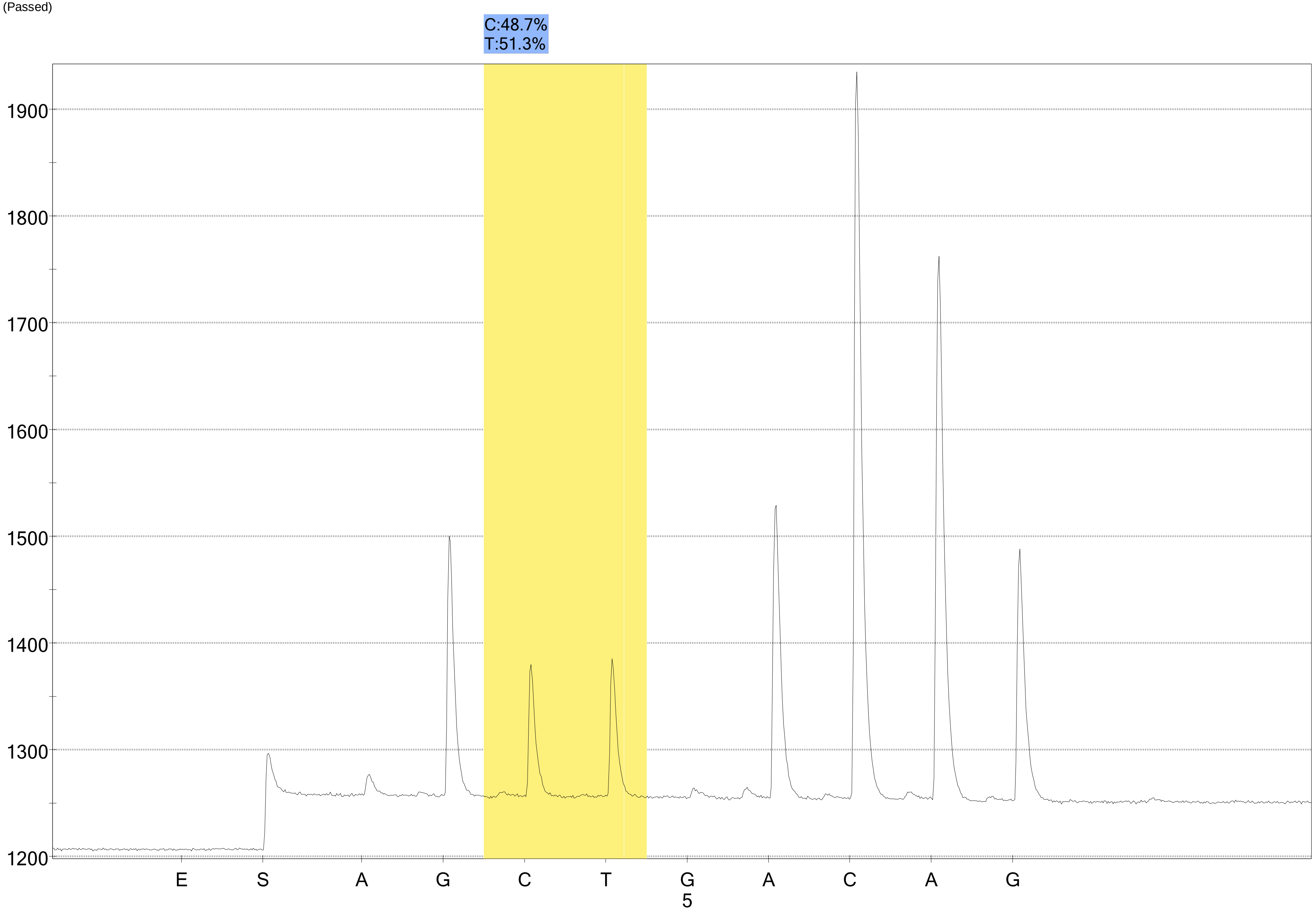

10 uL universal (141+157) - Well F2
Entry: Ccdc40
1: G: 54.8% / A: 45.2%
(Passed)

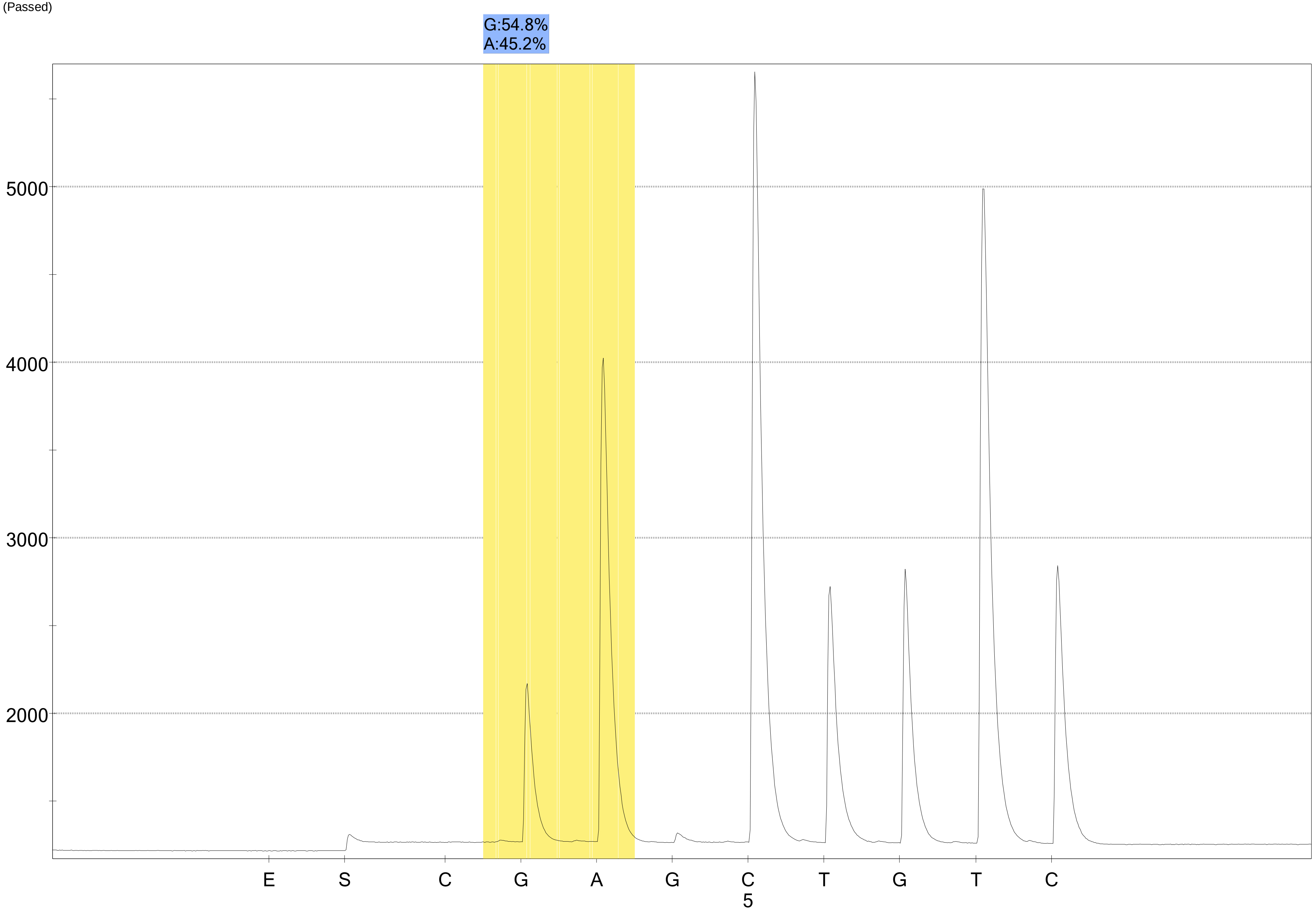

10 uL universal (141+157) - Well F8
Entry: Ccdc40
1: G: 43.9% / A: 56.1%
(Passed)

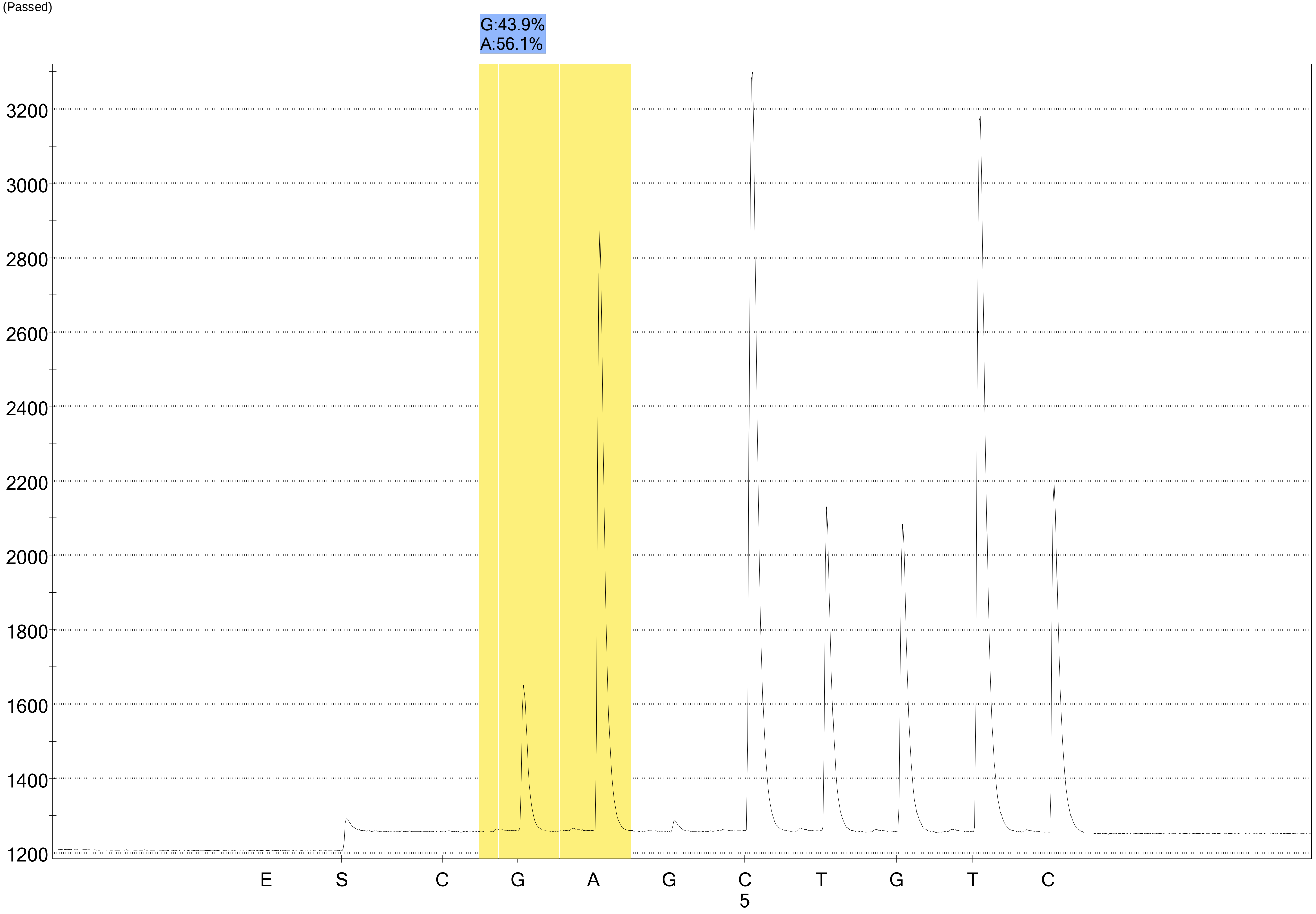

145 - Well F2
Entry: Ccdc40
1: G: 59.6% / A: 40.4%
(Passed)

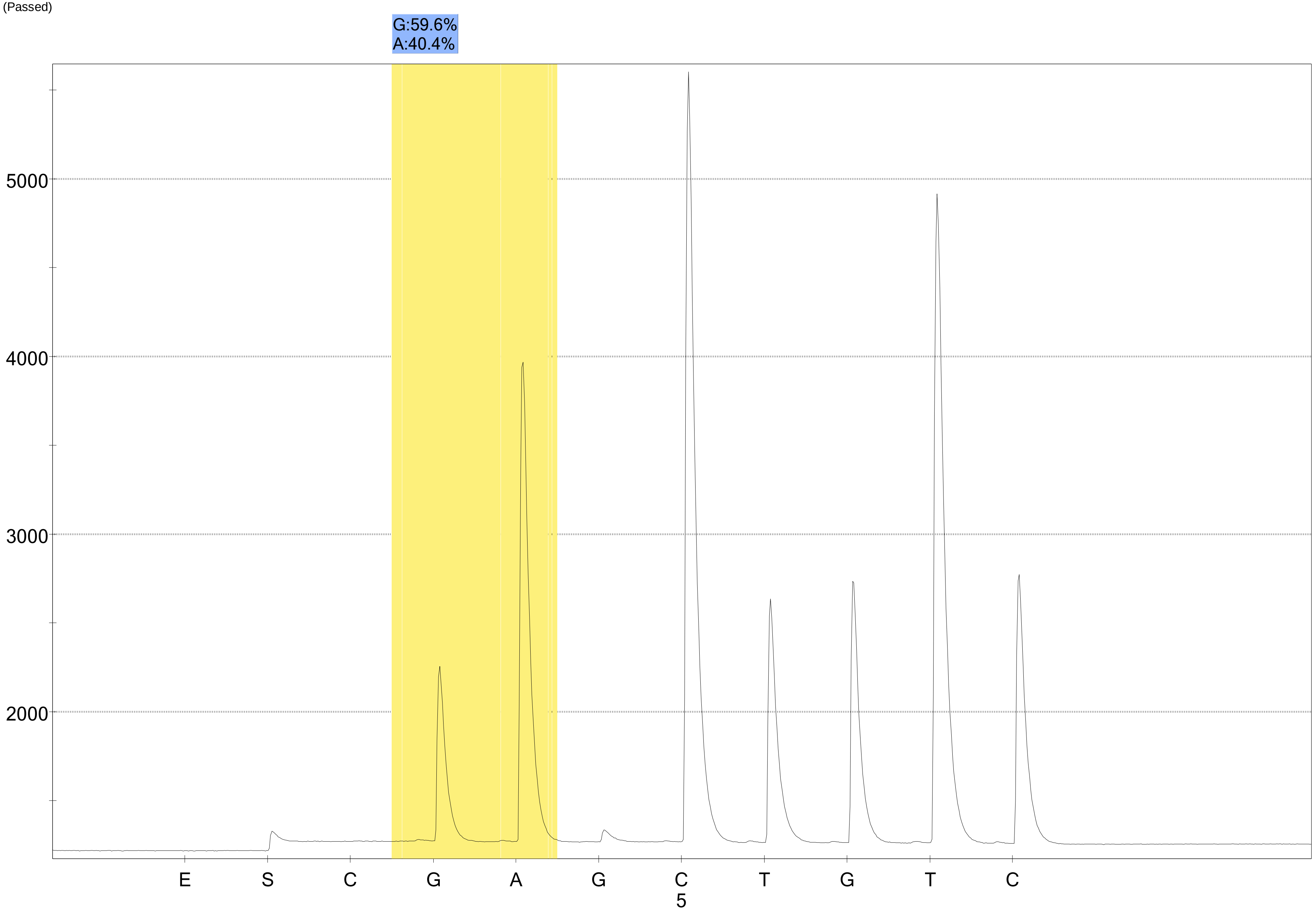

dna - Well F2
Entry: Ccdc40
1: G: 53.2% / A: 46.8%
(Passed)

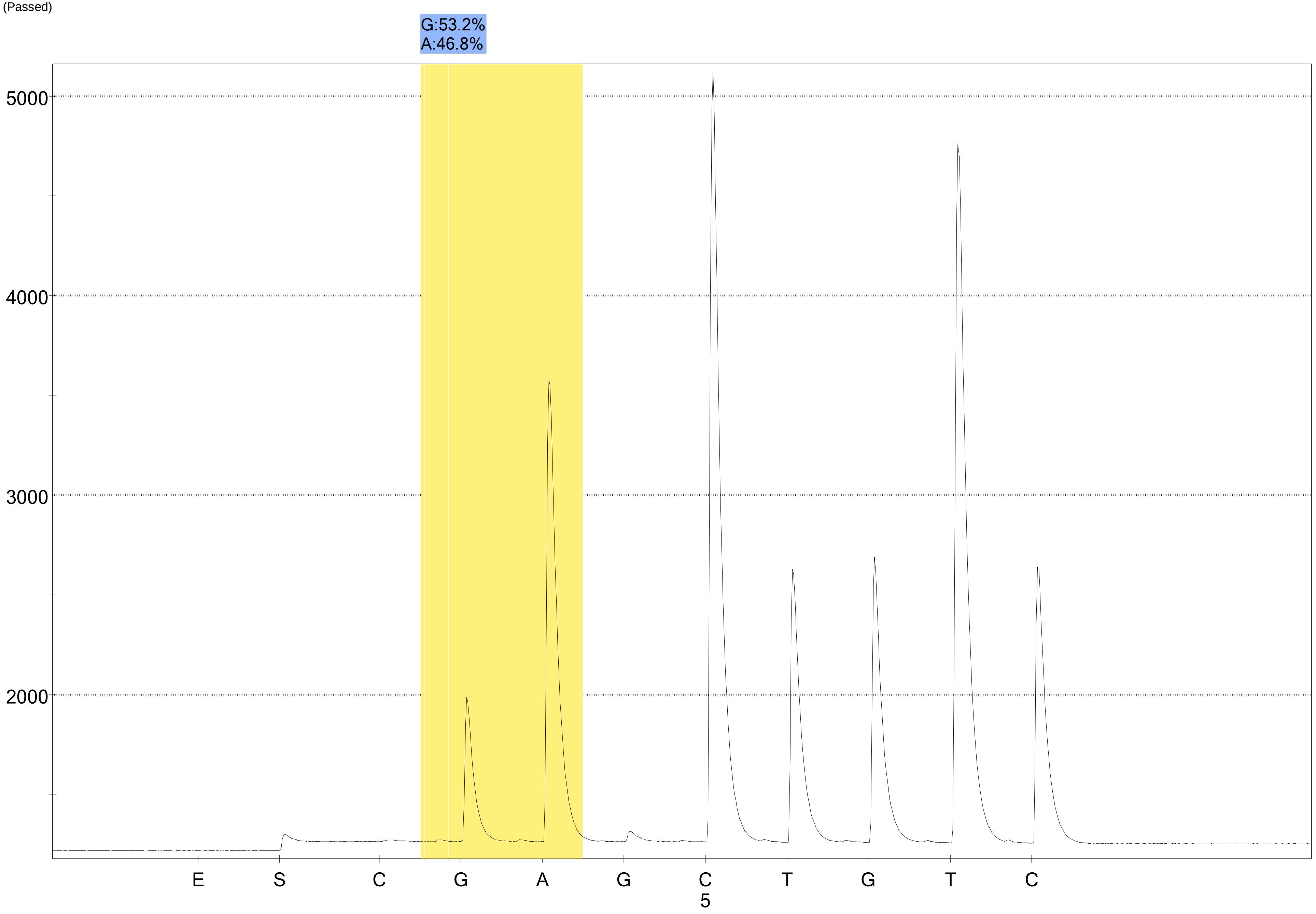

dna - Well F8
Entry: Ccdc40
1: G: 47.2% / A: 52.8%
(Passed)

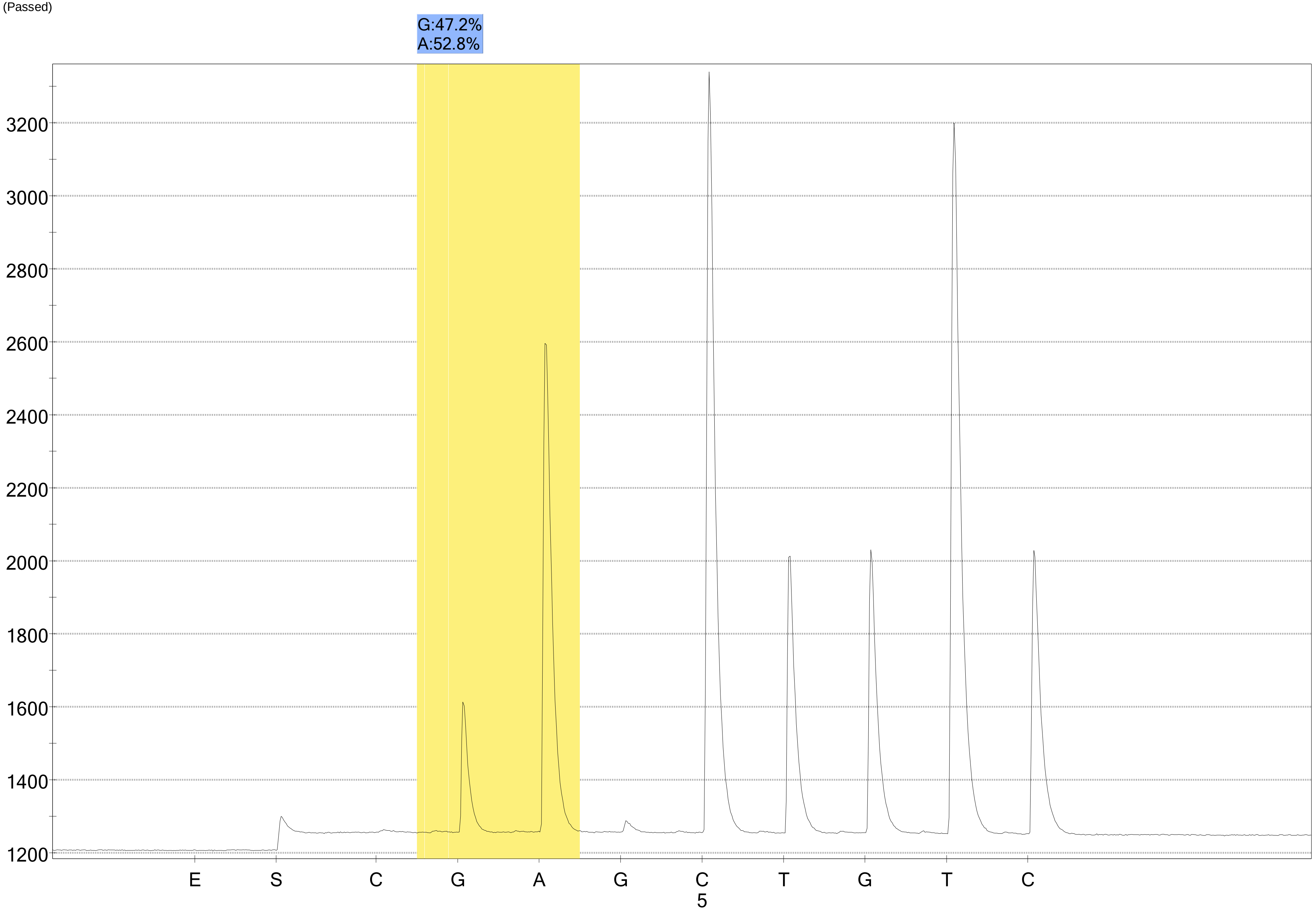

10 uL universal (141+157) - Well H11
Entry: Rhbd13
3: A: 52.4% / G: 47.6%
(Passed)

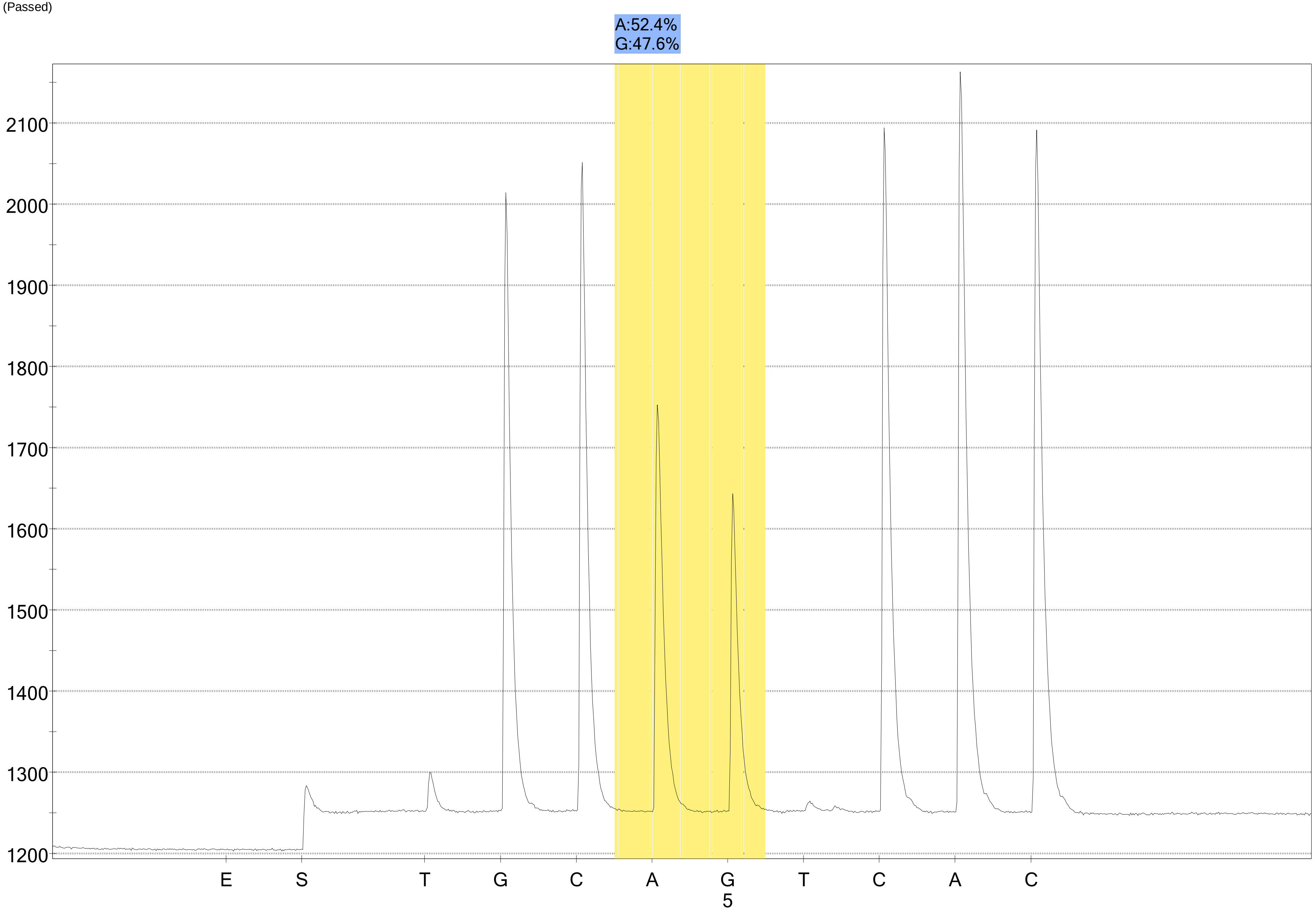

10 uL universal (141+157) - Well H5
Entry: Rhbd13
3: A: 51.3% / G: 48.7%
(Passed)

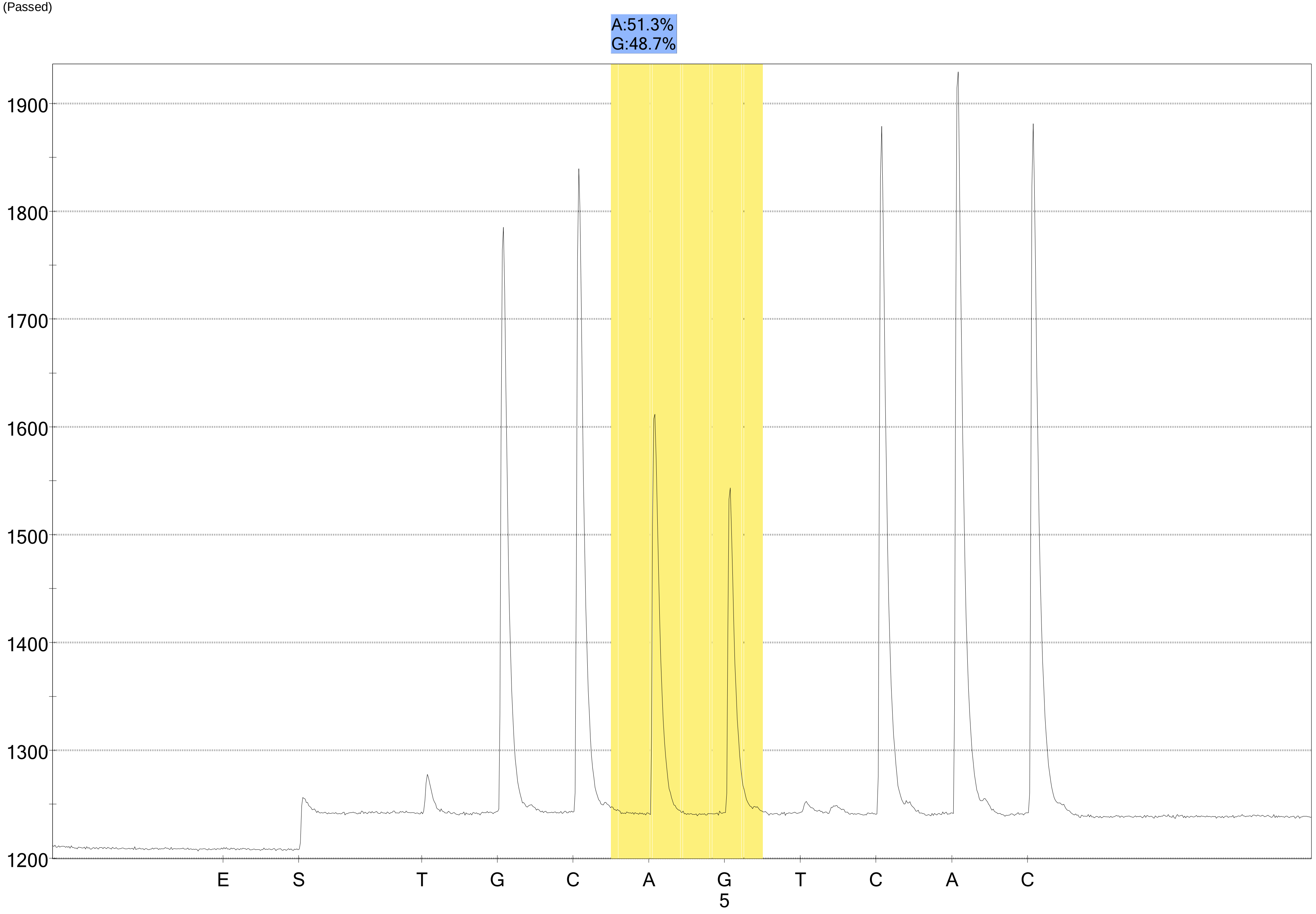

145 - Well H5
Entry: Rhbd13
3: A: 54.7% / G: 45.3%
(Passed)

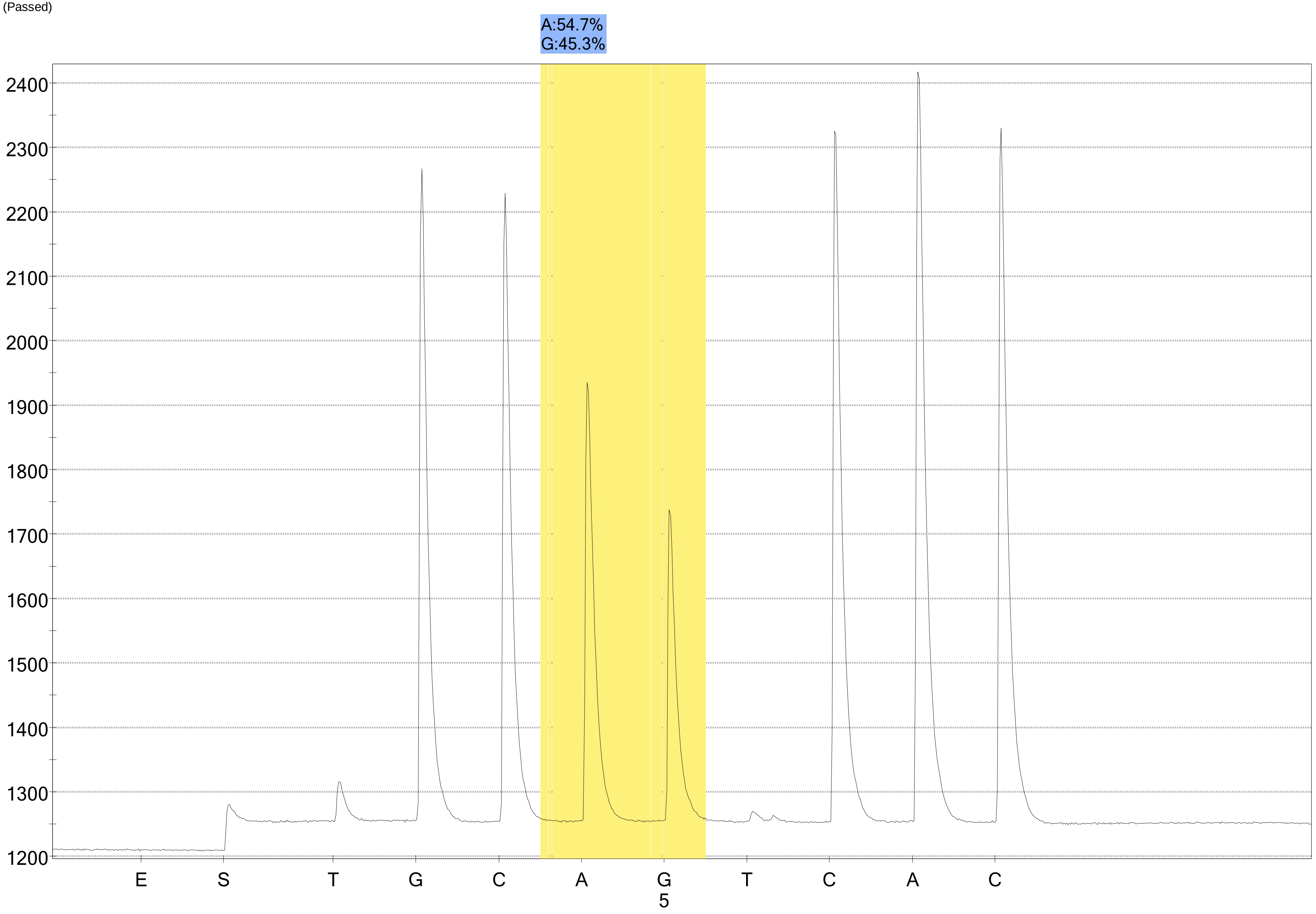

dna - Well H5
Entry: Rhbd13
3: A: 54.8% / G: 45.2%
(Passed)

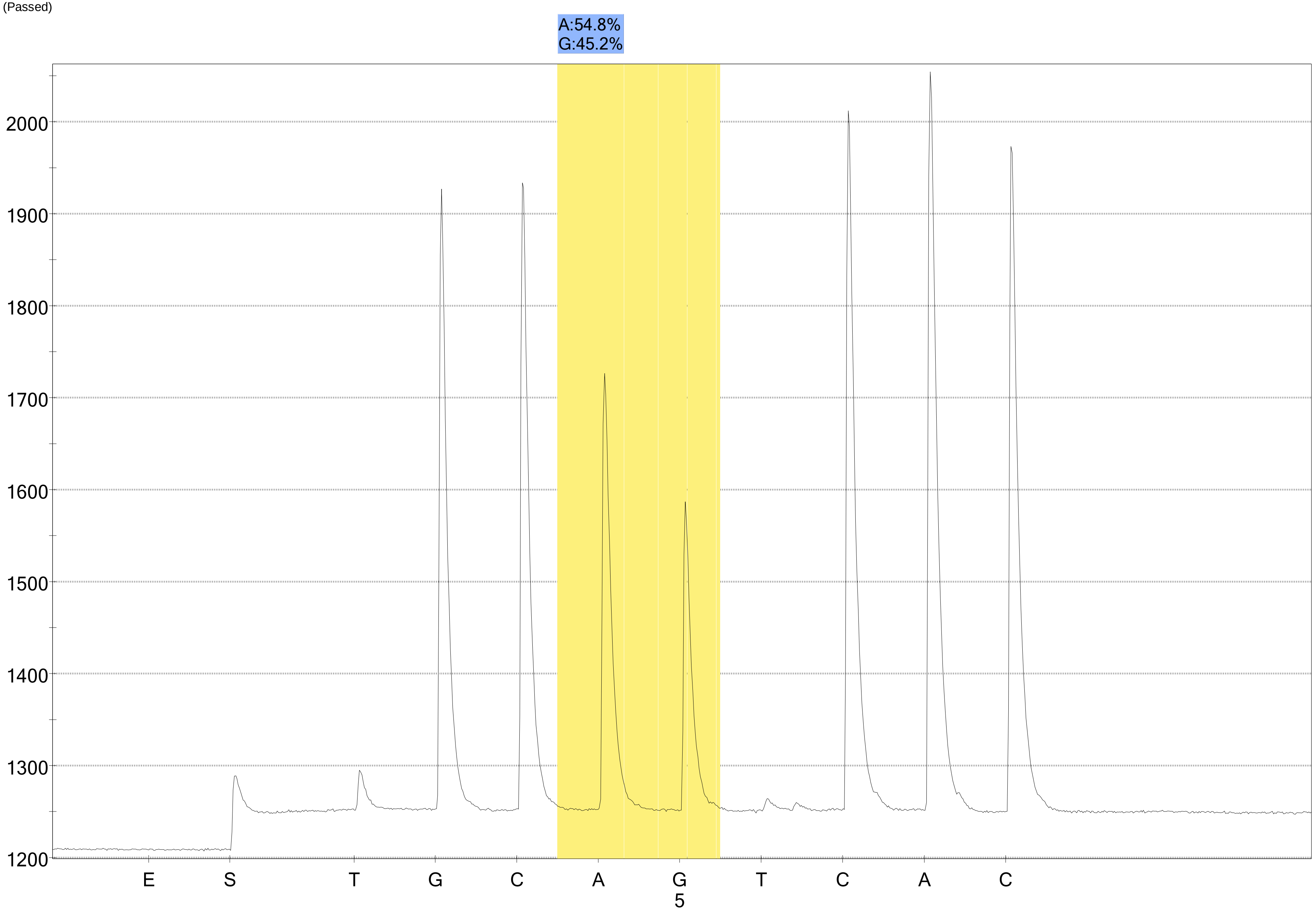

dna - Well H11
Entry: Rhbd13
3: A: 51.3% / G: 48.7%
(Passed)

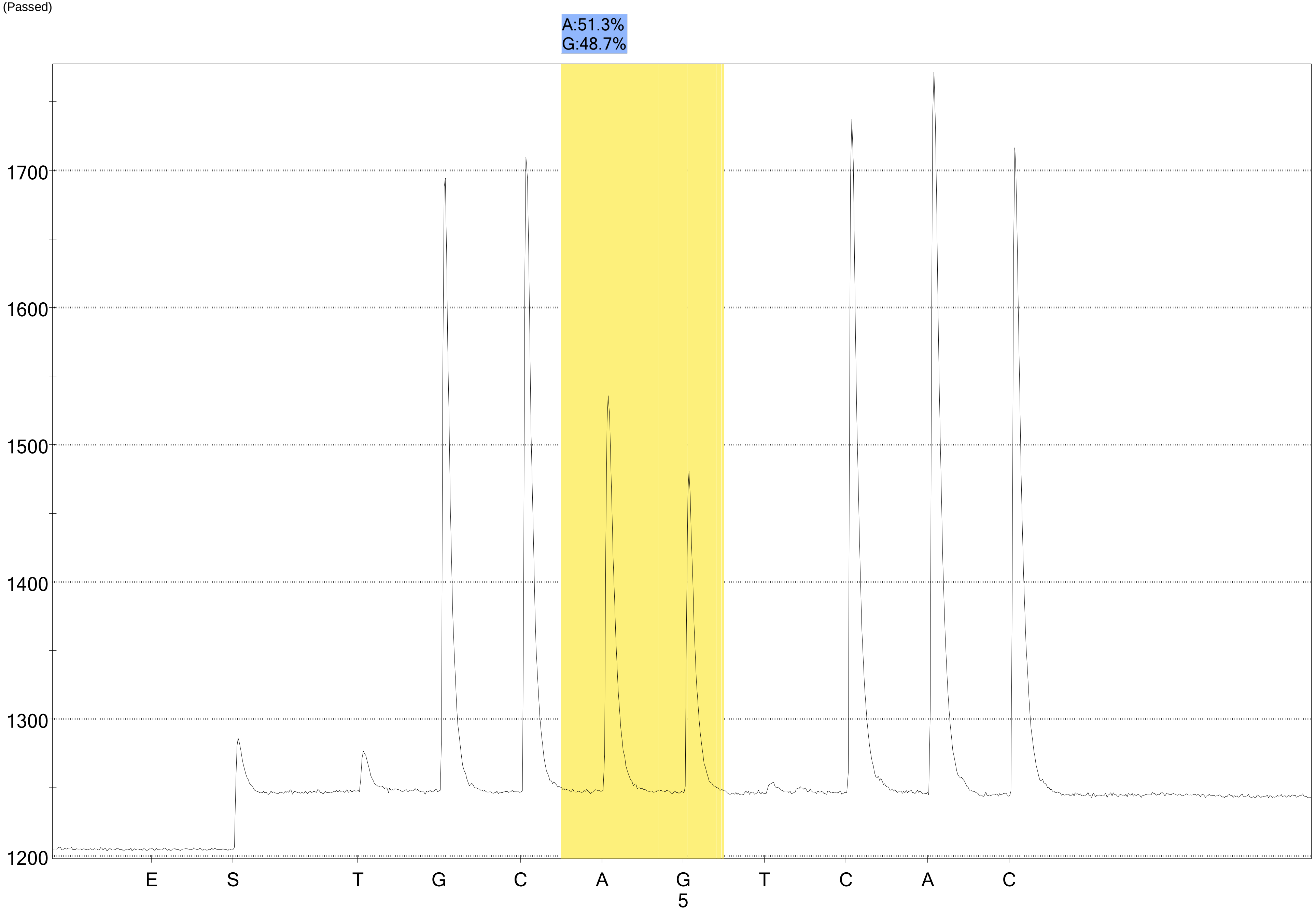

10 uL universal (141+157) - Well A2
Entry: Bcl2l1
1: C: 24.6% / T: 75.4%
(Passed)

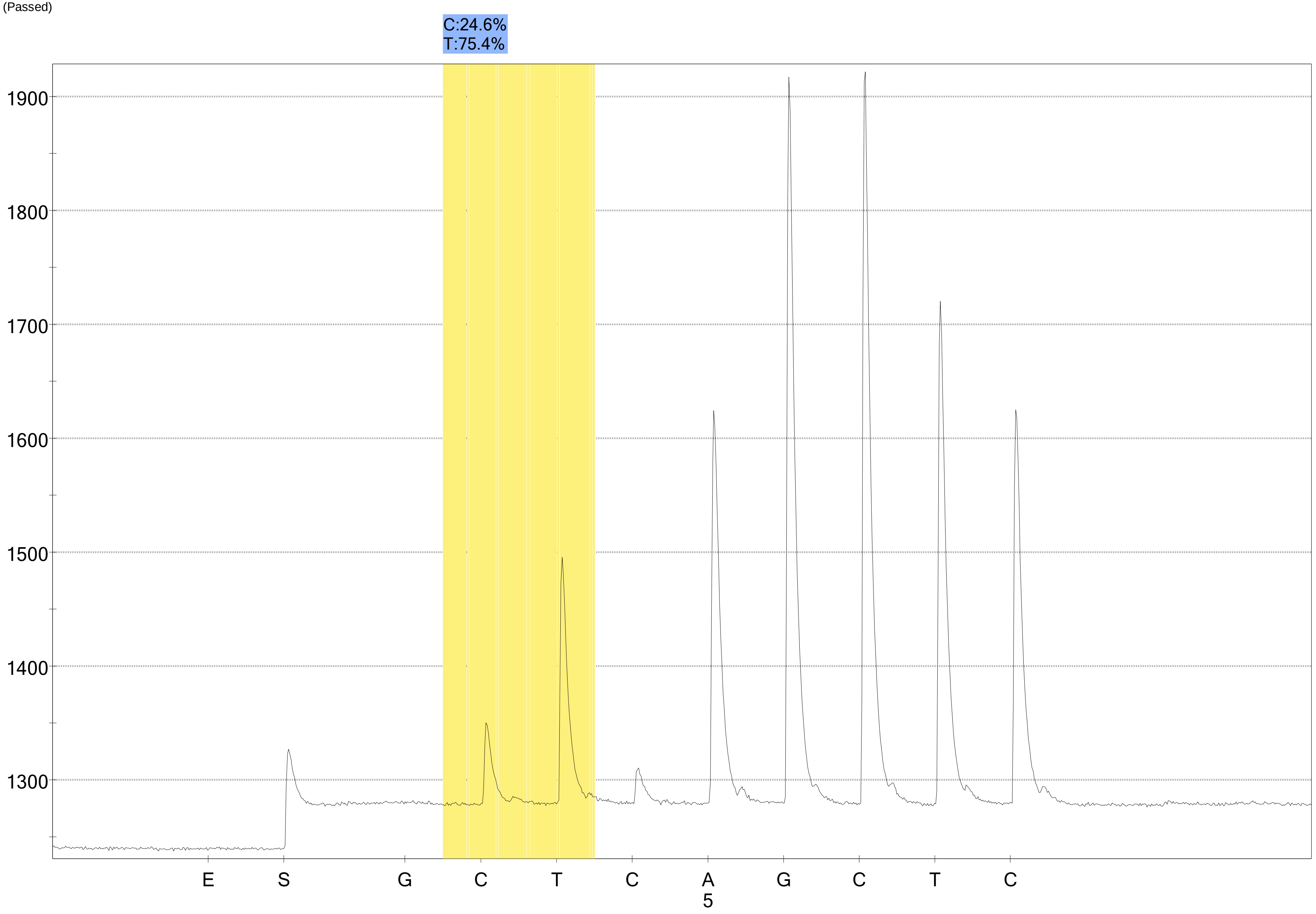

10 uL universal (141+157) - Well A8
Entry: Bcl2l1
1: C: 49.5% / T: 50.5%
(Passed)

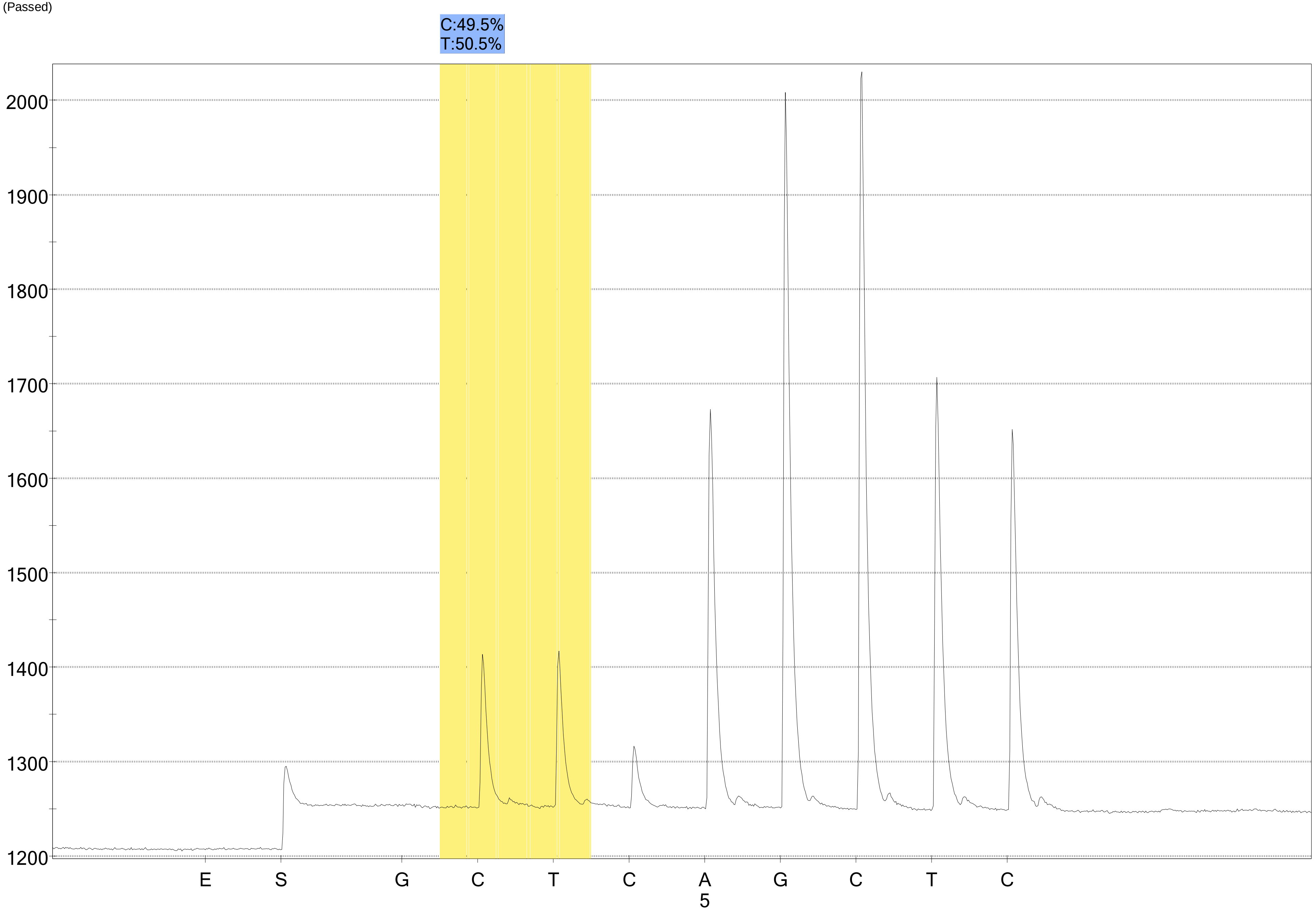

145 - Well A2
Entry: Bcl2l1
1: C: 27.9% / T: 72.1%
(Passed)

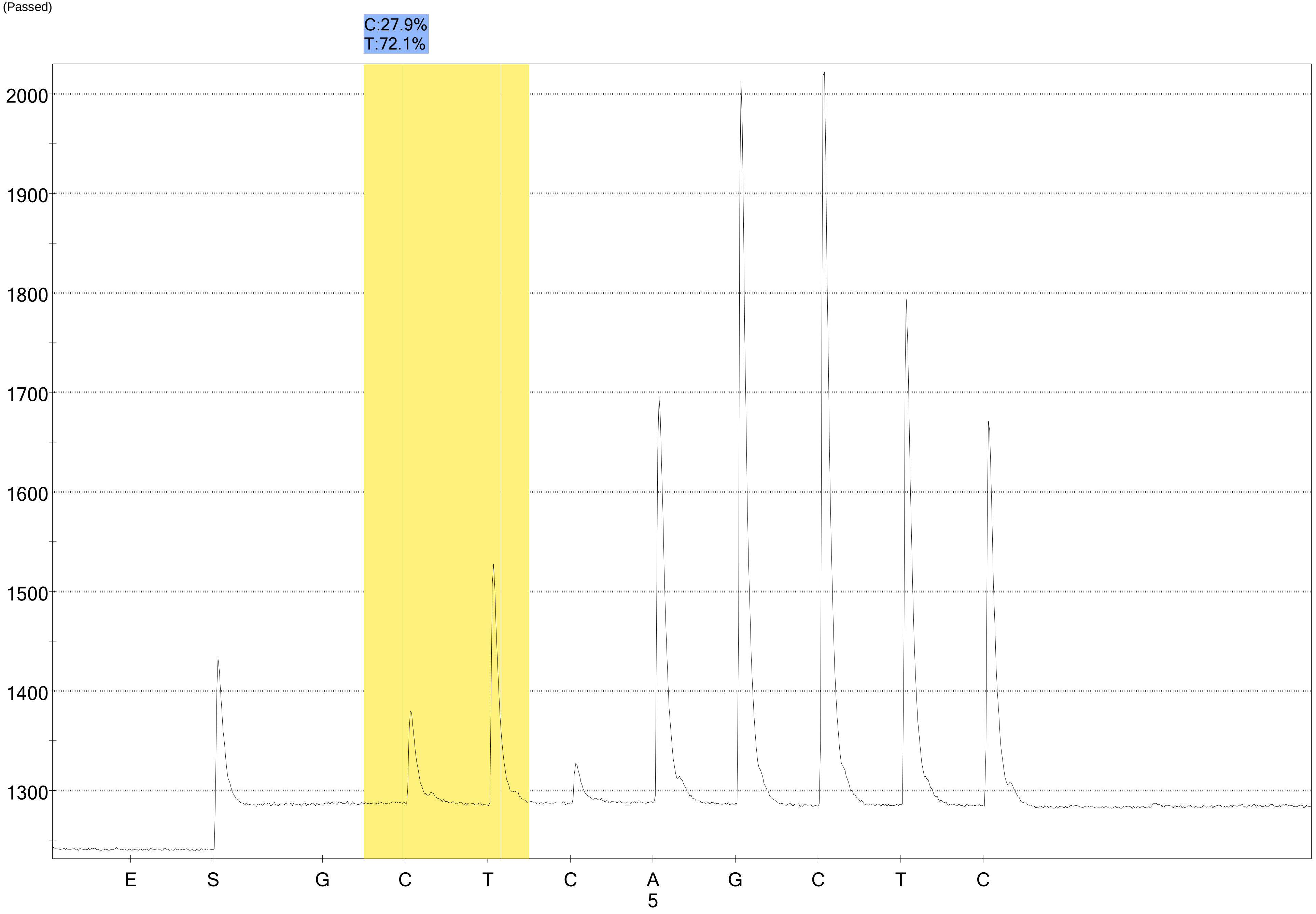

dna - Well A2
Entry: Bcl2l1
1: C: 41.6% / T: 58.4%
(Passed)

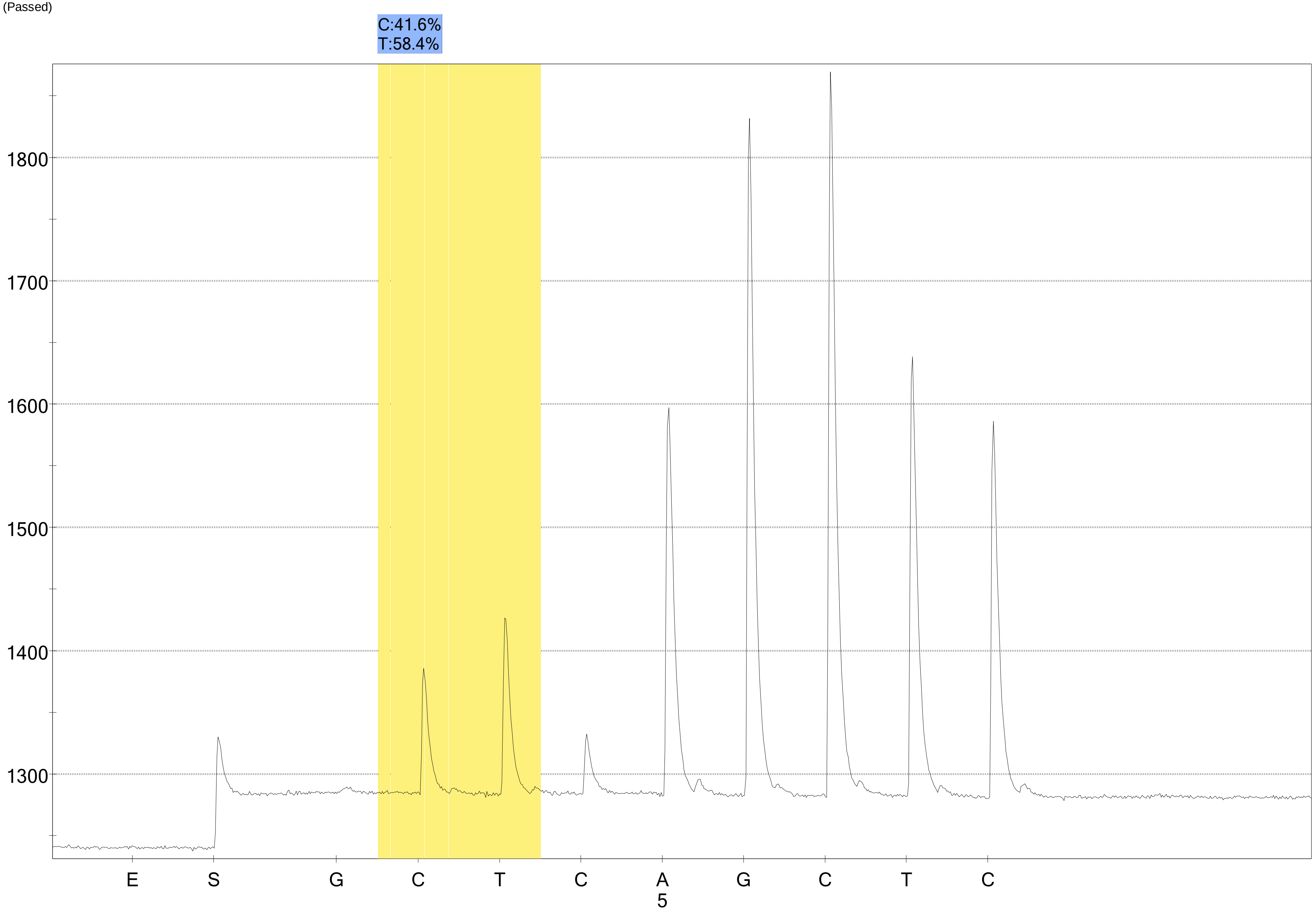

dna - Well A8
Entry: Bcl2l1
1: C: 42.9% / T: 57.1%
(Passed)

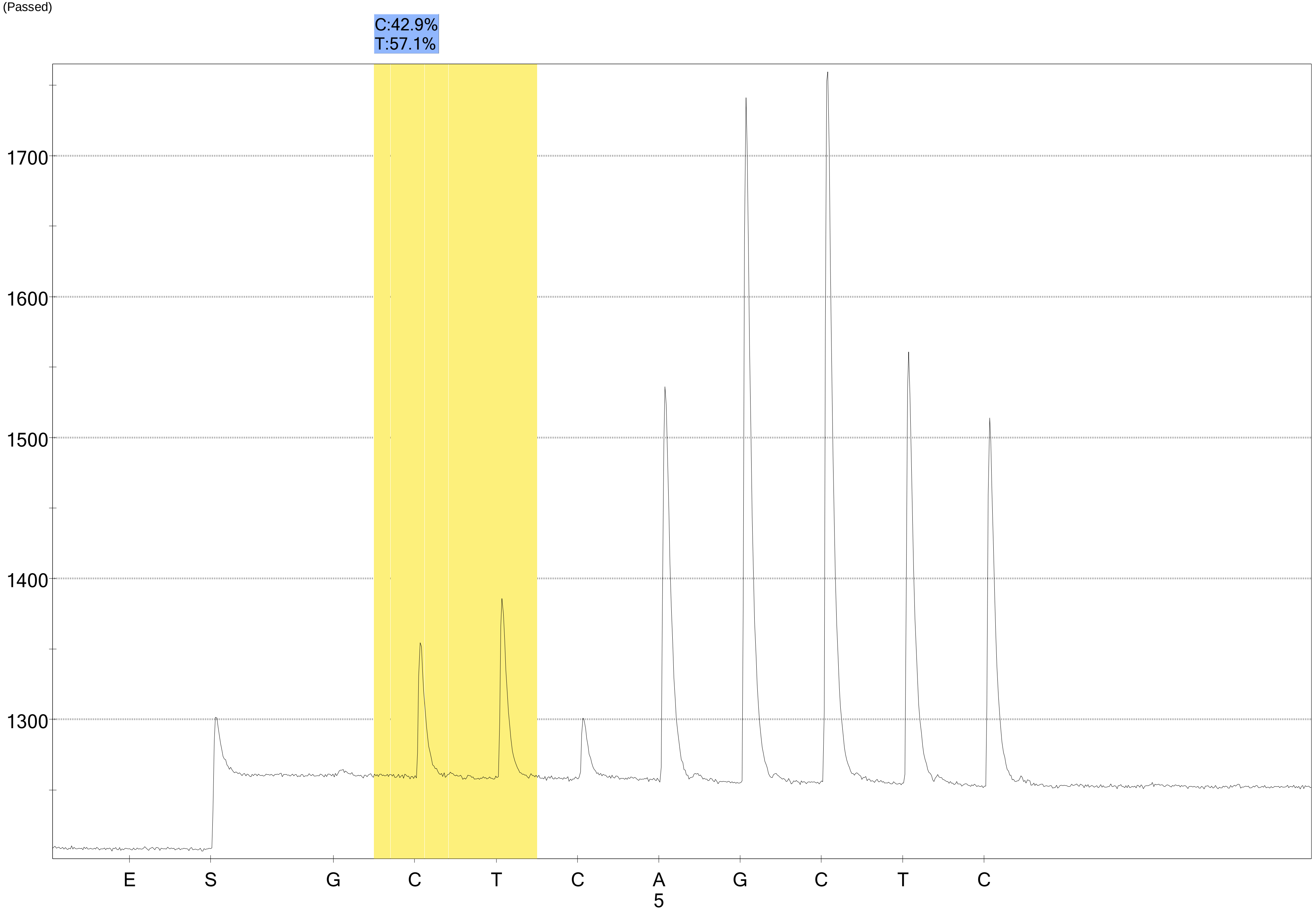

10 uL universal (141+157) - Well B5
Entry: Nsg1
3: C: 41.1% / T: 58.9%
(Passed)

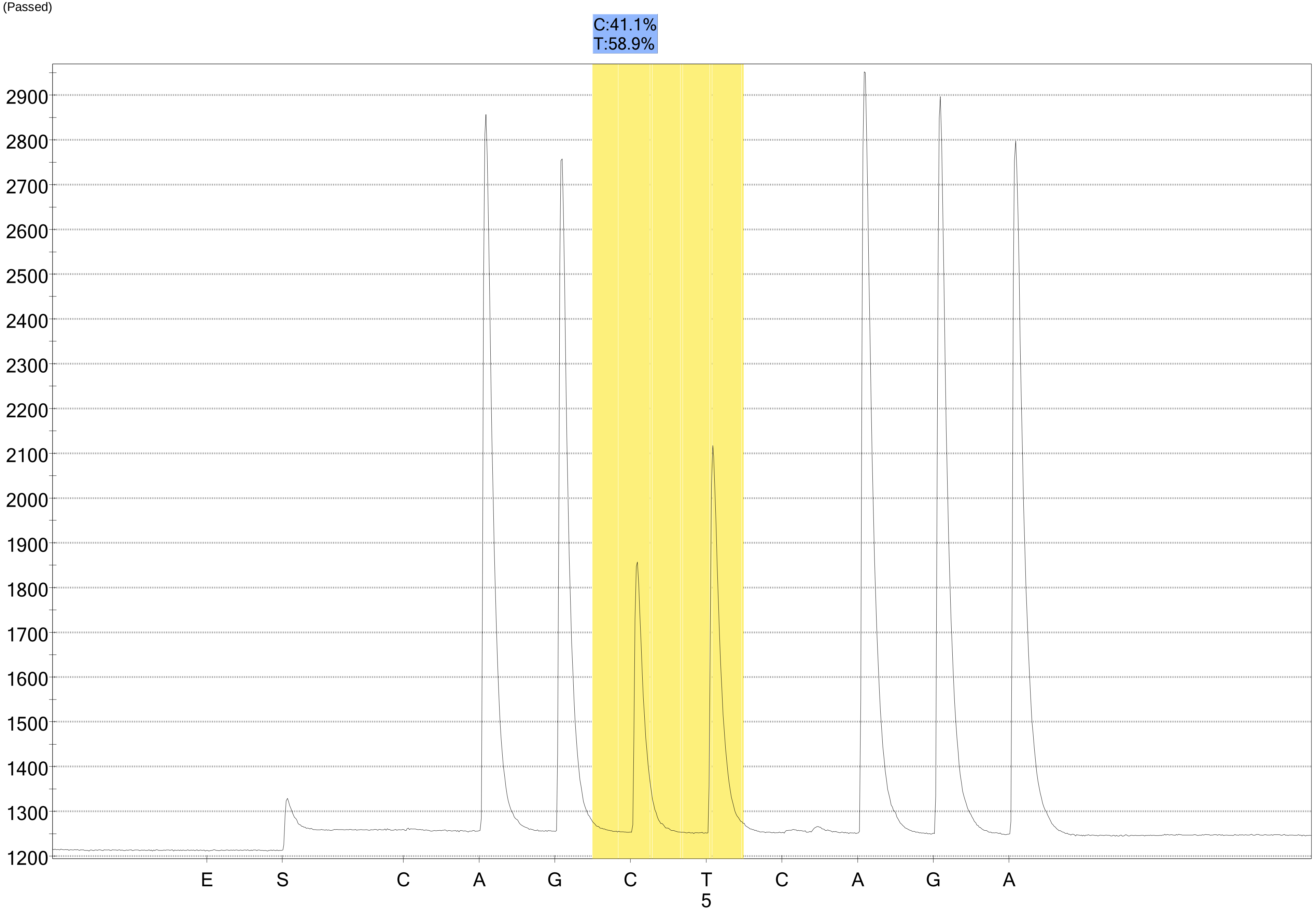

10 uL universal (141+157) - Well B11
Entry: Nsg1
3: C: 41.3% / T: 58.7%
(Passed)

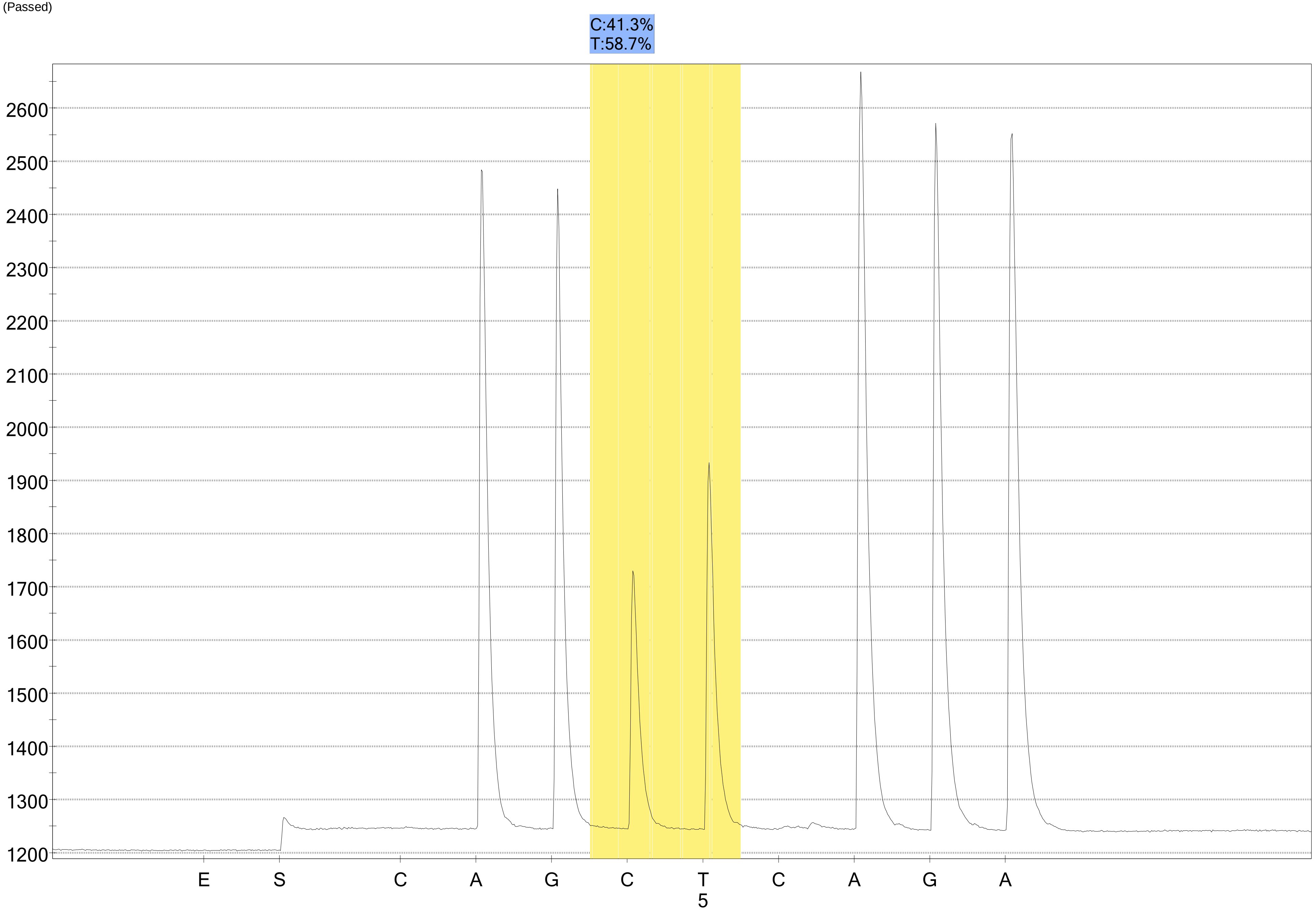

145 - Well B5
Entry: Nsg1
3: C: 41.6% / T: 58.4%
(Passed)

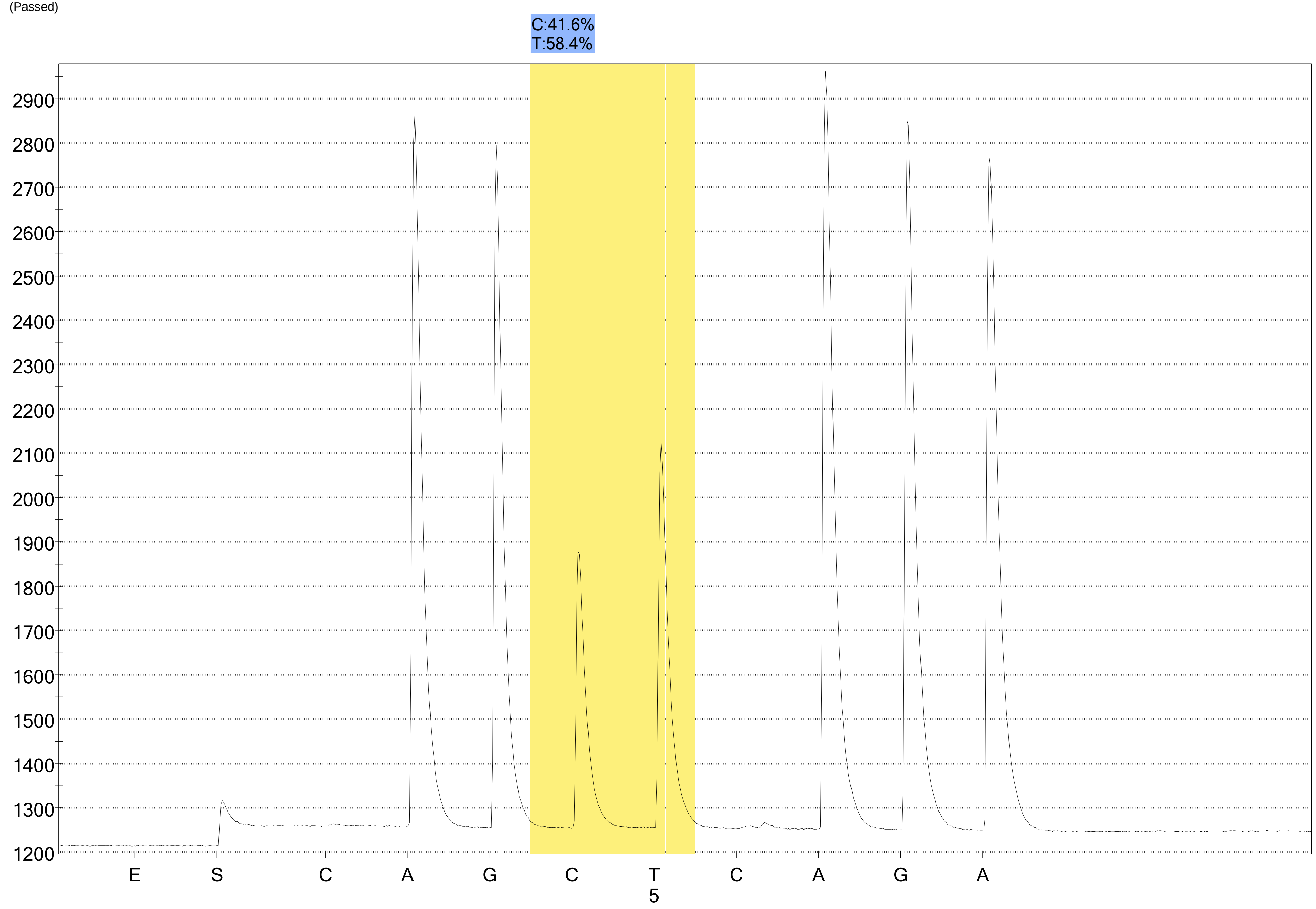

dna - Well B5
Entry: Nsg1
3: C: 49.6% / T: 50.4%
(Passed)

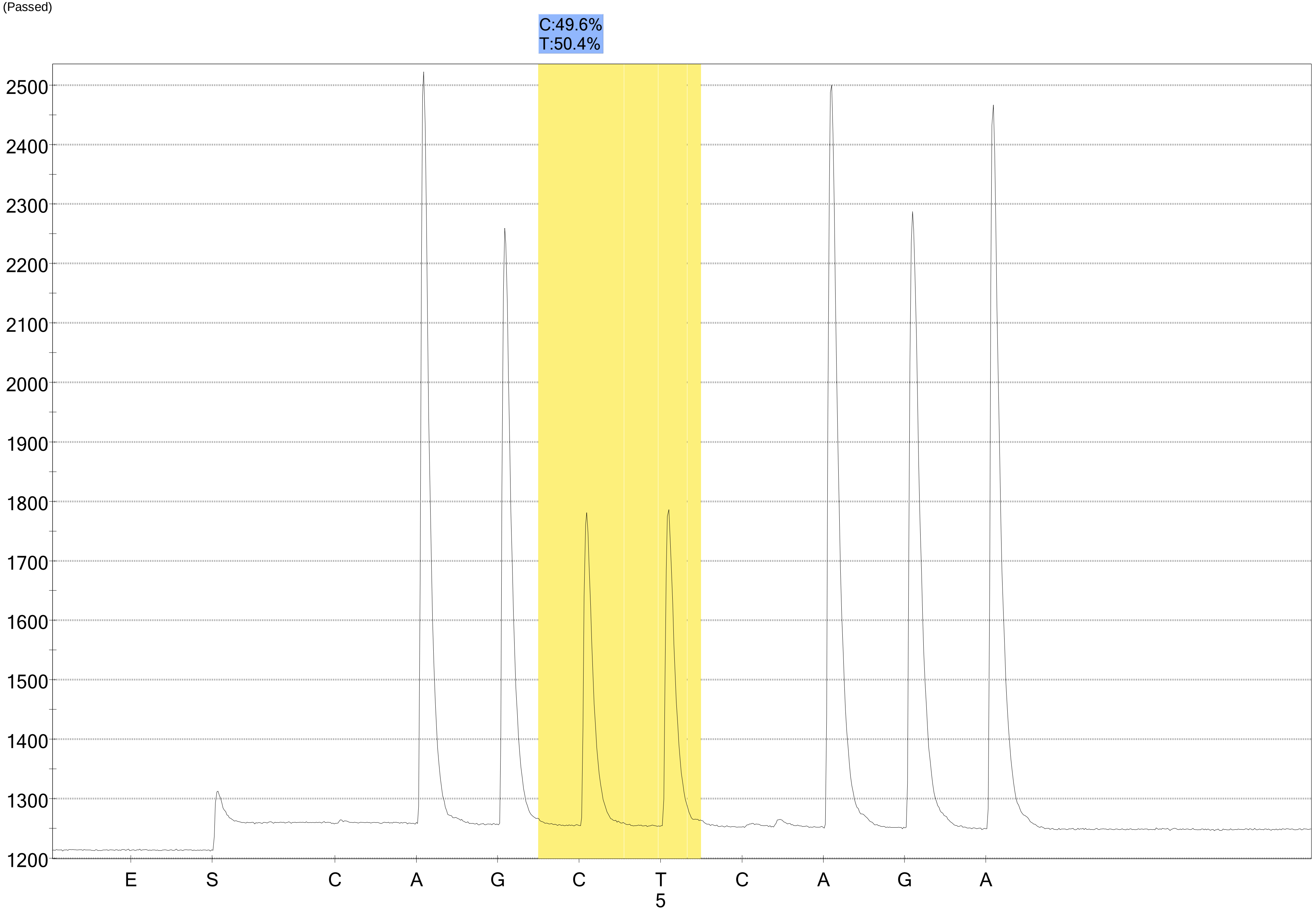

dna - Well B11
Entry: Nsg1
3: C: 51.1% / T: 48.9%
(Passed)

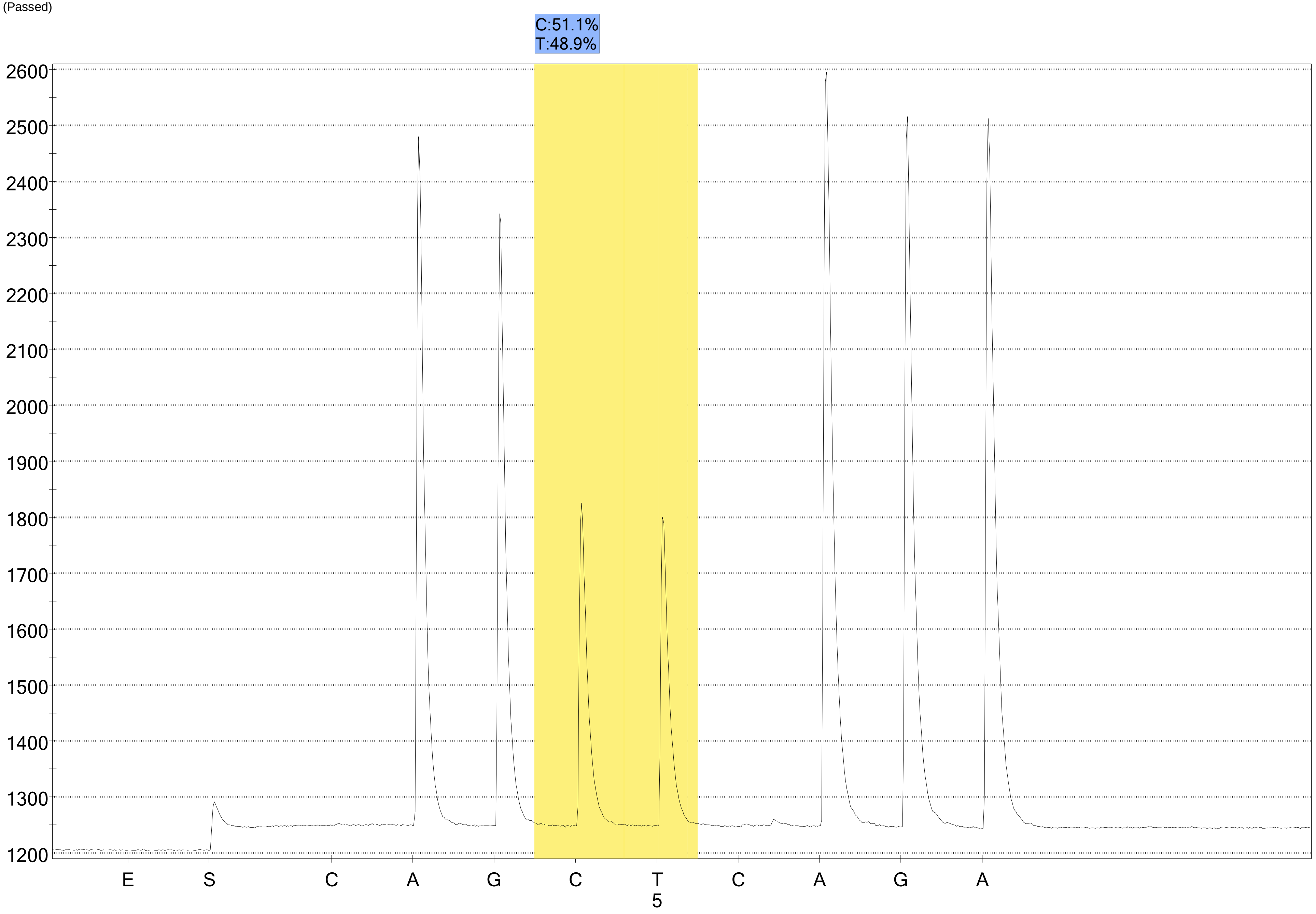

10 uL universal (141+157) - Well F4
Entry: Malat1
4: T: 44.7% / C: 55.3%
(Passed)

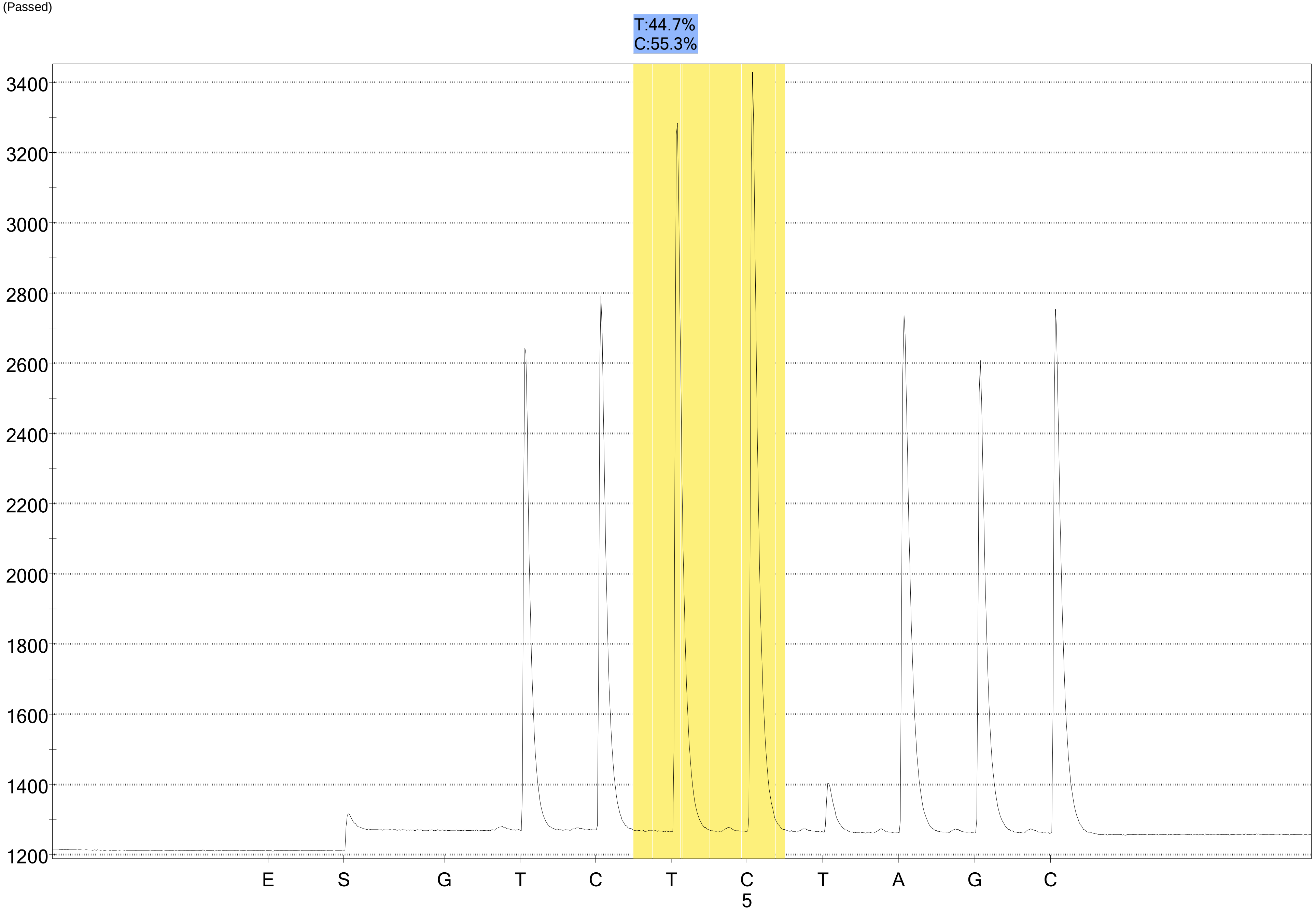

10 uL universal (141+157) - Well F10
Entry: Malat1
4: T: 48.7% / C: 51.3%
(Passed)

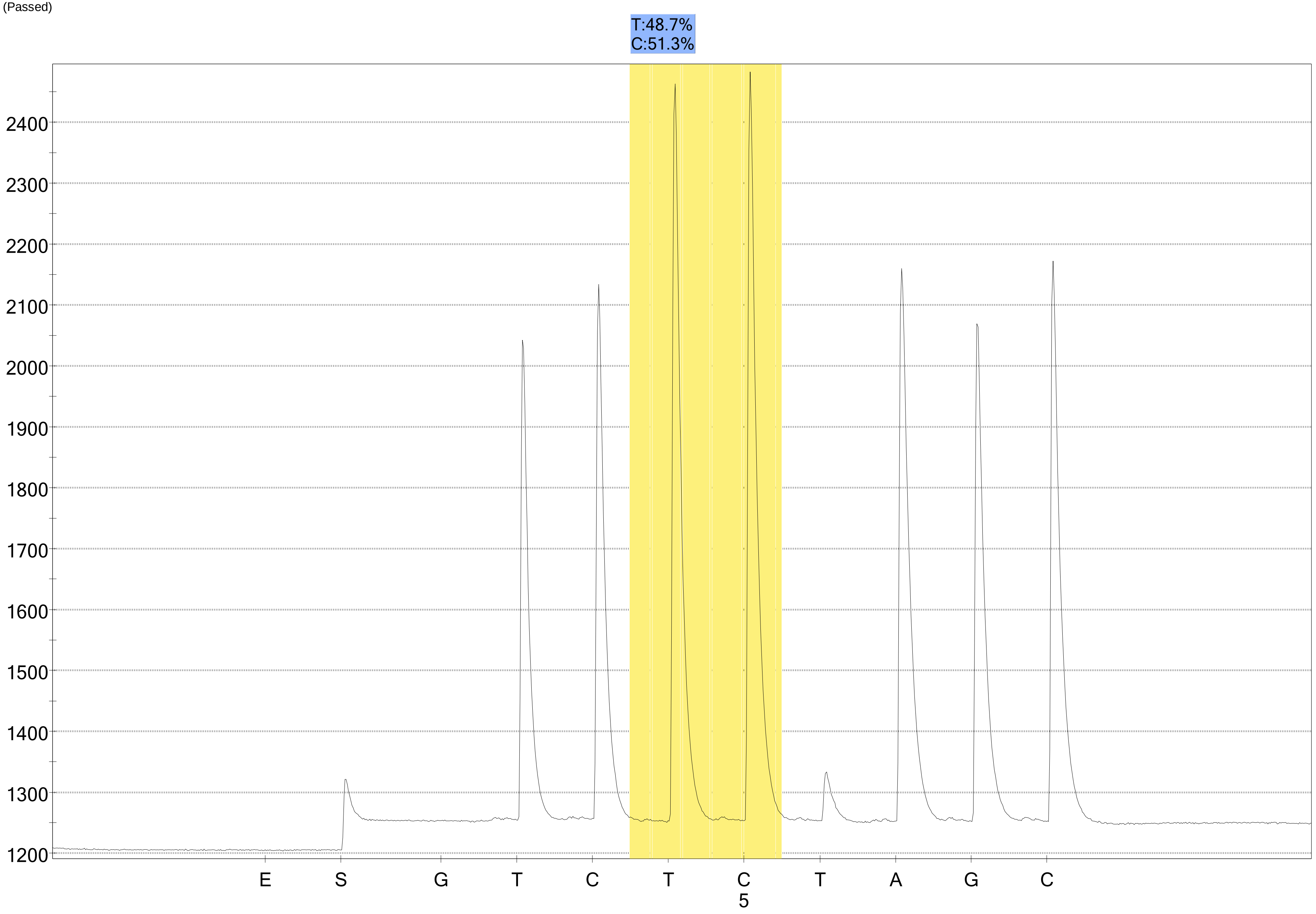

145 - Well F4
Entry: Malat1
4: T: 44.1% / C: 55.9%
(Passed)

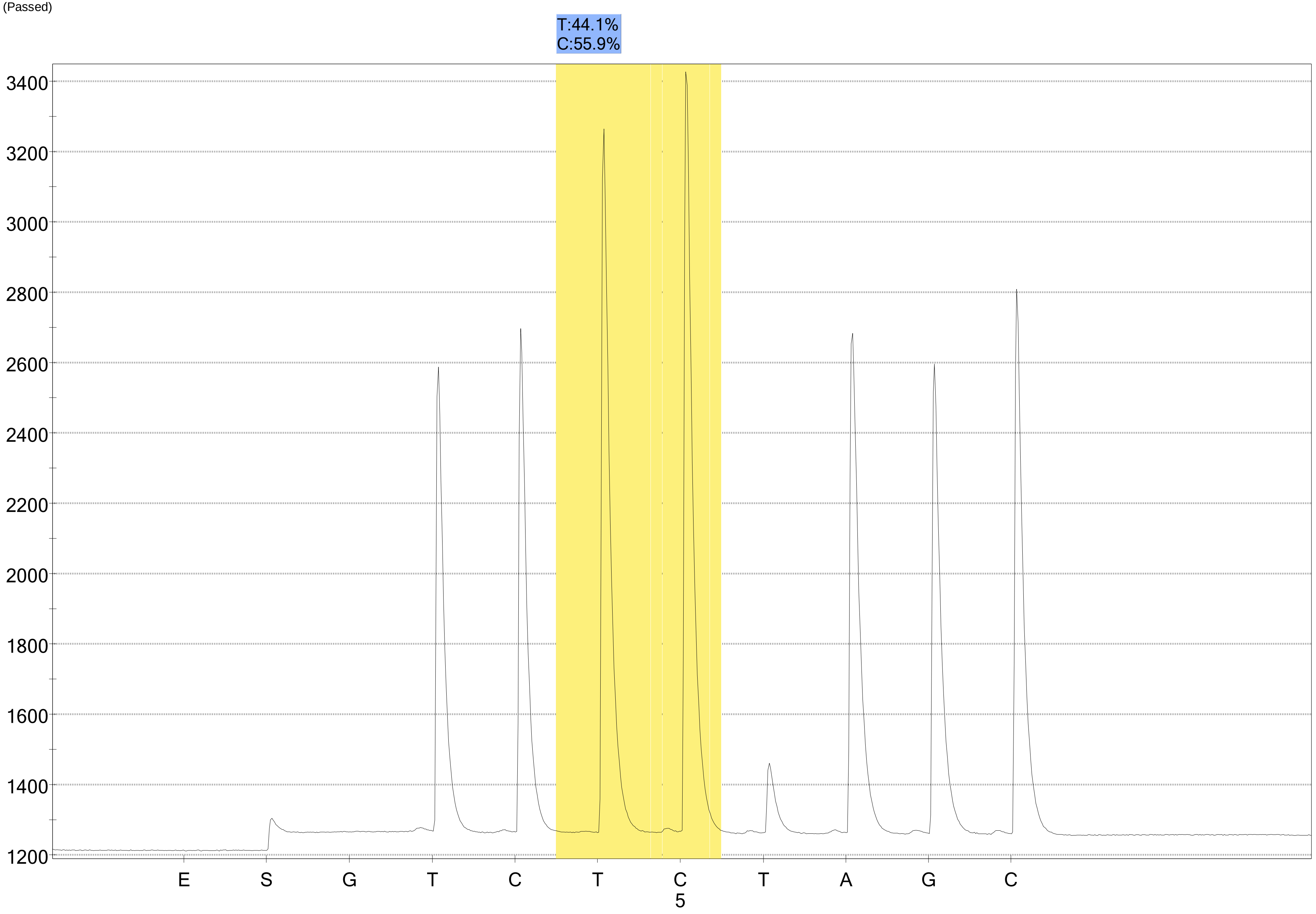

dna - Well F4
Entry: Malat1
4: T: 43.9% / C: 56.1%
(Passed)

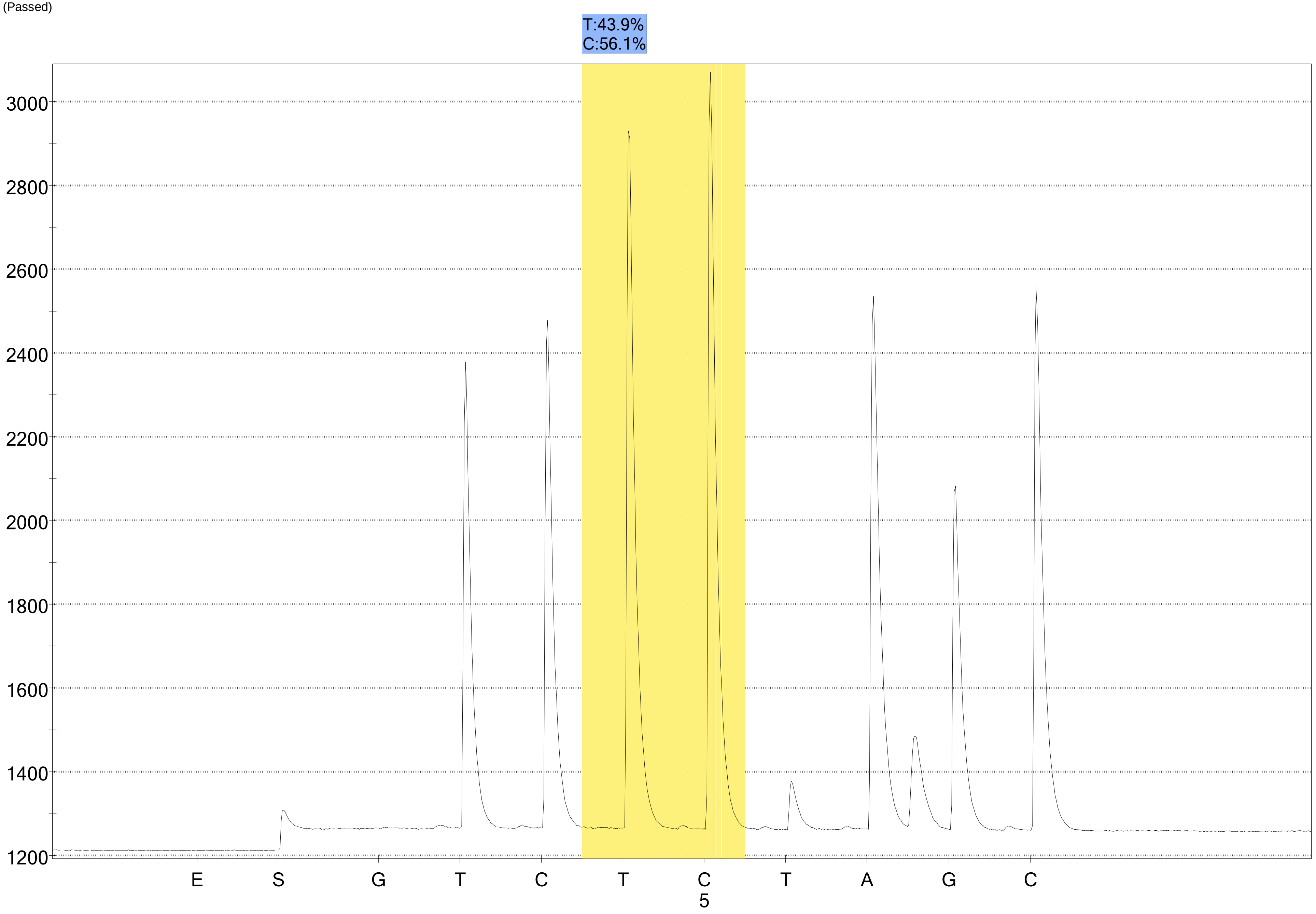

dna - Well F10
Entry: Malat1
4: T: 48.7% / C: 51.3%
(Passed)

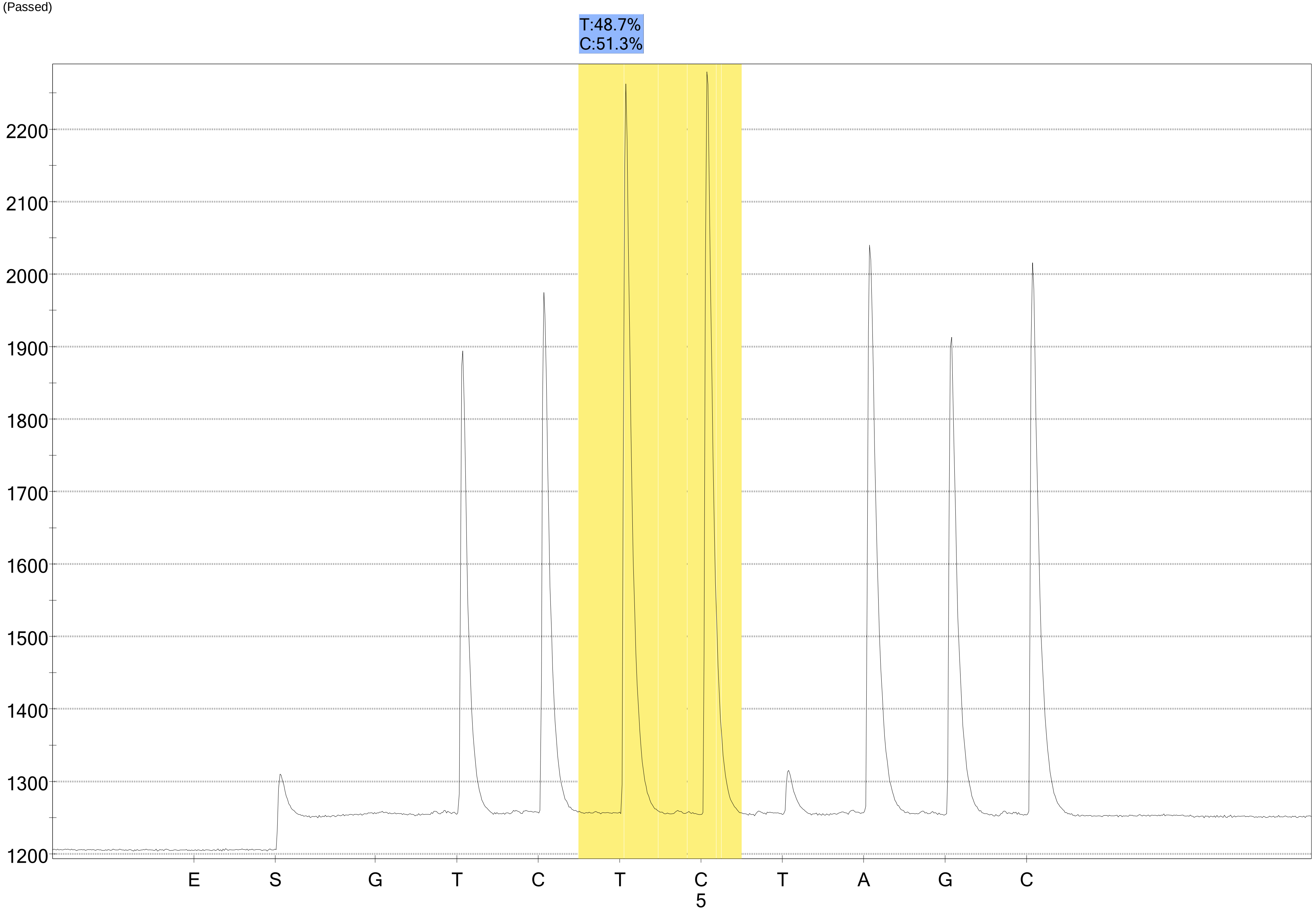

10 uL universal (141+157) - Well G4
Entry: Mapt
2: C: 56.7% / T: 43.3%
(Passed)

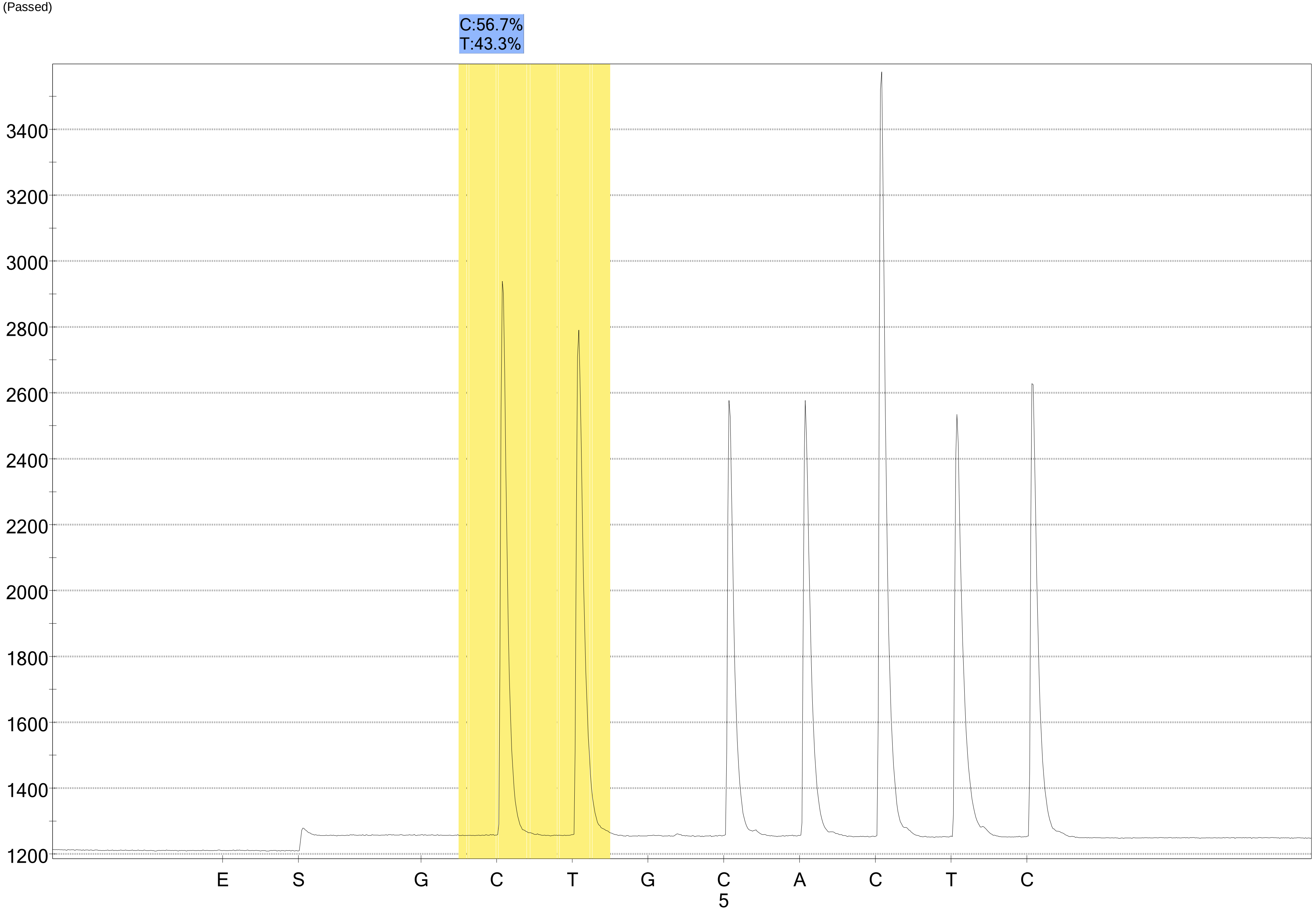

10 uL universal (141+157) - Well G10
Entry: Mapt
2: C: 49.5% / T: 50.5%
(Passed)

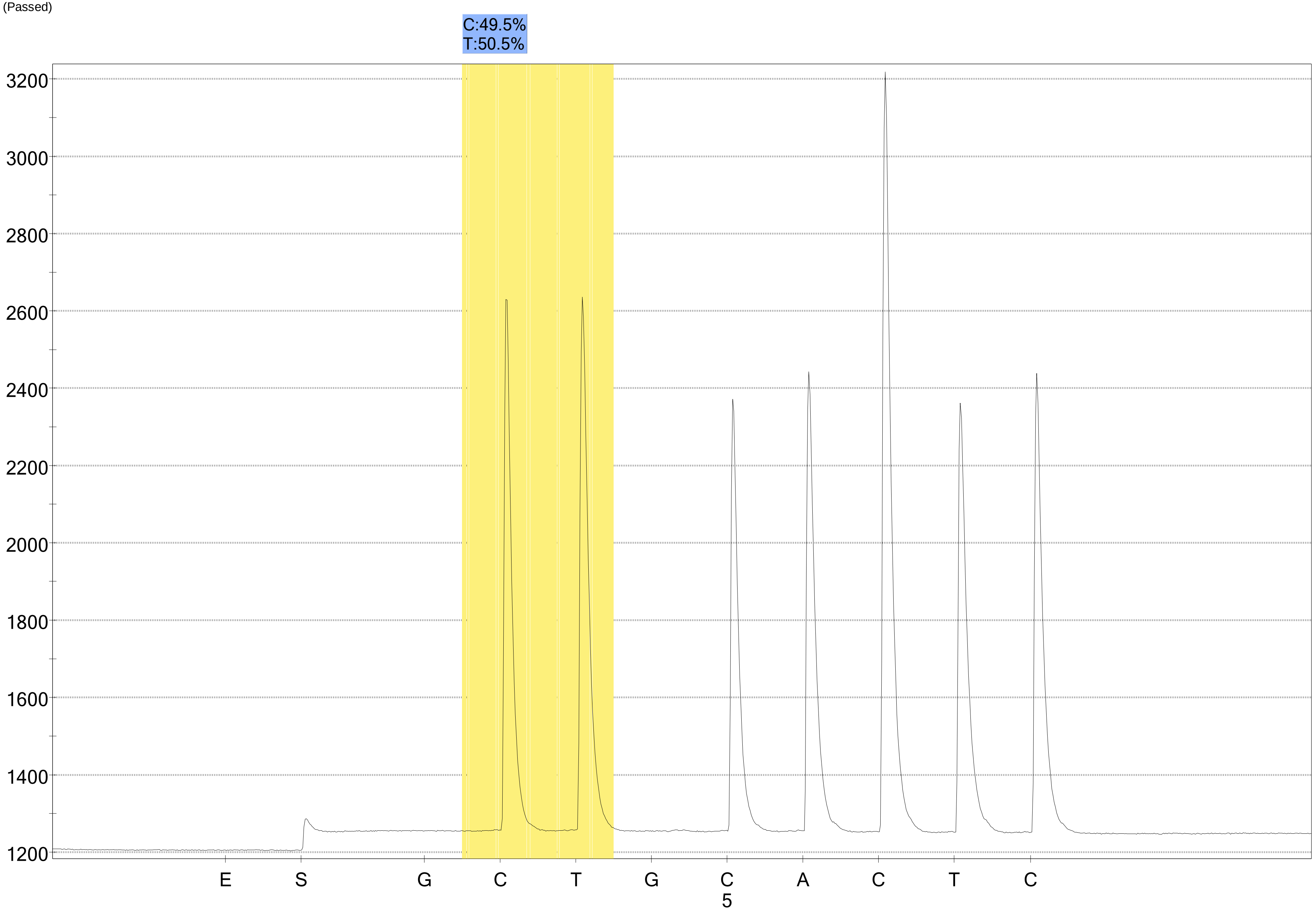

145 - Well G4
Entry: Mapt
2: C: 61.0% / T: 39.0%
(Passed)

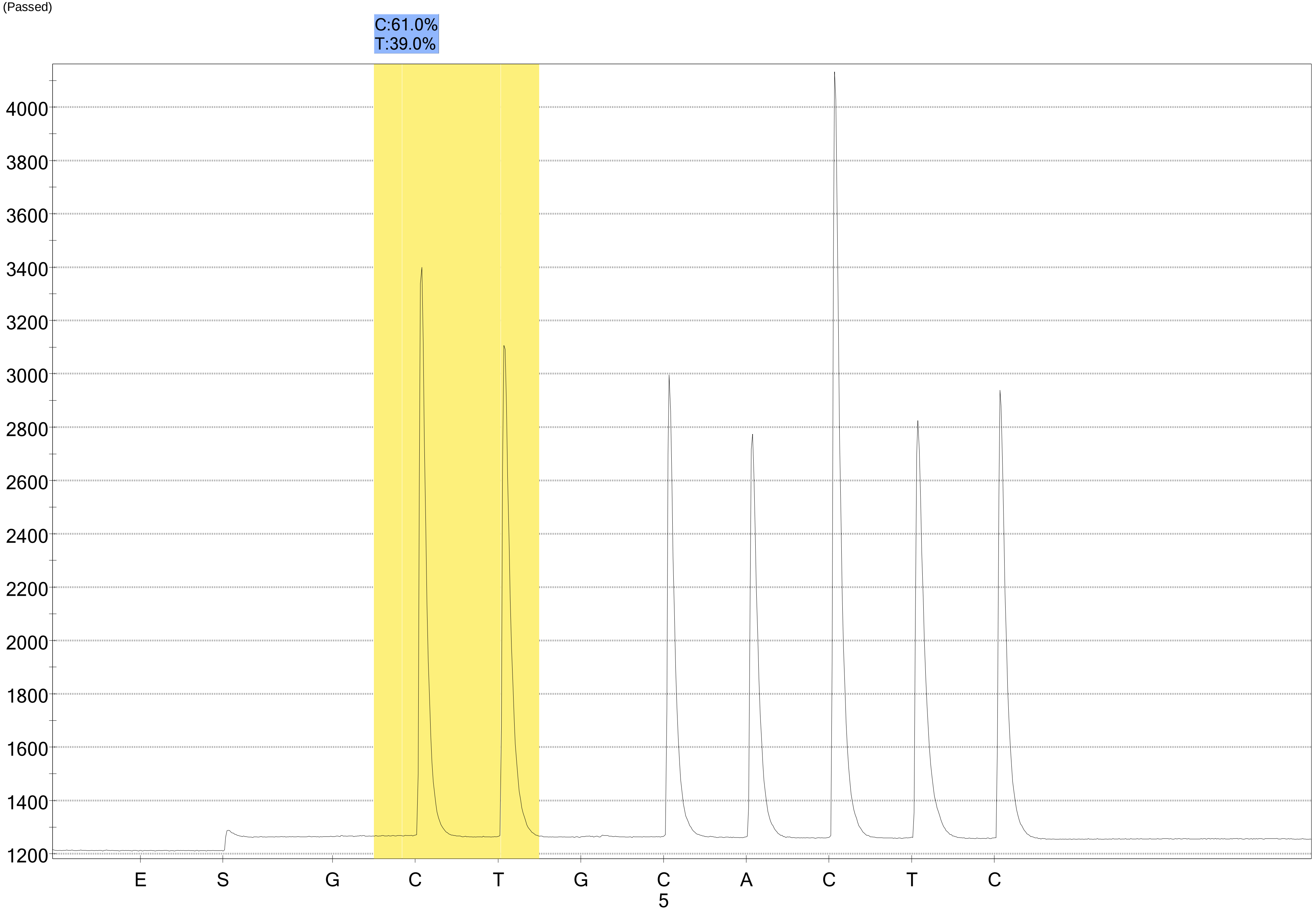

dna - Well G4
Entry: Mapt
2: C: 55.9% / T: 44.1%
(Passed)

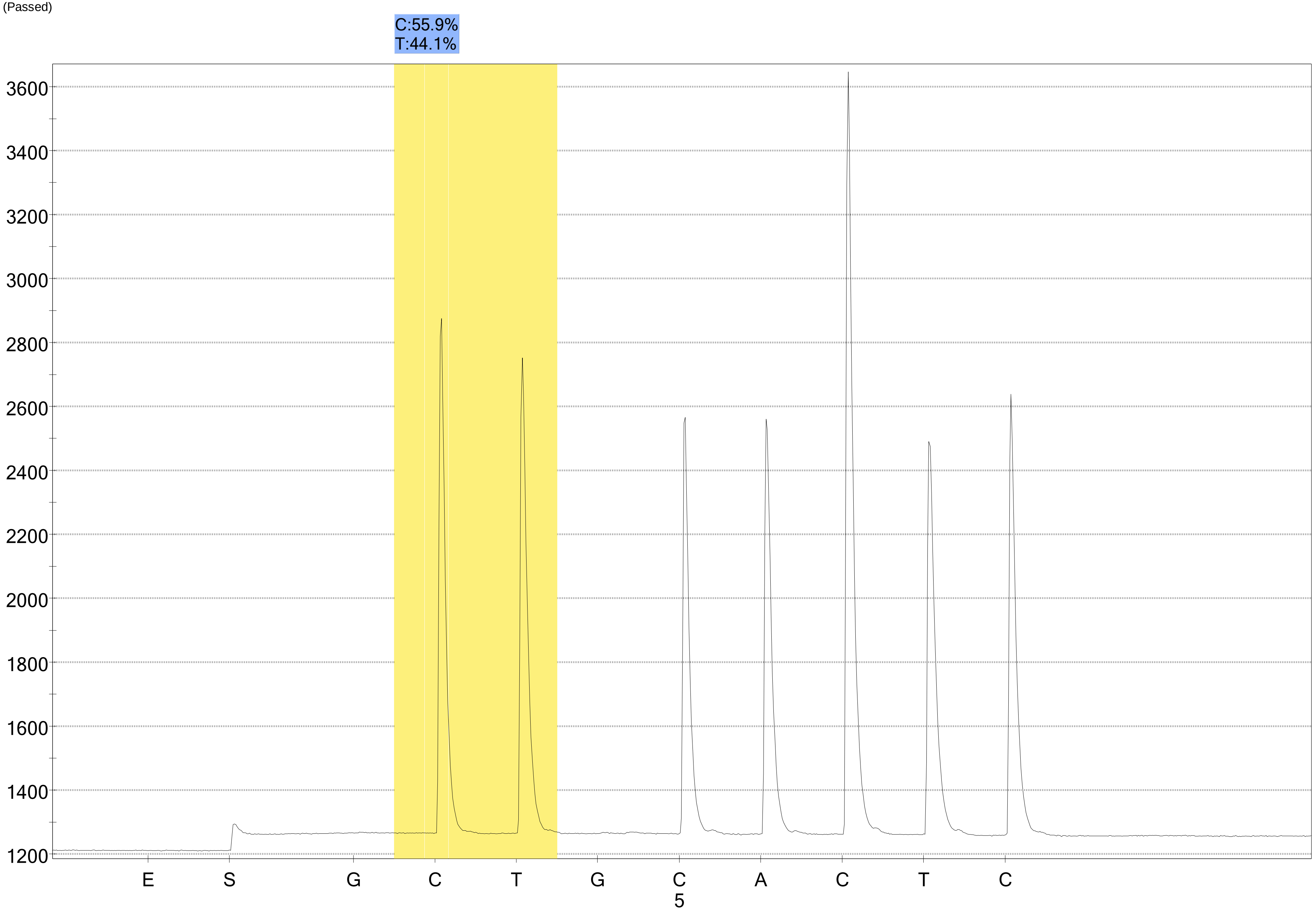

dna - Well G10
Entry: Mapt
2: C: 57.0% / T: 43.0%
(Passed)

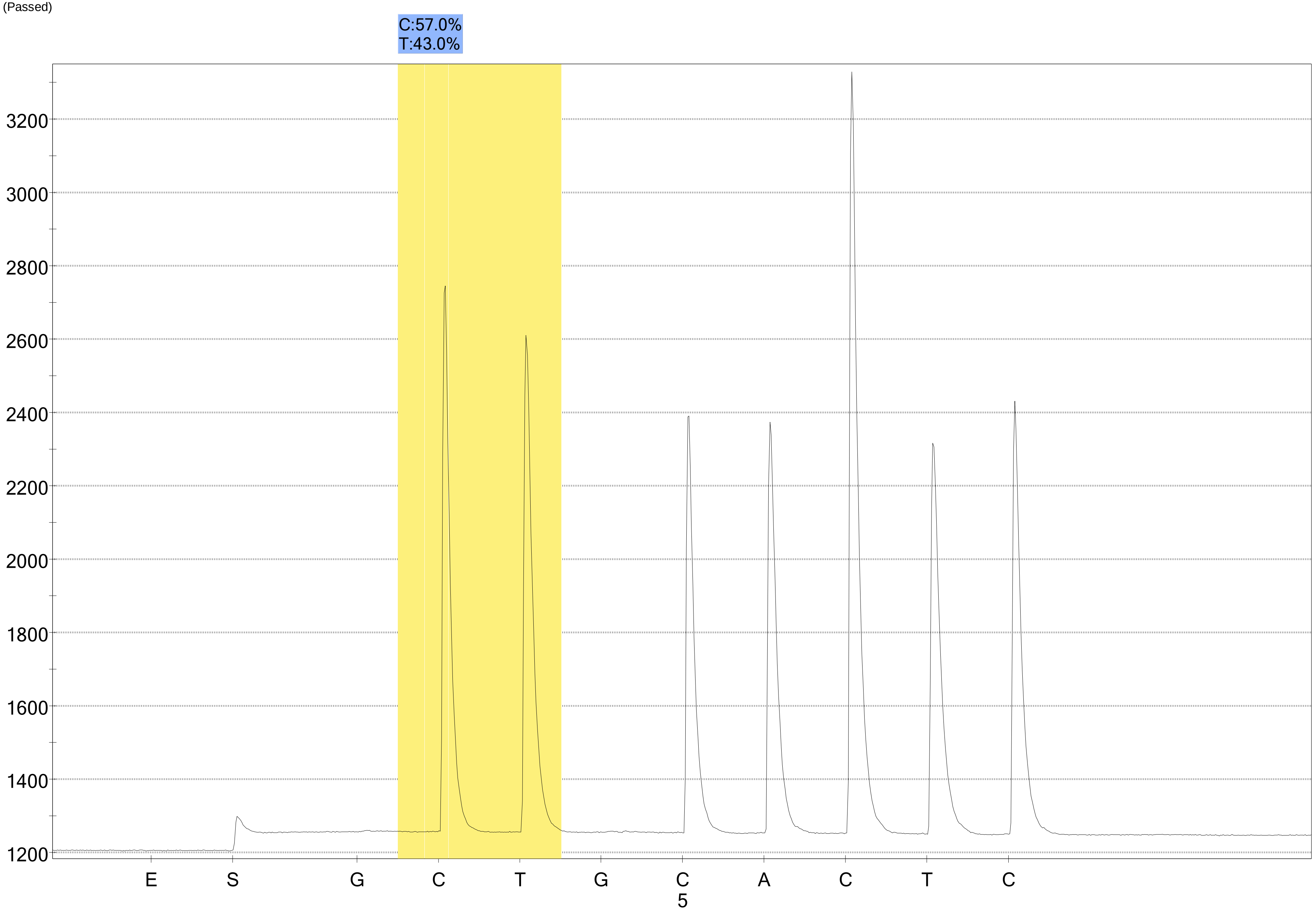

10 uL universal (141+157) - Well H3
Entry: Herc3
1: G: 41.8% / T: 58.2%
(Passed)

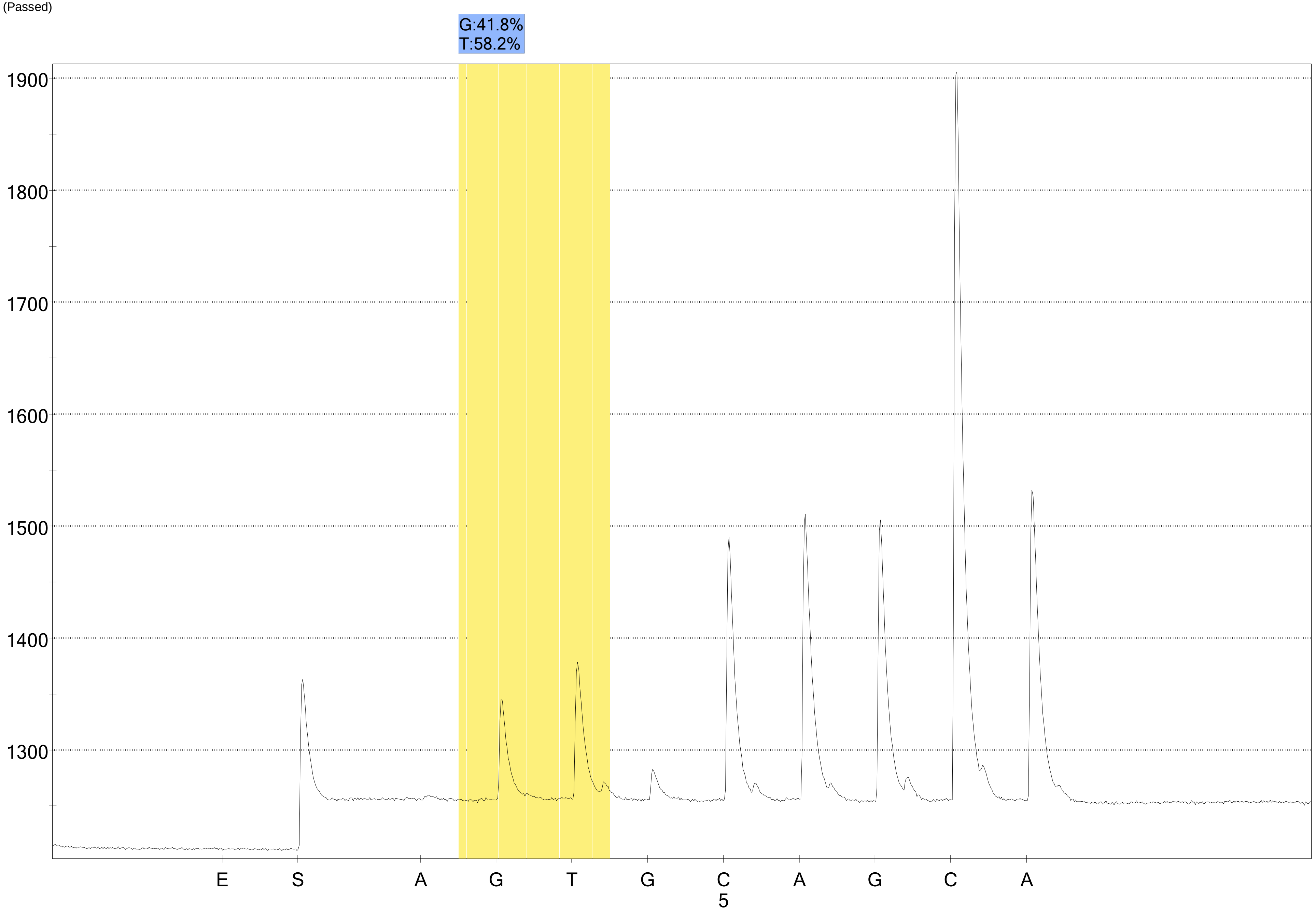

10 uL universal (141+157) - Well H9
Entry: Herc3
1: G: 23.4% / T: 76.6%
(Passed)

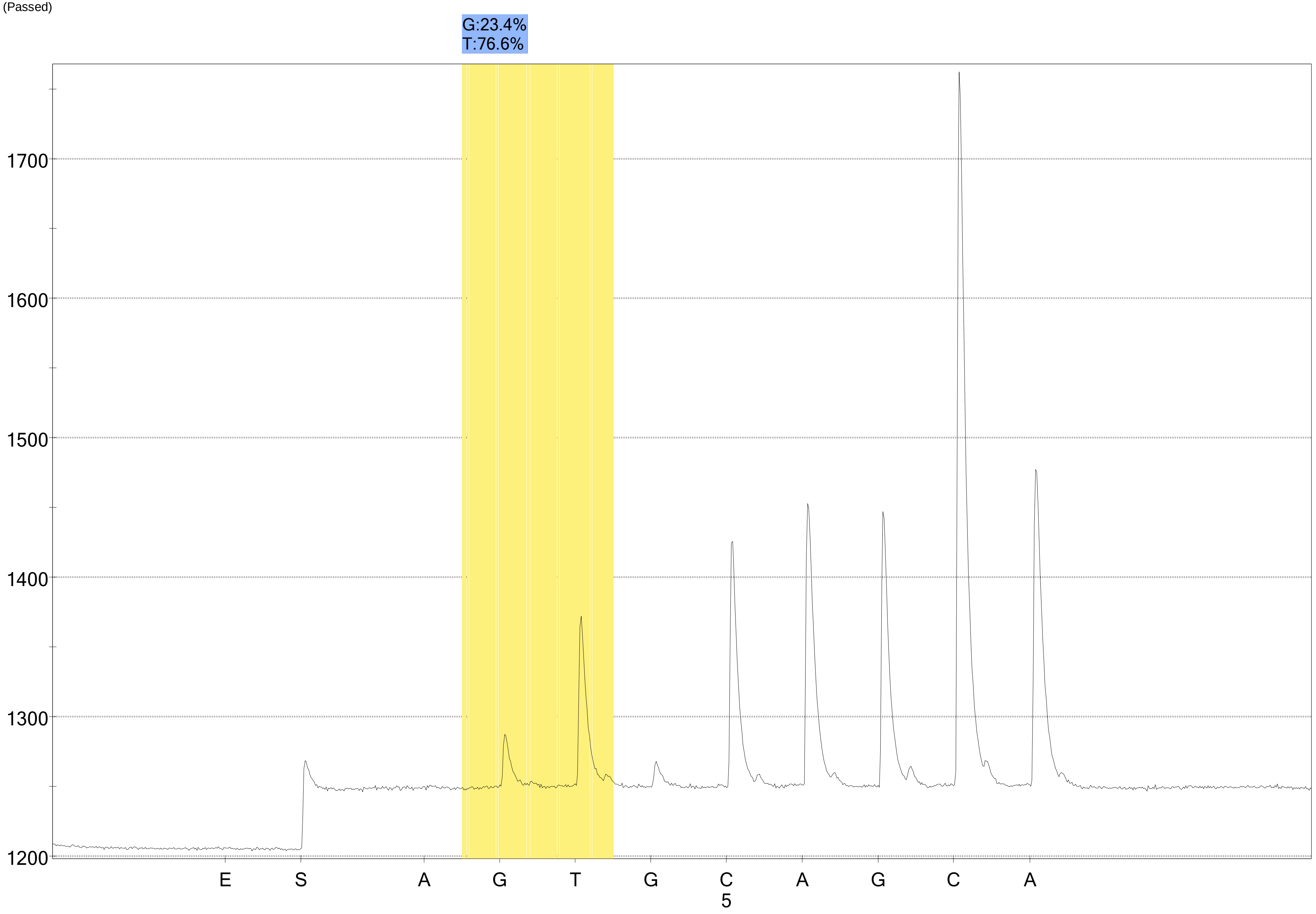

145 - Well H3
Entry: Herc3
1: G: 42.1% / T: 57.9%
(Passed)

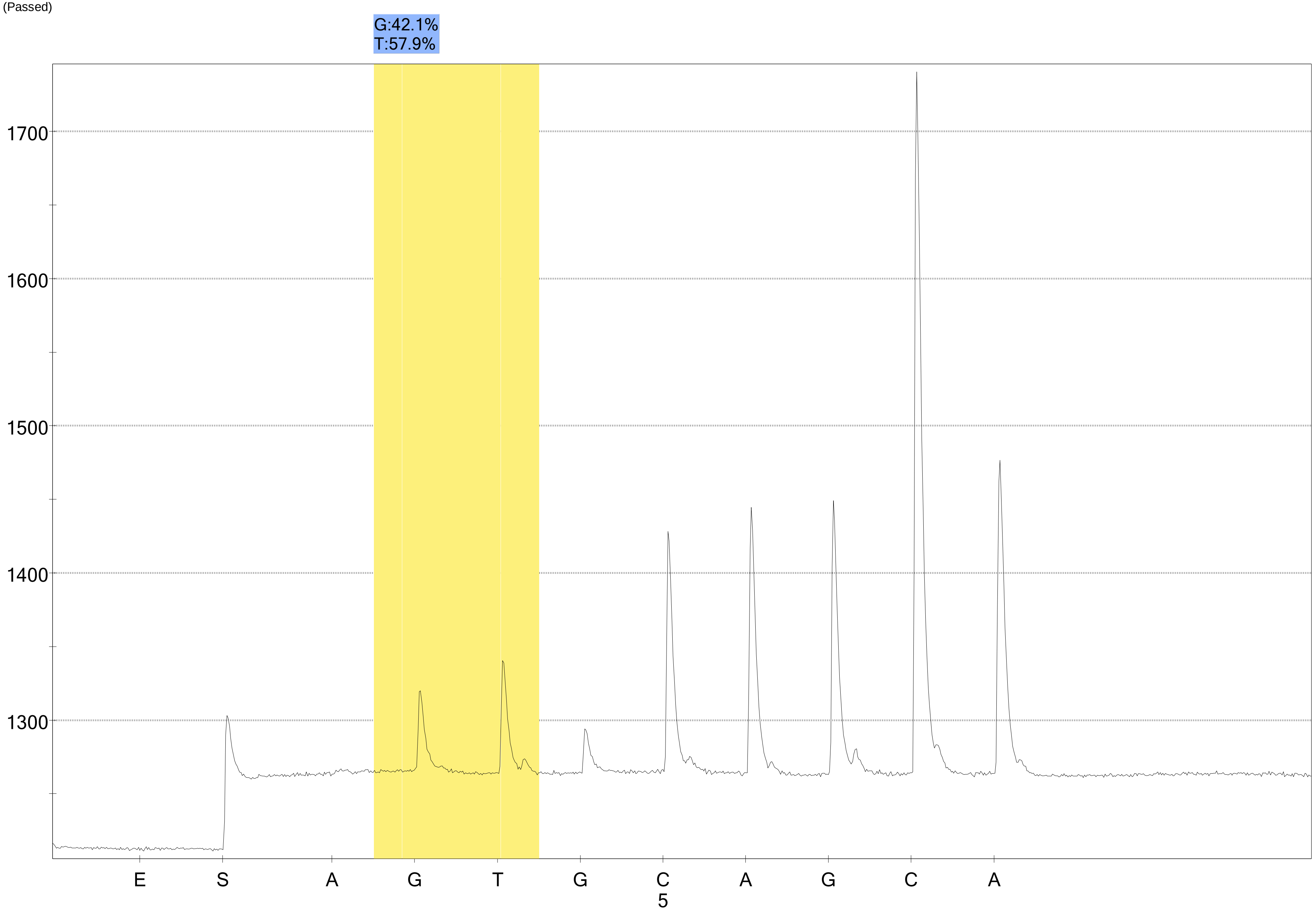

dna - Well H3
Entry: Herc3
1: G: 78.5% / T: 21.5%
(Passed)

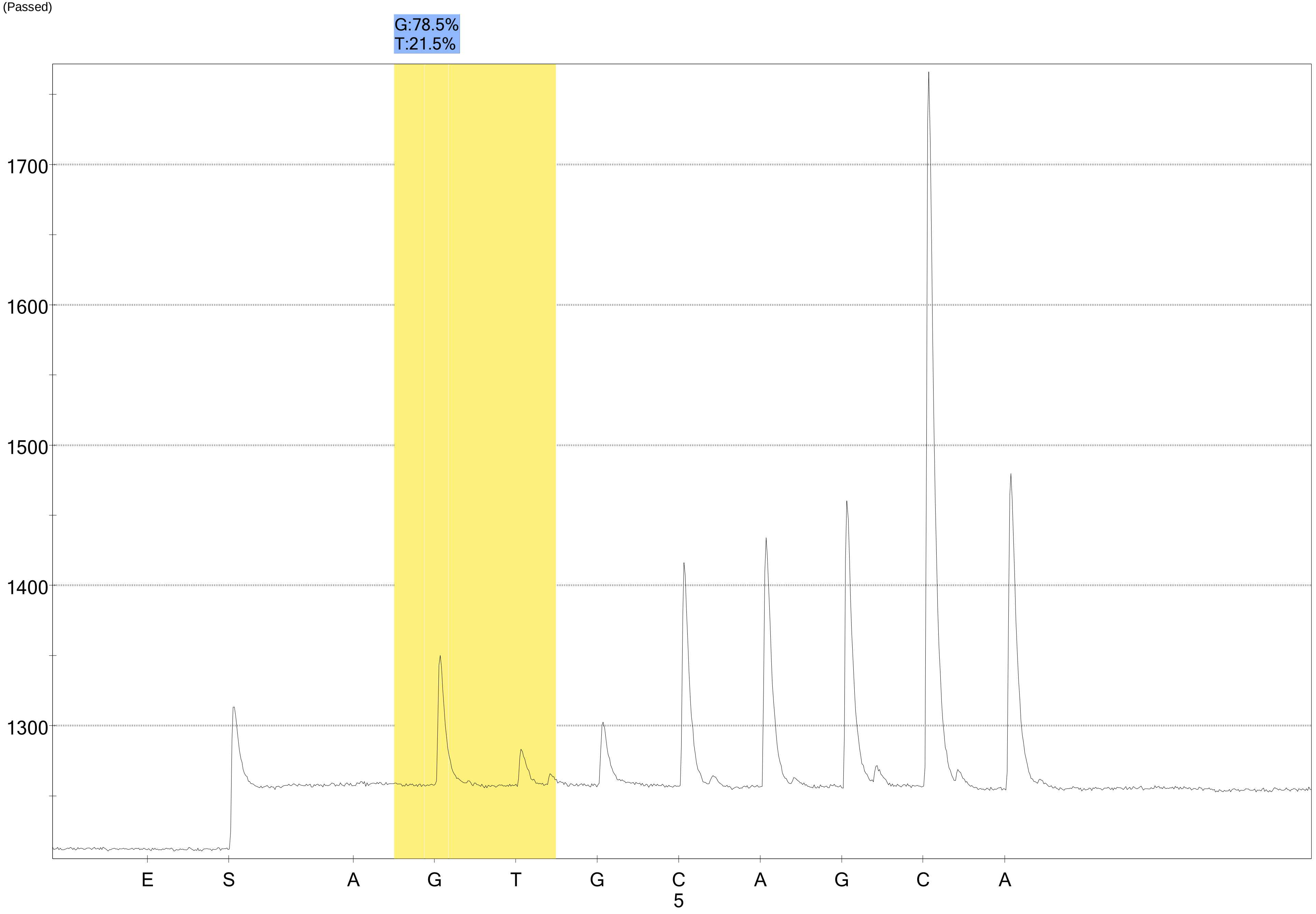

dna - Well H9
Entry: Herc3
1: G: 76.1% / T: 23.9%
(Passed)

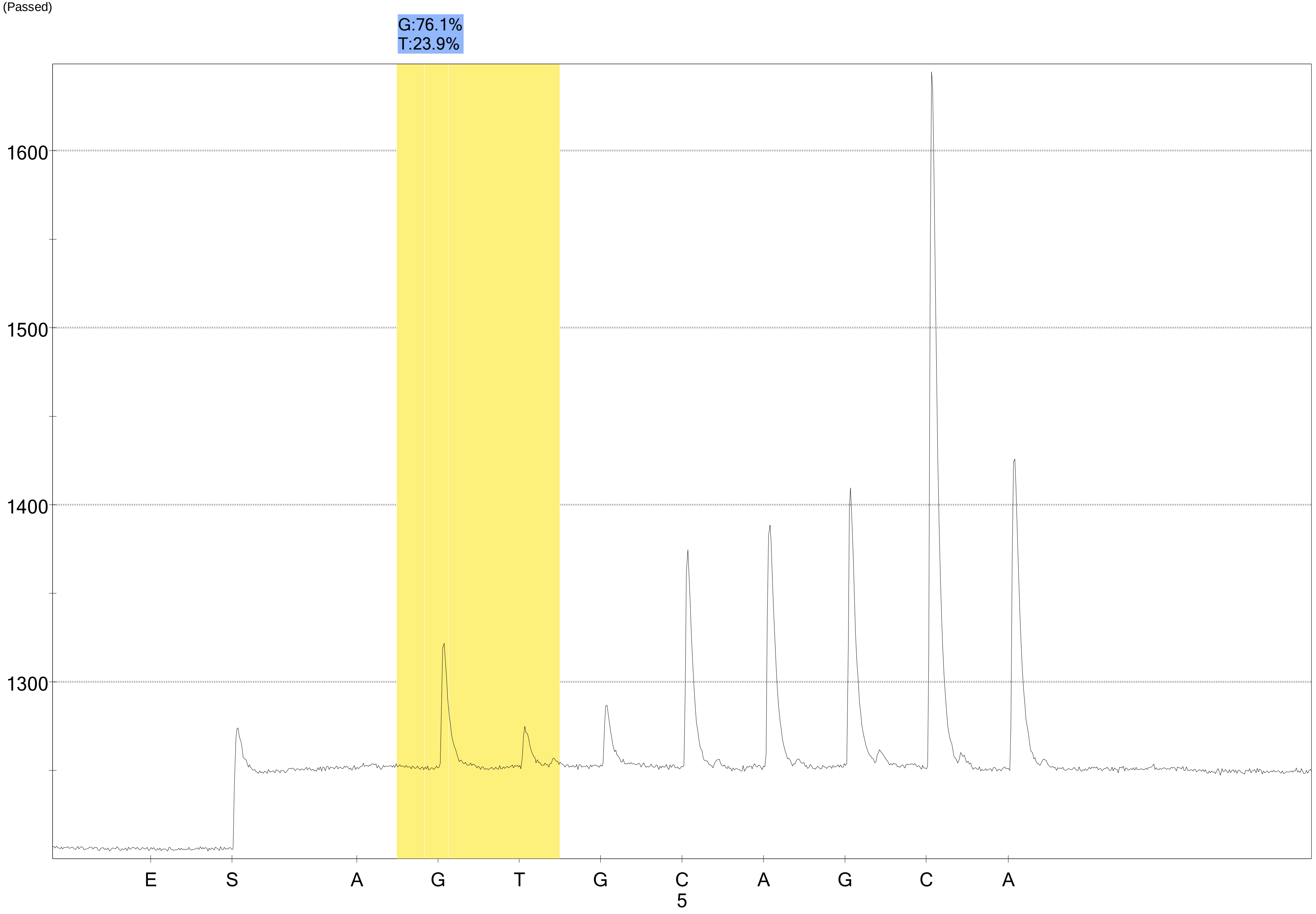

10 uL universal (141+157) - Well G1
Entry: Apba2
1: C: 21.1% / T: 78.9%
(Passed)

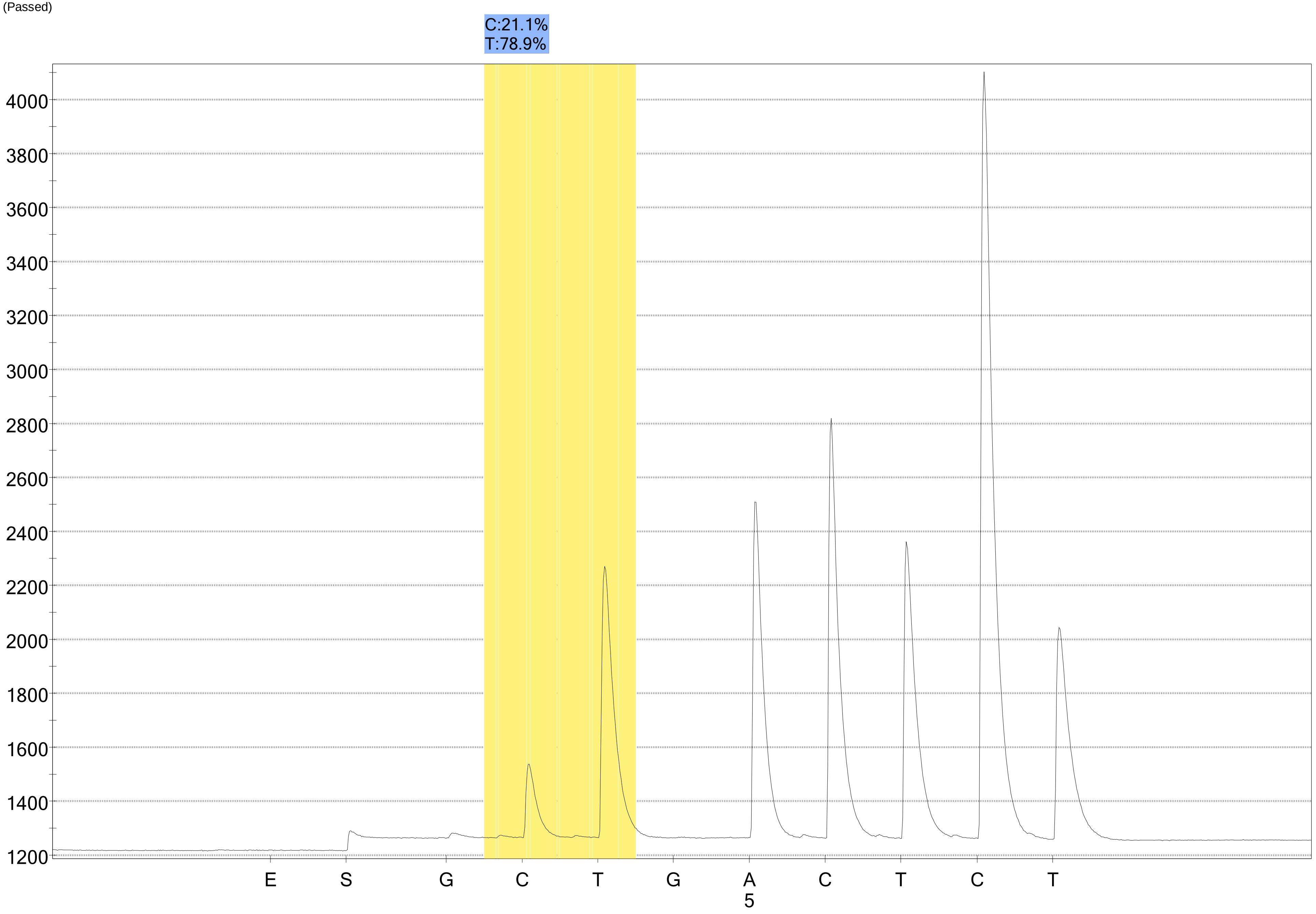

10 uL universal (141+157) - Well G7
Entry: Apba2
1: C: 26.5% / T: 73.5%
(Passed)

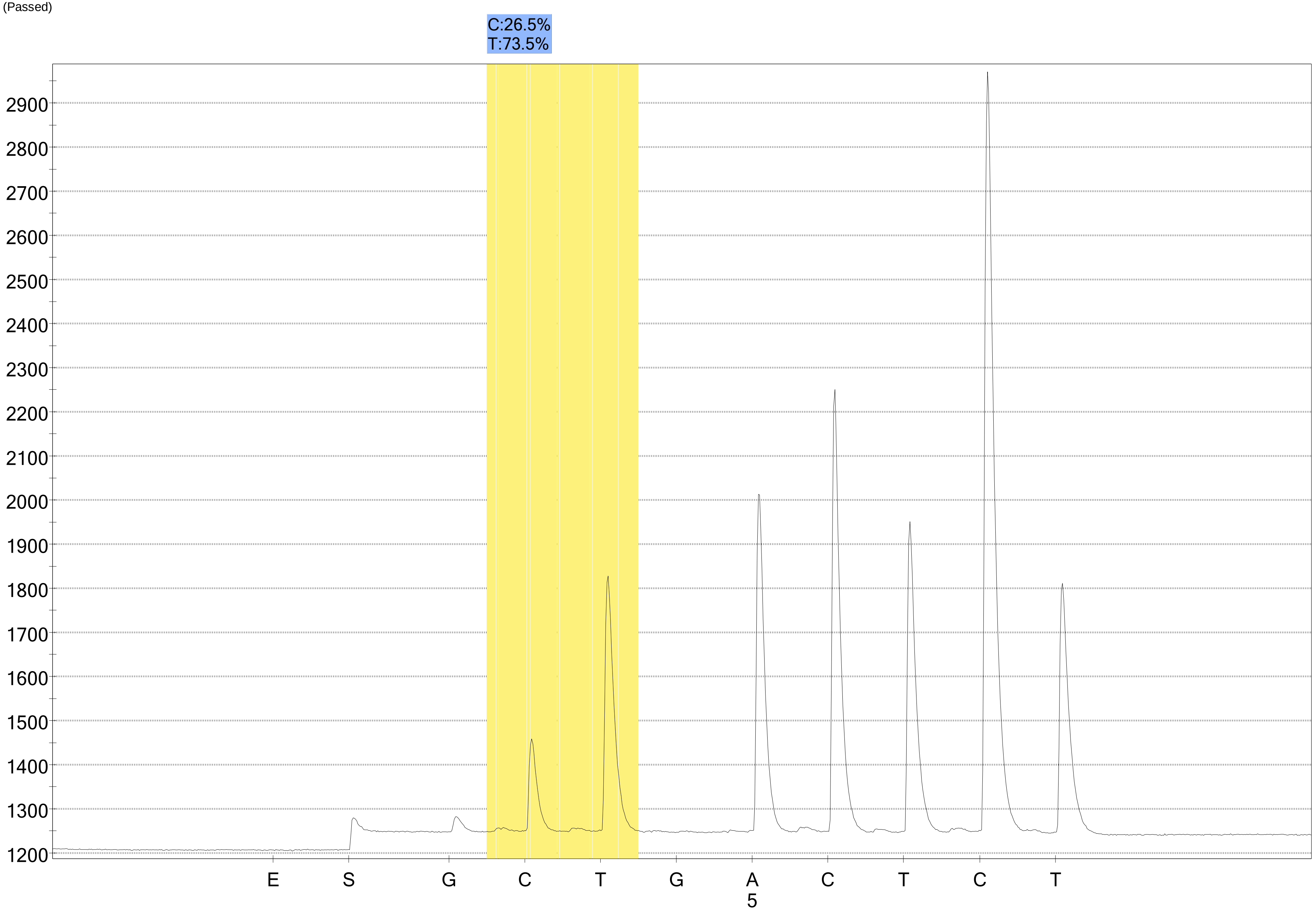

145 - Well G1
Entry: Apba2
1: C: 23.6% / T: 76.4%
(Passed)

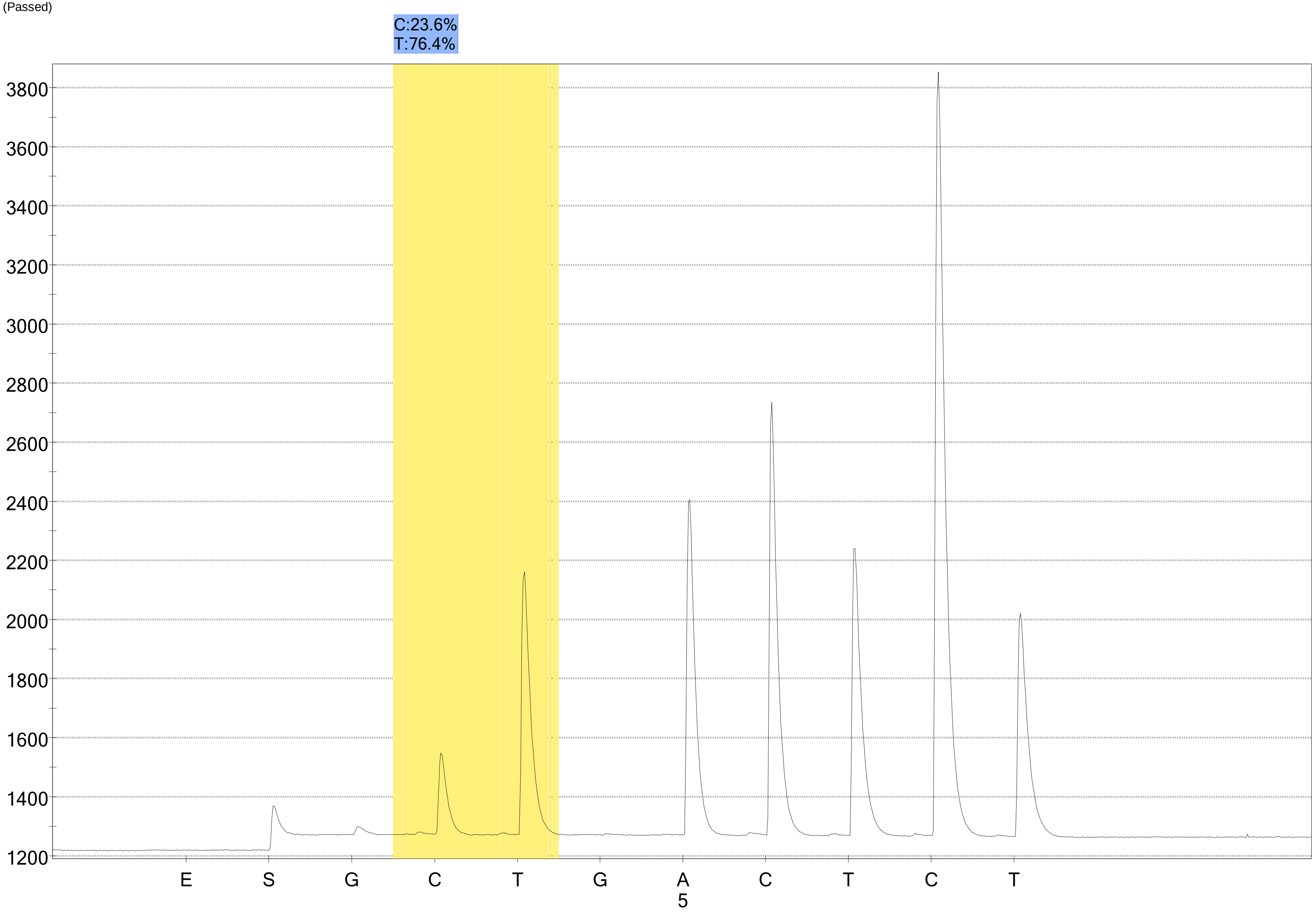

dna - Well G1
Entry: Apba2
1: C: 21.4% / T: 78.6%
(Passed)

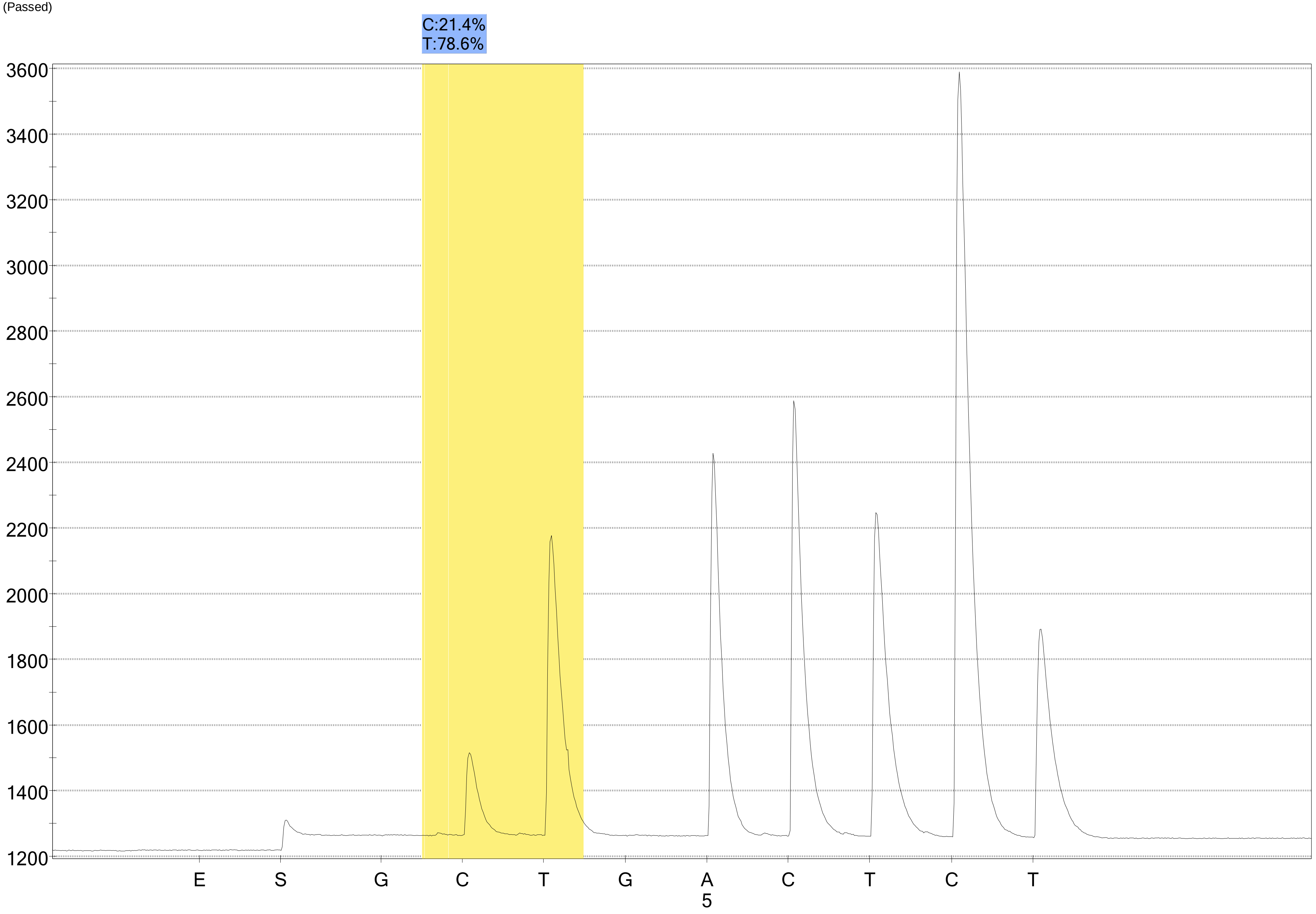

dna - Well G7
Entry: Apba2
1: C: 17.6% / T: 82.4%
(Passed)

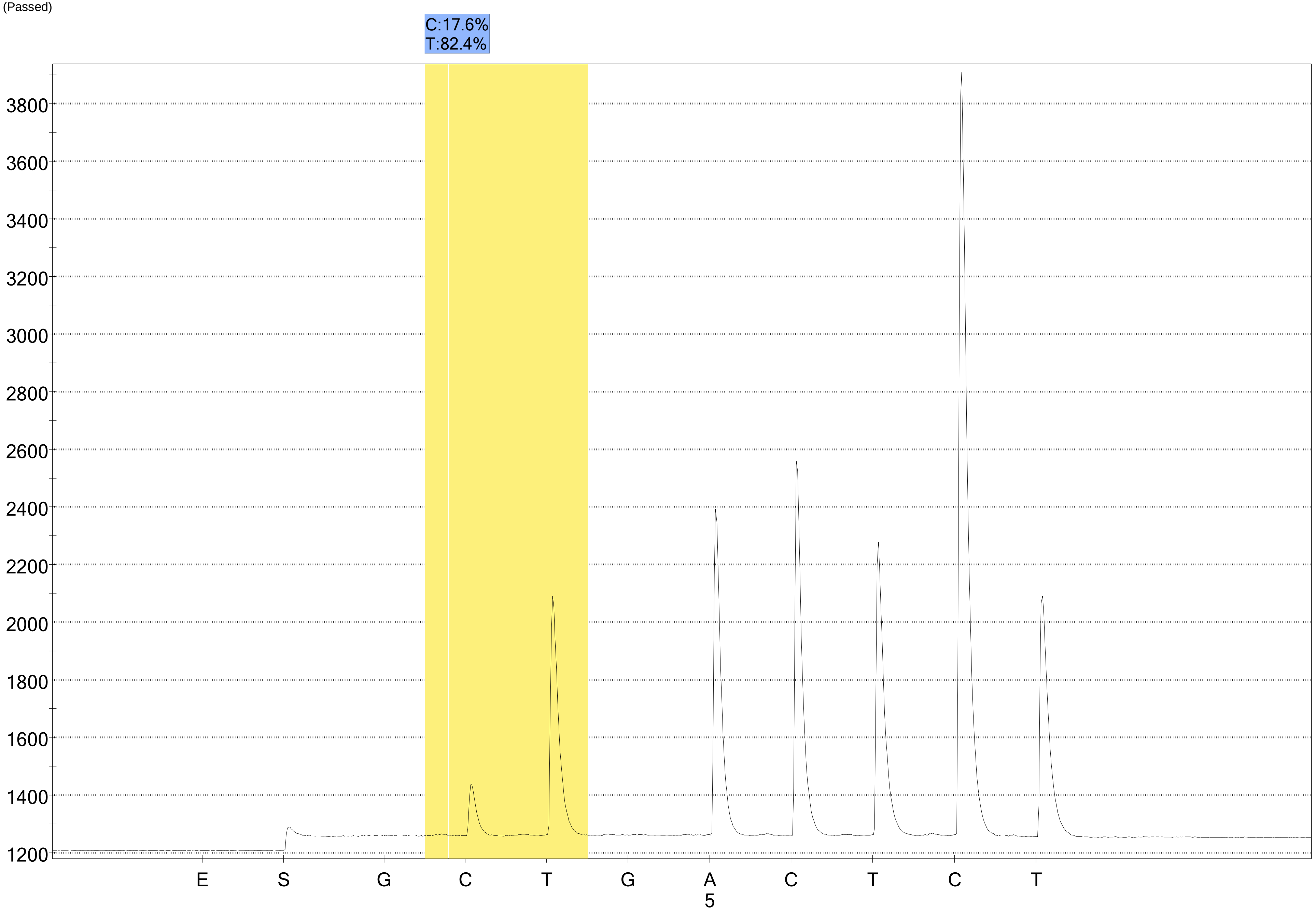

10 uL universal (141+157) - Well C1
Entry: Adam23
2: C: 39.7% / T: 60.3%
(Passed)

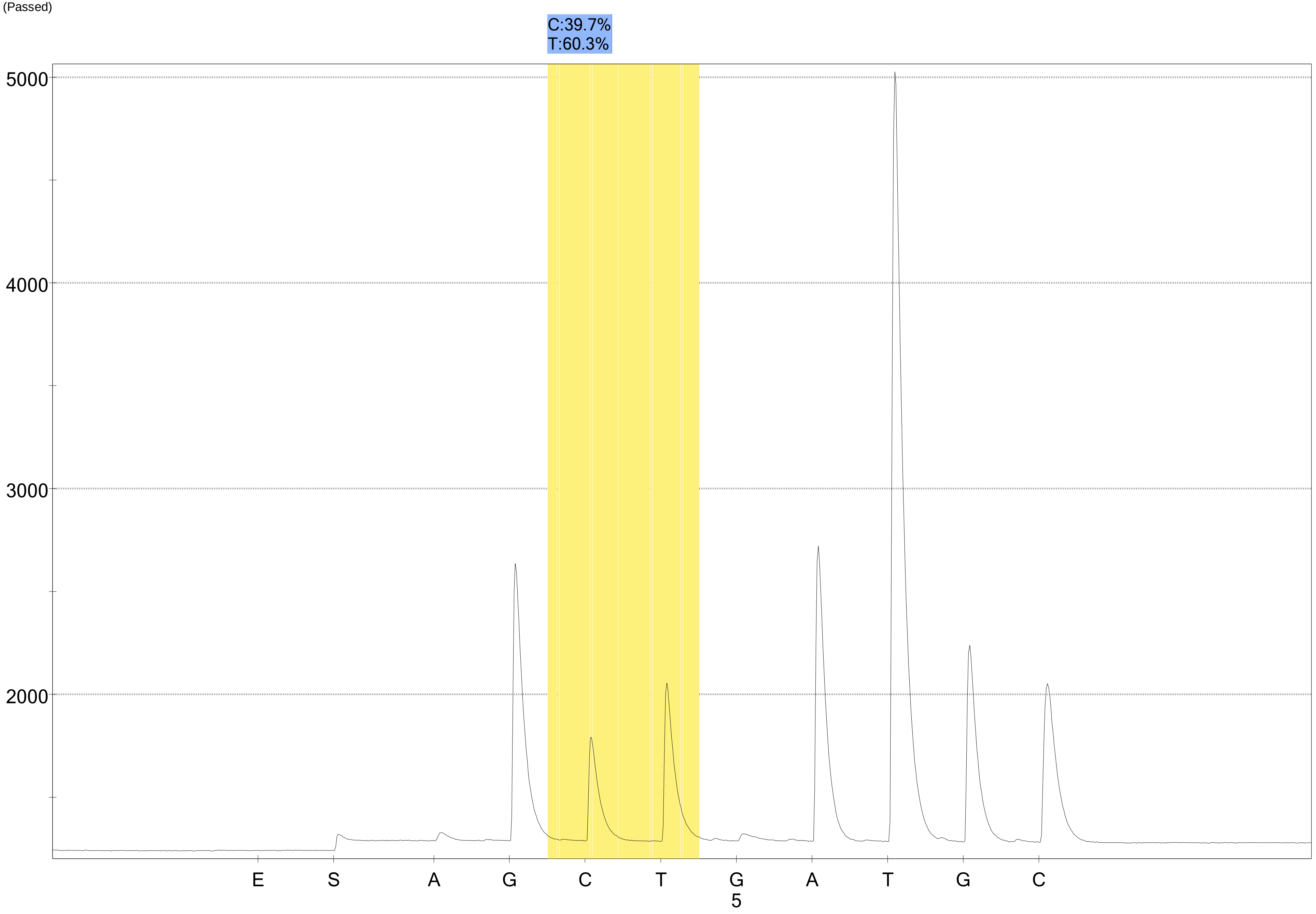

10 uL universal (141+157) - Well C7
Entry: Adam23
2: C: 54.9% / T: 45.1%
(Passed)

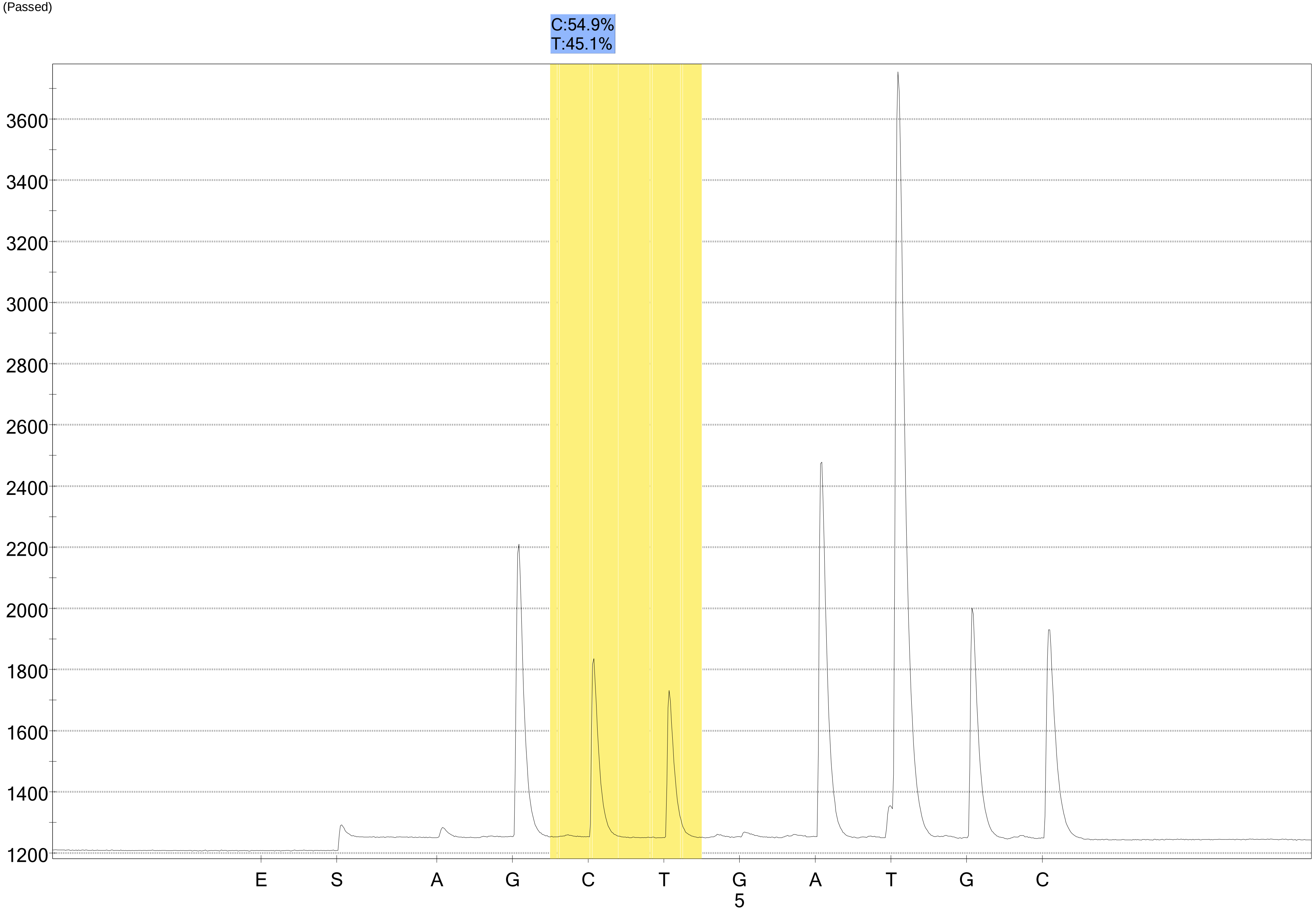

145 - Well C1
Entry: Adam23
2: C: 35.8% / T: 64.2%
(Passed)

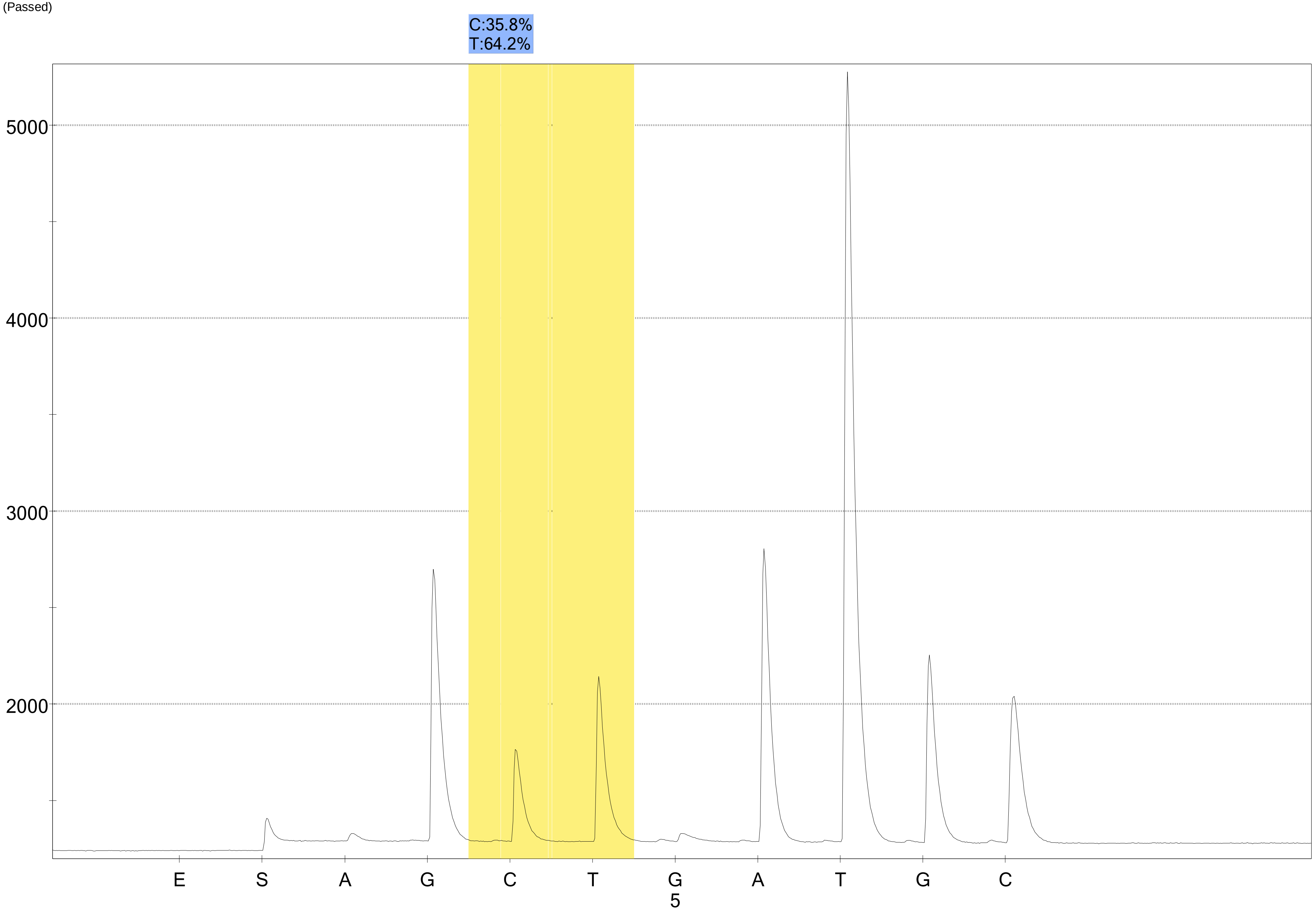

dna - Well C1
Entry: Adam23
2: C: 56.6% / T: 43.4%
(Passed)

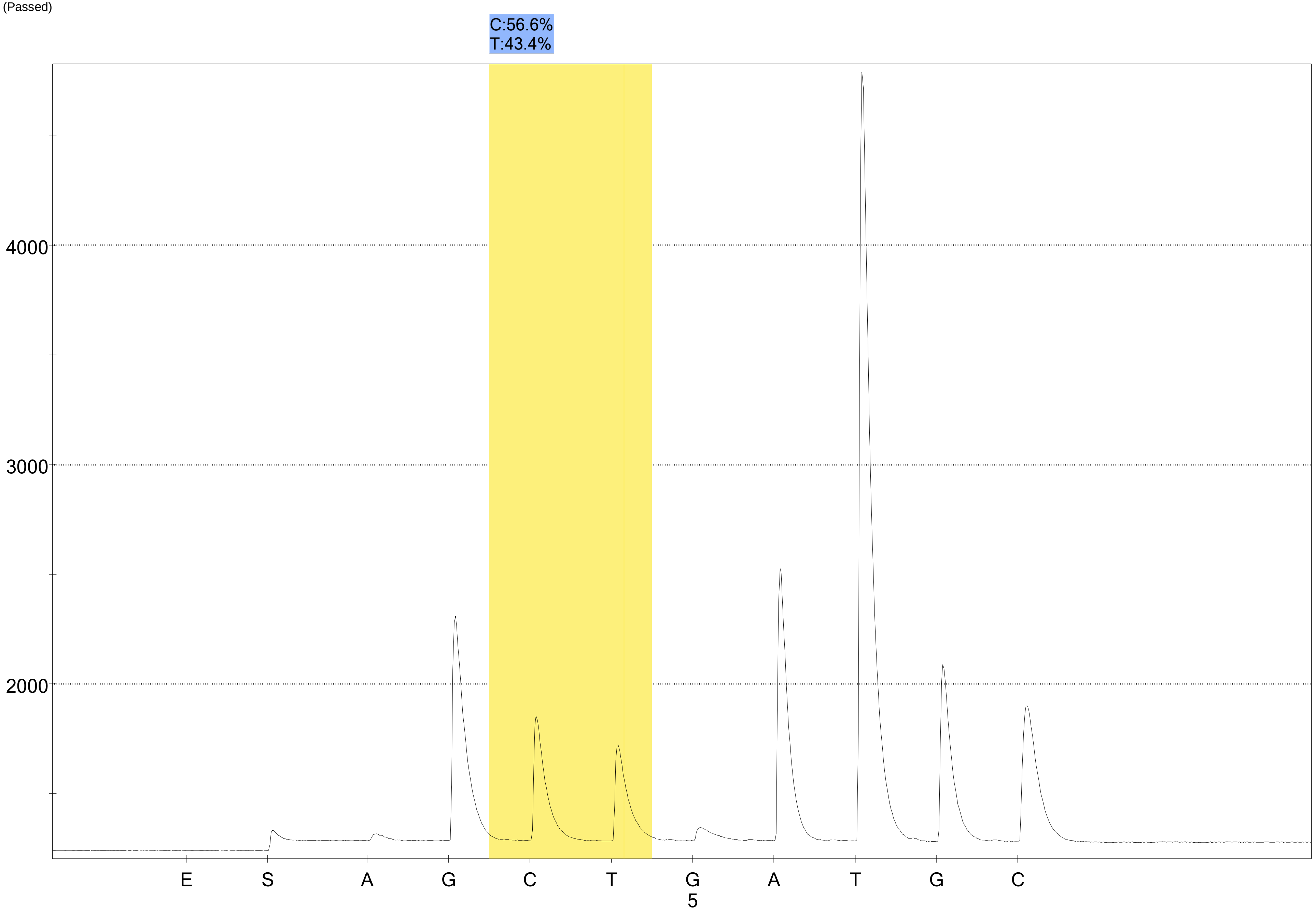

dna - Well C7
Entry: Adam23
2: C: 50.1% / T: 49.9%
(Passed)

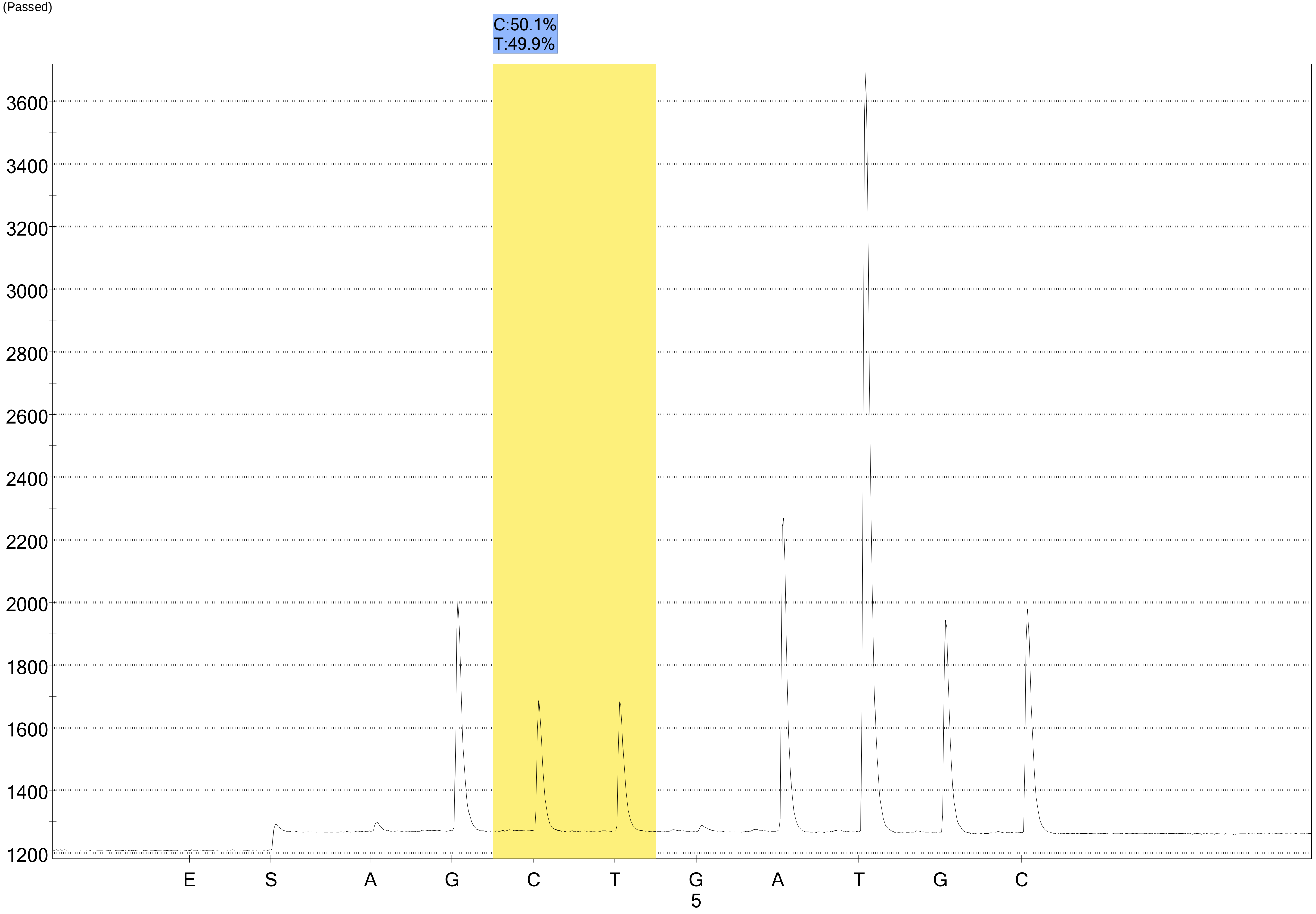

10 uL universal (141+157) - Well G6
Entry: Wars
4: A: 53.6% / G: 46.4%
(Passed)

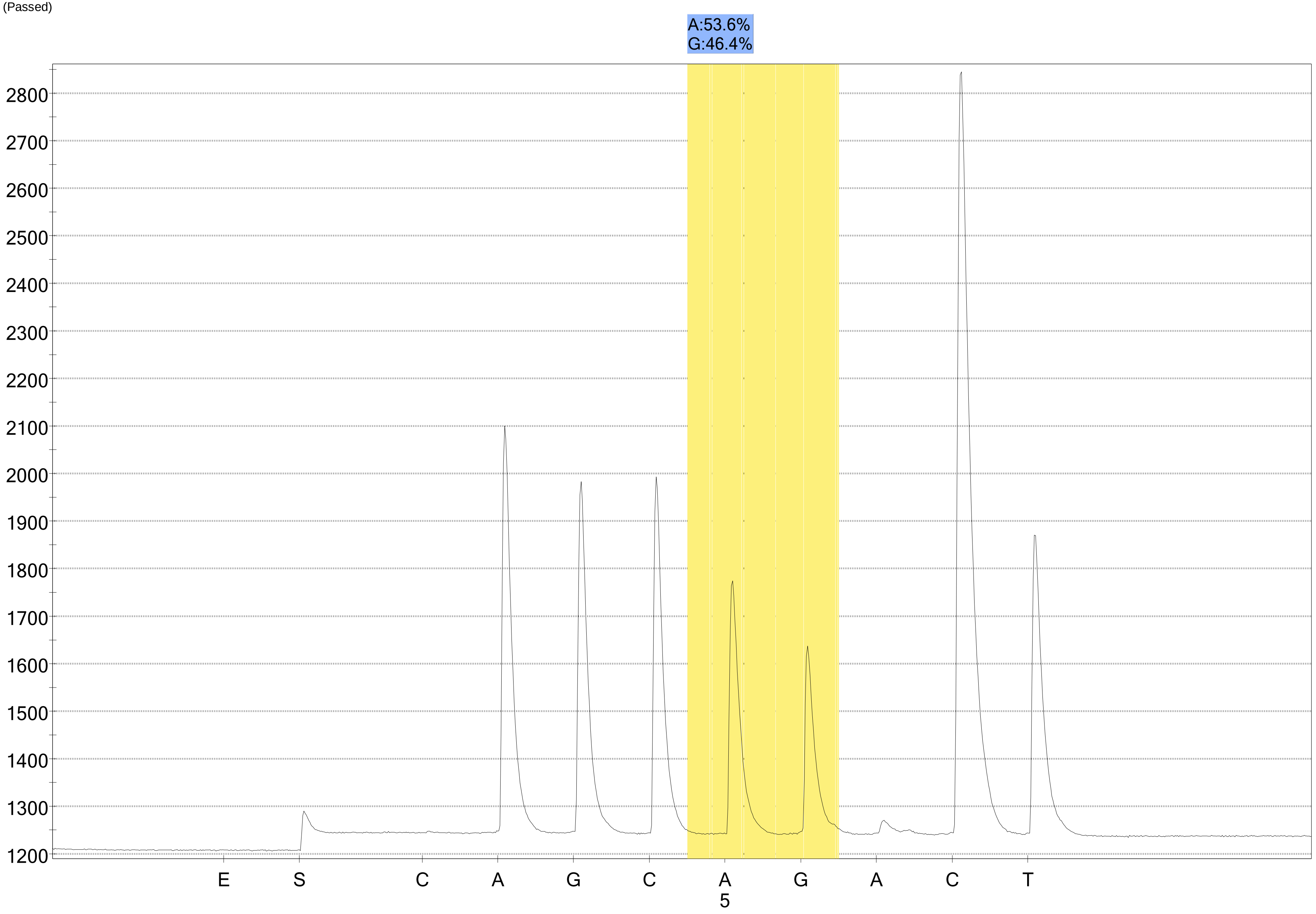

10 uL universal (141+157) - Well G12
Entry: Wars
4: A: 45.8% / G: 54.2%
(Passed)

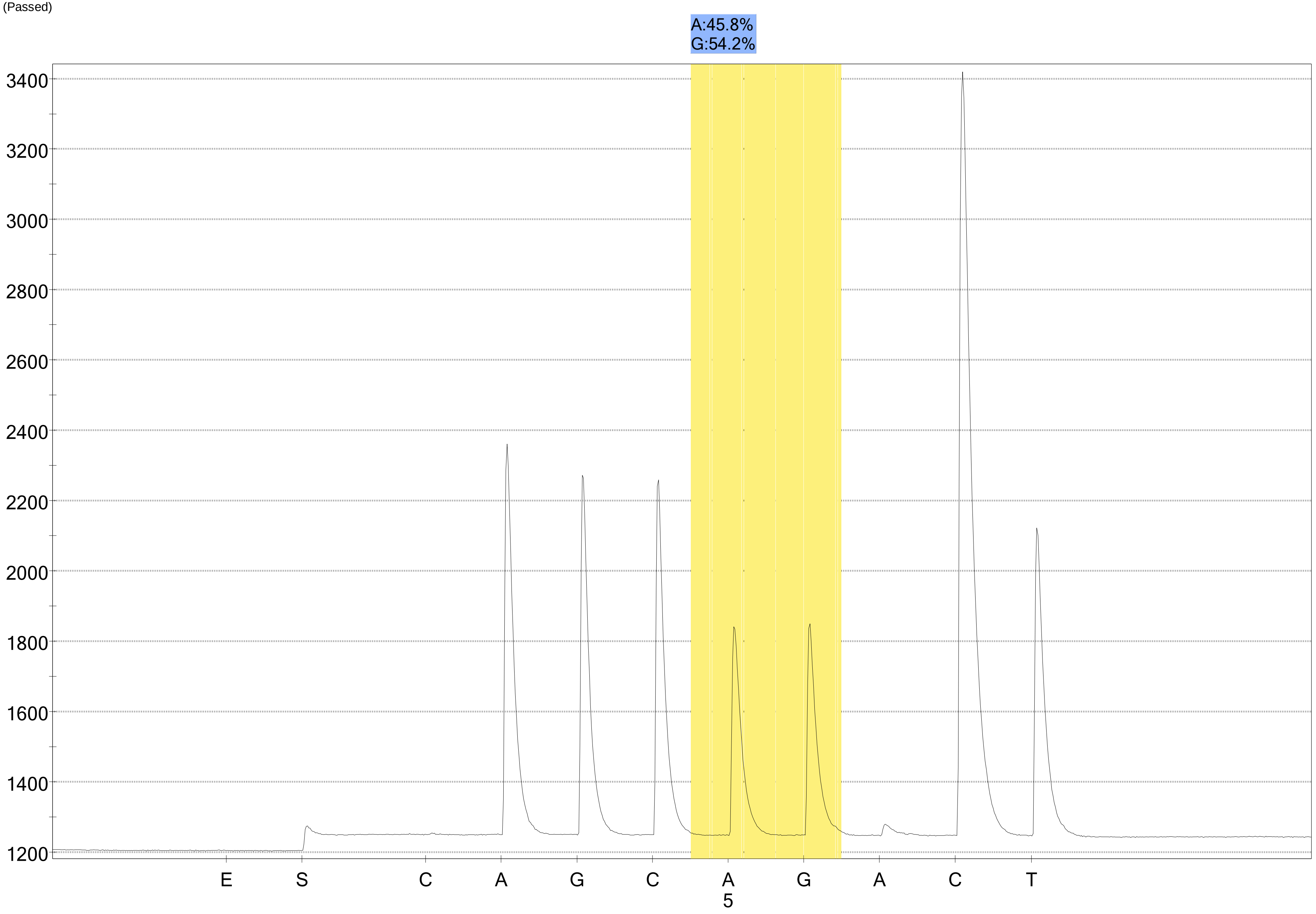

145 - Well G6
Entry: Wars
4: A: 54.8% / G: 45.2%
(Passed)

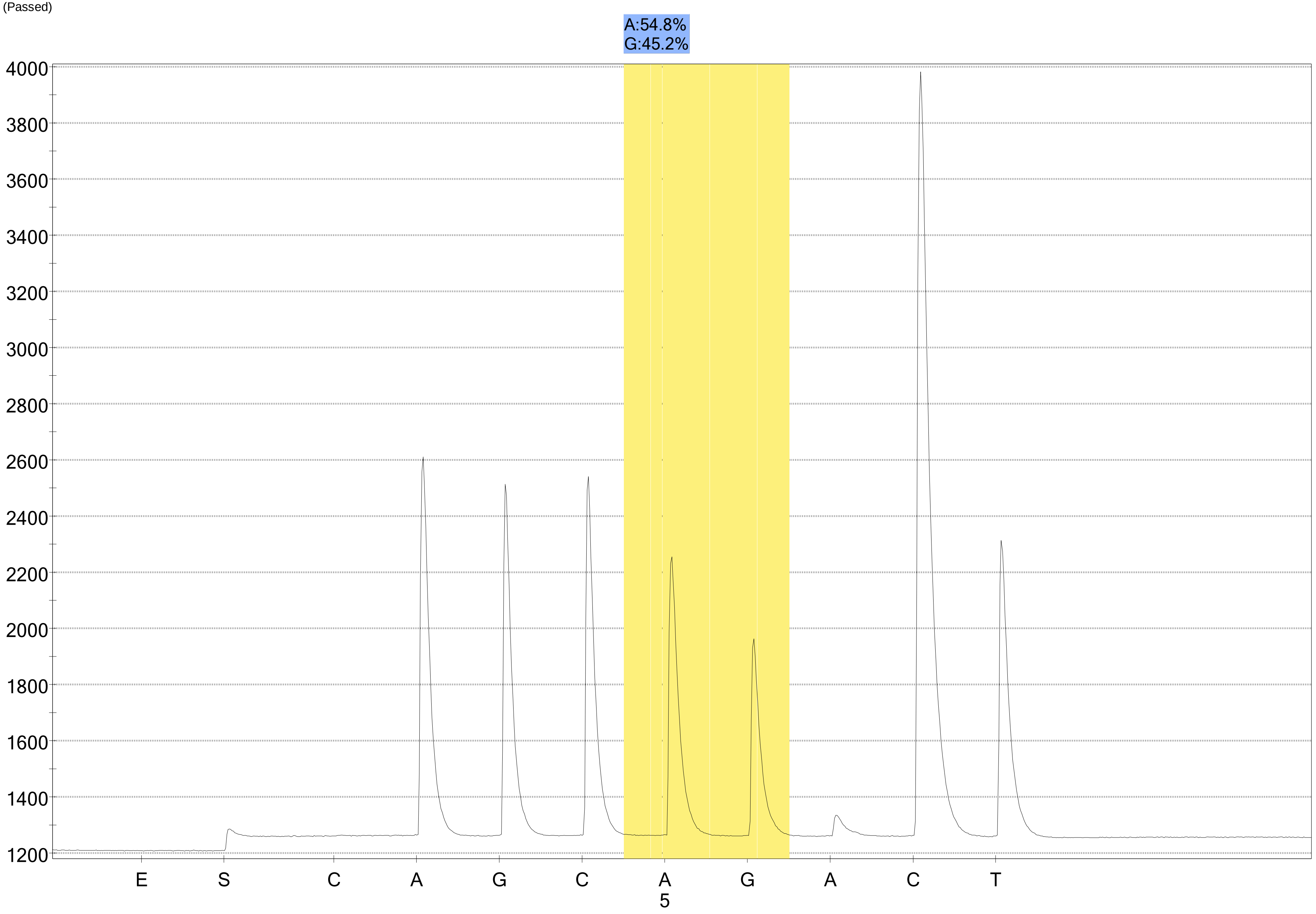

dna - Well G6
Entry: Wars
4: A: 51.6% / G: 48.4%
(Passed)

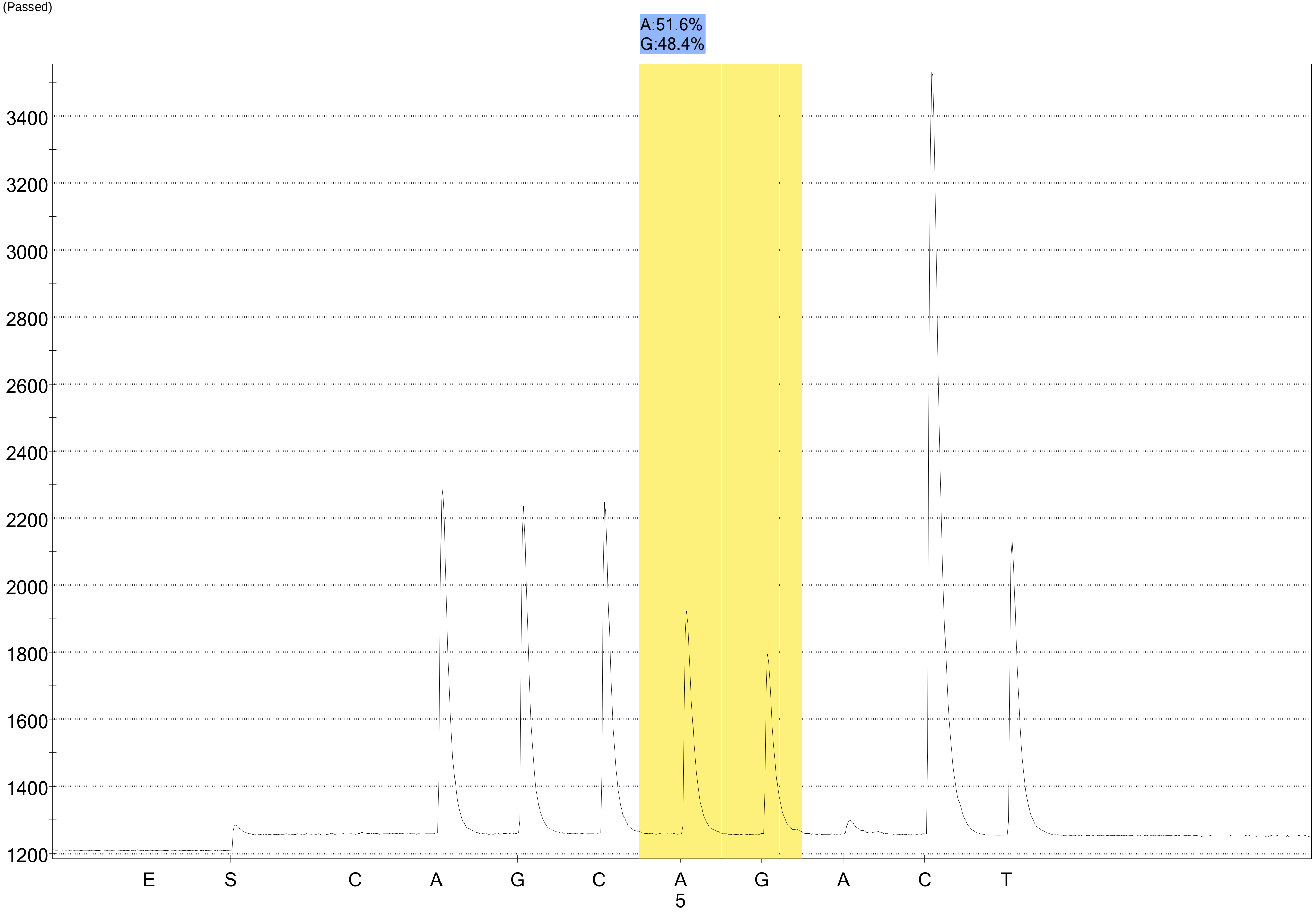

dna - Well G12
Entry: Wars
4: A: 51.0% / G: 49.0%
(Passed)

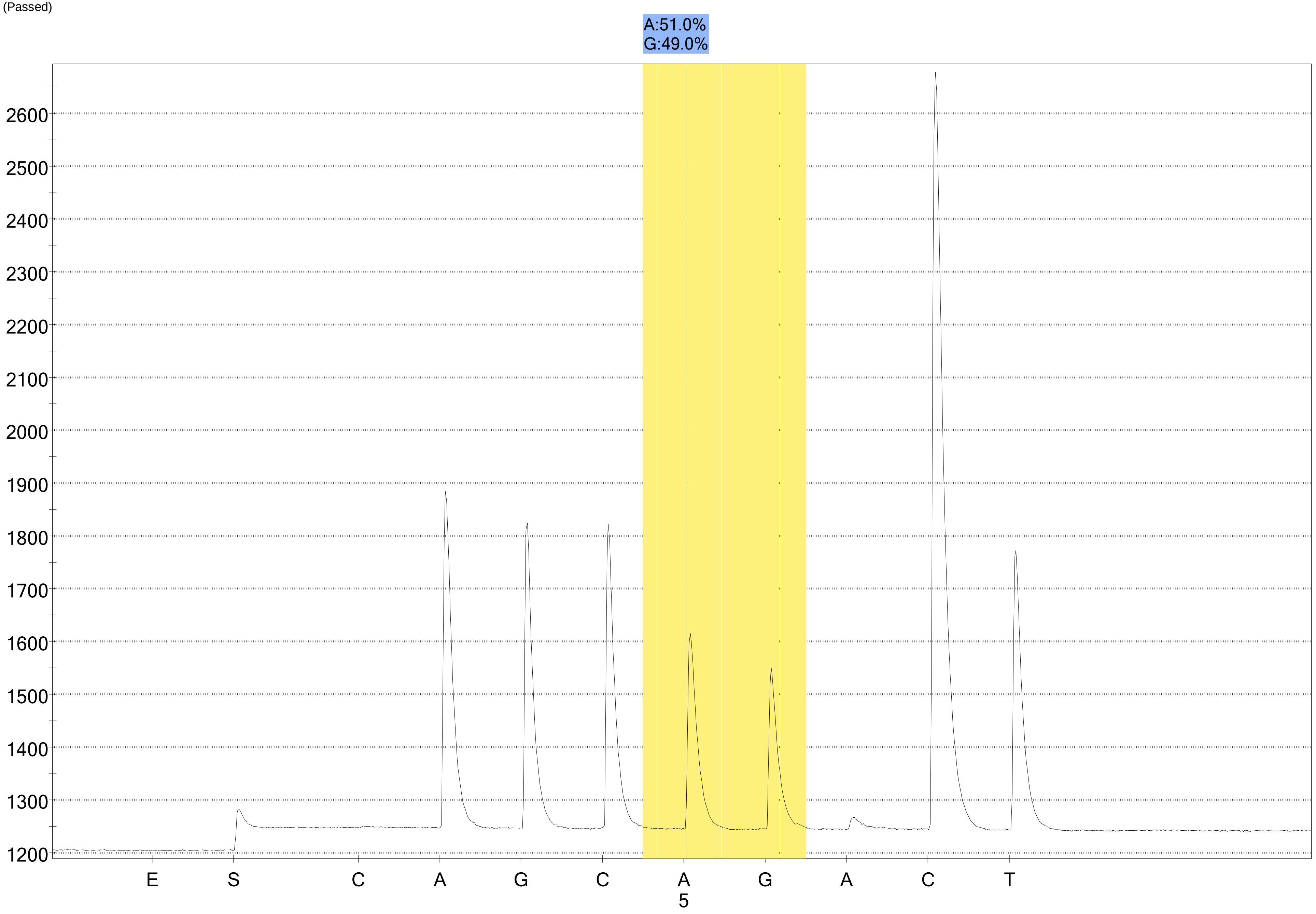

10 uL universal (141+157) - Well B1
Entry: 5230400G24Rik
1: C: 87.3% / T: 12.7%
(Passed)

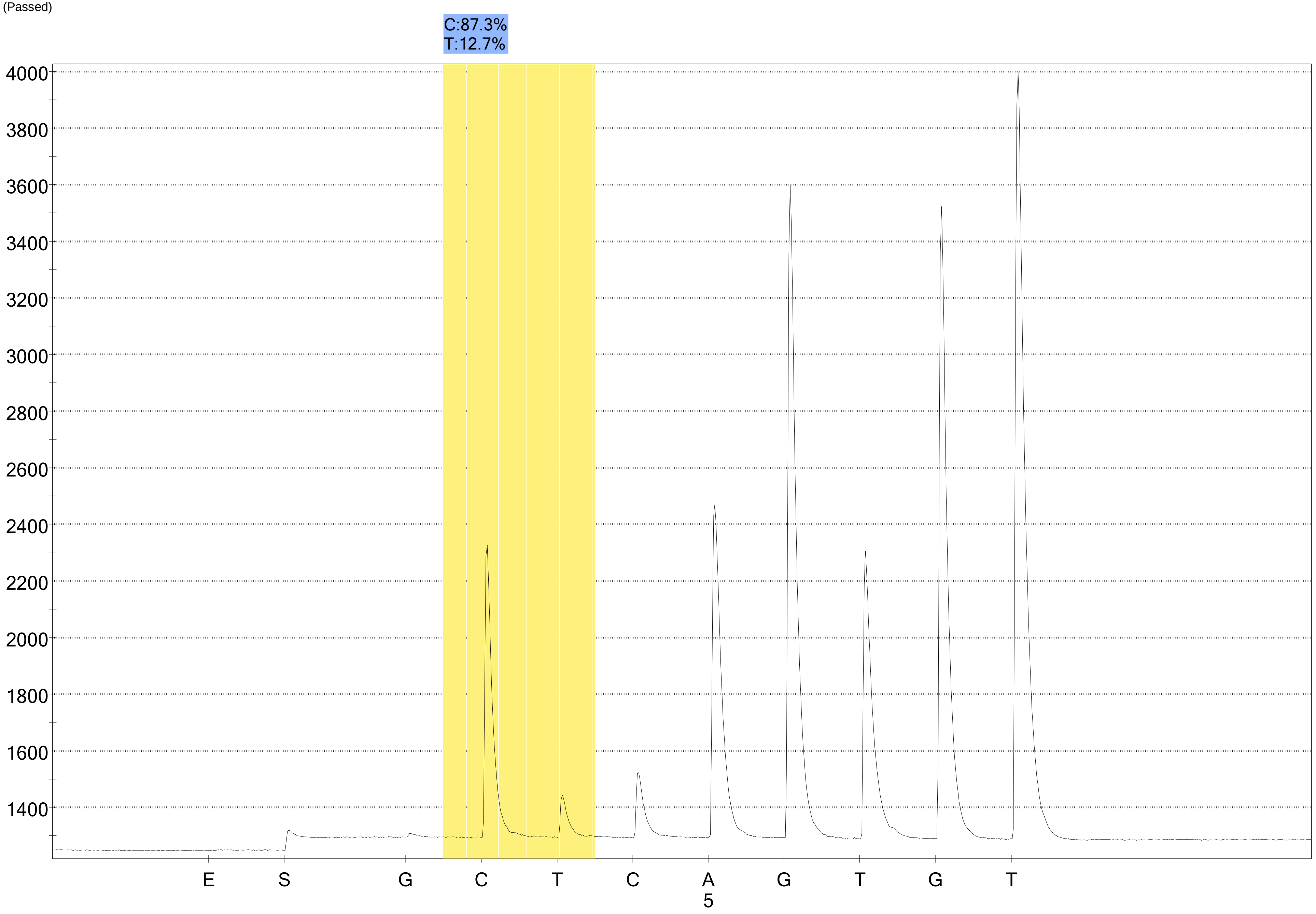

10 uL universal (141+157) - Well B7
Entry: 5230400G24Rik
1: C: 86.9% / T: 13.1%
(Passed)

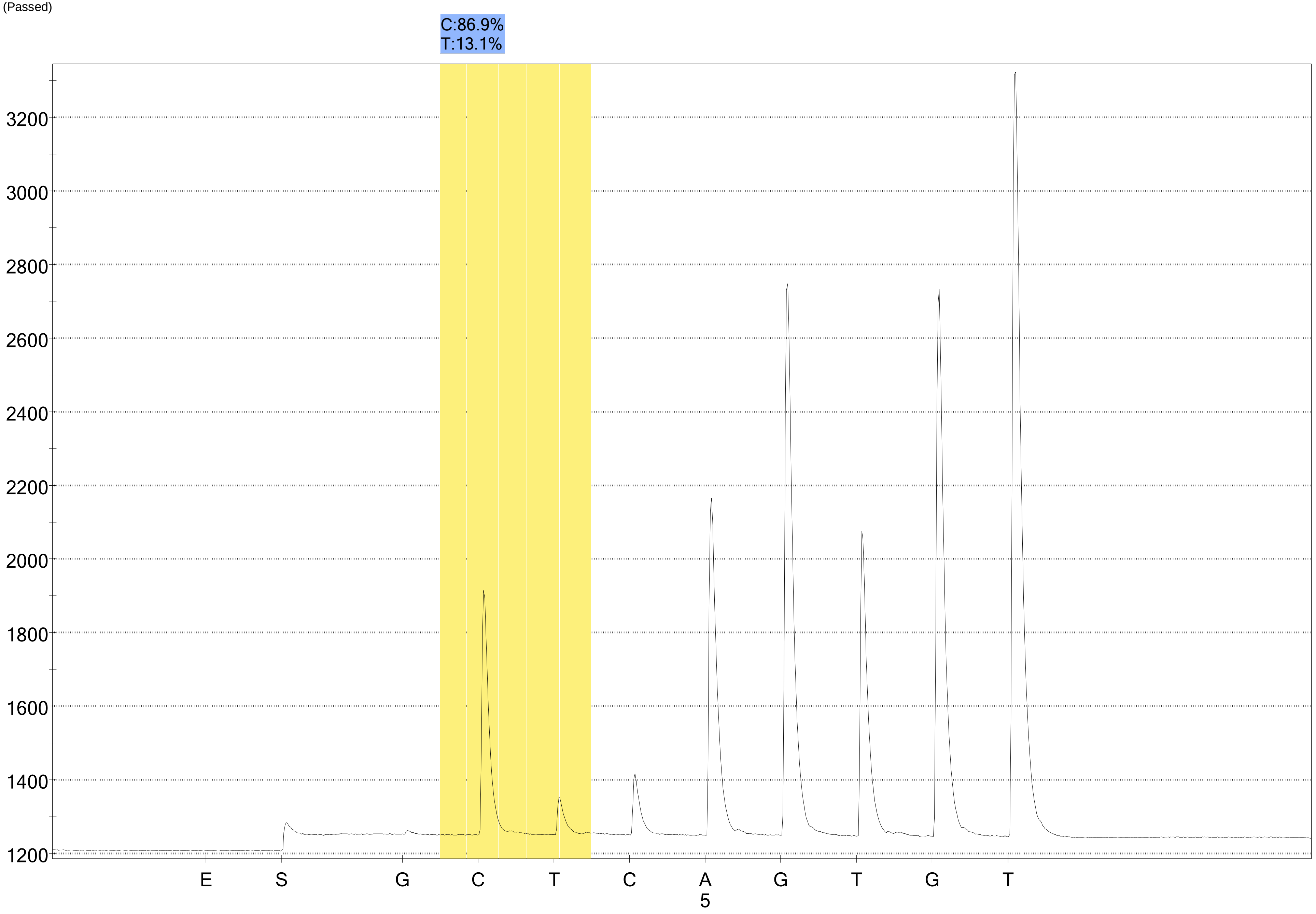

145 - Well B1
Entry: 5230400G24Rik
1: C: 87.5% / T: 12.5%
(Passed)

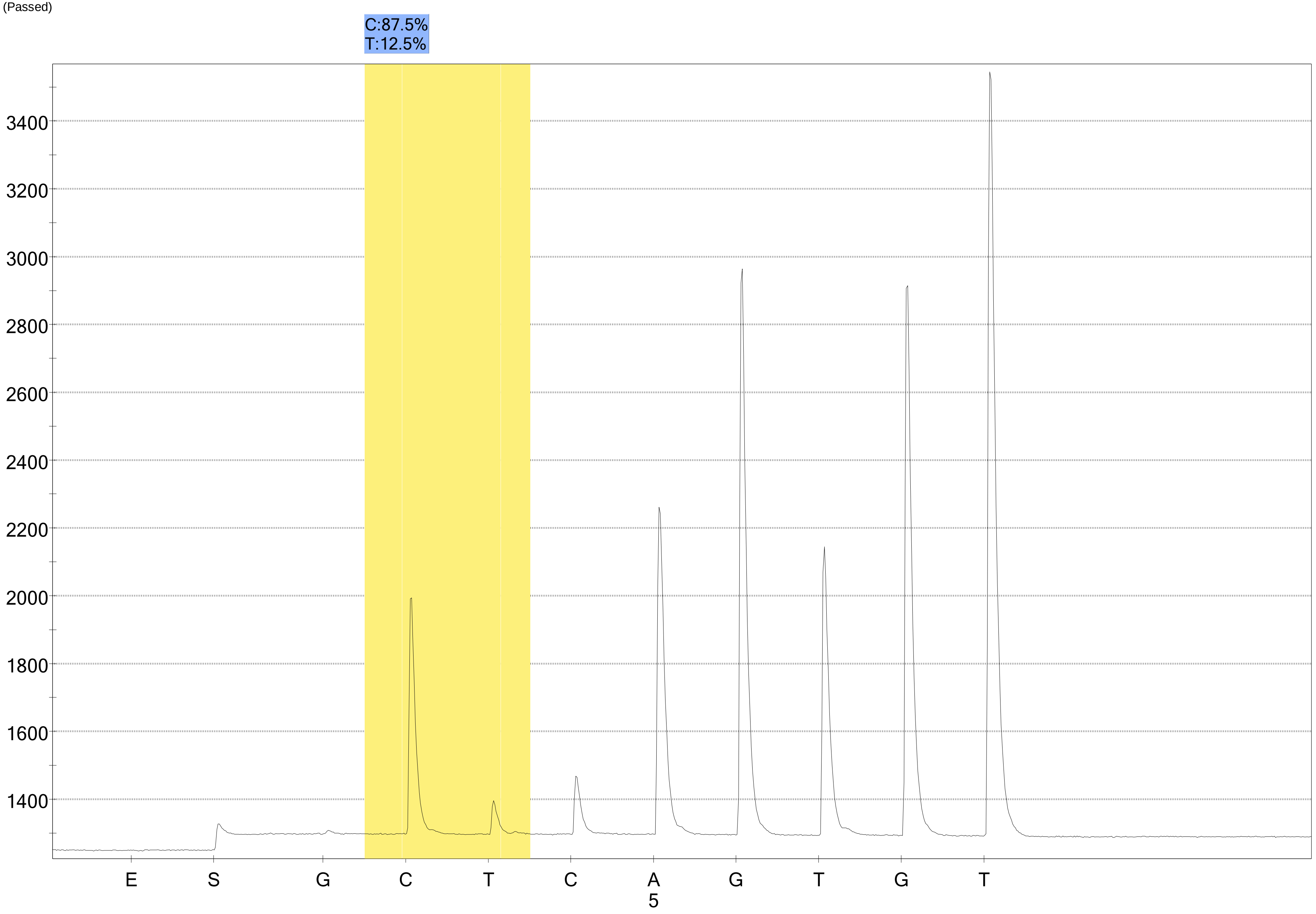

dna - Well B1
Entry: 5230400G24Rik
1: C: 89.9% / T: 10.1%
(Passed)

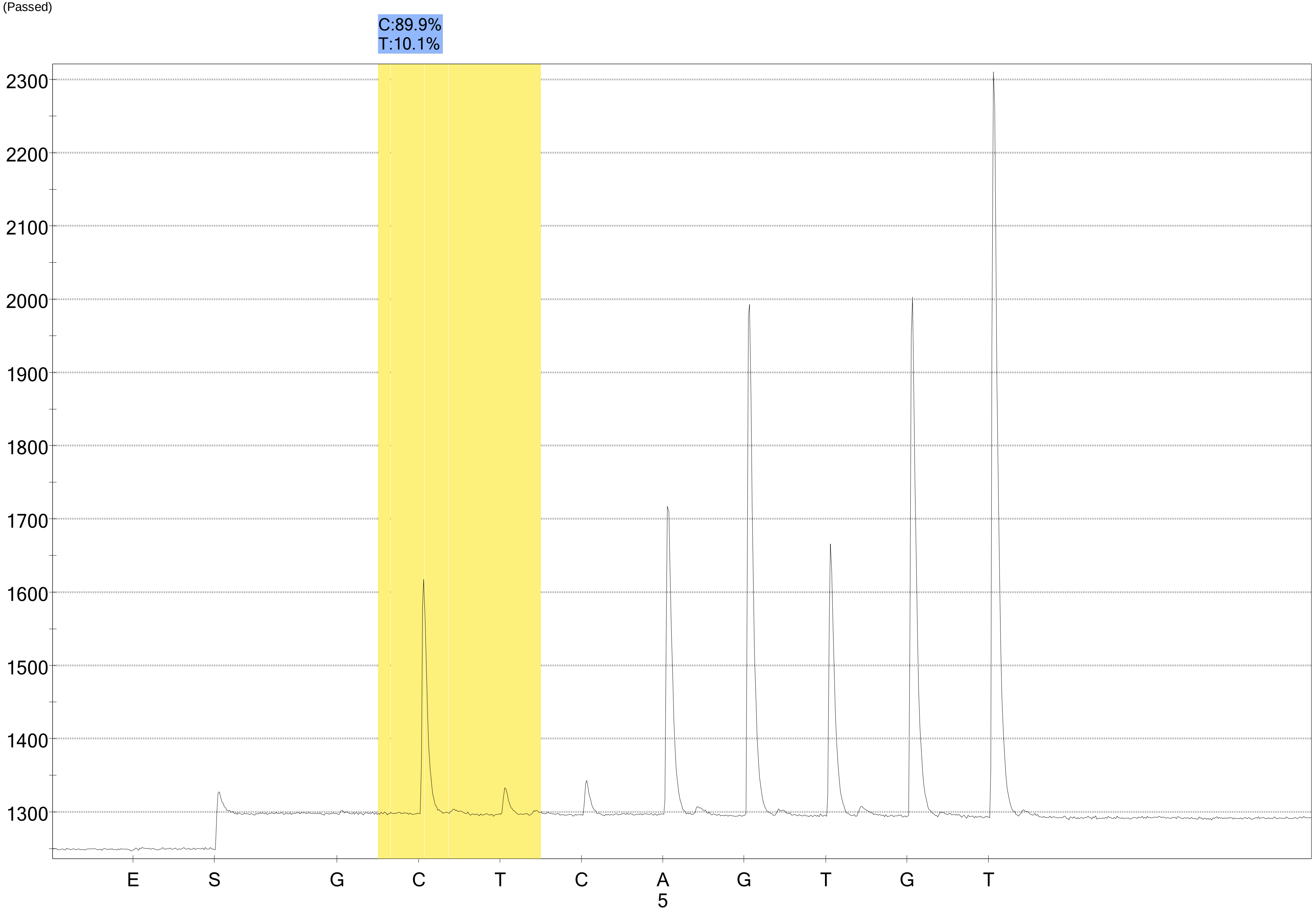

dna - Well B7
Entry: 5230400G24Rik
1: C: 89.6% / T: 10.4%
(Passed)

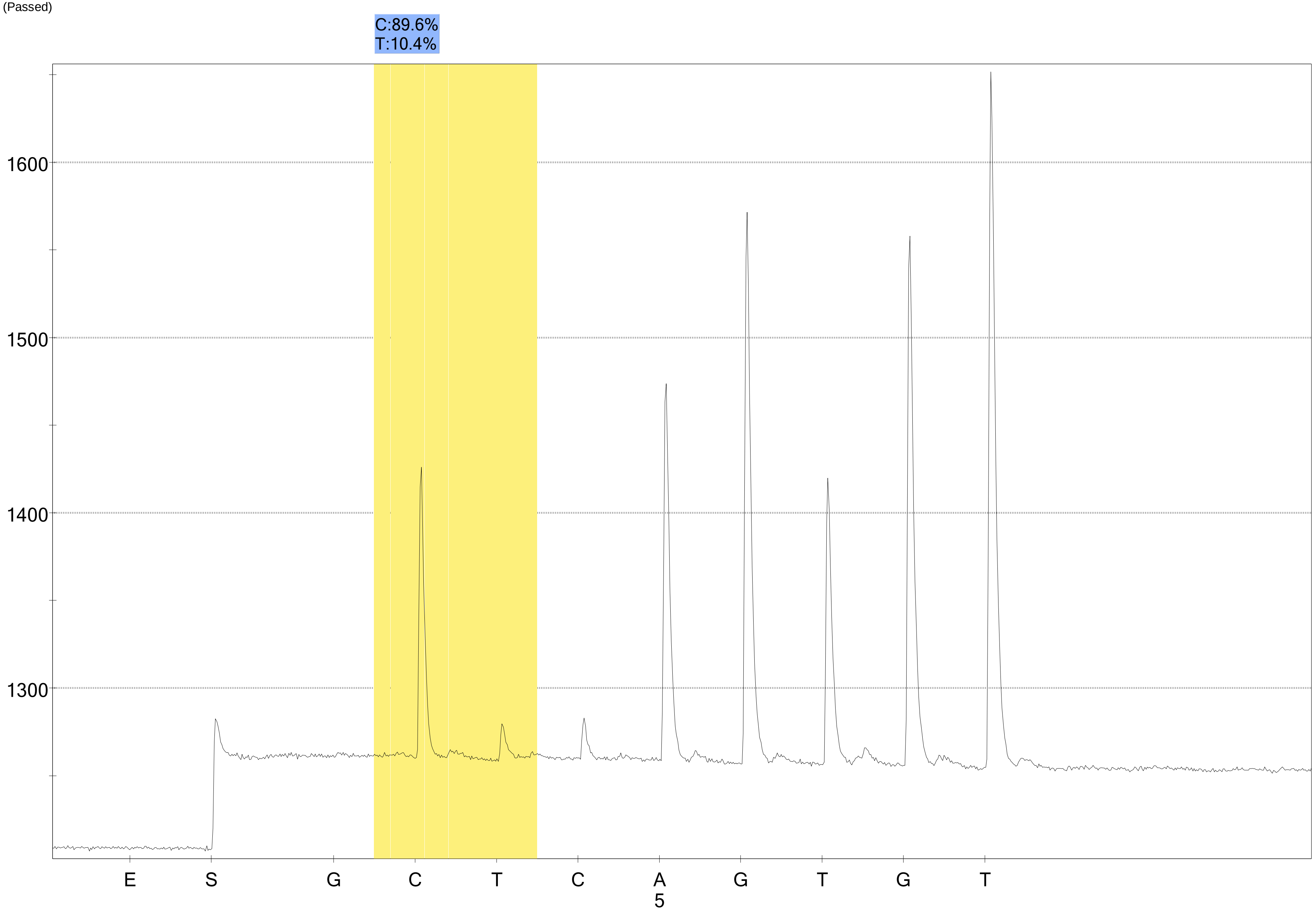

10 uL universal (141+157) - Well A4
Entry: Hsp90ab1
3: C: 56.2% / A: 43.8%
(Passed)

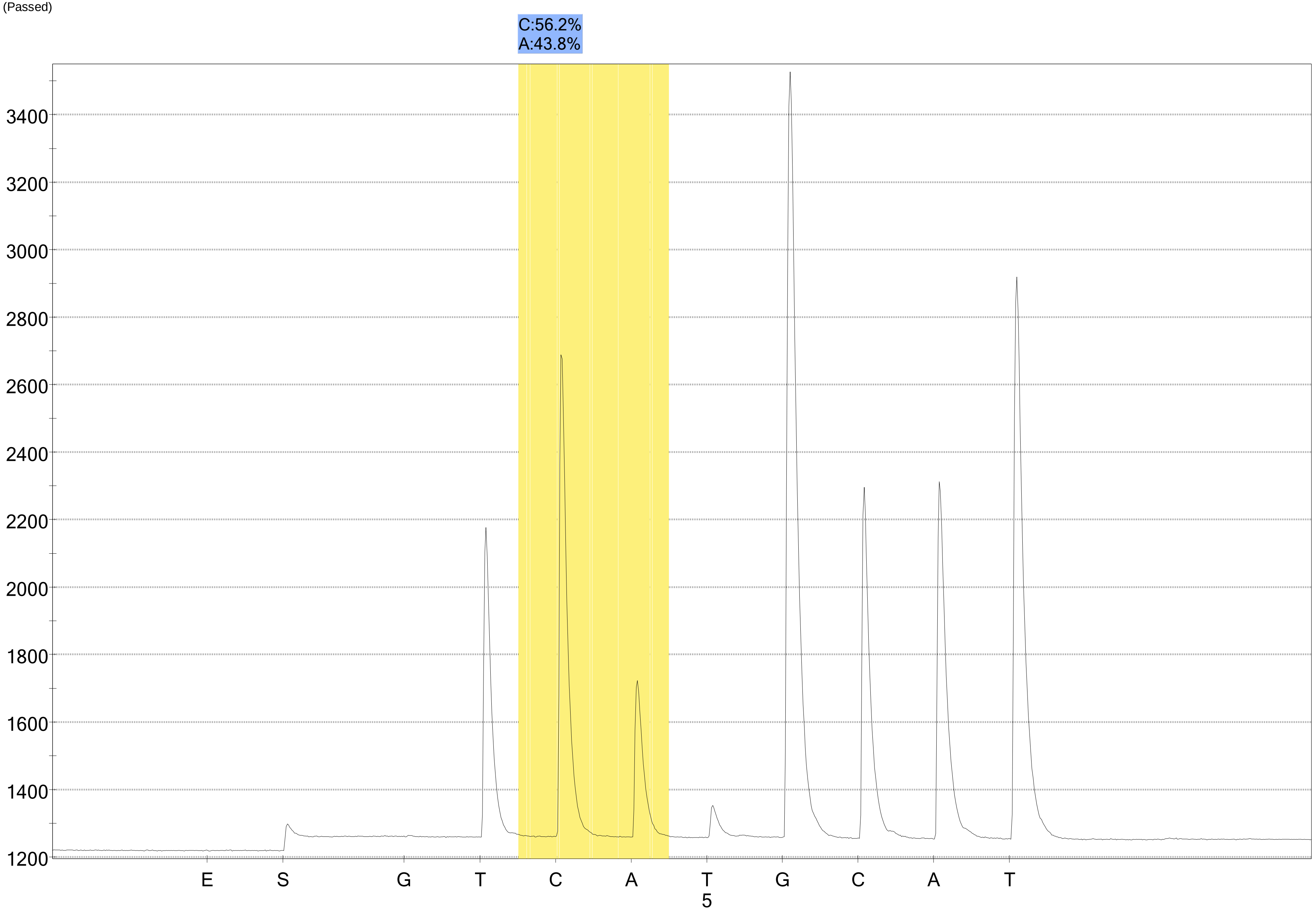

10 uL universal (141+157) - Well A10
Entry: Hsp90ab1
3: C: 61.3% / A: 38.7%
(Passed)

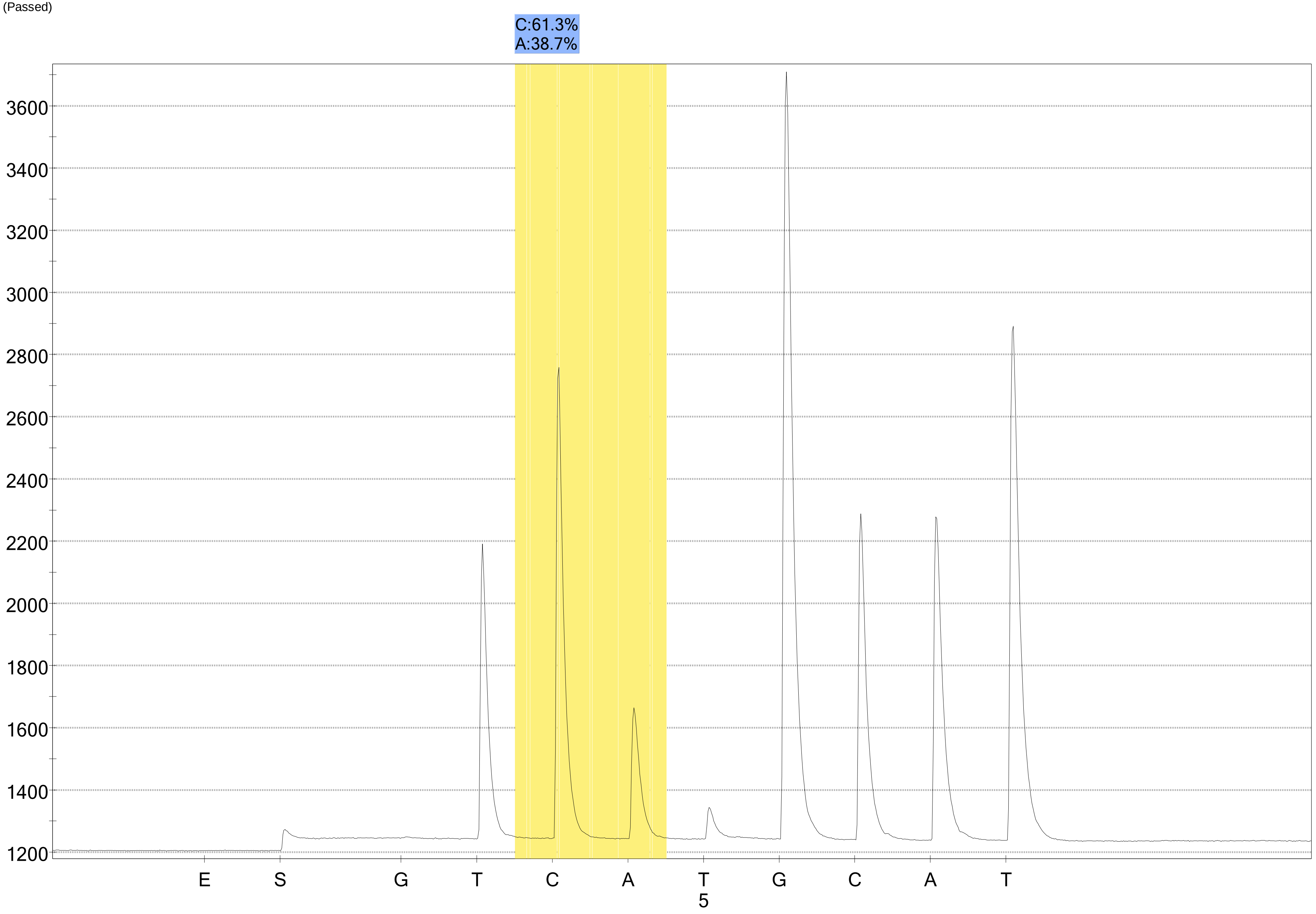

145 - Well A4
Entry: Hsp90ab1
3: C: 61.4% / A: 38.6%
(Passed)

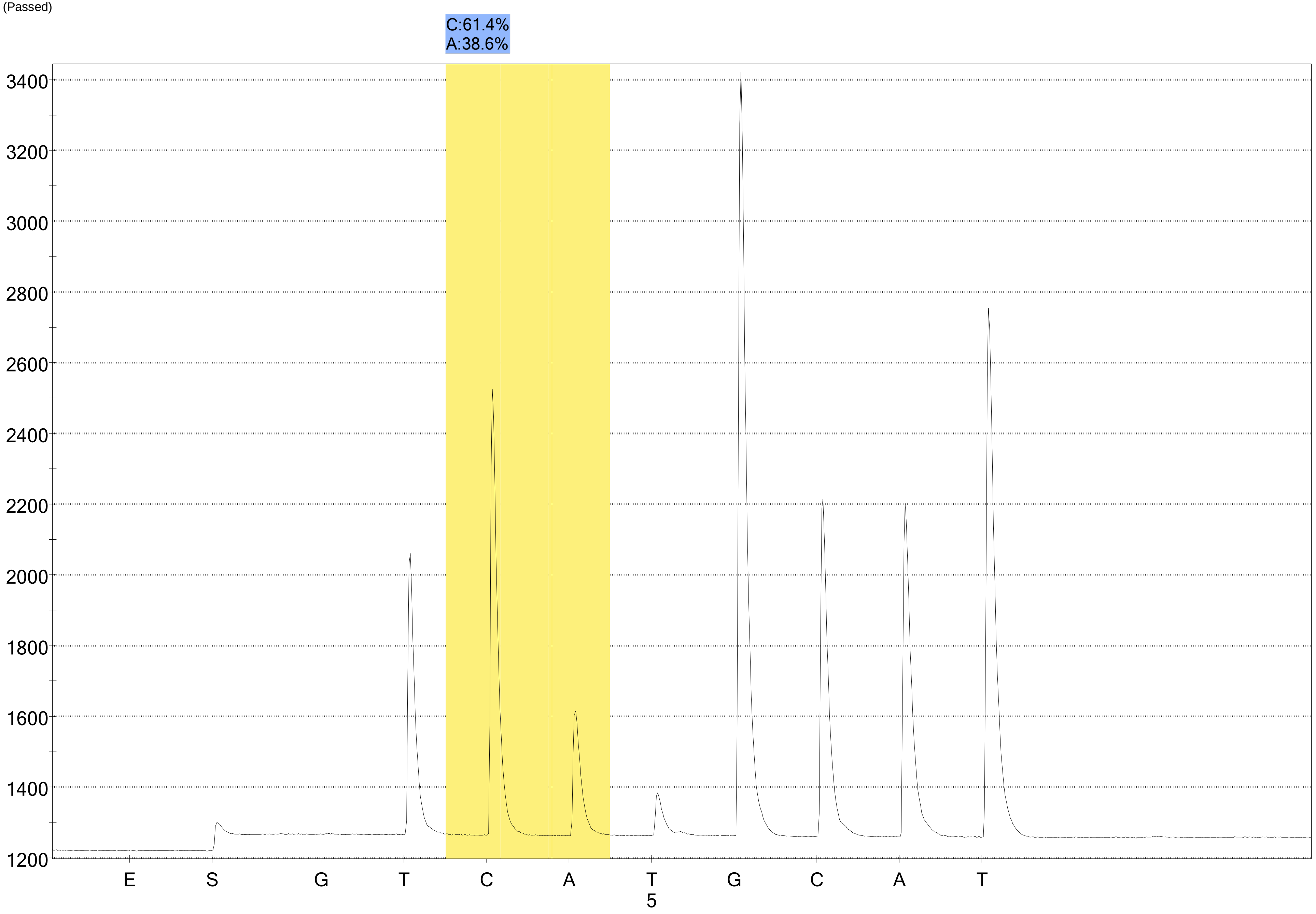

dna - Well A4
Entry: Hsp90ab1
3: C: 53.5% / A: 46.5%
(Passed)

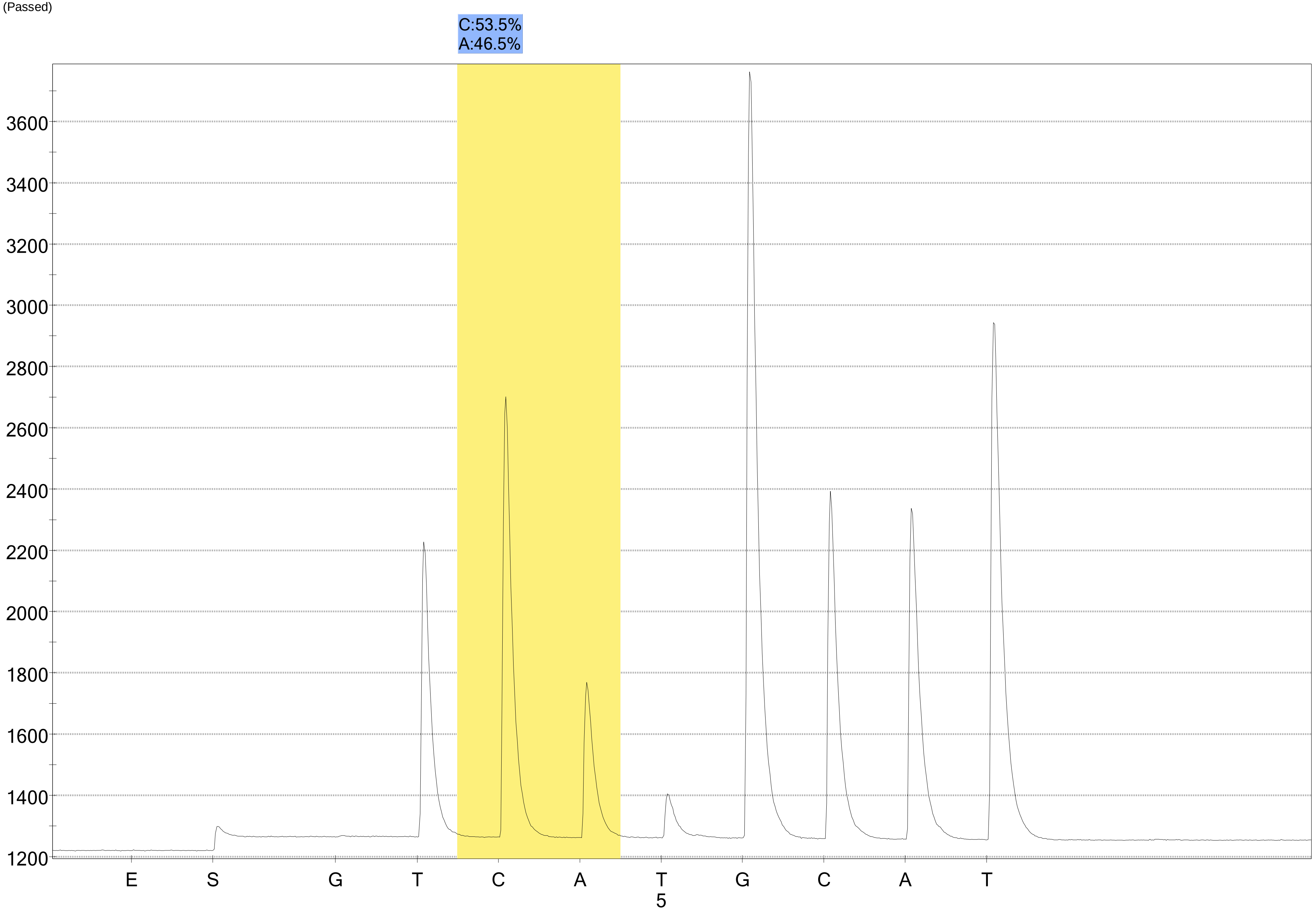

dna - Well A10
Entry: Hsp90ab1
3: C: 49.7% / A: 50.3%
(Passed)

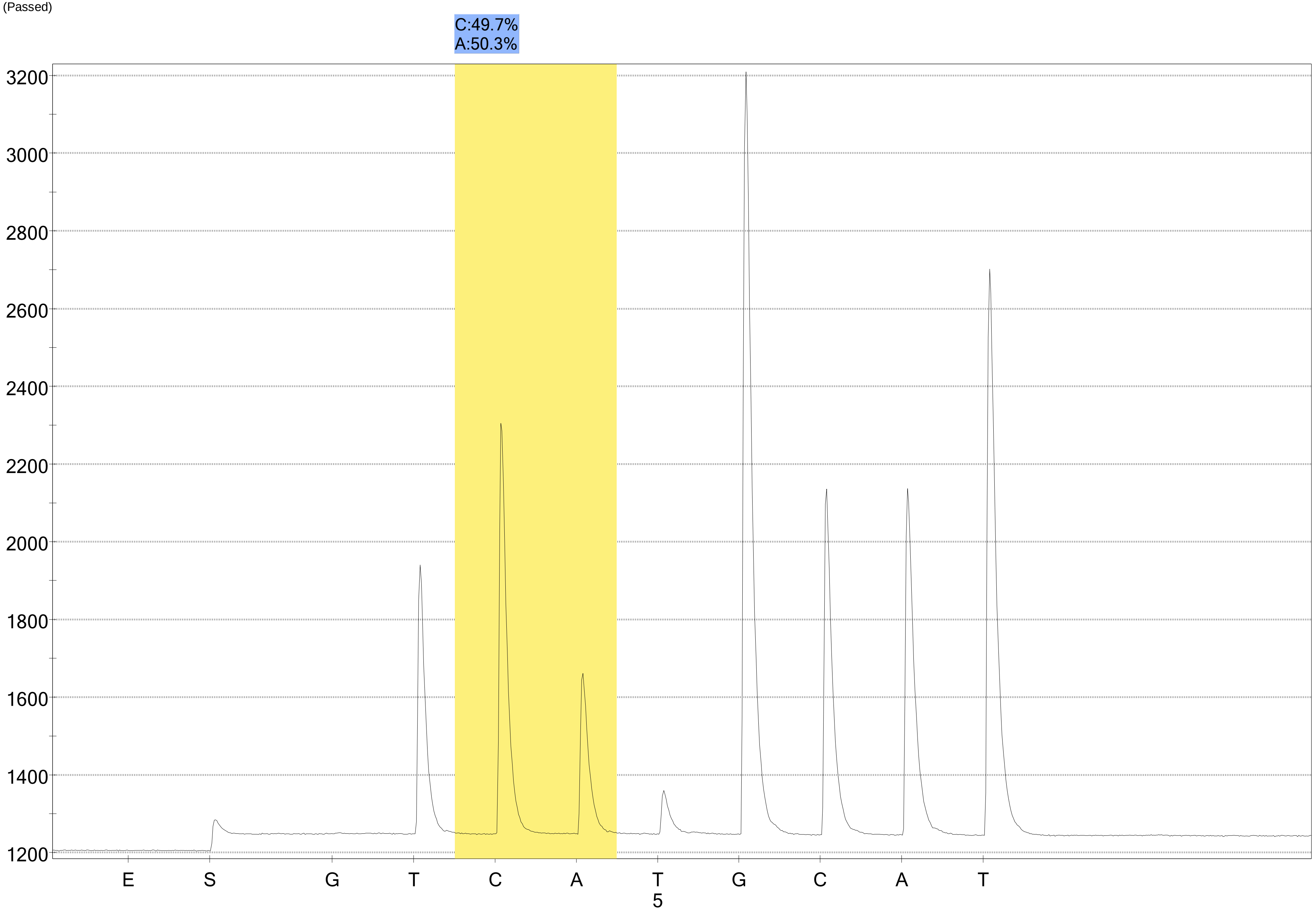

10 uL universal (141+157) - Well A5
Entry: Nme1
3: T: 63.3% / C: 36.7%
(Passed)

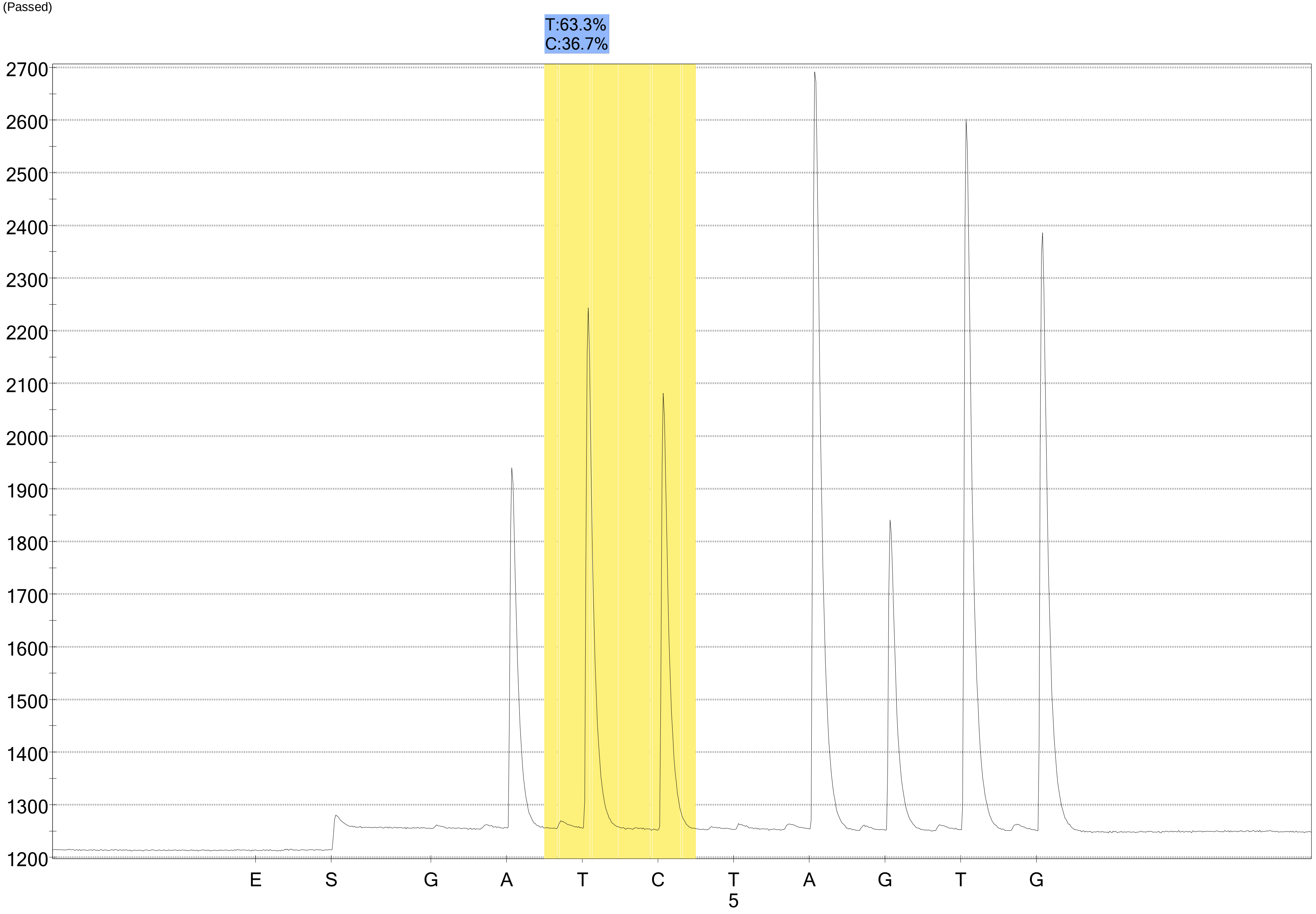

10 uL universal (141+157) - Well A11
Entry: Nme1
3: T: 61.9% / C: 38.1%
(Passed)

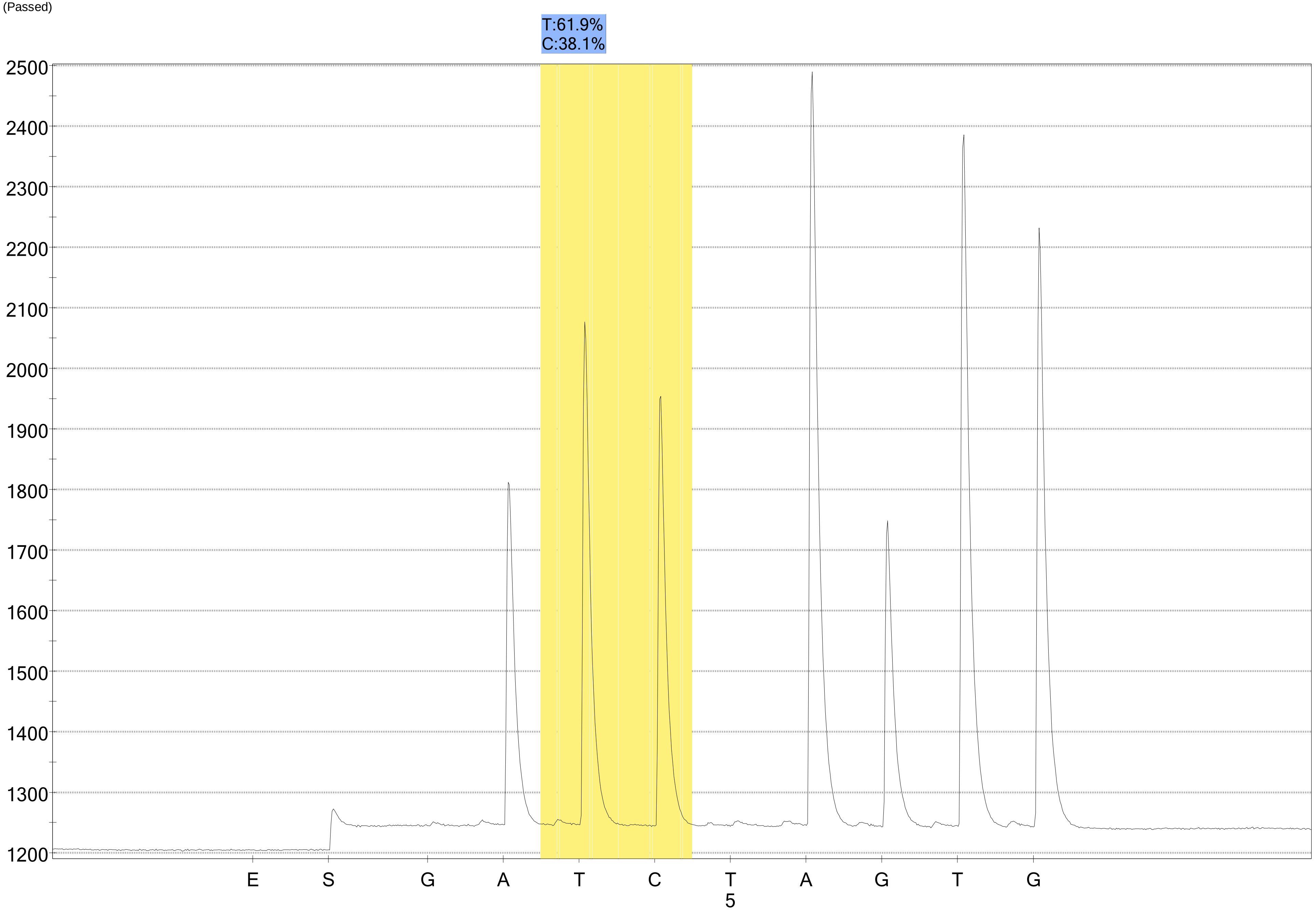

145 - Well A5
Entry: Nme1
3: T: 59.2% / C: 40.8%
(Passed)

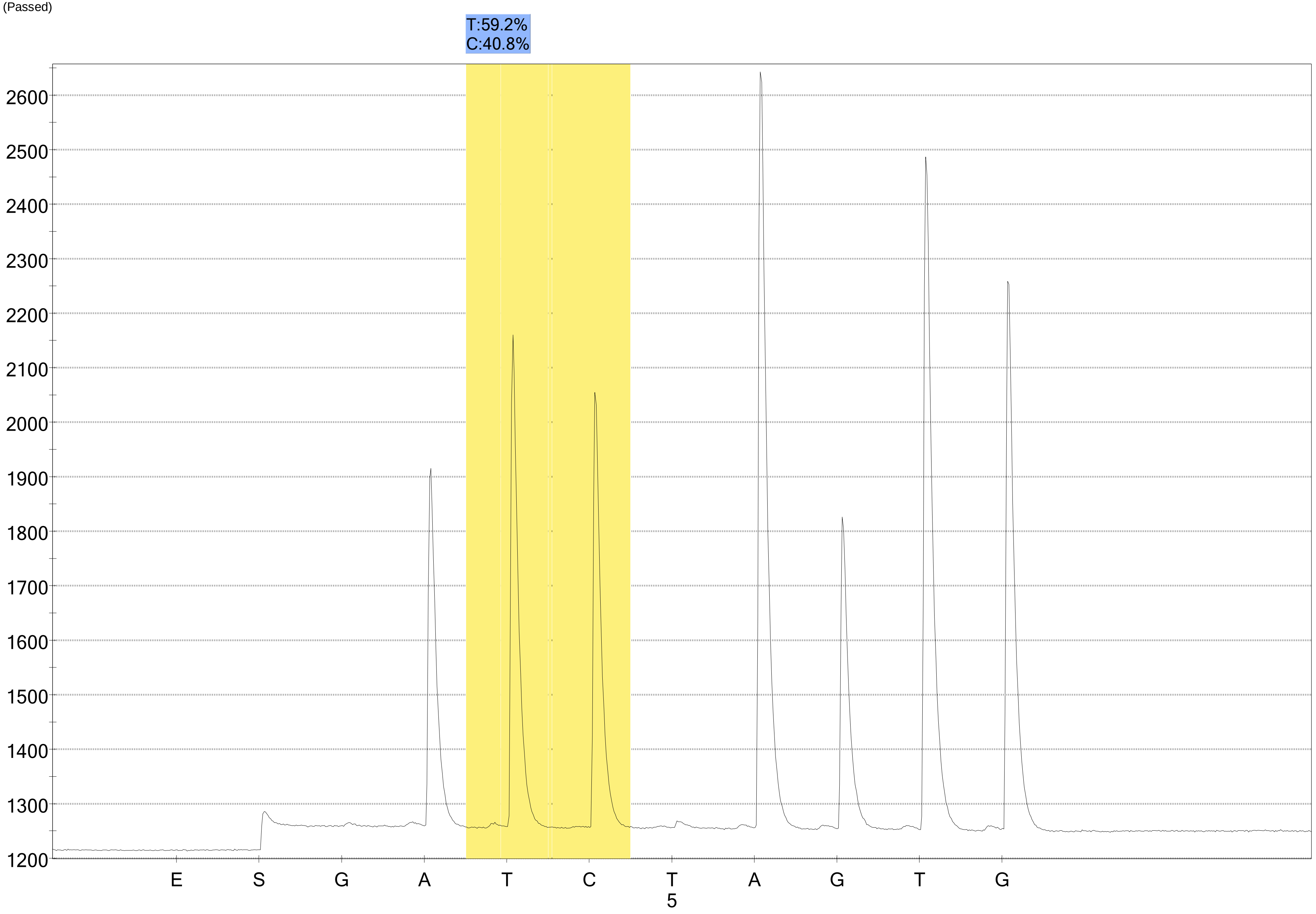

dna - Well A5
Entry: Nme1
3: T: 48.3% / C: 51.7%
(Passed)

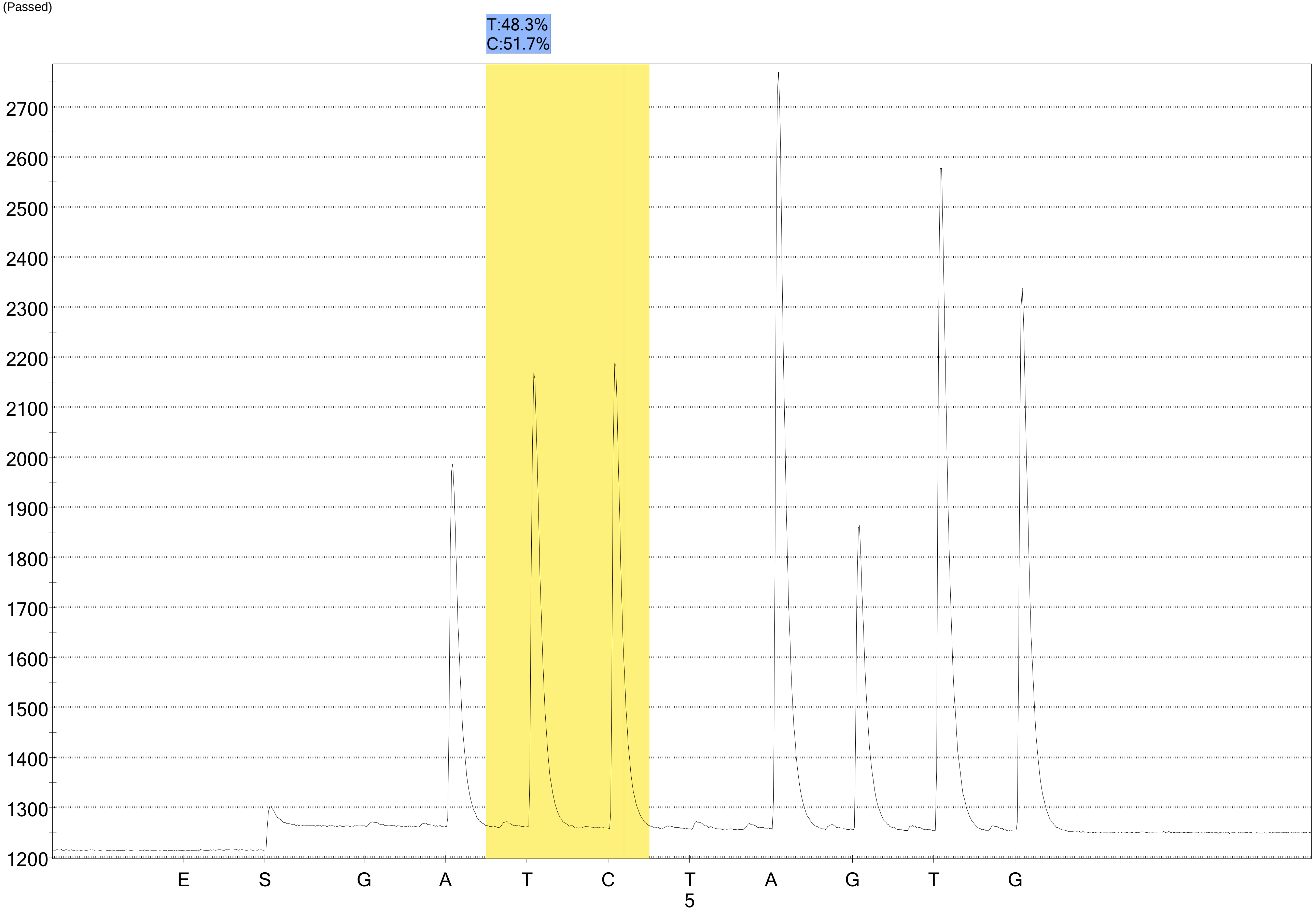

dna - Well A11
Entry: Nme1
3: T: 48.9% / C: 51.1%
(Passed)

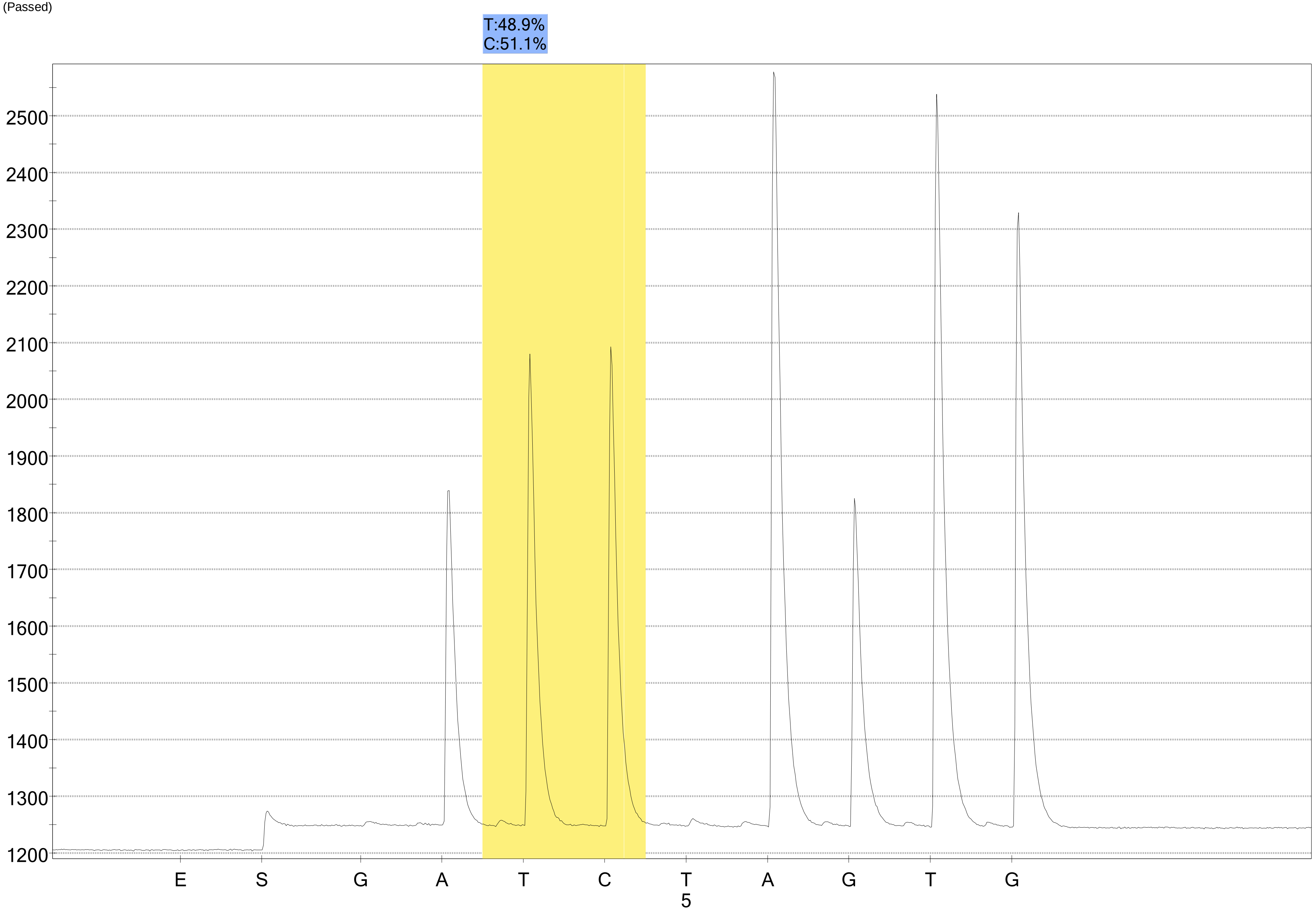

10 uL universal (141+157) - Well C5
Entry: Nsg2
4: A: 41.7% / G: 58.3%
(Passed)

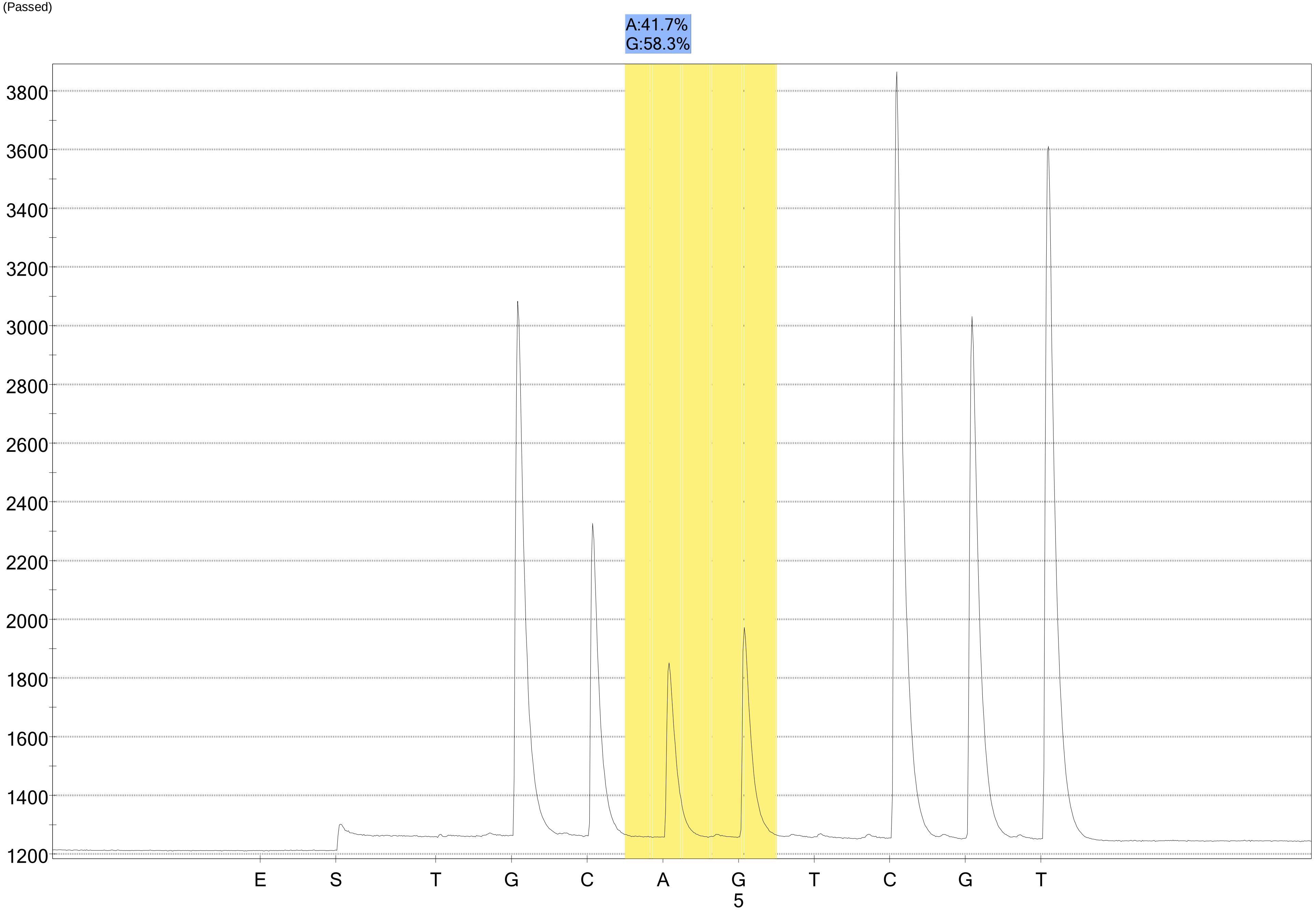

10 uL universal (141+157) - Well C11
Entry: Nsg2
4: A: 41.2% / G: 58.8%
(Passed)

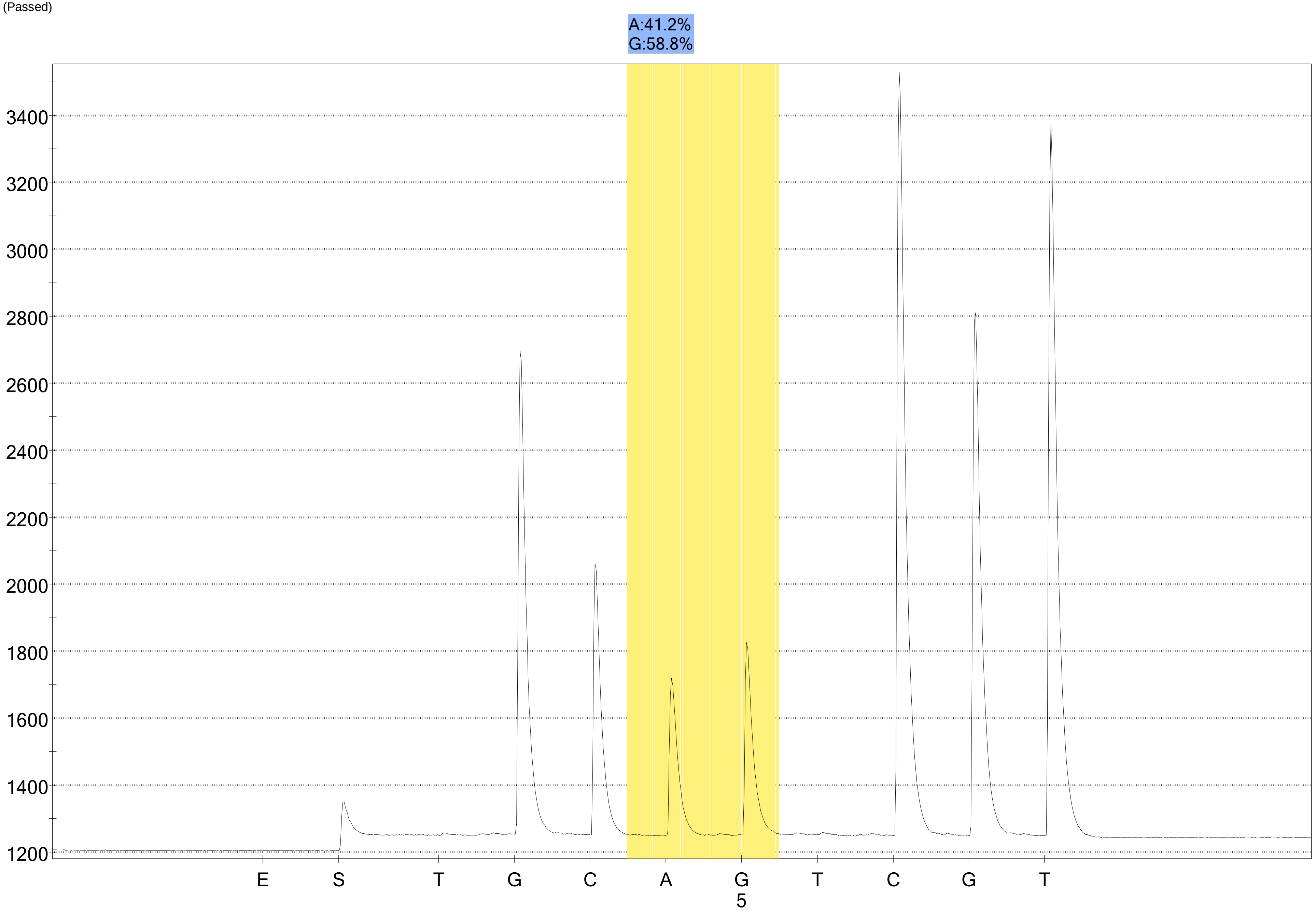

145 - Well C5
Entry: Nsg2
4: A: 38.9% / G: 61.1%
(Passed)

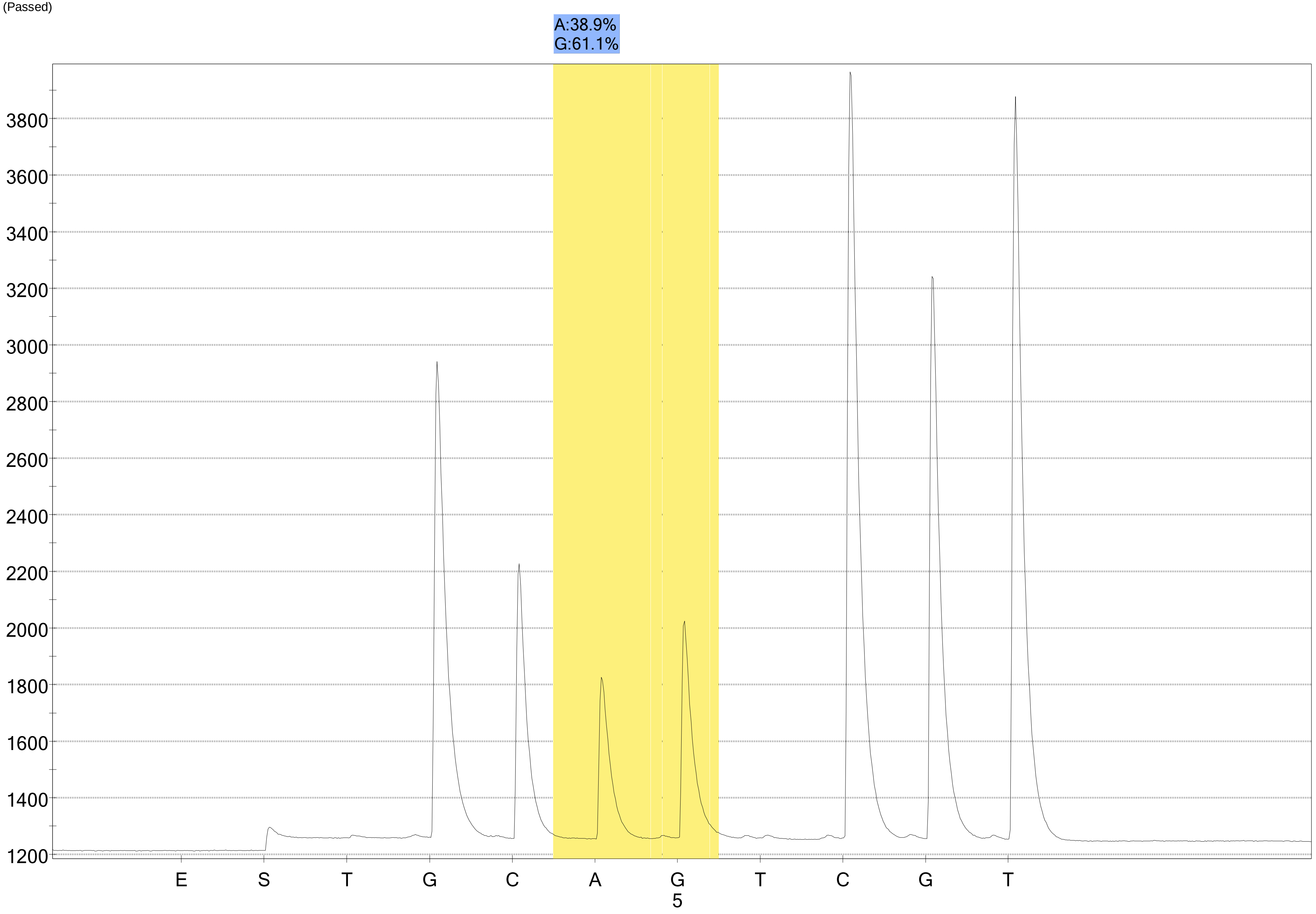

dna - Well C5
Entry: Nsg2
4: A: 43.3% / G: 56.7%
(Passed)

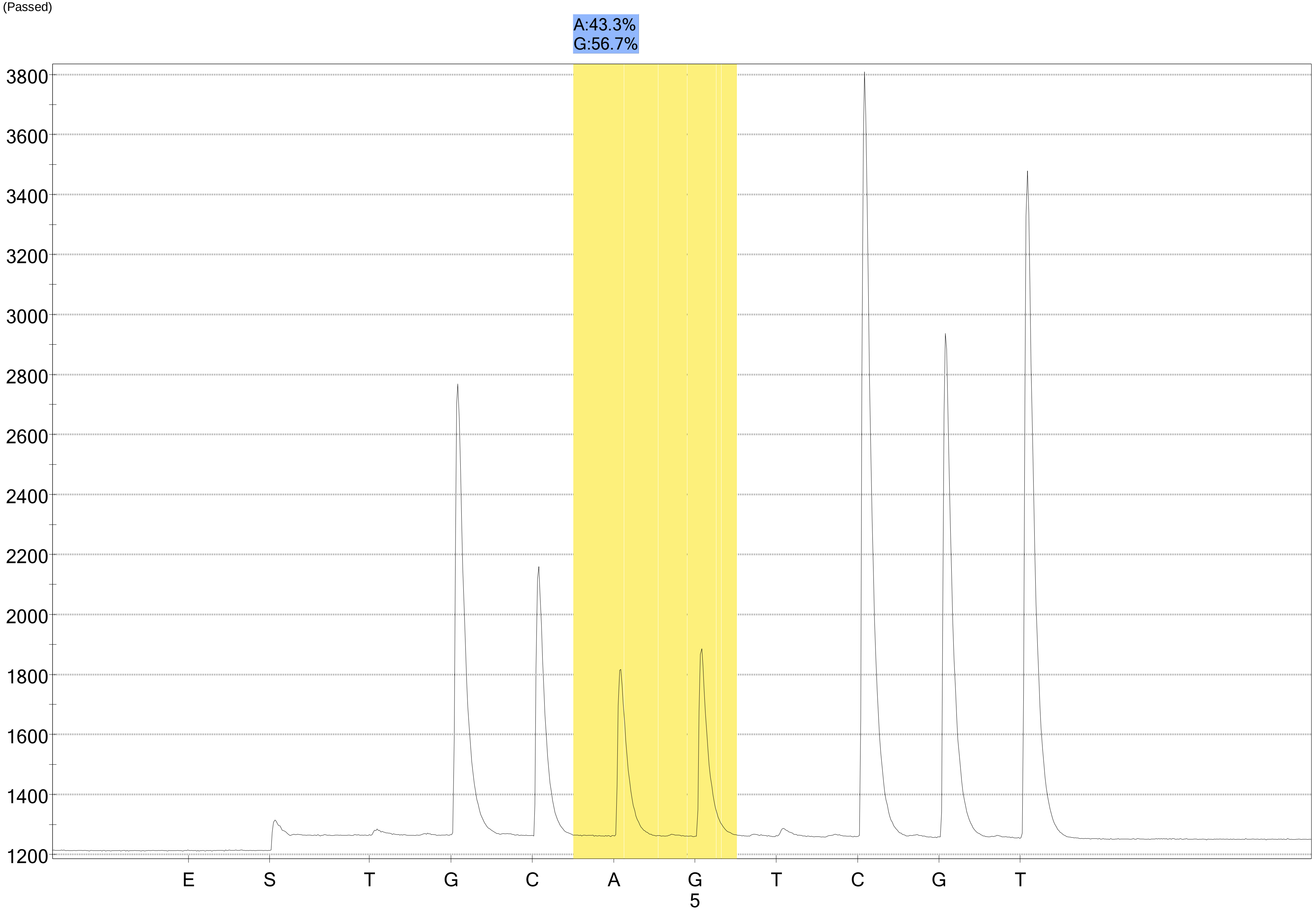

dna - Well C11
Entry: Nsg2
4: A: 43.7% / G: 56.3%
(Passed)

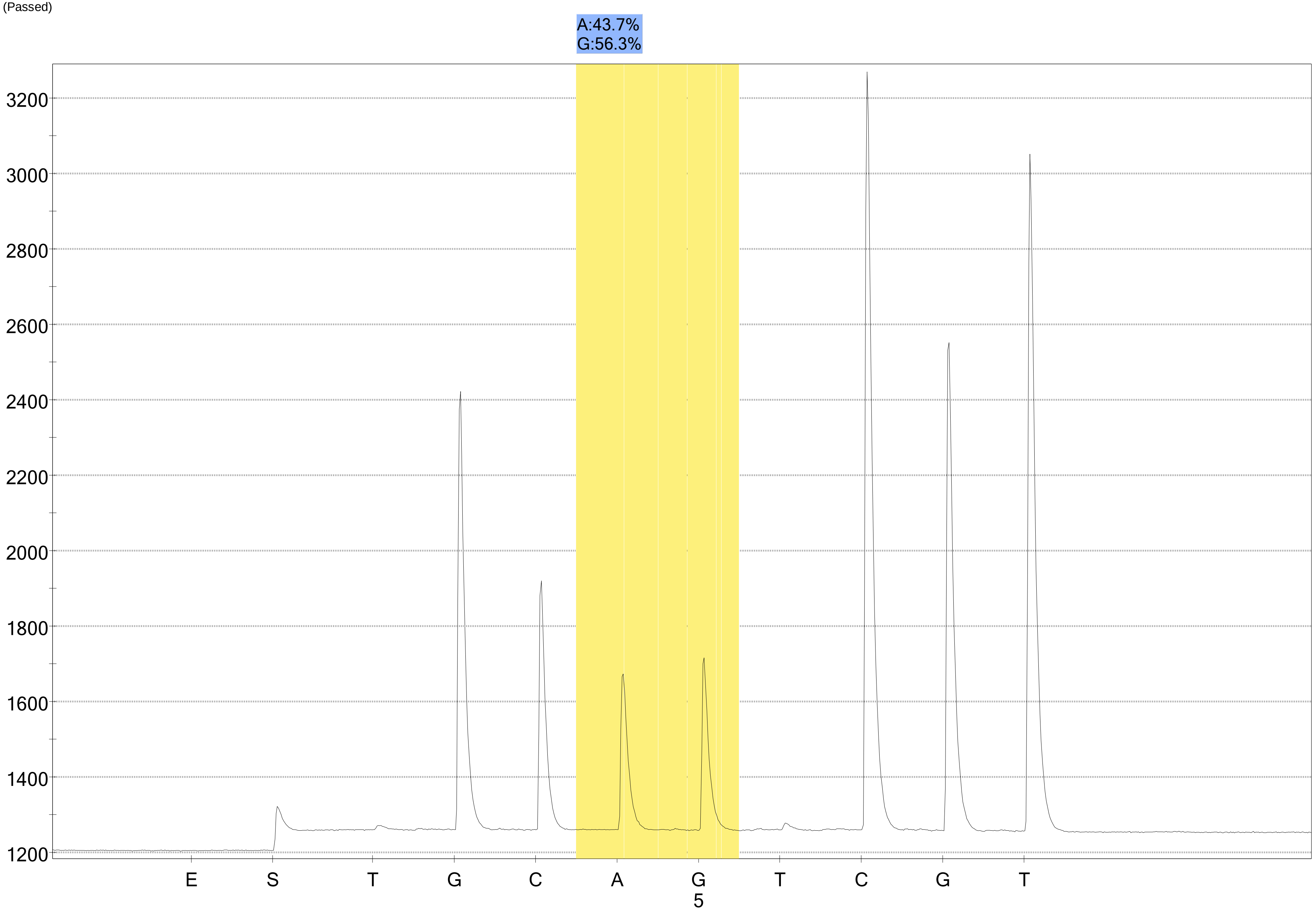

10 uL universal (141+157) - Well F1
Entry: AK138412
4: T: 72.8% / C: 27.2%
(Passed)

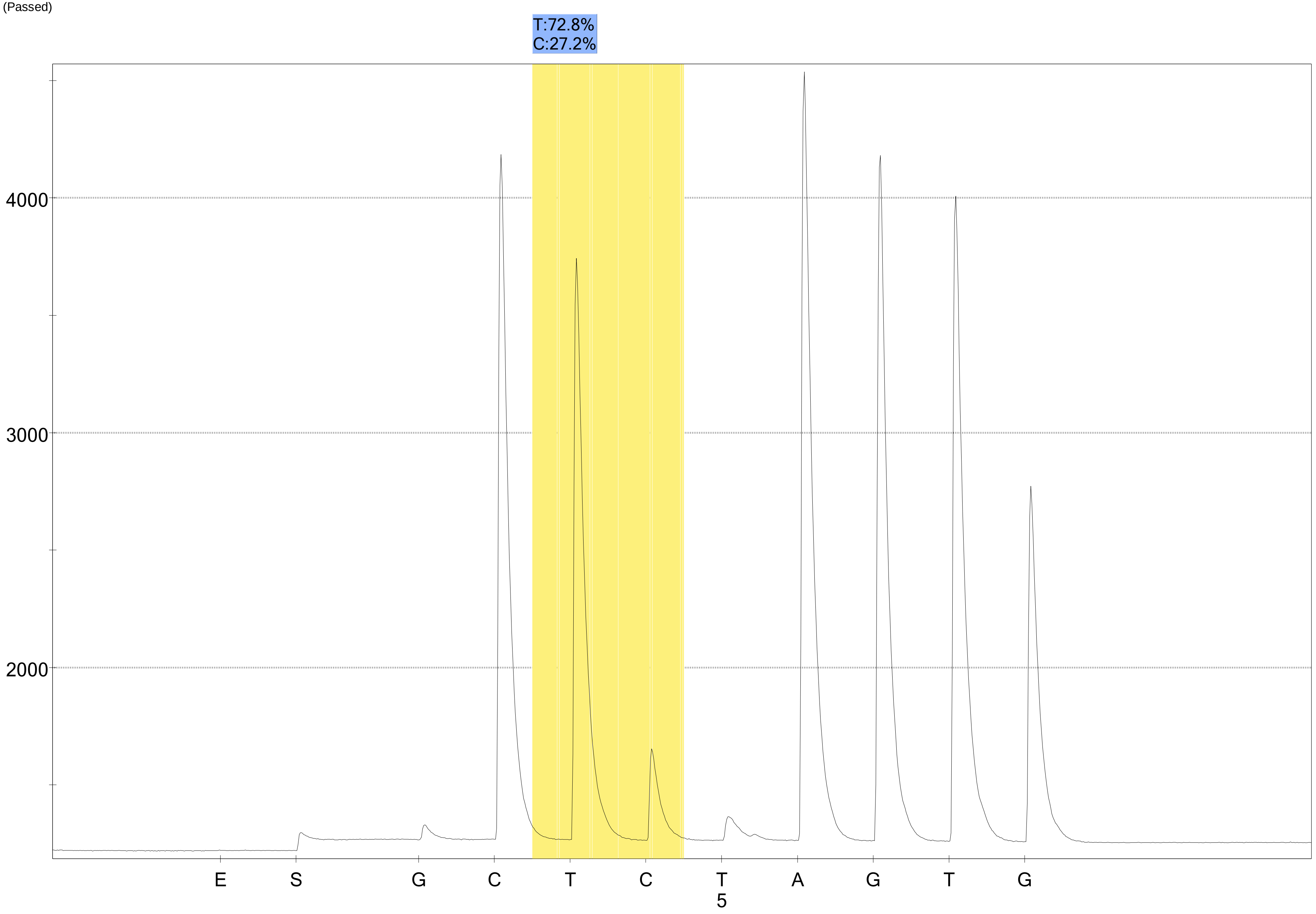

10 uL universal (141+157) - Well F7
Entry: AK138412
4: T: 69.2% / C: 30.8%
(Passed)

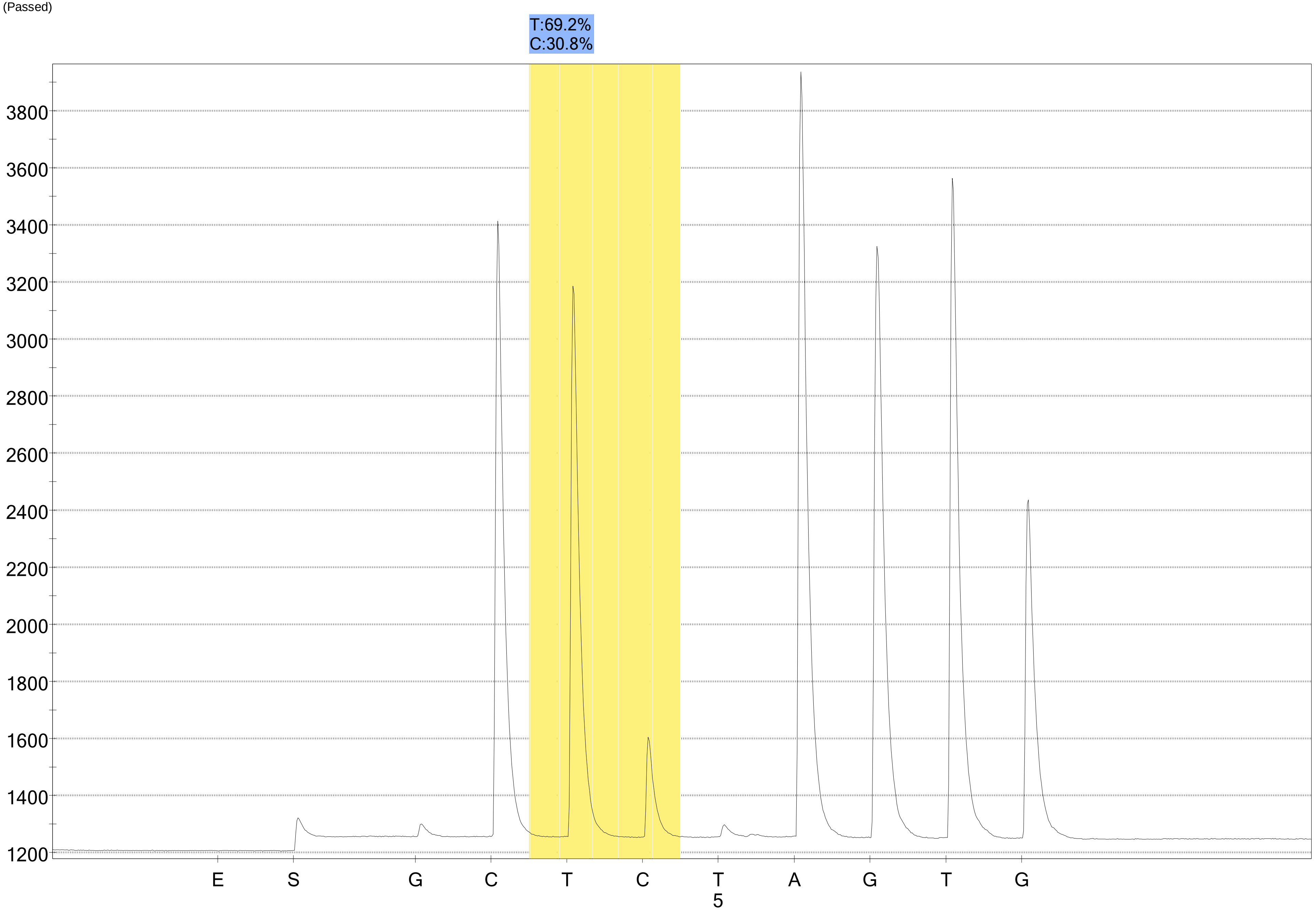

145 - Well F1
Entry: AK138412
4: T: 65.6% / C: 34.4%
(Passed)

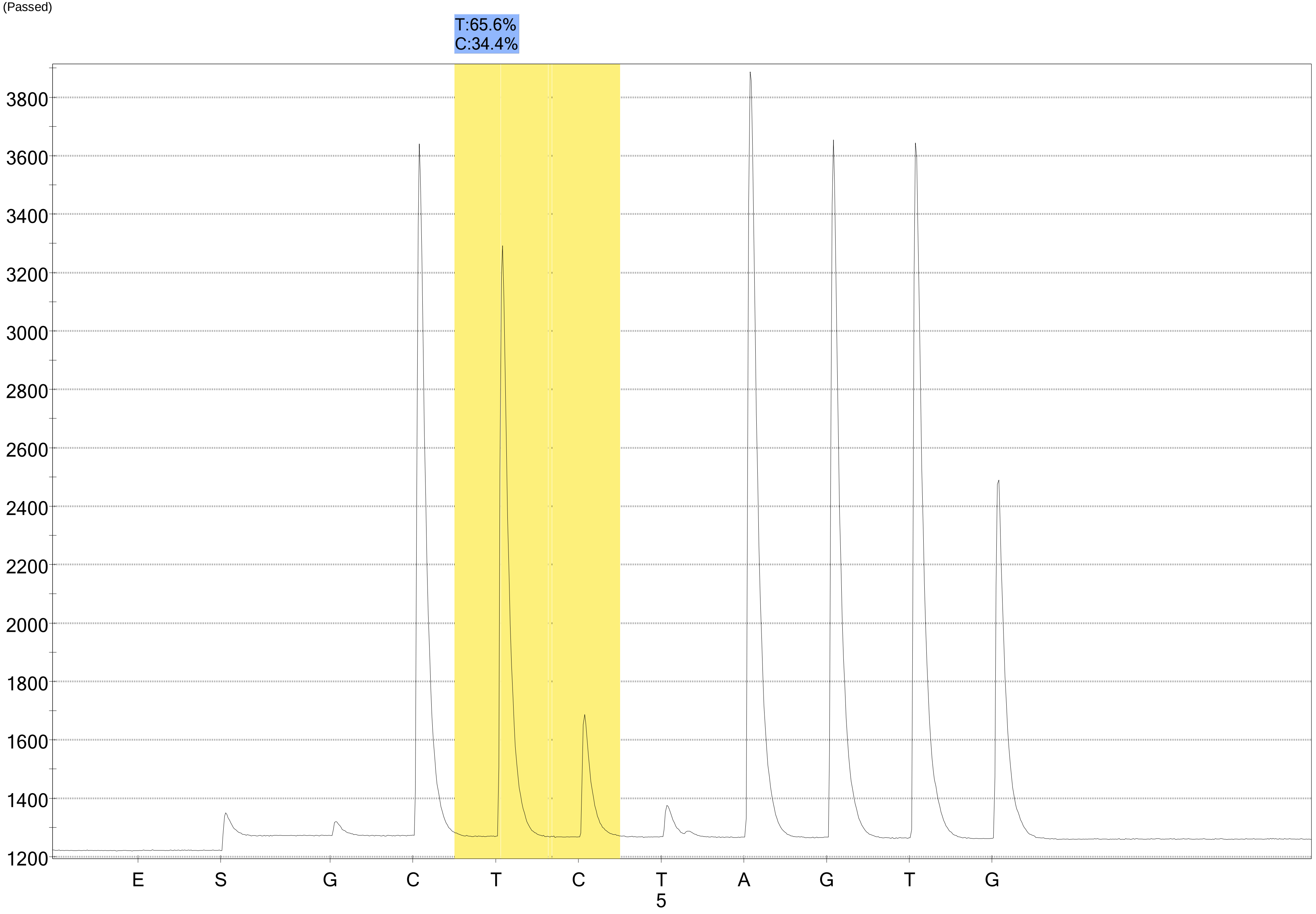

dna - Well F1
Entry: AK138412
4: T: 65.6% / C: 34.4%
(Passed)

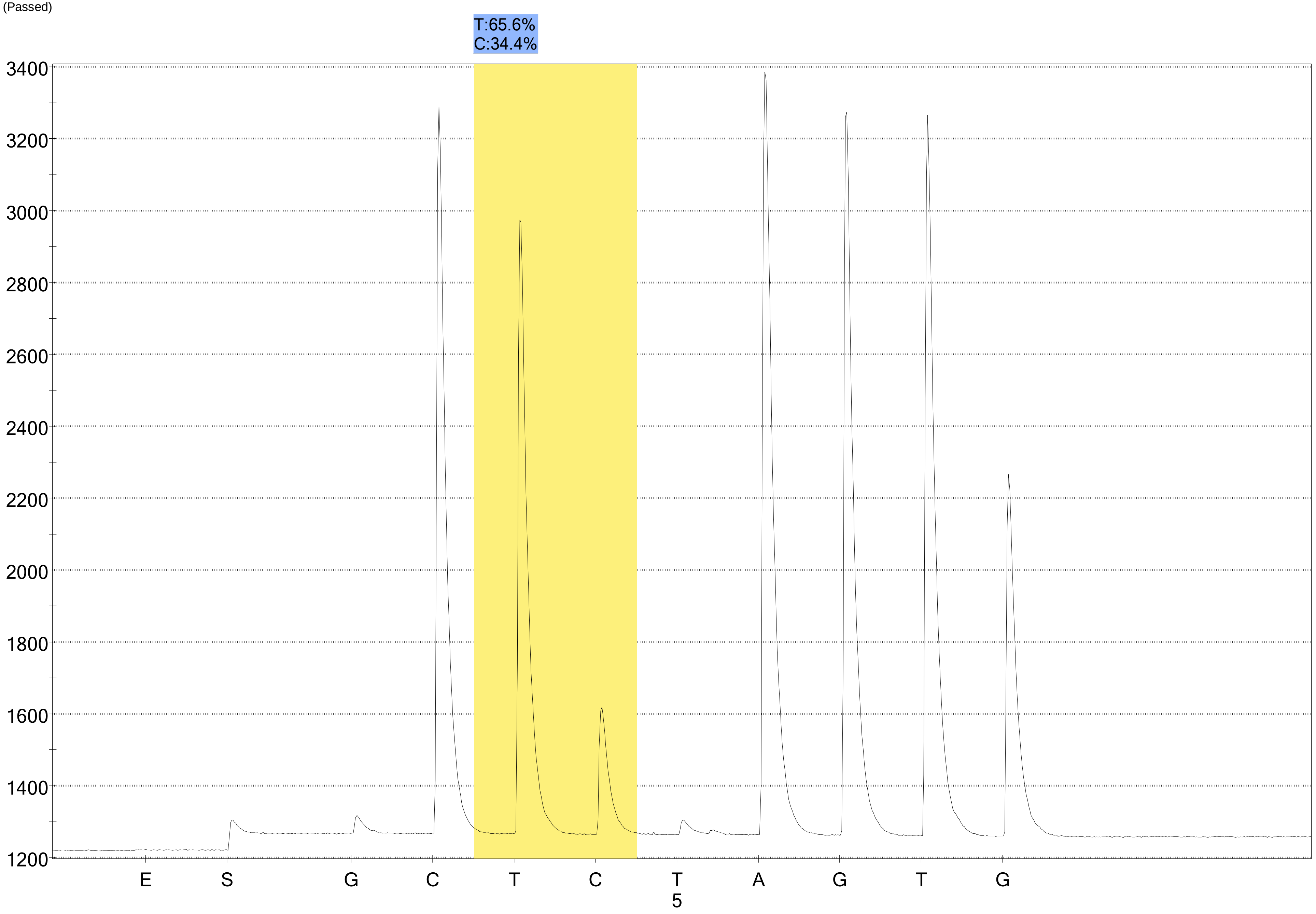

dna - Well F7
Entry: AK138412
4: T: 73.1% / C: 26.9%
(Passed)

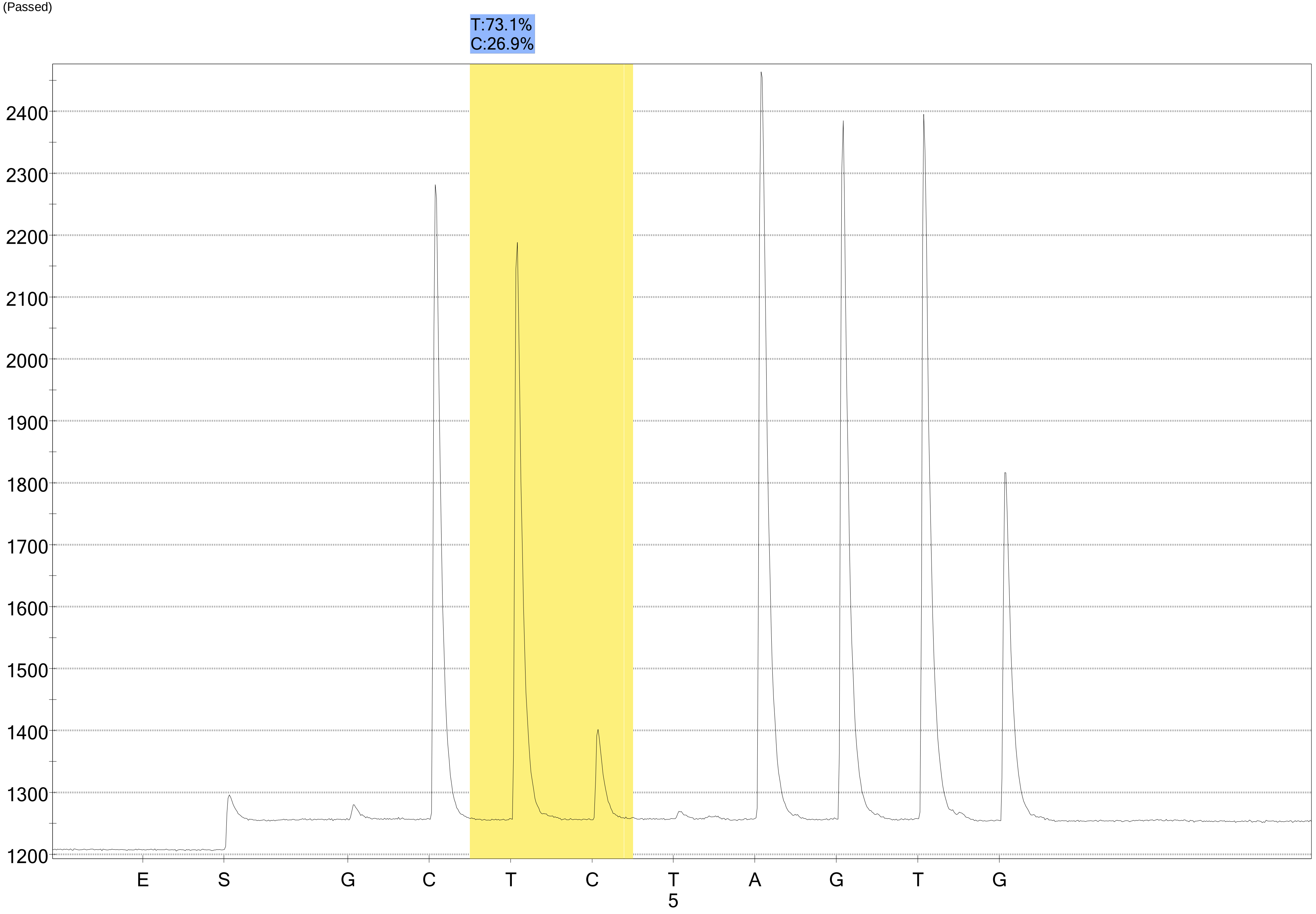

10 uL universal (141+157) - Well D2
Entry: Cald1
1: T: 42.6% / C: 57.4%
(Passed)

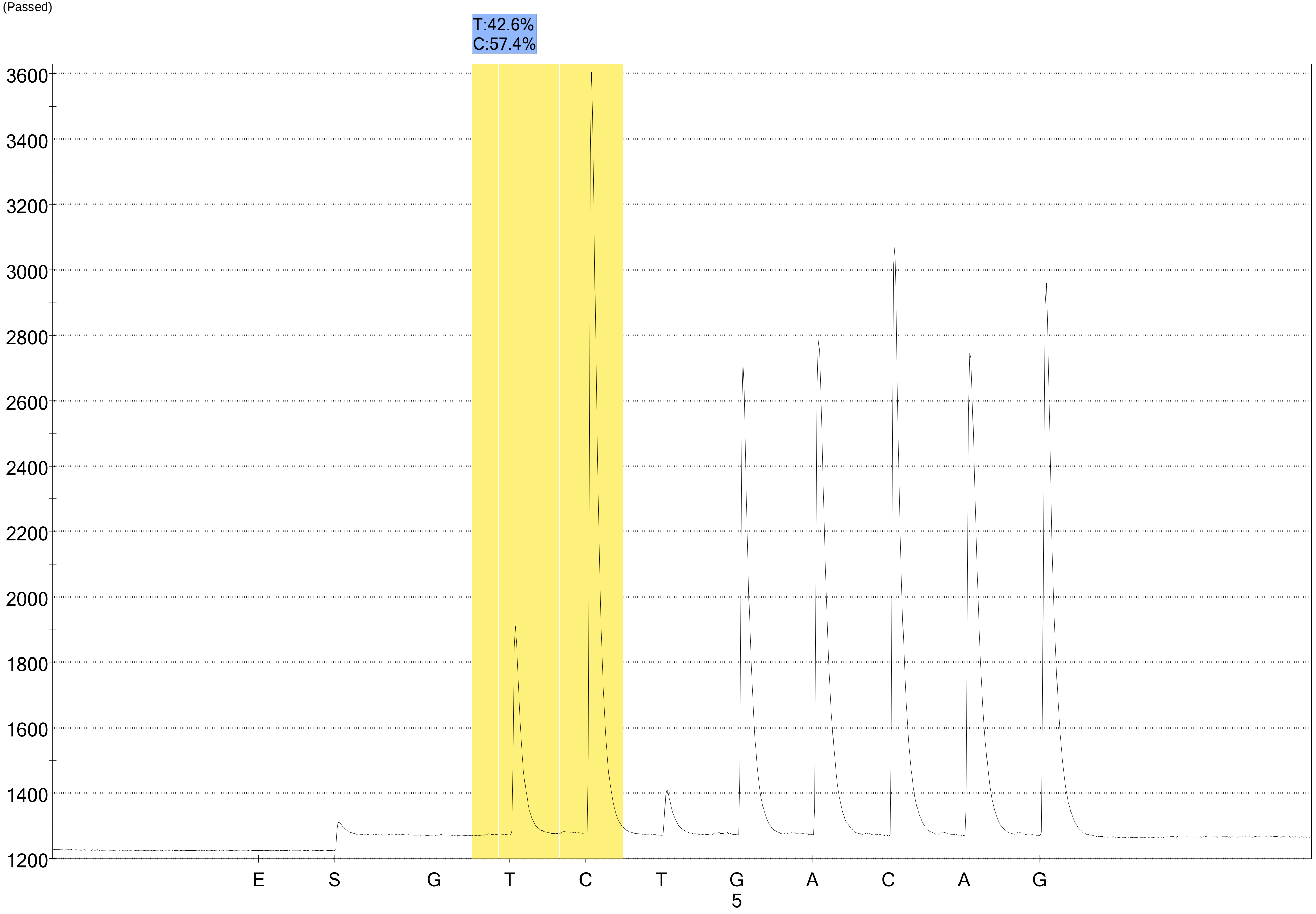

10 uL universal (141+157) - Well D8
Entry: Cald1
1: T: 43.8% / C: 56.2%
(Passed)

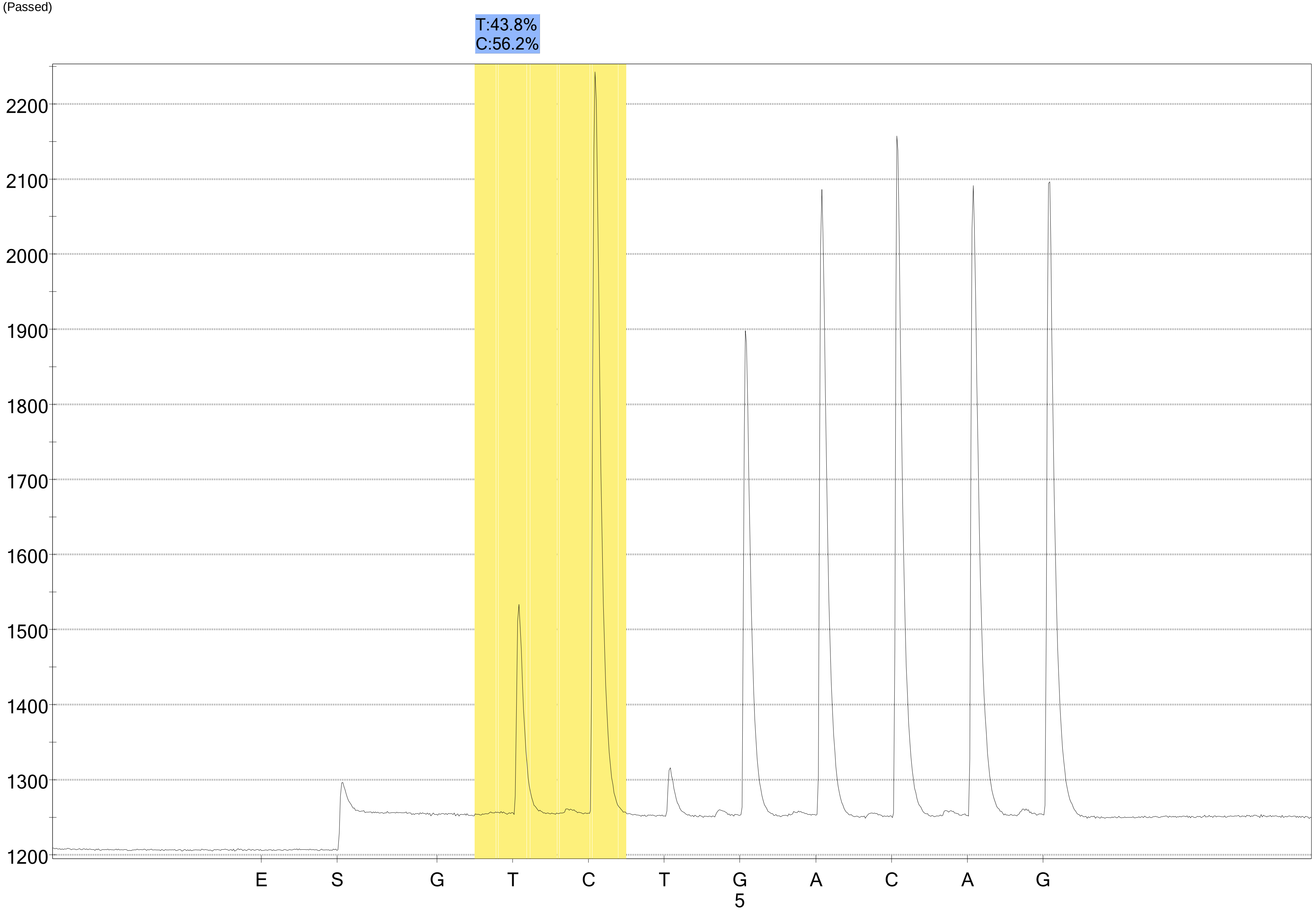

145 - Well D2
Entry: Cald1
1: T: 44.2% / C: 55.8%
(Passed)

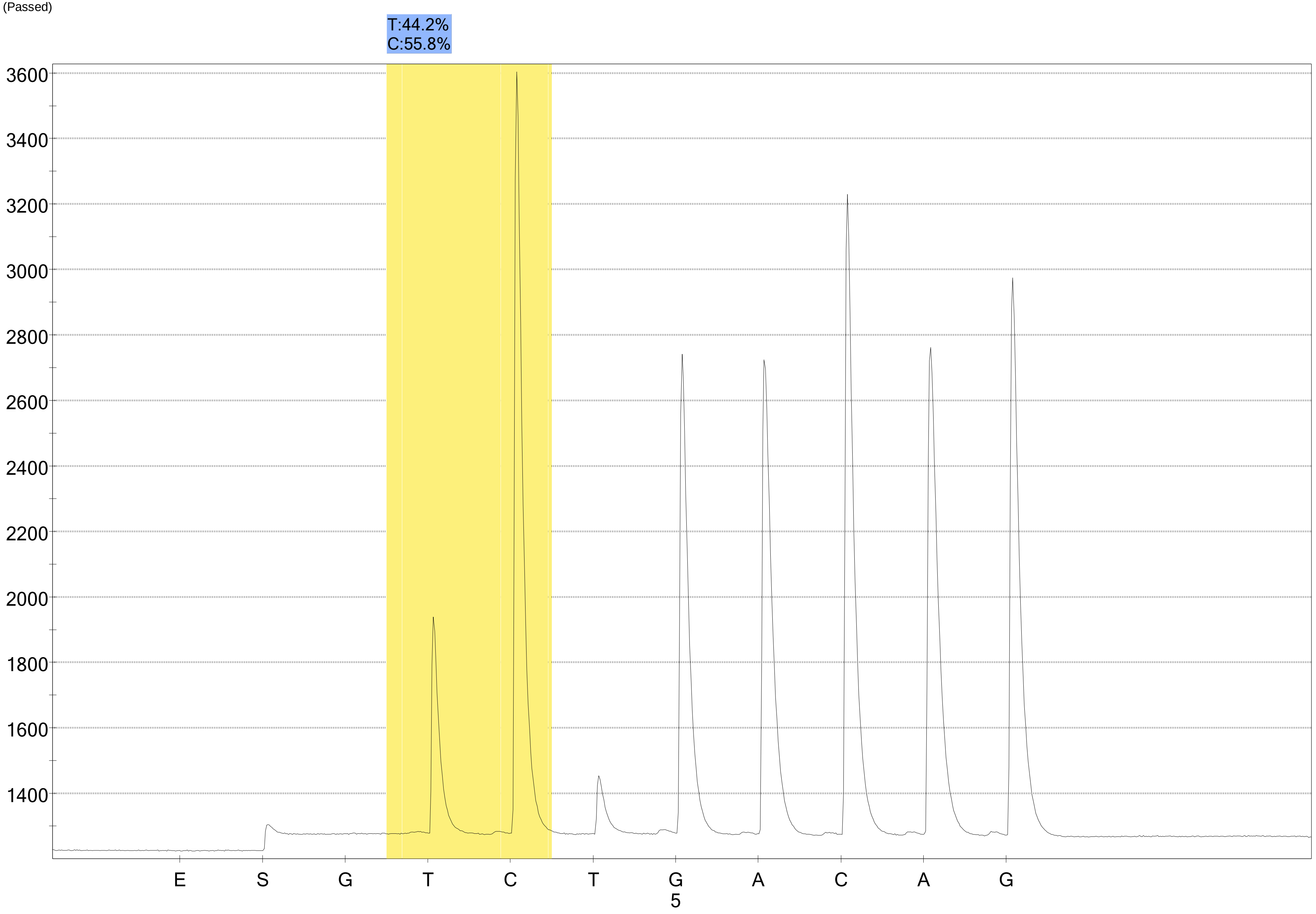

dna - Well D2
Entry: Cald1
1: T: 37.1% / C: 62.9%
(Passed)

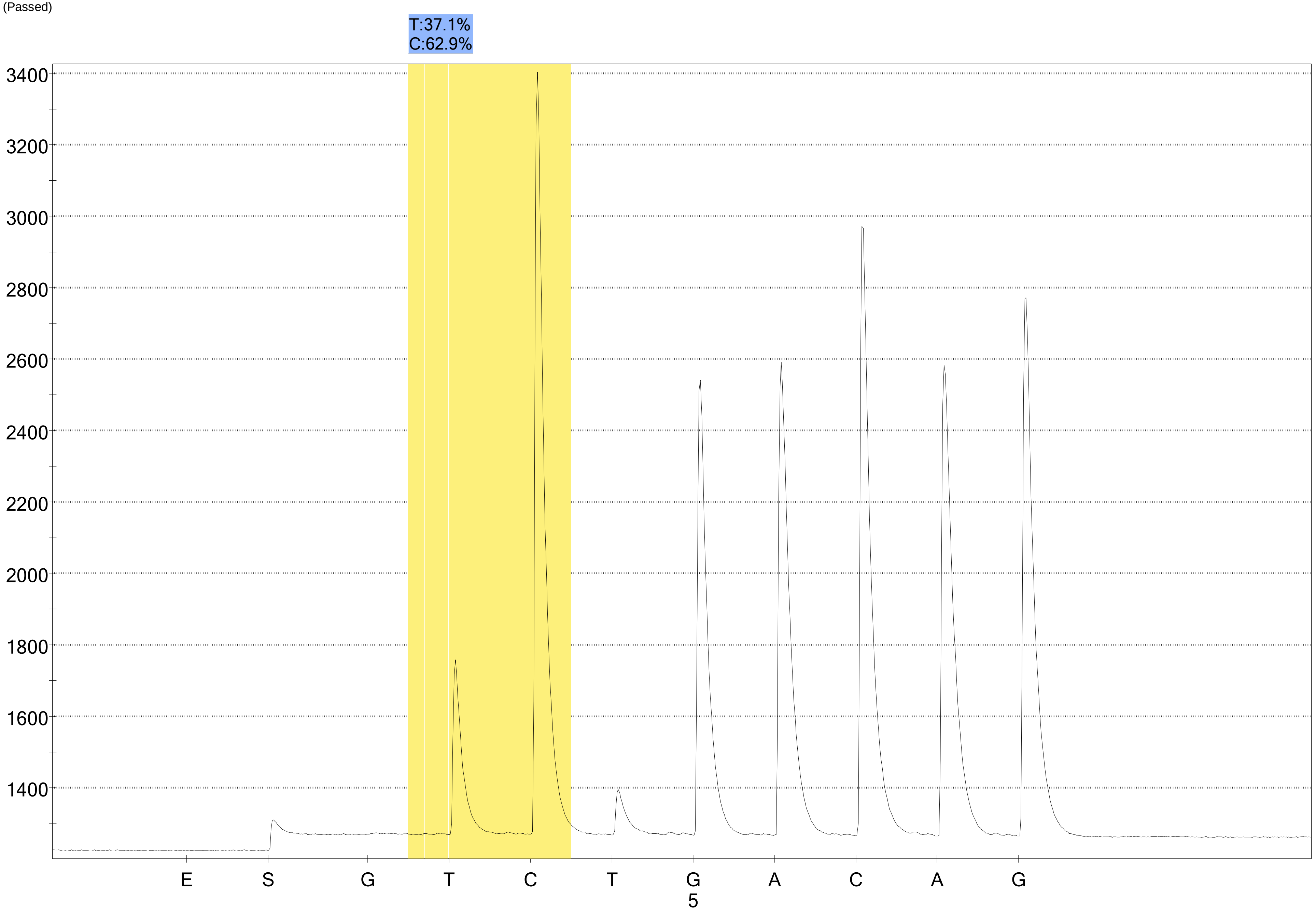

dna - Well D8
Entry: Cald1
1: T: 40.6% / C: 59.4%
(Passed)

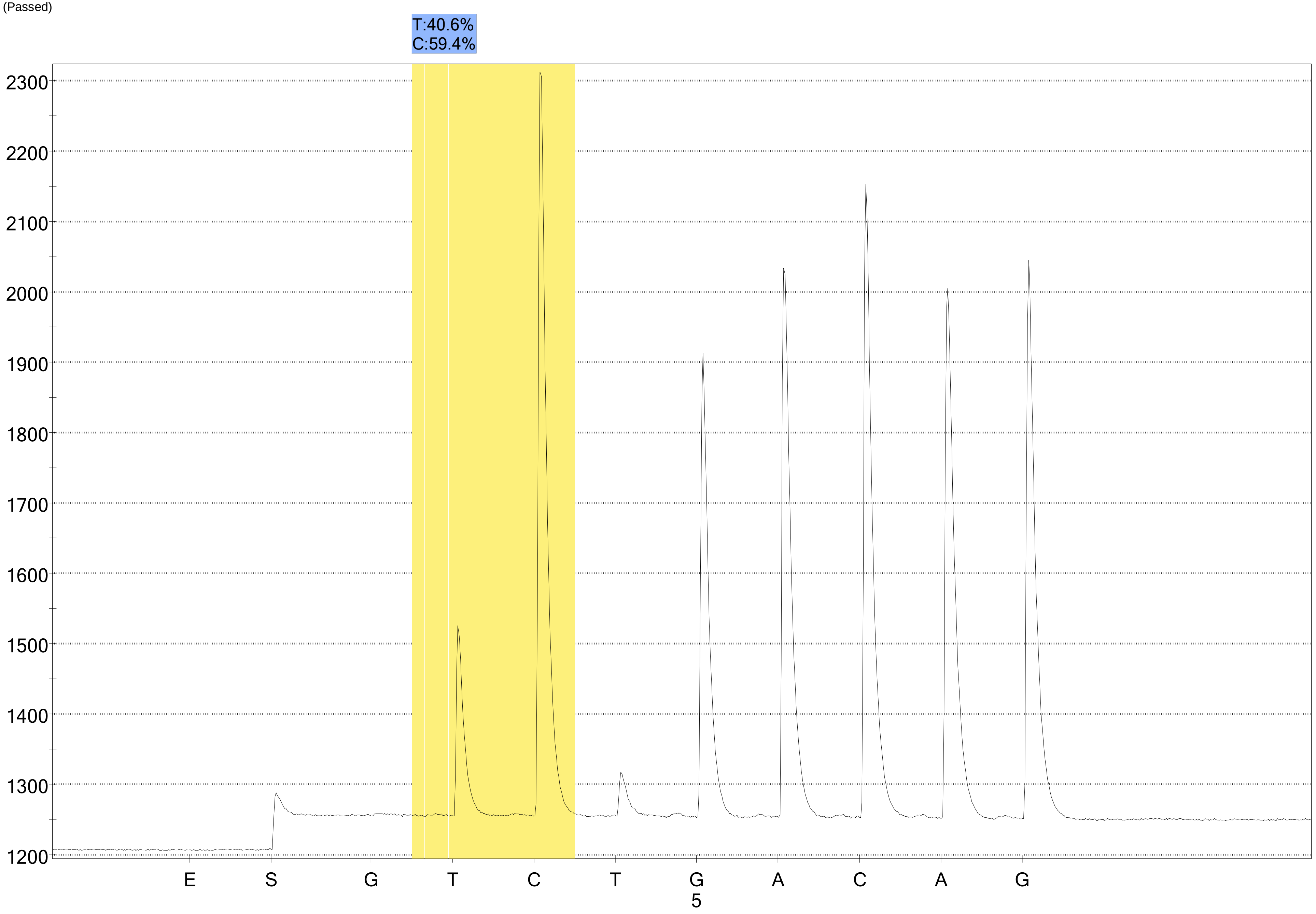

10 uL universal (141+157) - Well B3
Entry: Ddit4
4: G: 59.2% / A: 40.8%
(Passed)

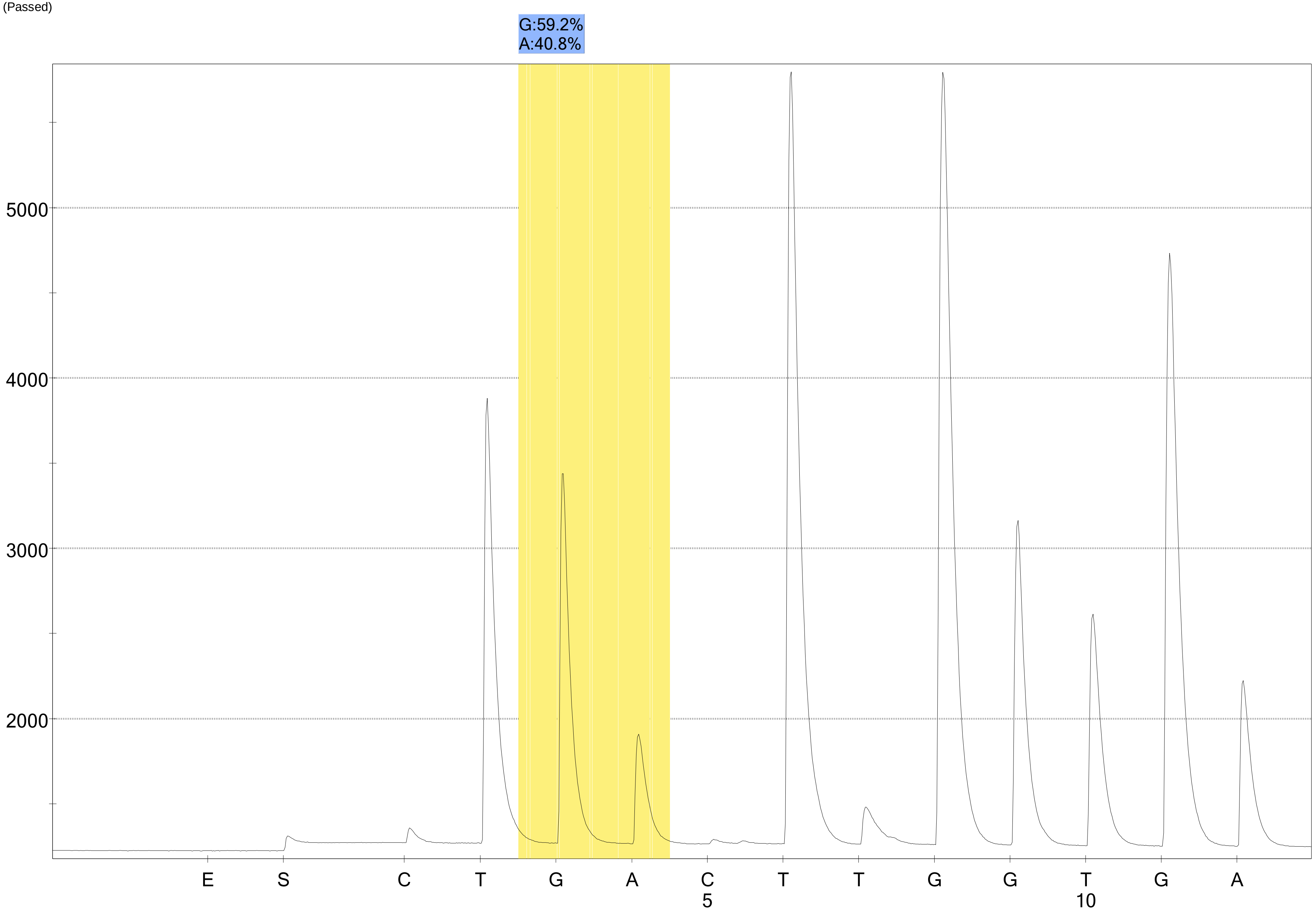

10 uL universal (141+157) - Well B9
Entry: Ddit4
4: G: 52.3% / A: 47.7%
(Passed)

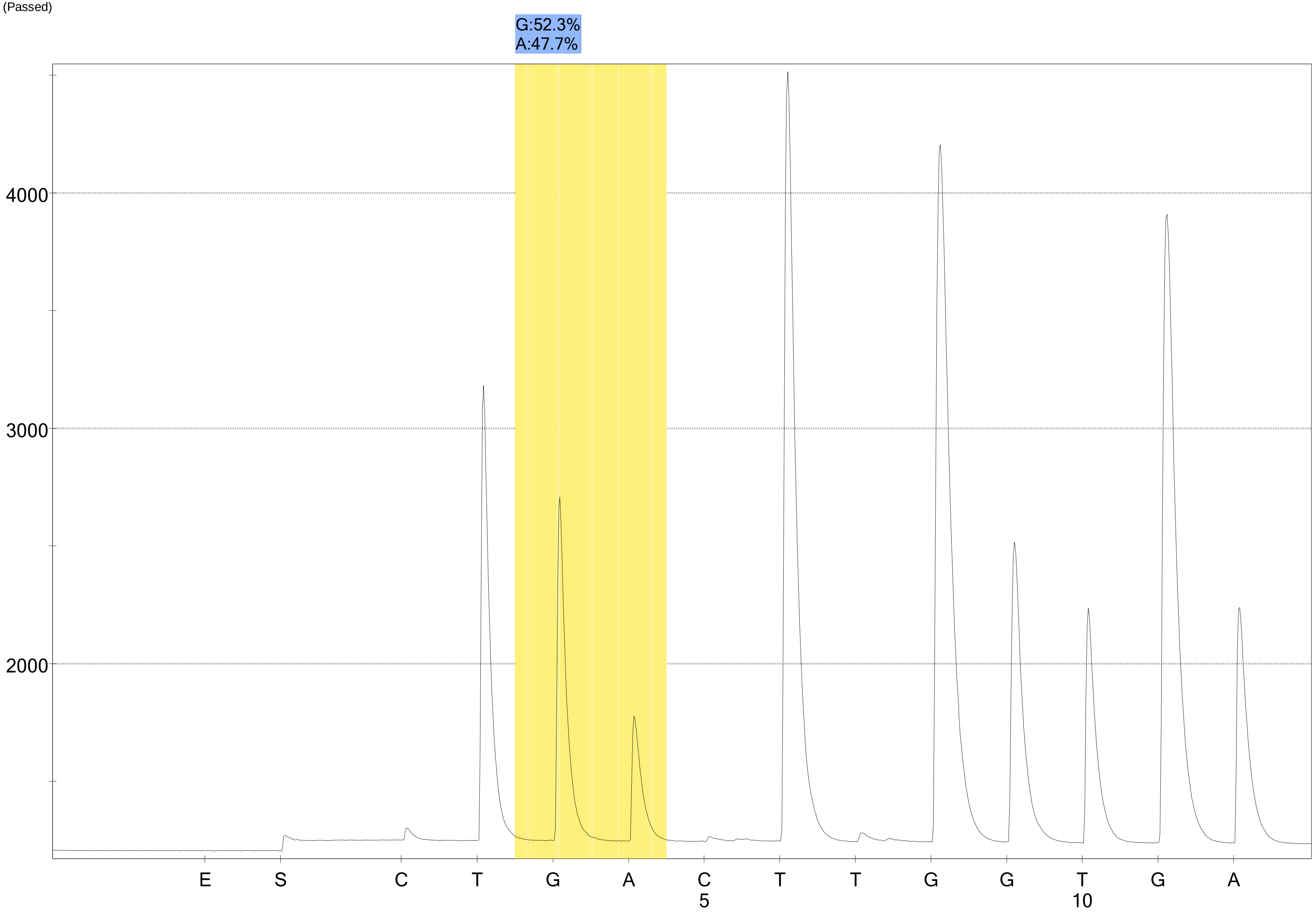

145 - Well B3
Entry: Ddit4
4: G: 59.7% / A: 40.3%
(Passed)

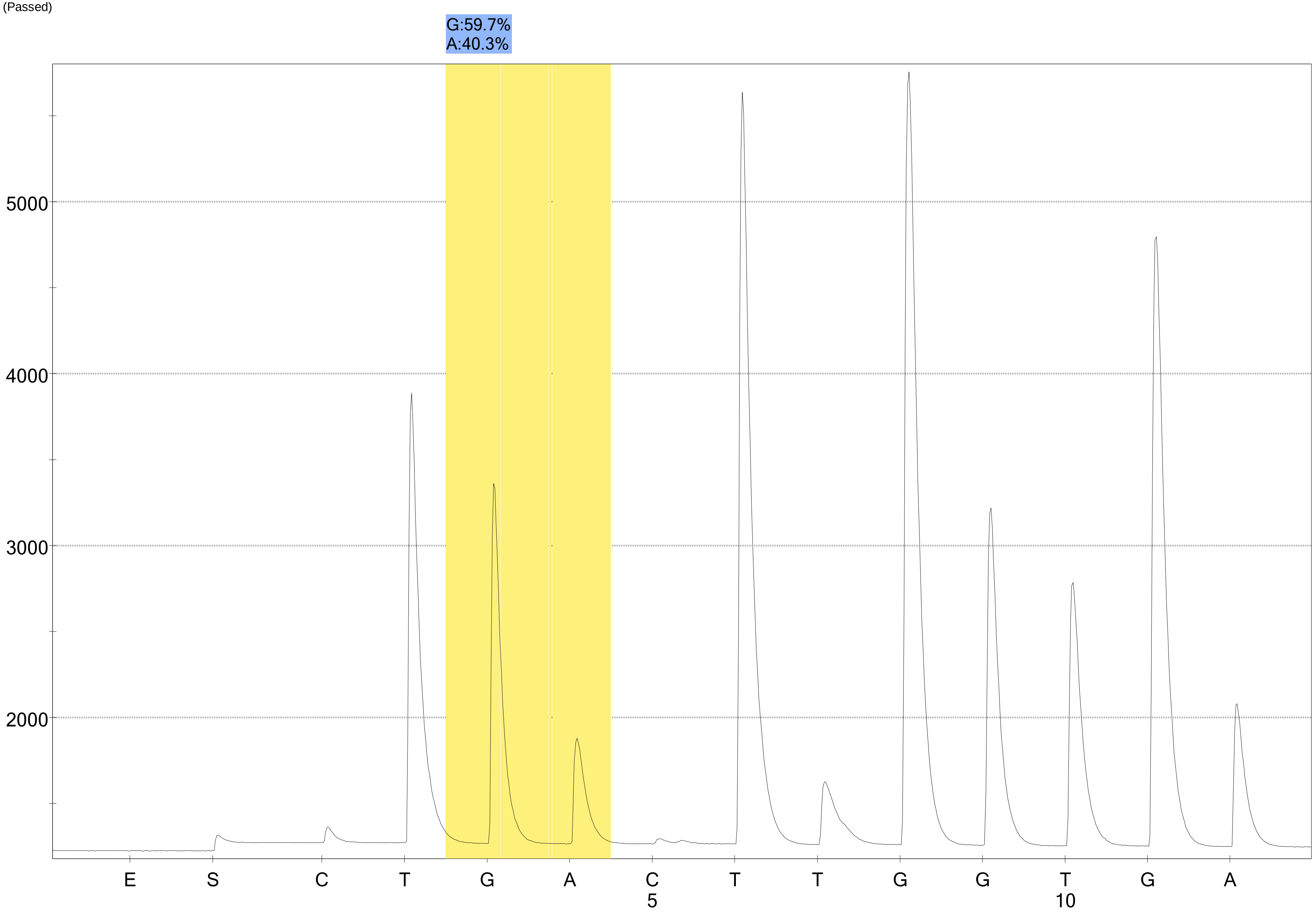

dna - Well B3
Entry: Ddit4
4: G: 46.7% / A: 53.3%
(Passed)

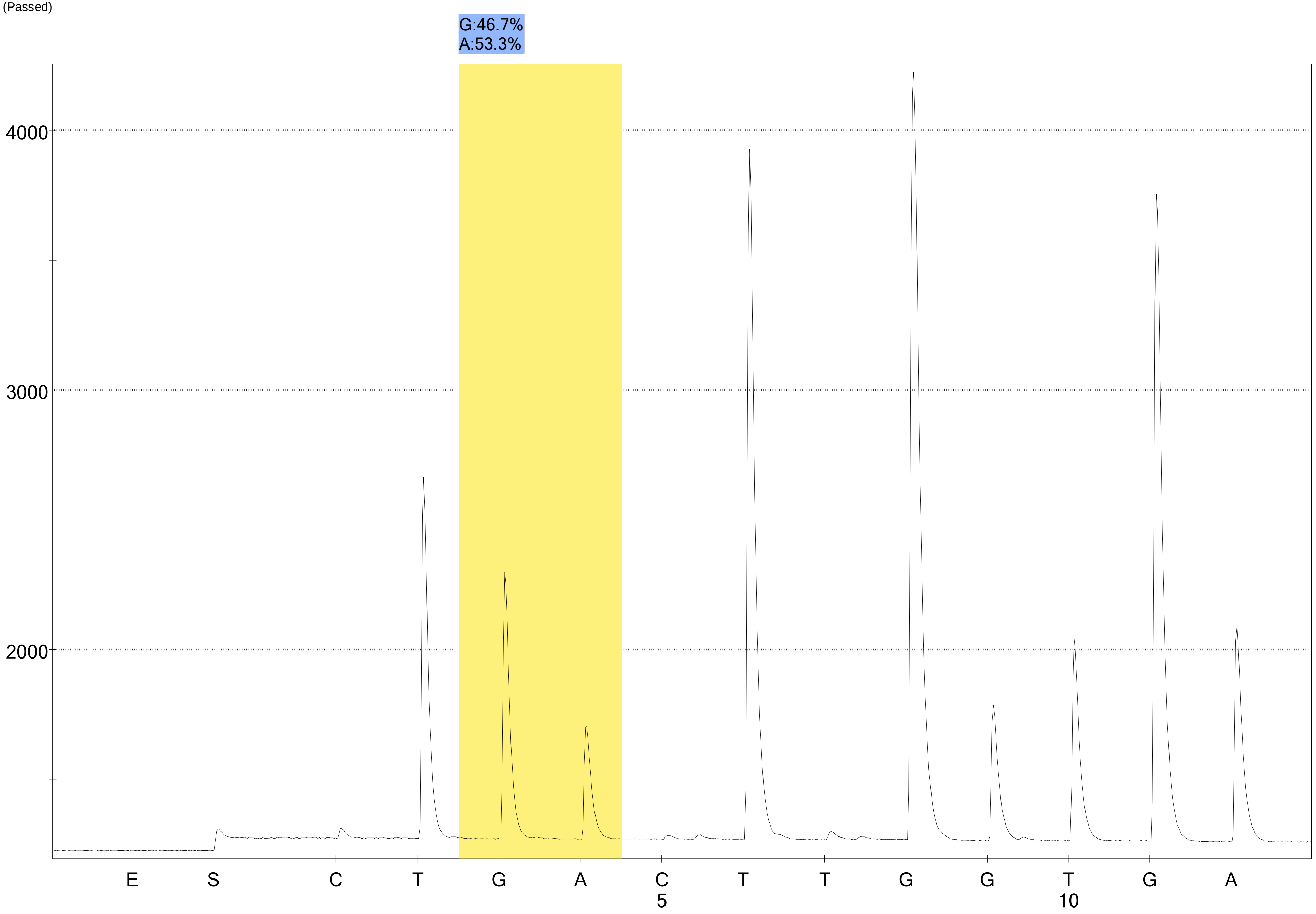

dna - Well B9
Entry: Ddit4
4: G: 40.5% / A: 59.5%
(Passed)

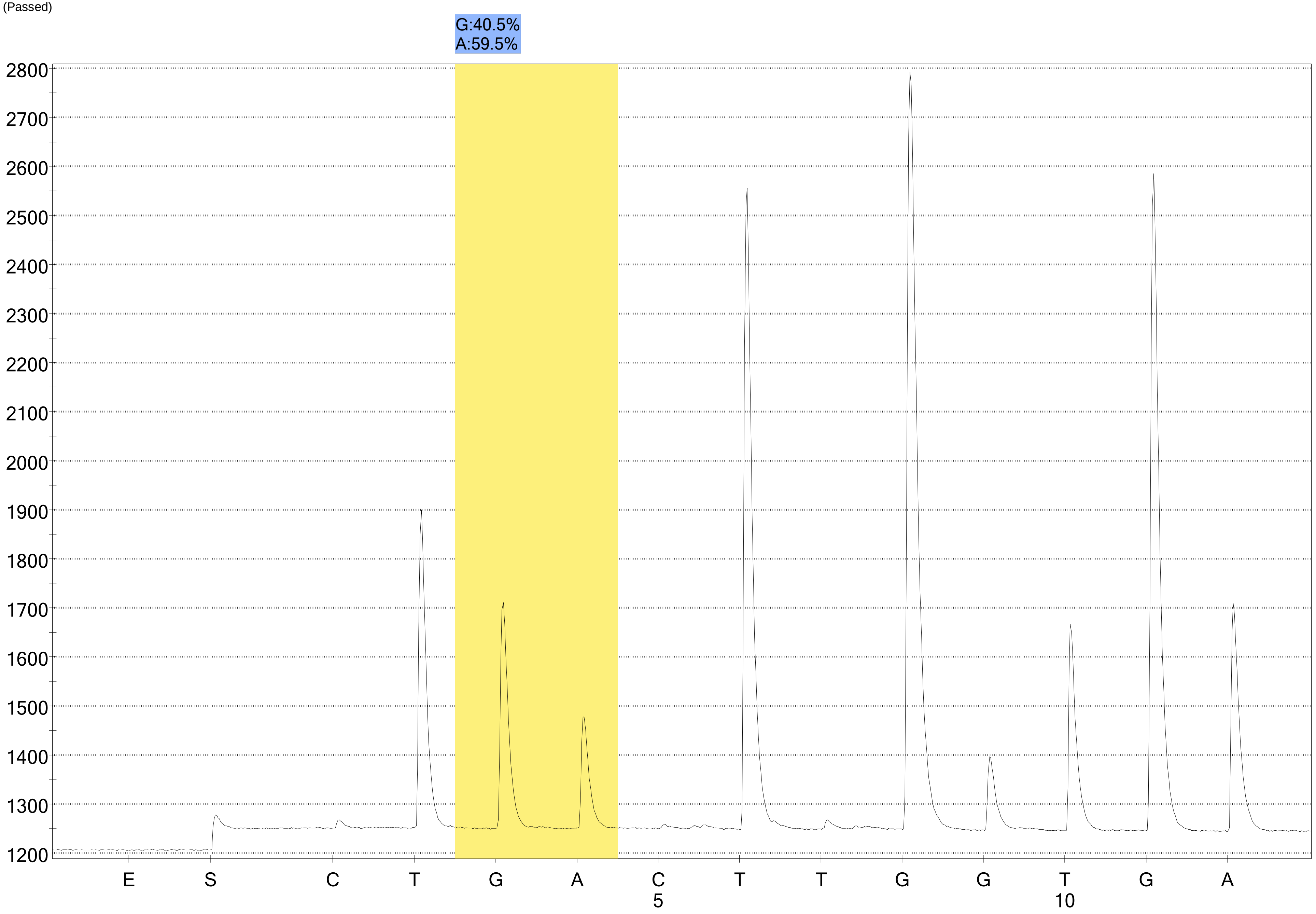

10 uL universal (141+157) - Well D3
Entry: Dvl3
1: T: 65.7% / C: 34.3%
(Passed)

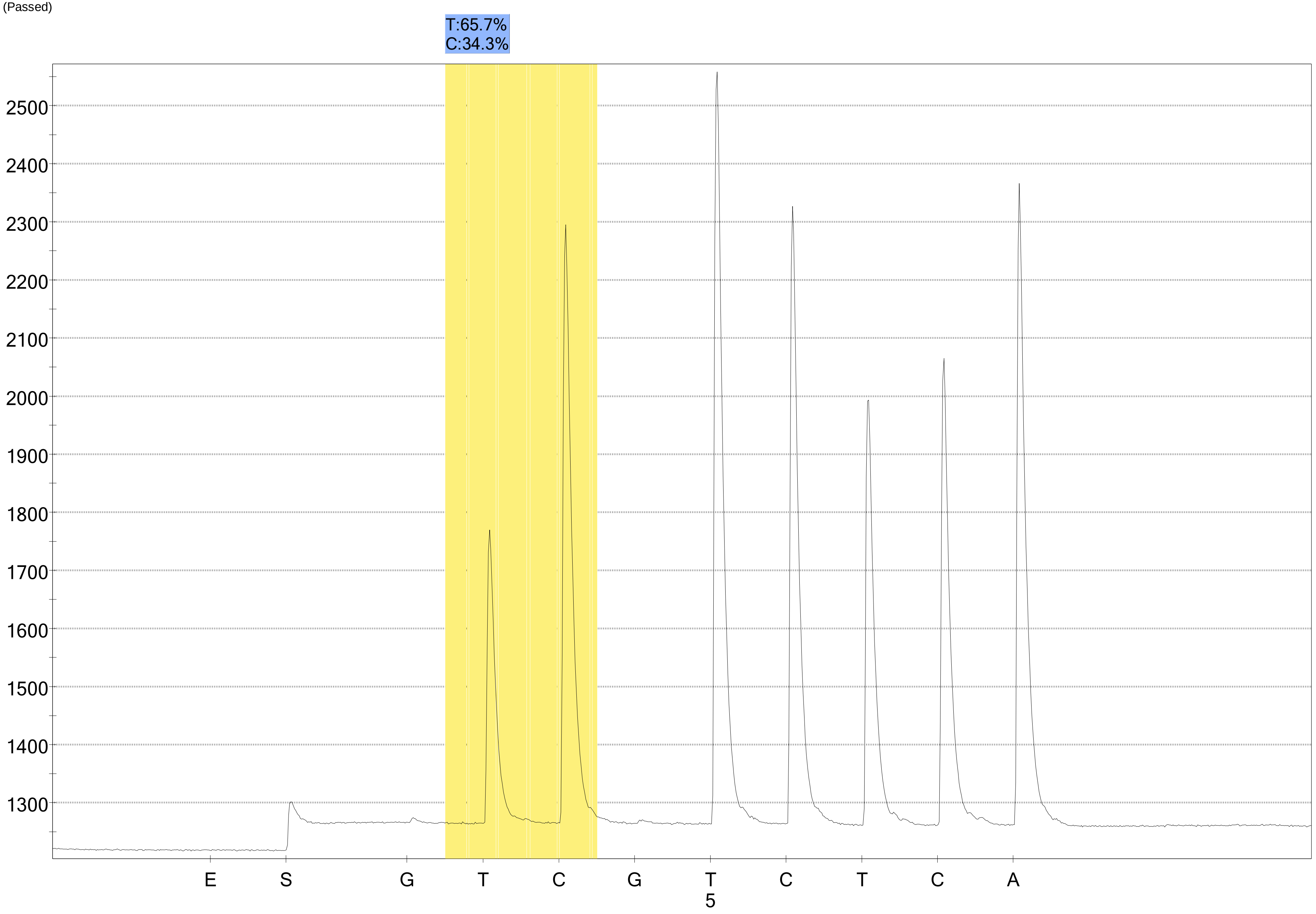

10 uL universal (141+157) - Well D9
Entry: Dvl3
1: T: 64.1% / C: 35.9%
(Passed)

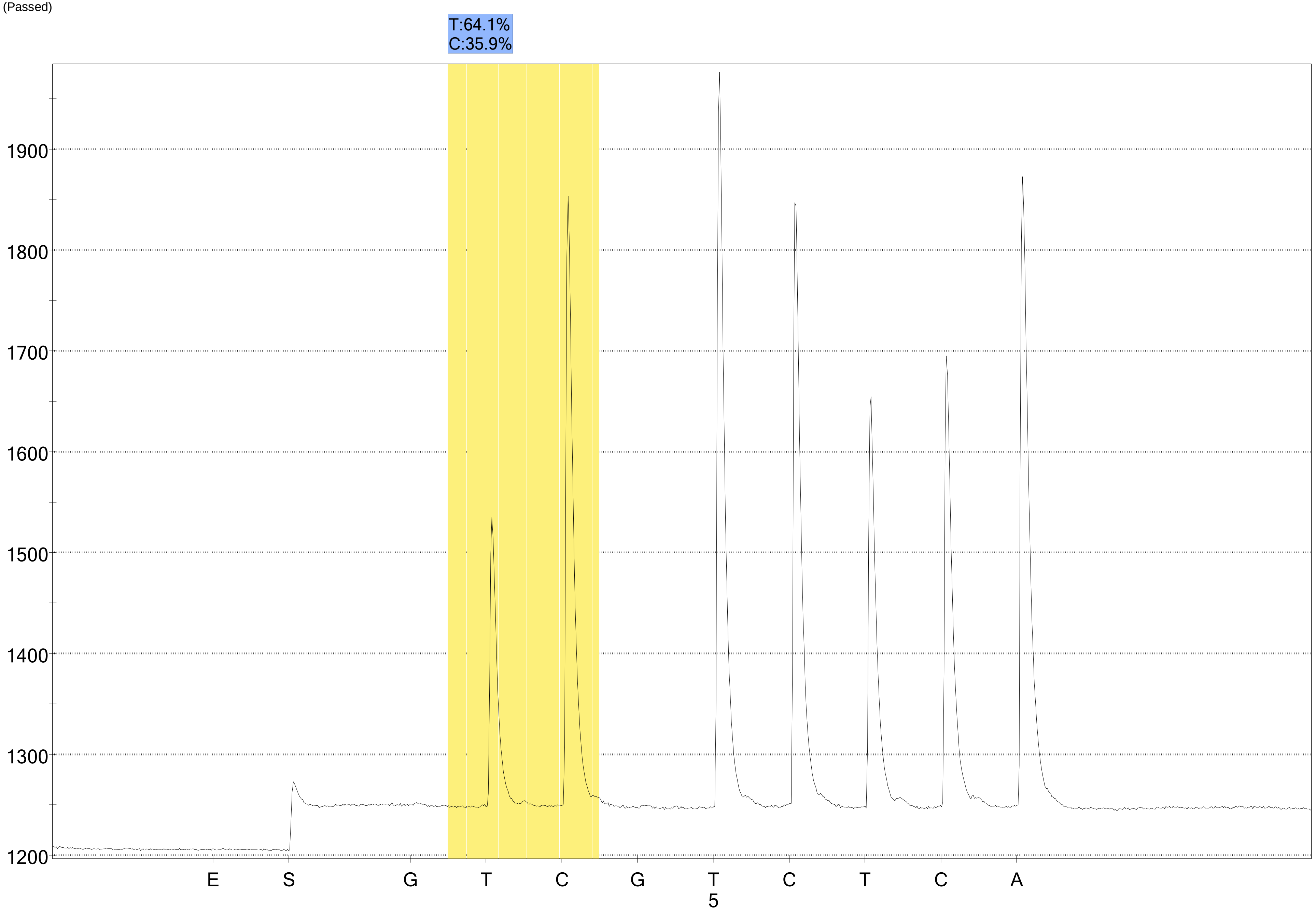

145 - Well D3
Entry: Dvl3
1: T: 75.8% / C: 24.2%
(Passed)

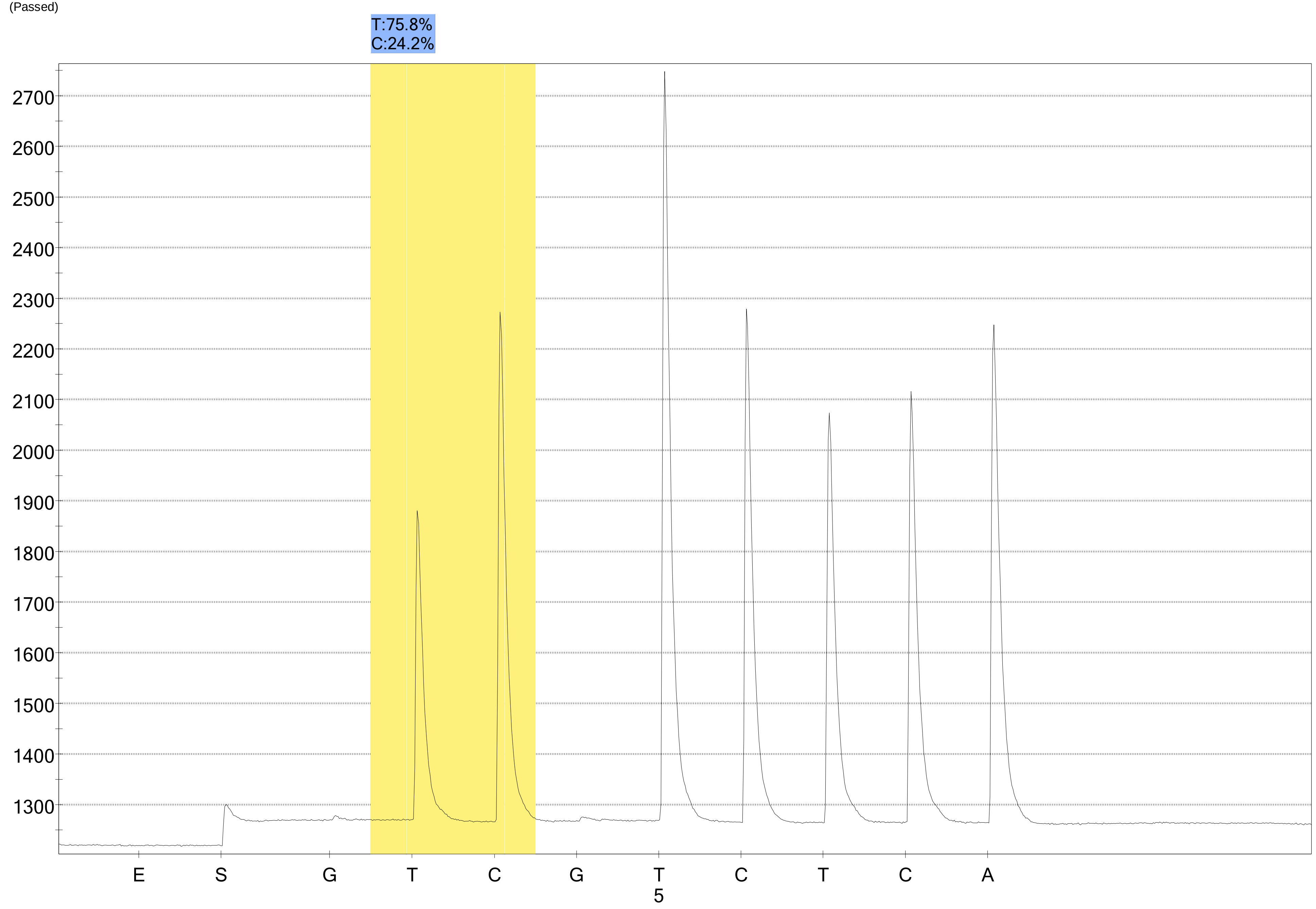

dna - Well D3
Entry: Dvl3
1: T: 62.5% / C: 37.5%
(Passed)

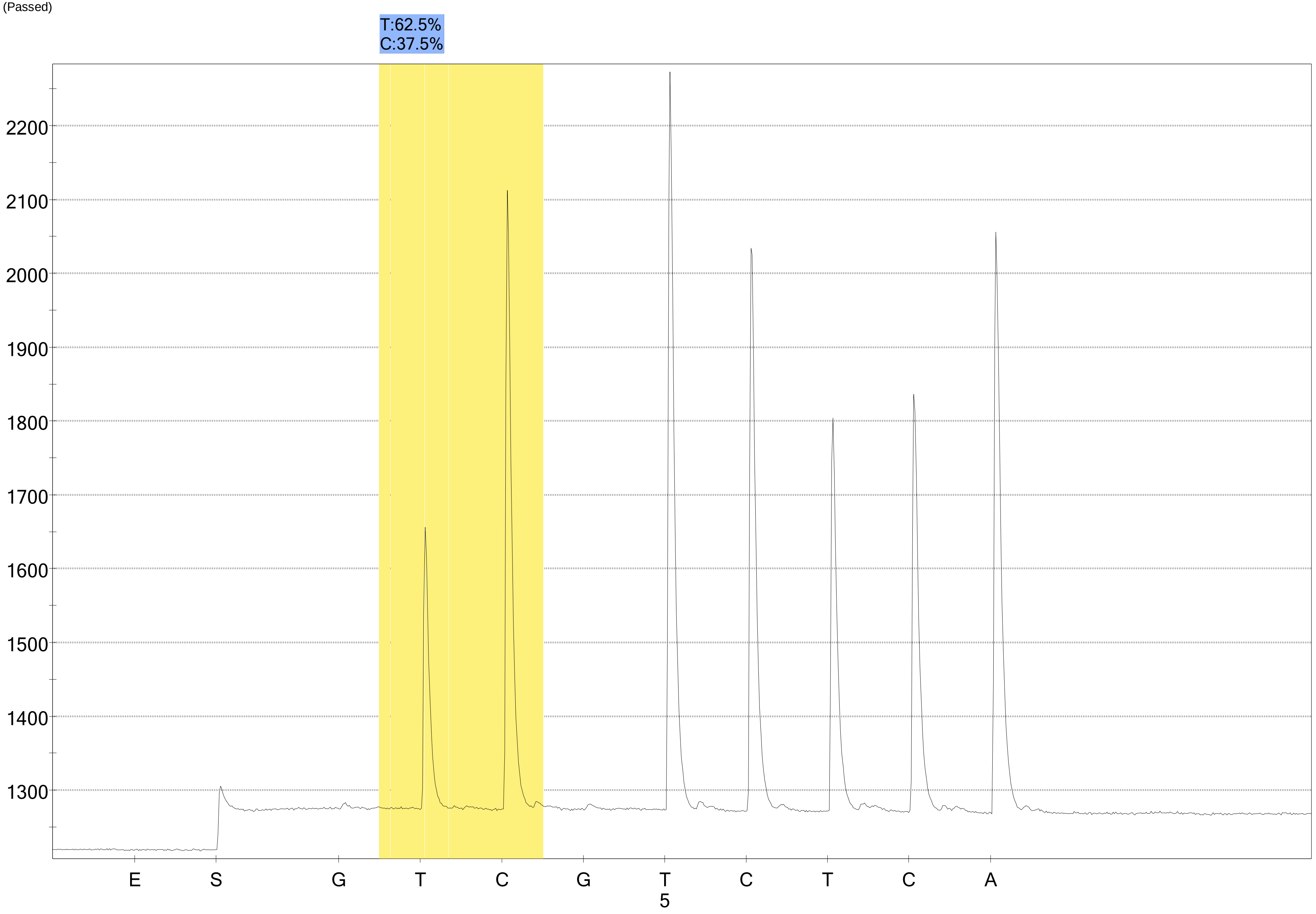

dna - Well D9
Entry: Dvl3
1: T: 64.7% / C: 35.3%
(Passed)

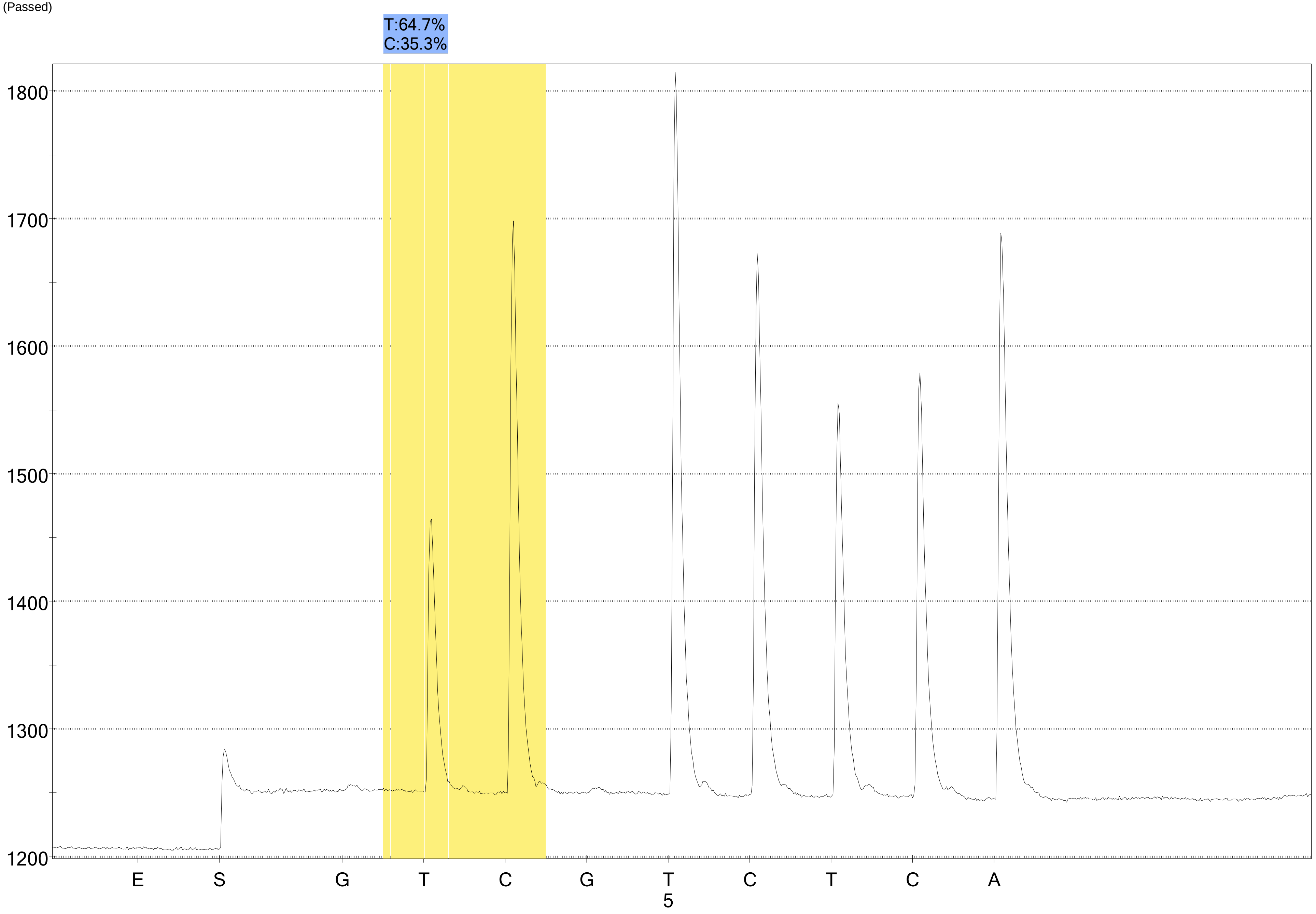

10 uL universal (141+157) - Well B4
Entry: Ina
1: C: 63.8% / T: 36.2%
(Passed)

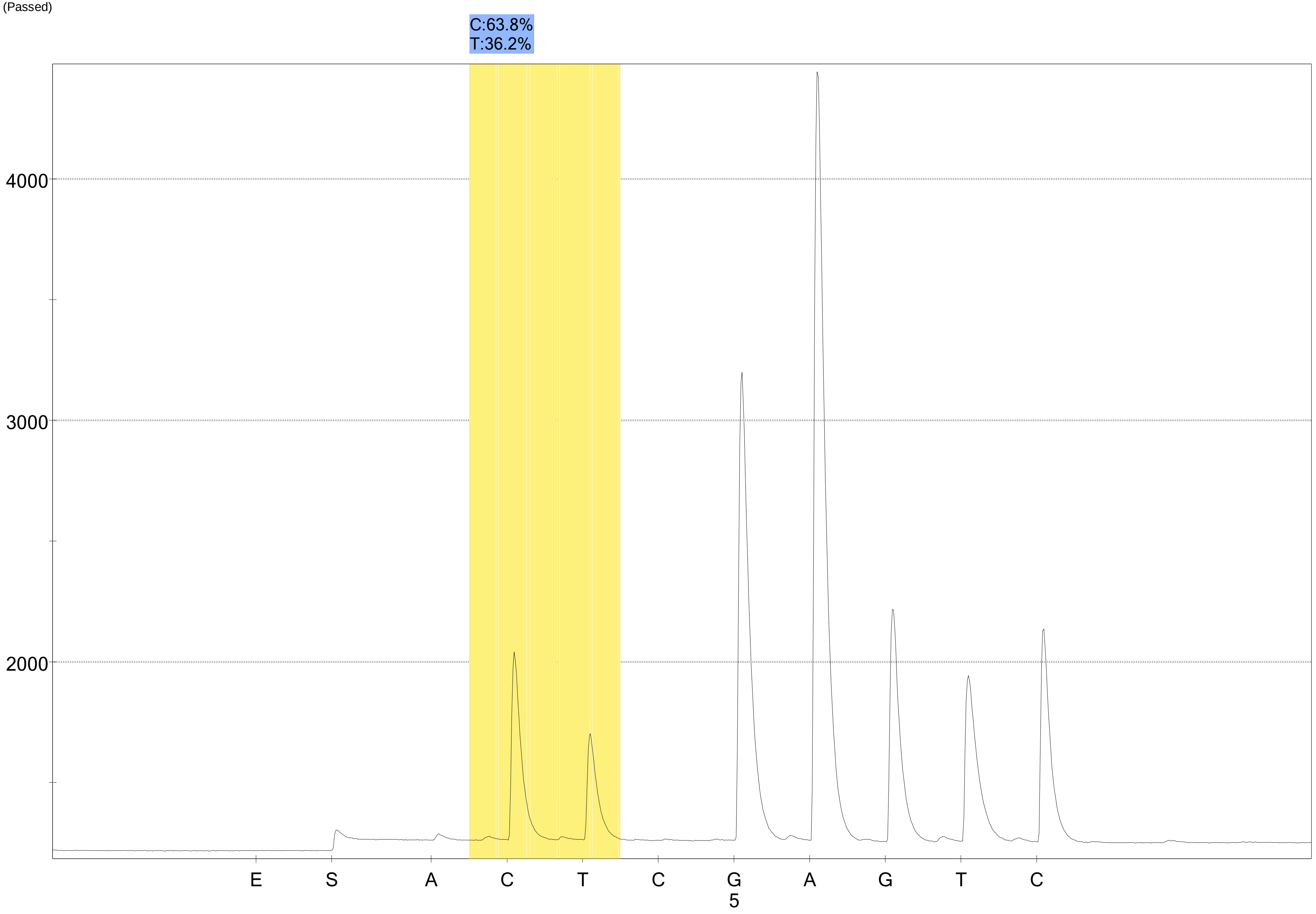

10 uL universal (141+157) - Well B10
Entry: Ina
1: C: 60.6% / T: 39.4%
(Passed)

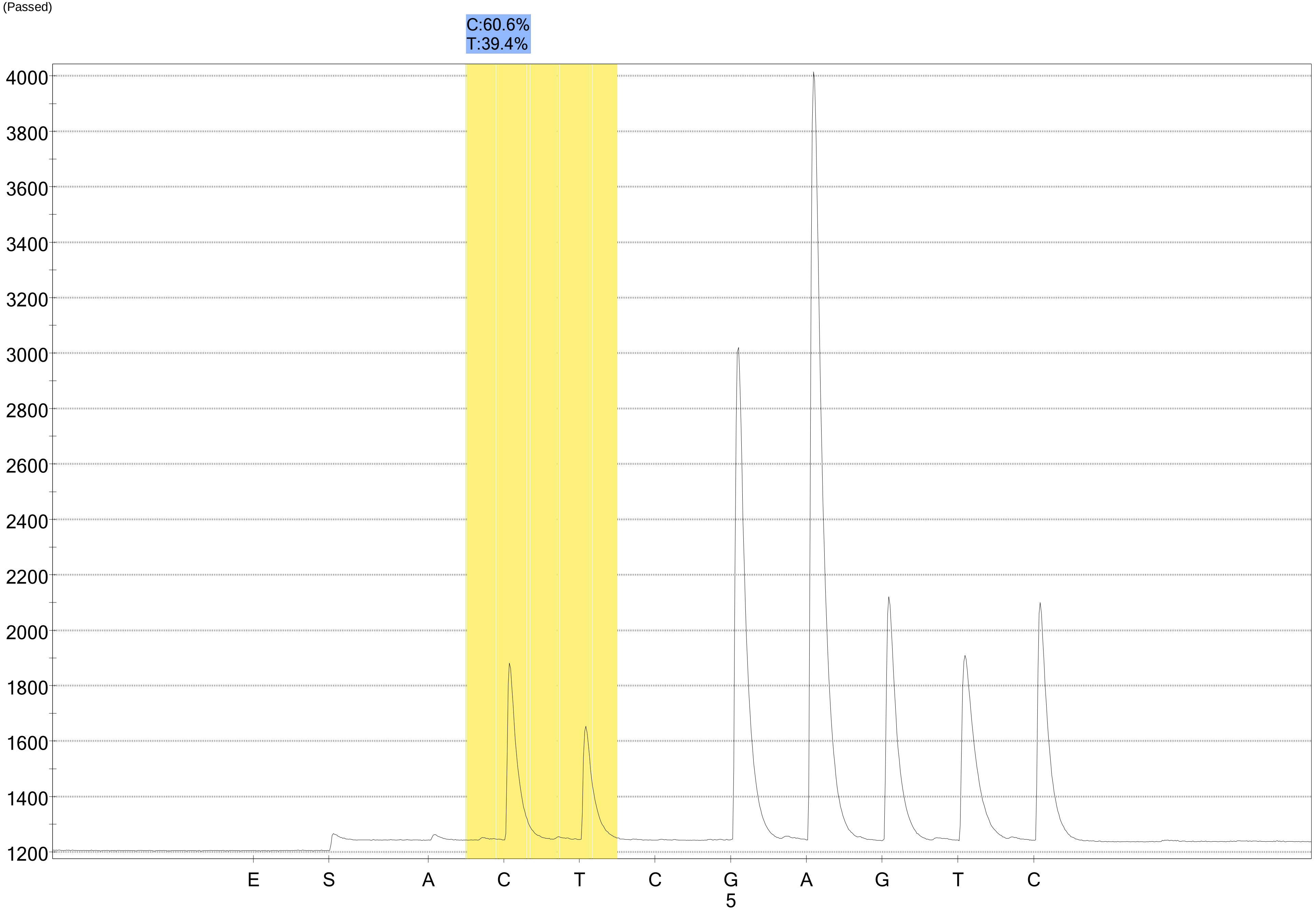

145 - Well B4
Entry: Ina
1: C: 64.6% / T: 35.4%
(Passed)

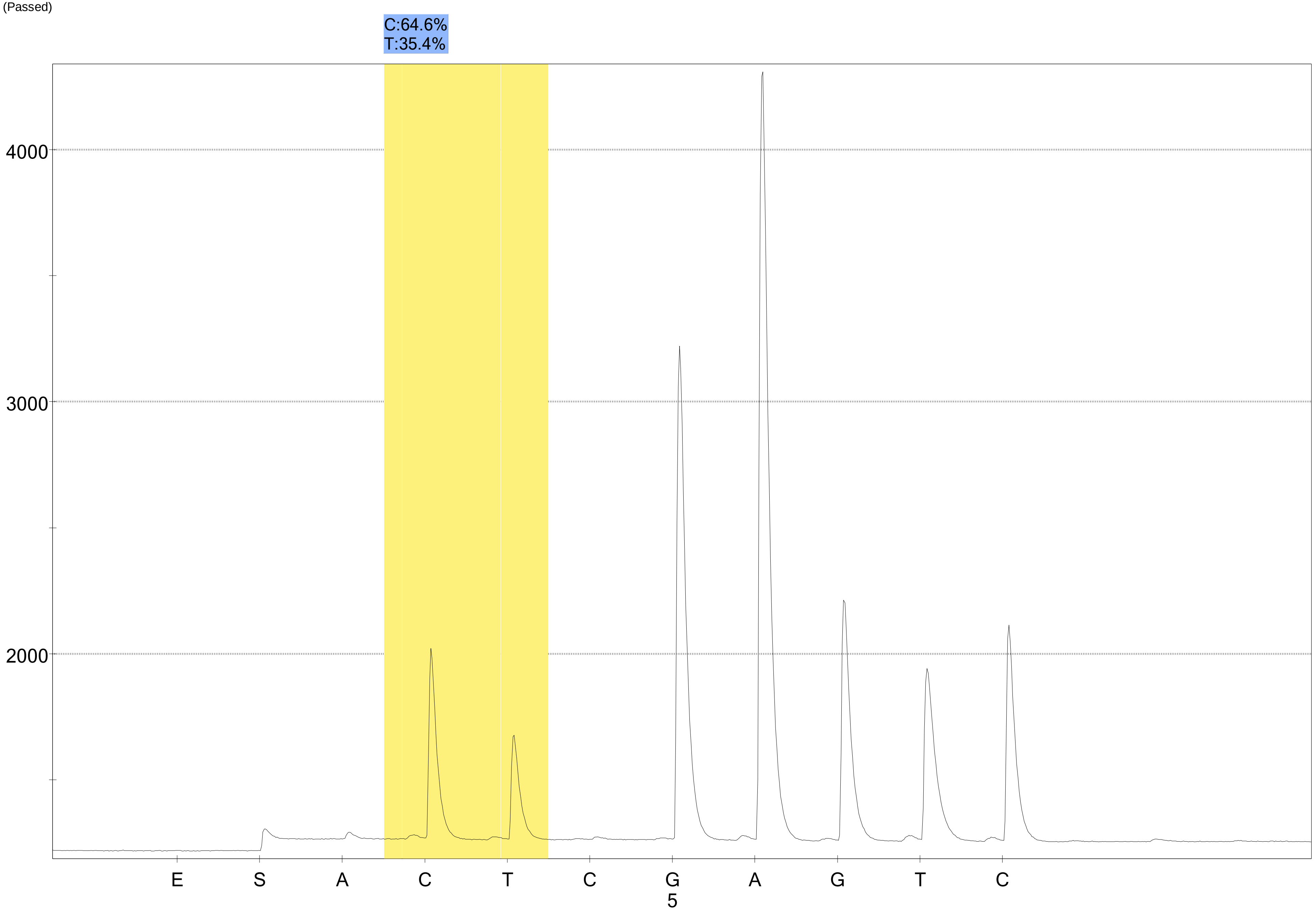

dna - Well B4
Entry: Ina
1: C: 58.9% / T: 41.1%
(Passed)

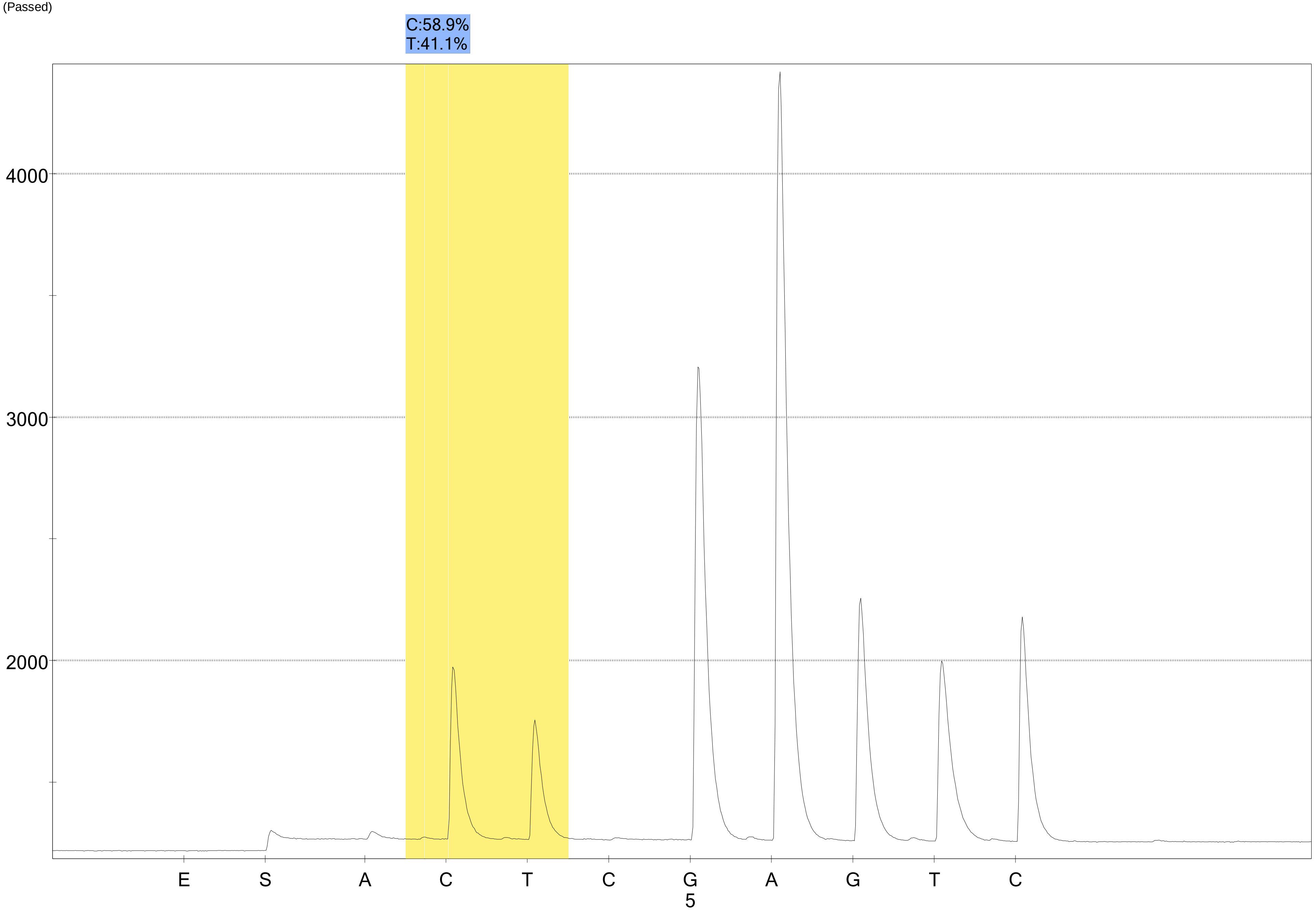

dna - Well B10
Entry: Ina
1: C: 59.8% / T: 40.2%
(Passed)

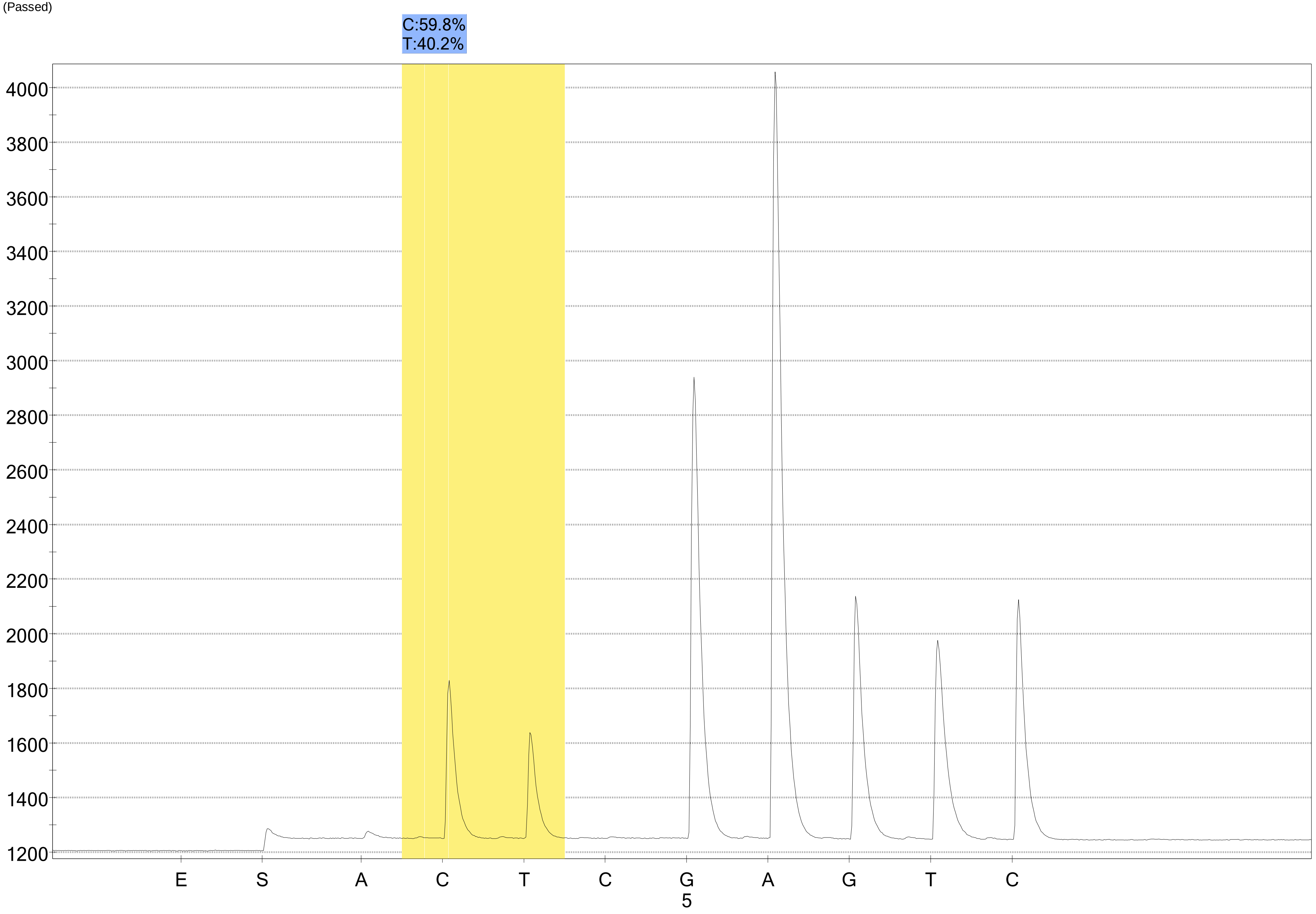

10 uL universal (141+157) - Well A1
Entry: 1700027N10Rik
1: C: 60.5% / G: 39.5%
(Check)

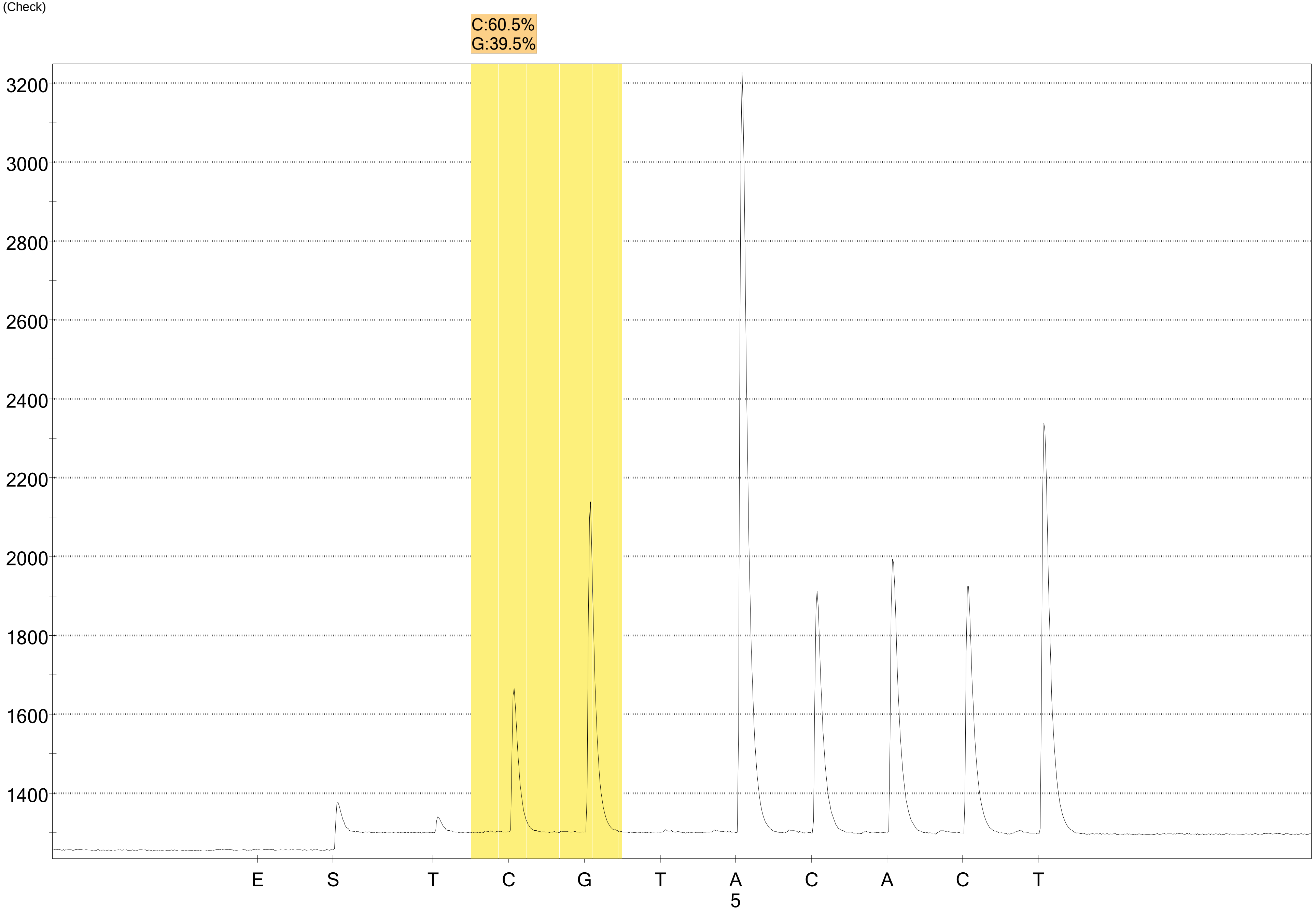

10 uL universal (141+157) - Well A7
Entry: 1700027N10Rik
1: C: 56.6% / G: 43.4%
(Failed)

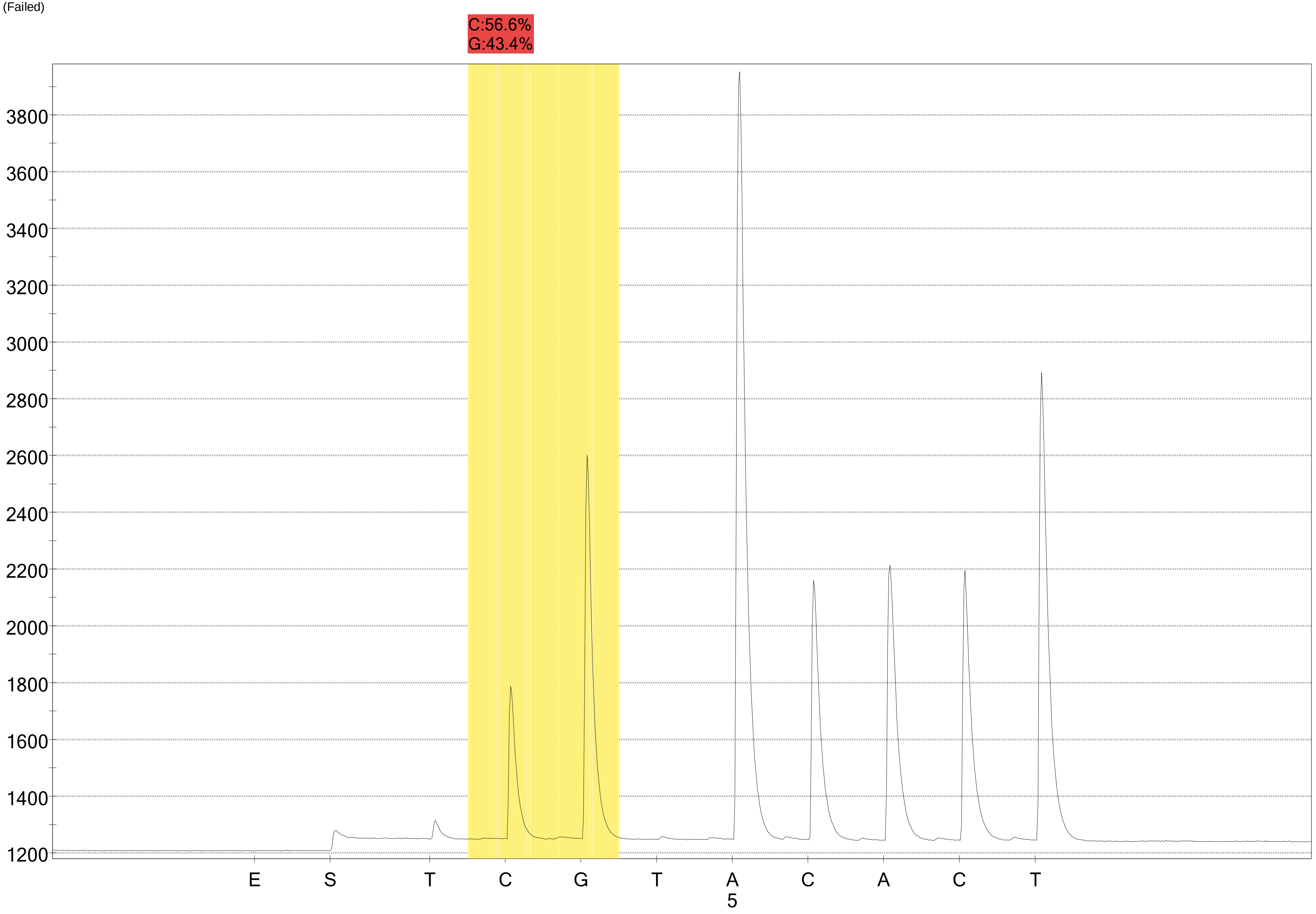

145 - Well A1
Entry: 1700027N10Rik
1: C: 59.1% / G: 40.9%
(Check)

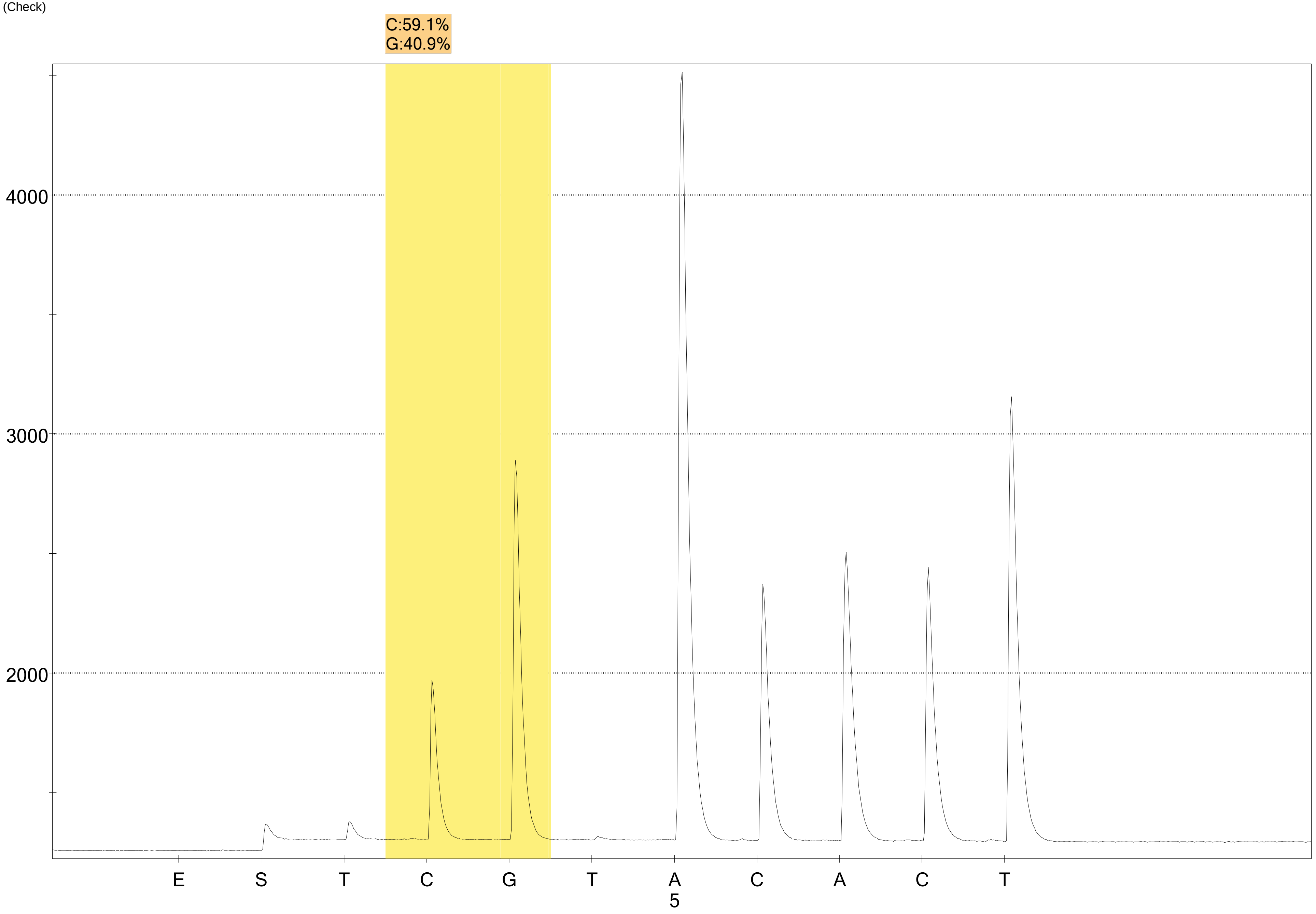

dna - Well A1
Entry: 1700027N10Rik
1: C: 55.2% / G: 44.8%
(Failed)

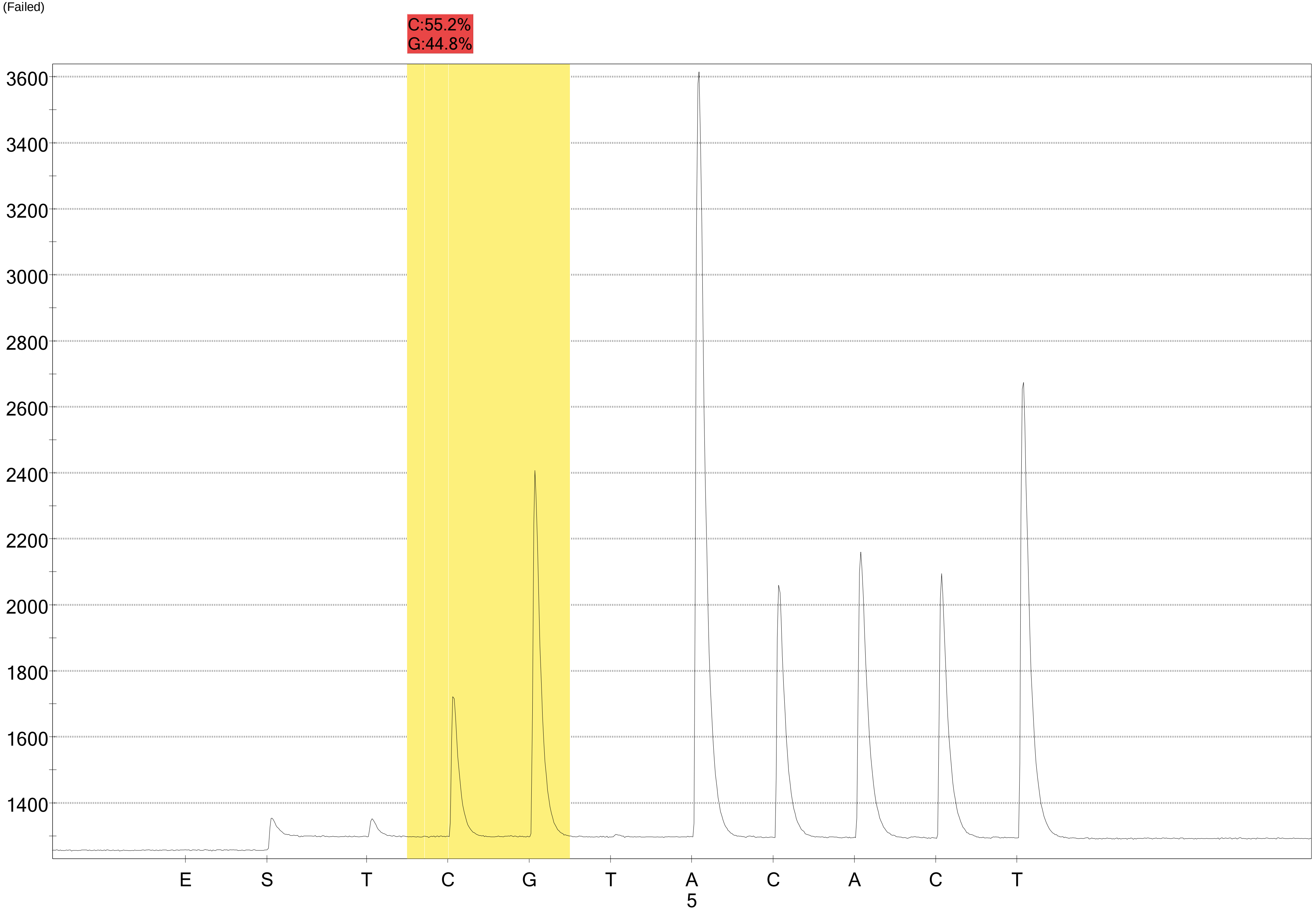

dna - Well A7
Entry: 1700027N10Rik
1: C: 58.9% / G: 41.1%
(Passed)

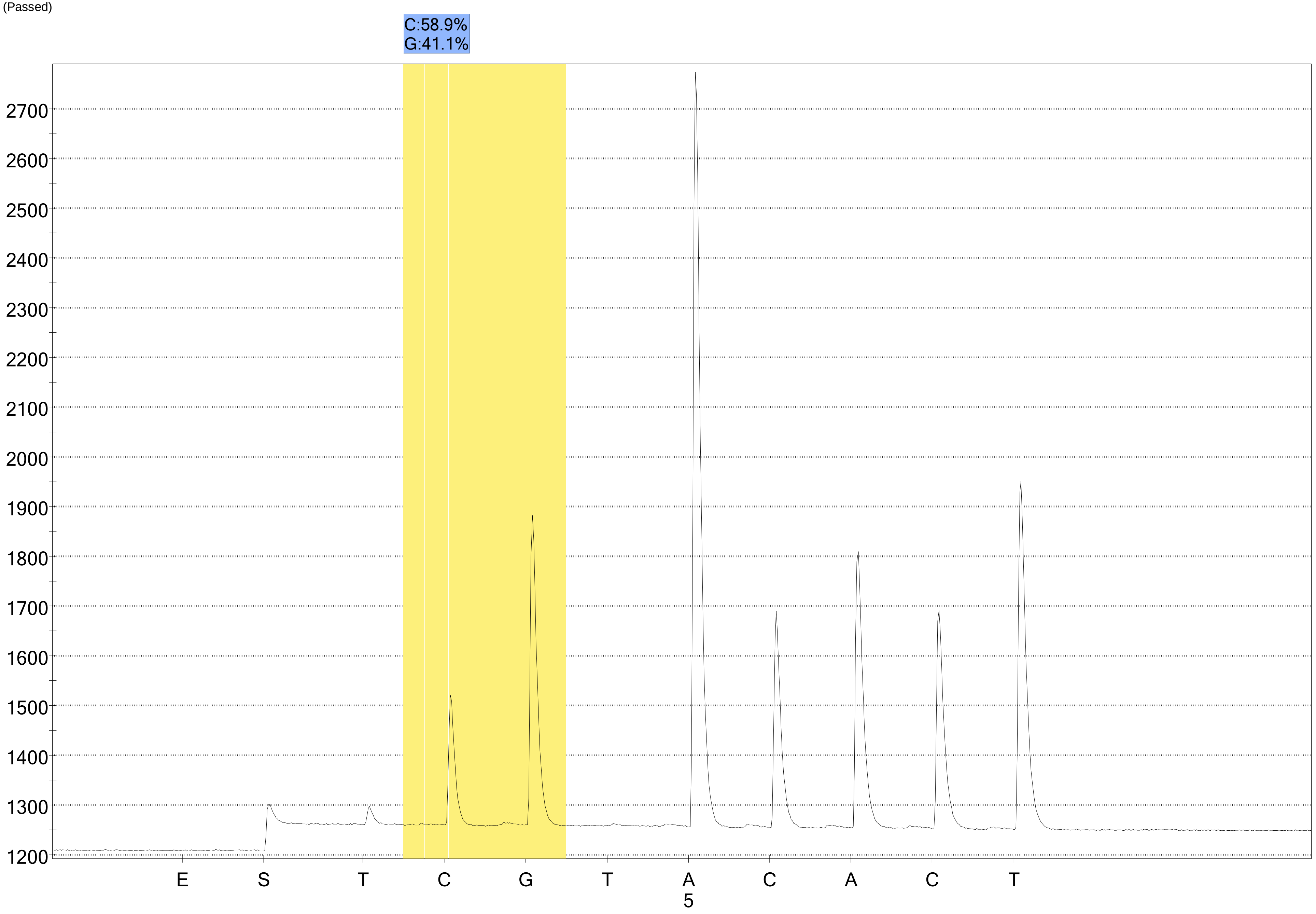

10 uL universal (141+157) - Well E5
Entry: Pitpnm2
1: C: 44.3% / T: 55.7%
(Passed)

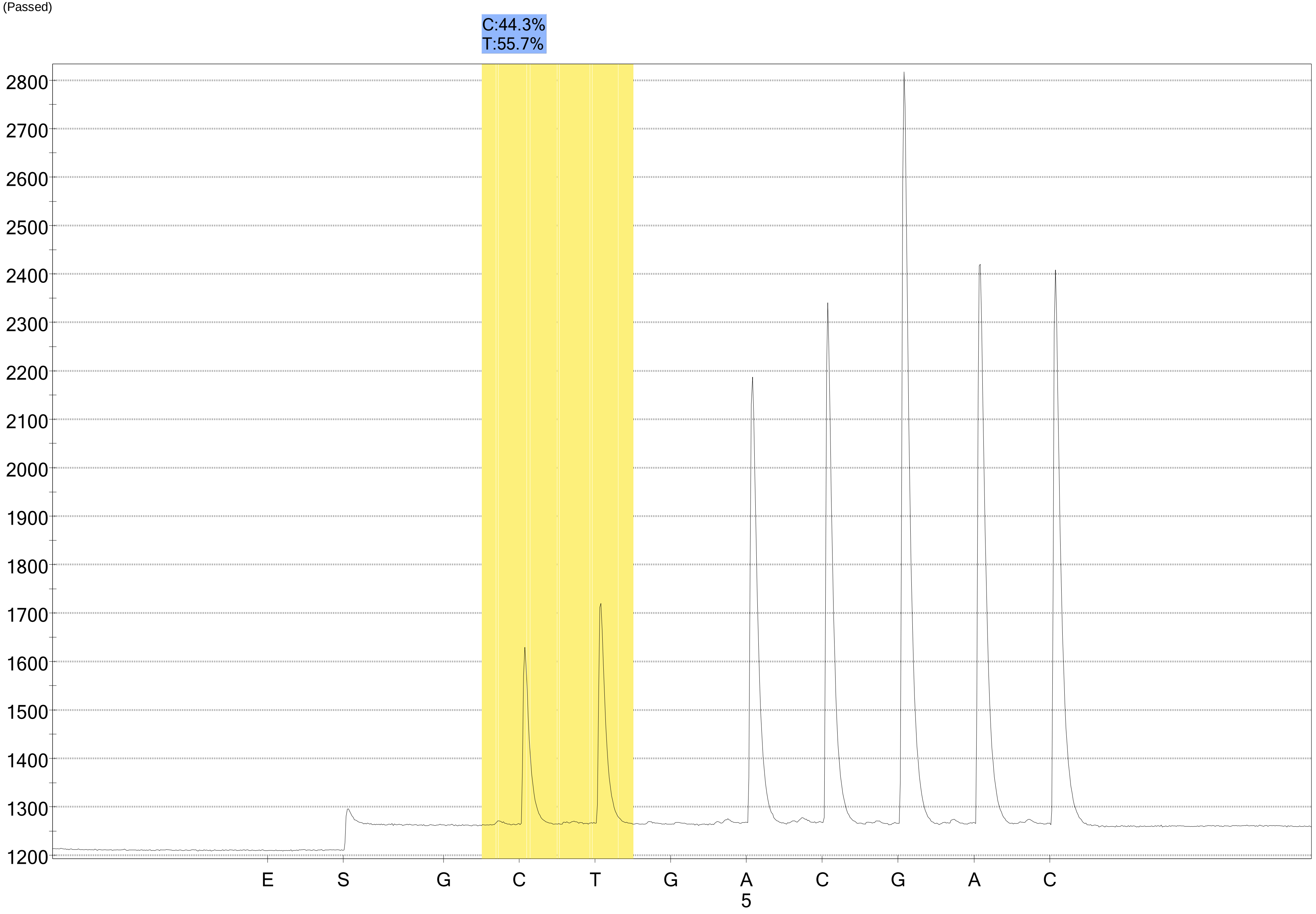

10 uL universal (141+157) - Well E11
Entry: Pitpnm2
1: C: 38.6% / T: 61.4%
(Passed)

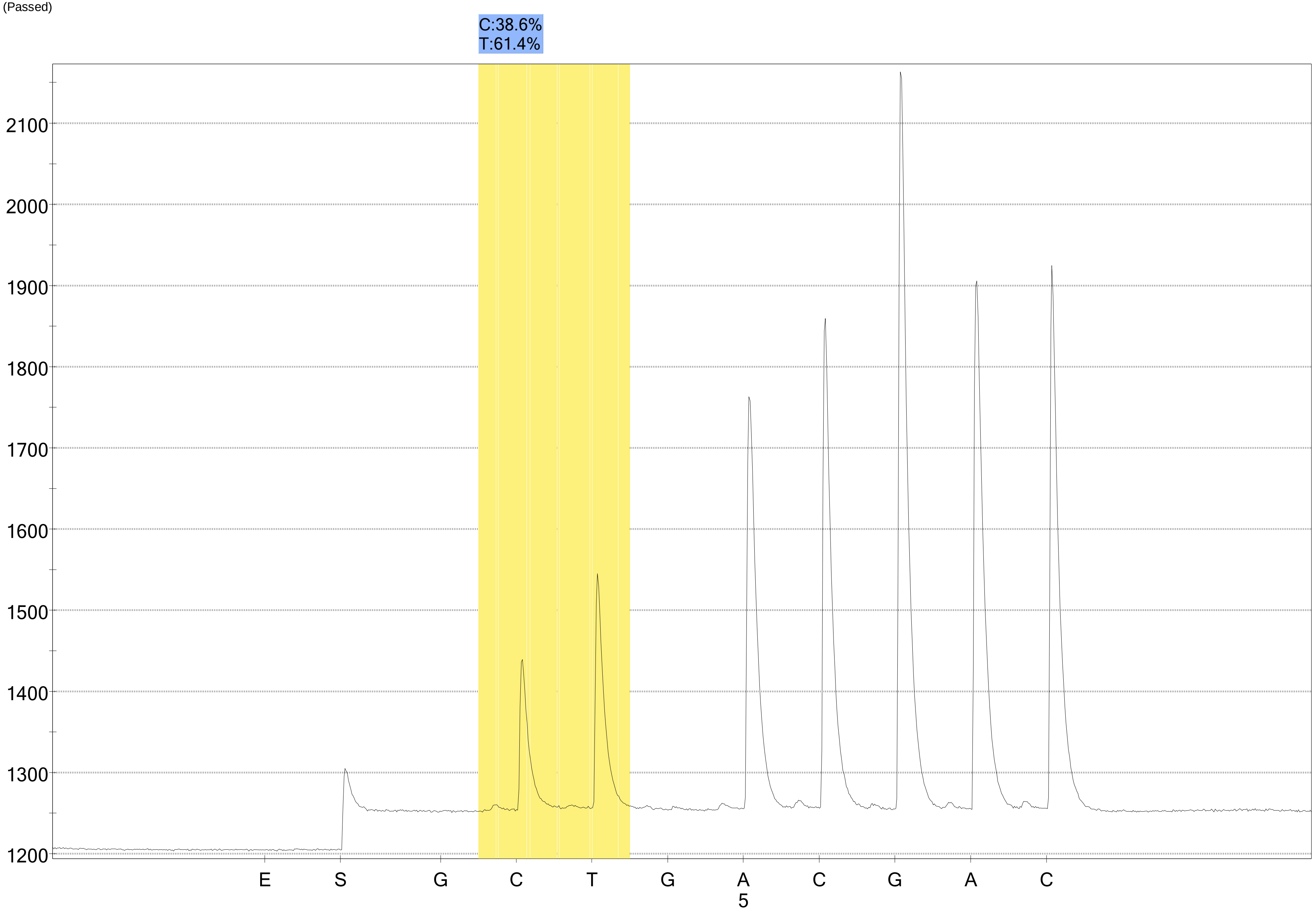

145 - Well E5
Entry: Pitpnm2
1: C: 41.3% / T: 58.7%
(Passed)

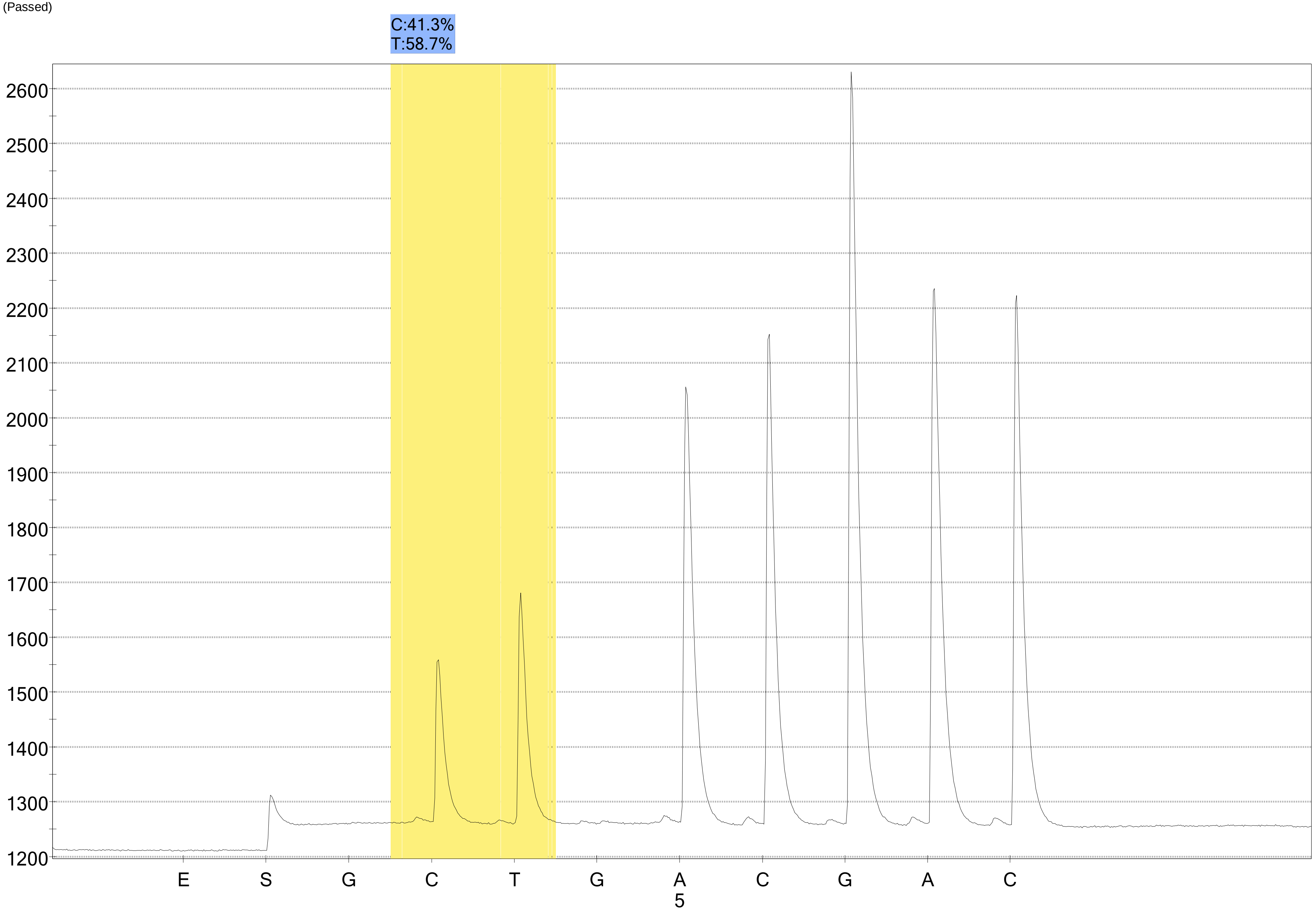

dna - Well E5
Entry: Pitpnm2
1: C: 40.2% / T: 59.8%
(Passed)

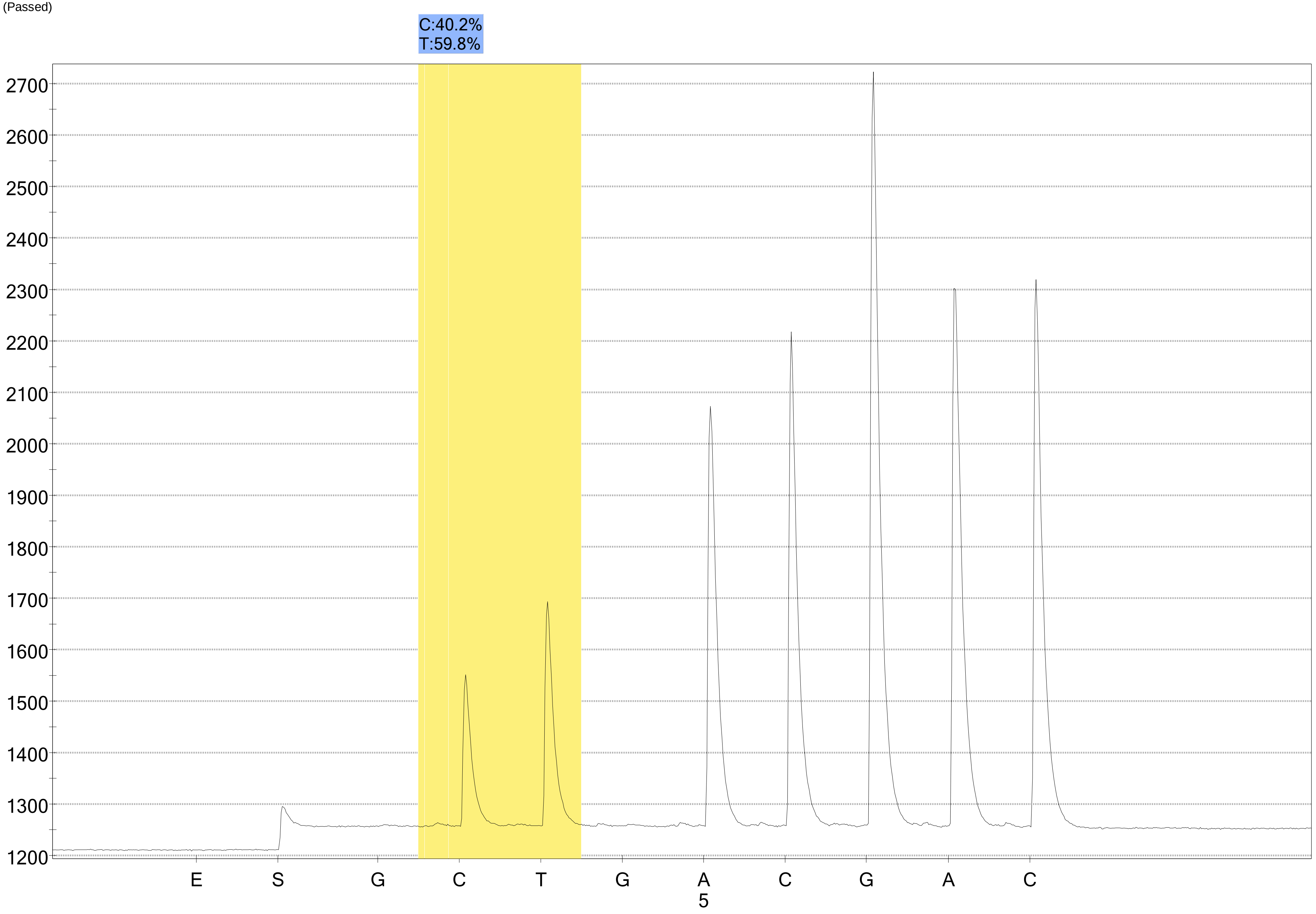

dna - Well E11
Entry: Pitpnm2
1: C: 41.6% / T: 58.4%
(Passed)

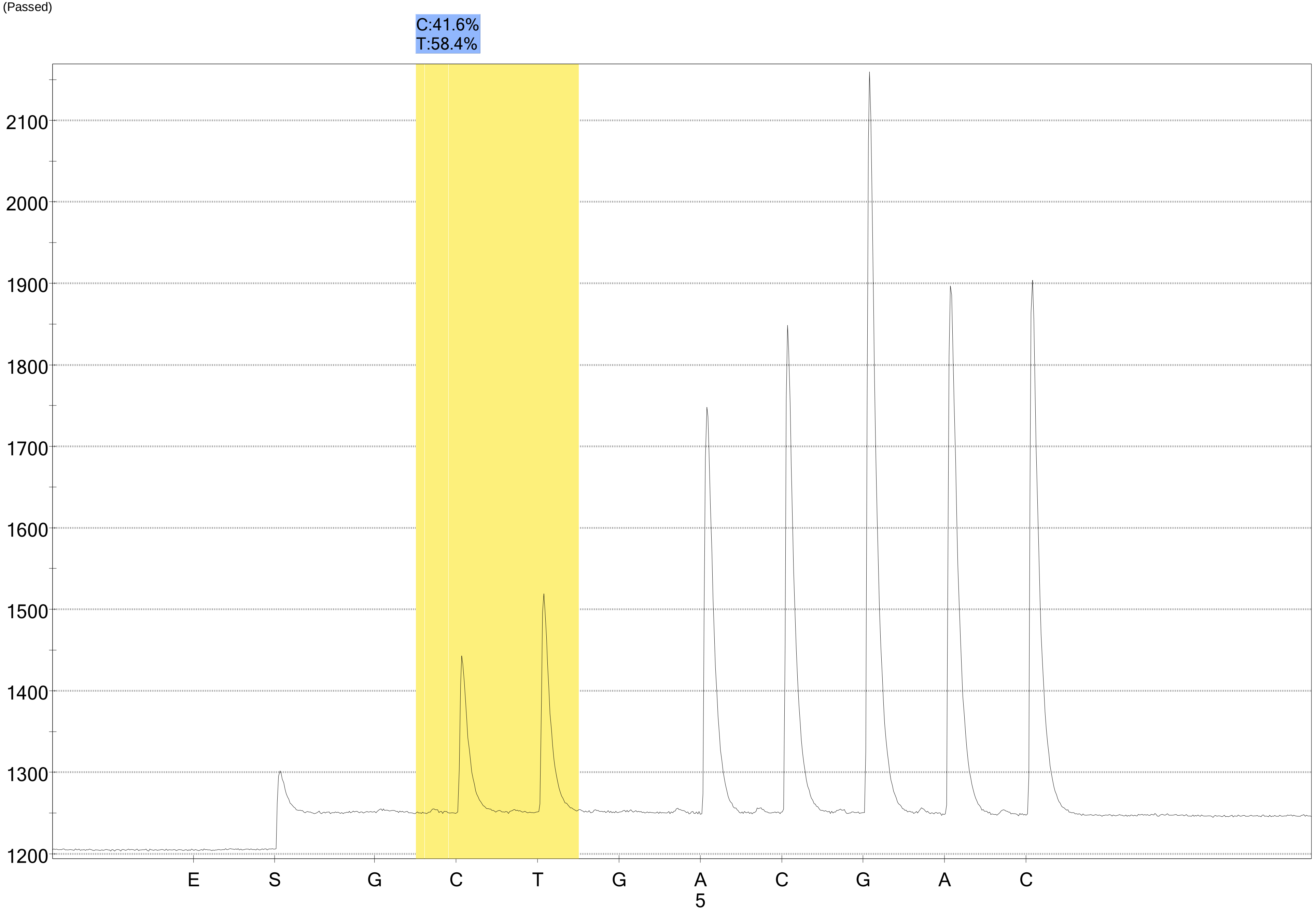

10 uL universal (141+157) - Well D1
Entry: AK011885
1: C: 50.2% / G: 49.8%
(Passed)

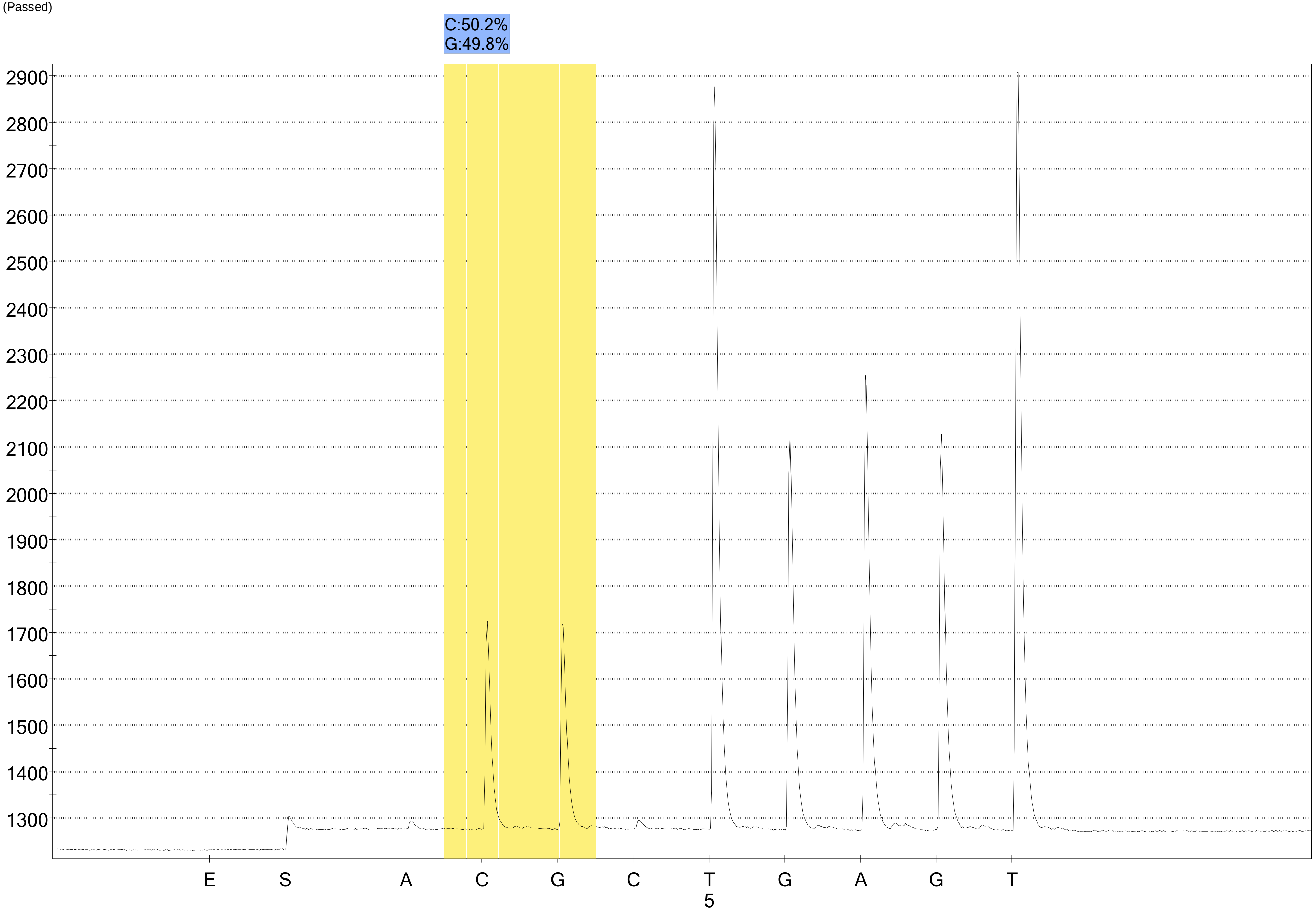

10 uL universal (141+157) - Well D7
Entry: AK011885
1: C: 50.5% / G: 49.5%
(Passed)

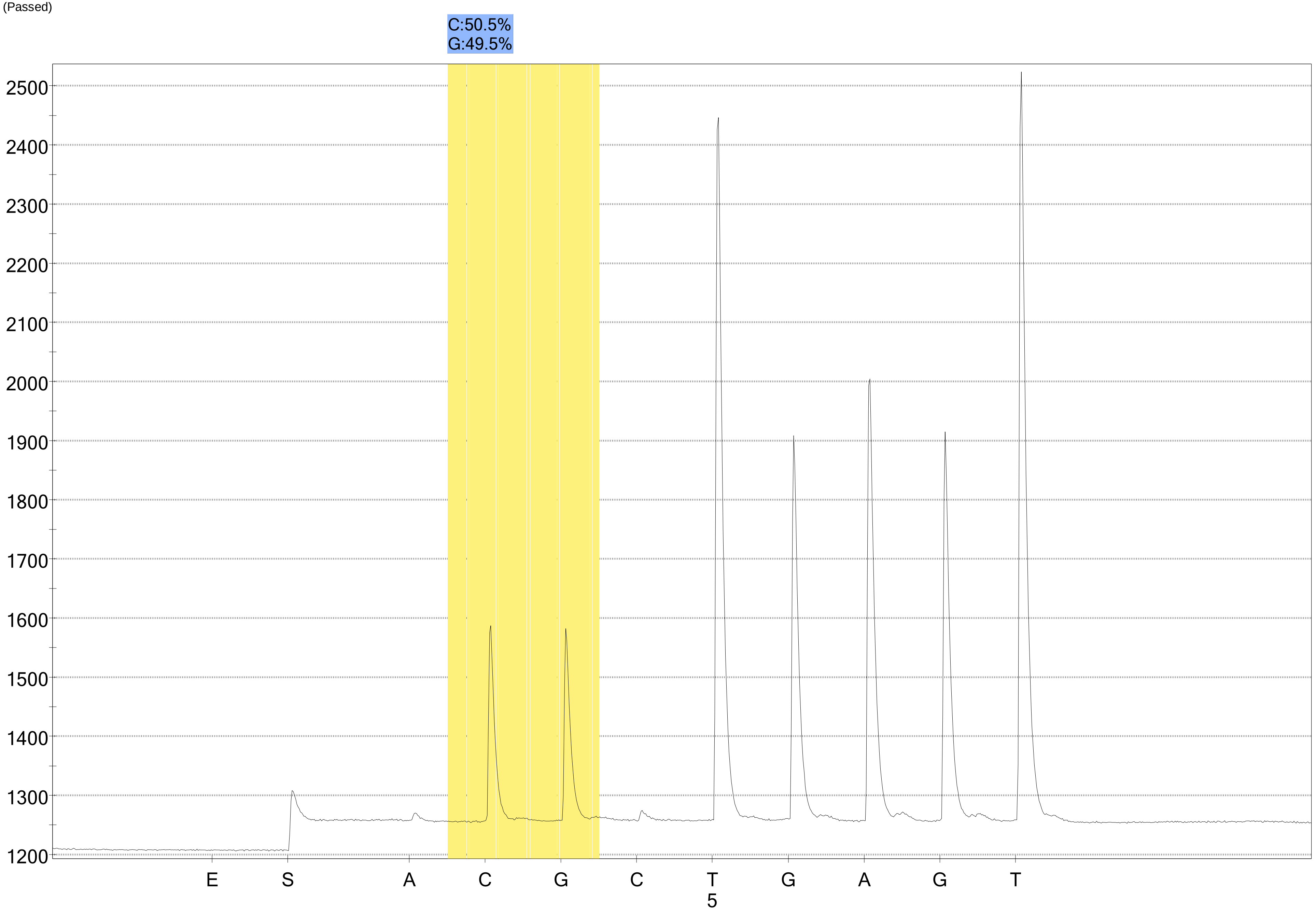

145 - Well D1
Entry: AK011885
1: C: 51.4% / G: 48.6%
(Passed)

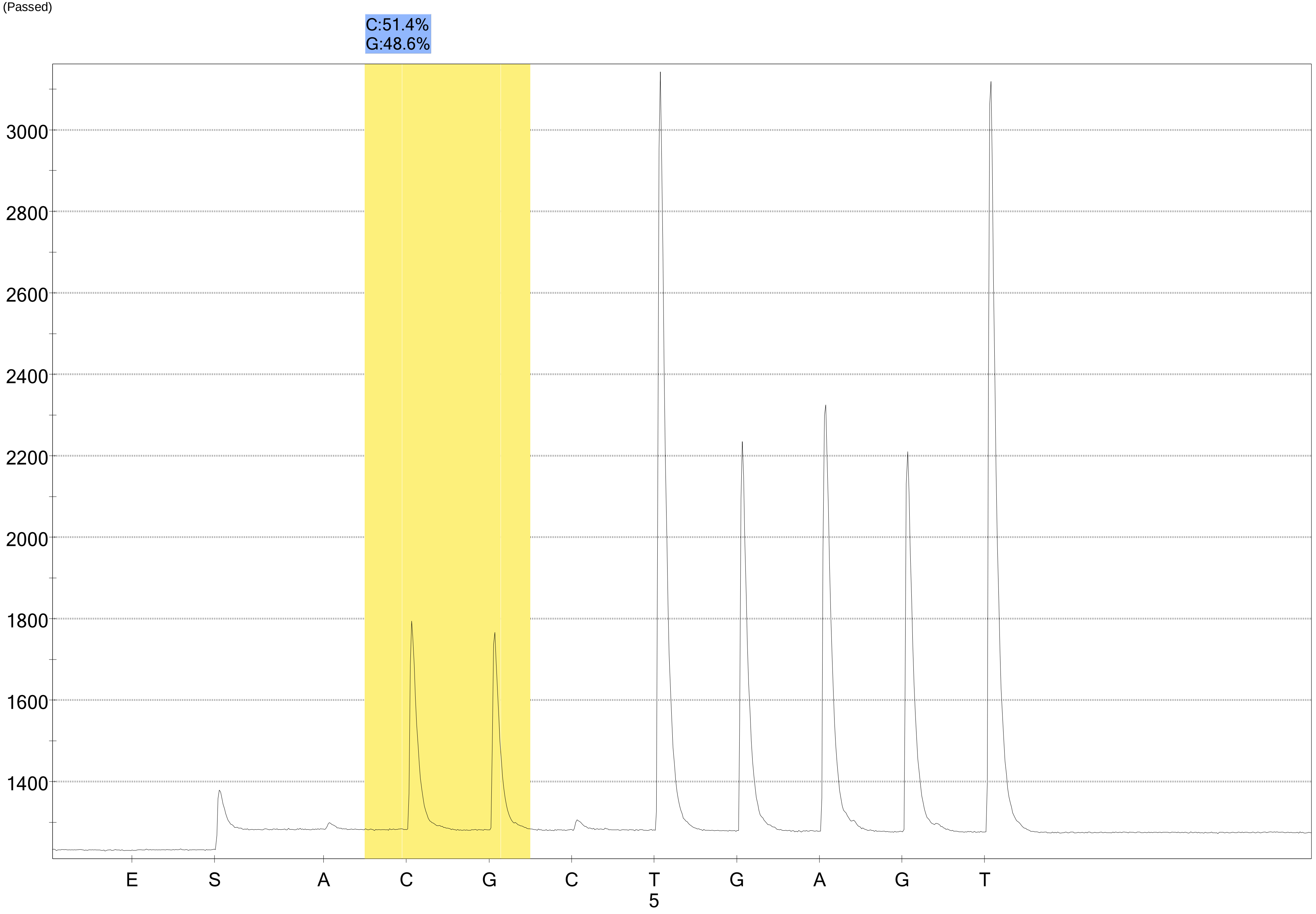

dna - Well D1
Entry: AK011885
1: C: 51.3% / G: 48.7%
(Passed)

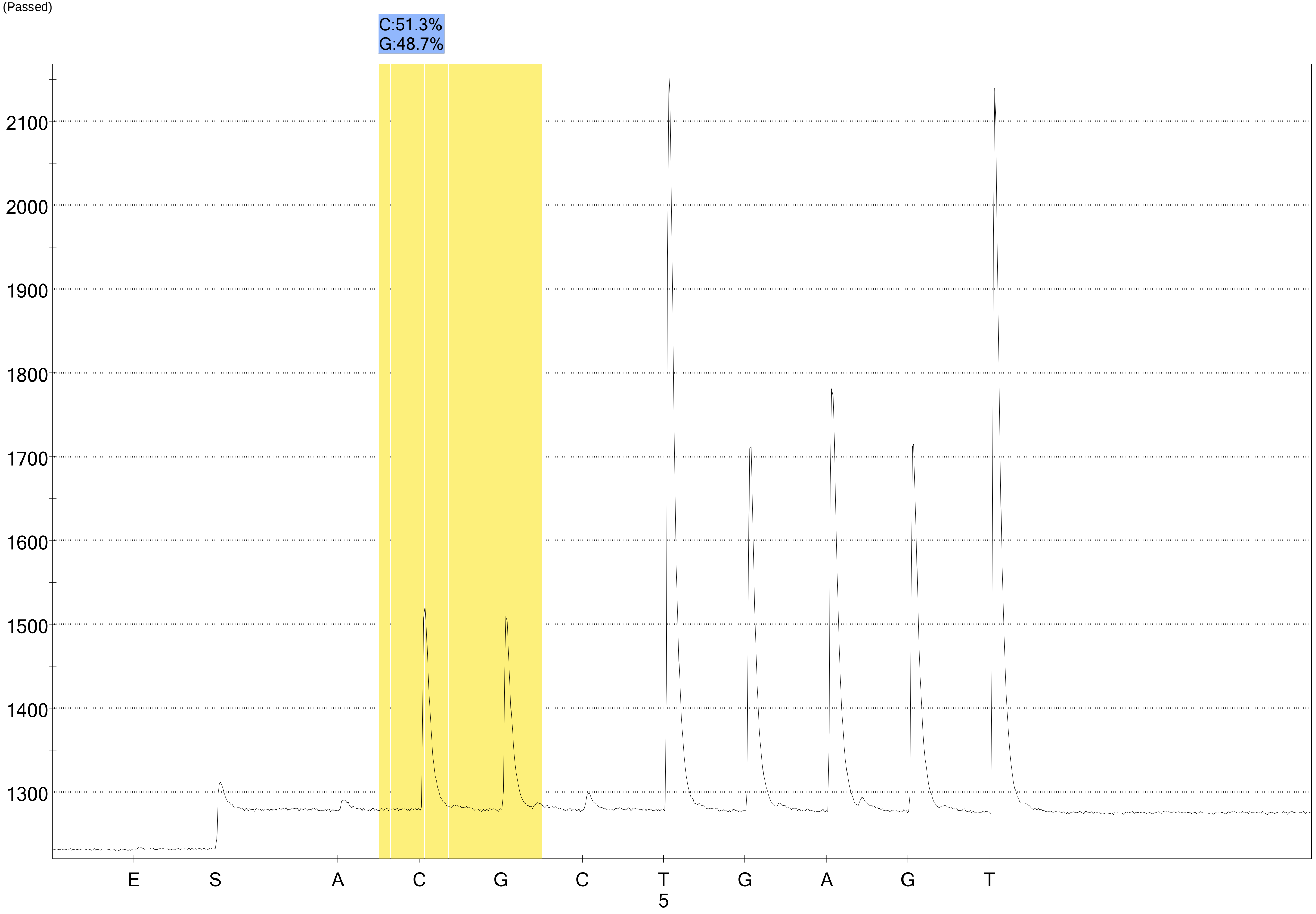

dna - Well D7
Entry: AK011885
1: C: 50.7% / G: 49.3%
(Passed)

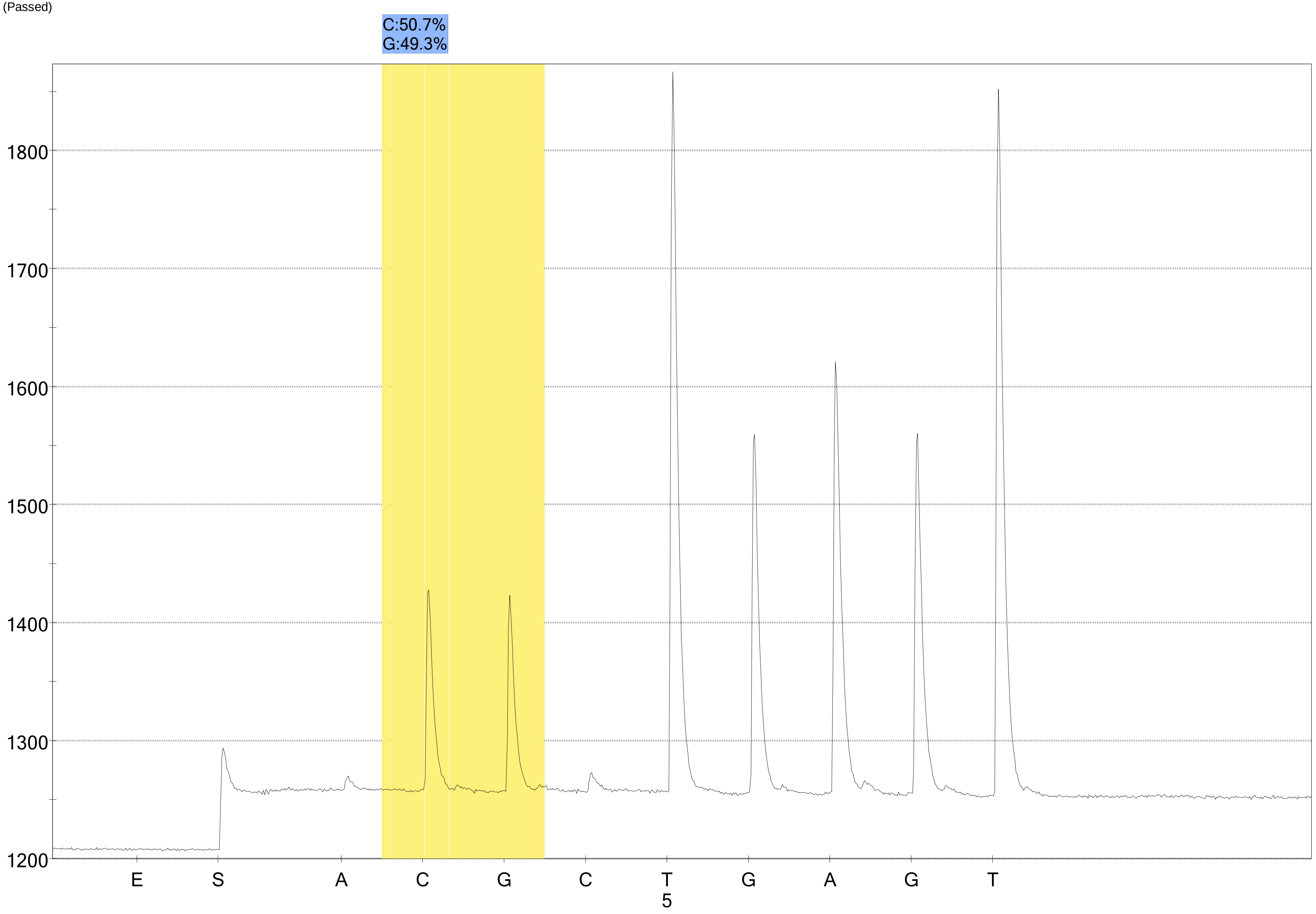

10 uL universal (141+157) - Well H1
Entry: Asb6
1: C: 55.6% / T: 44.4%
(Passed)

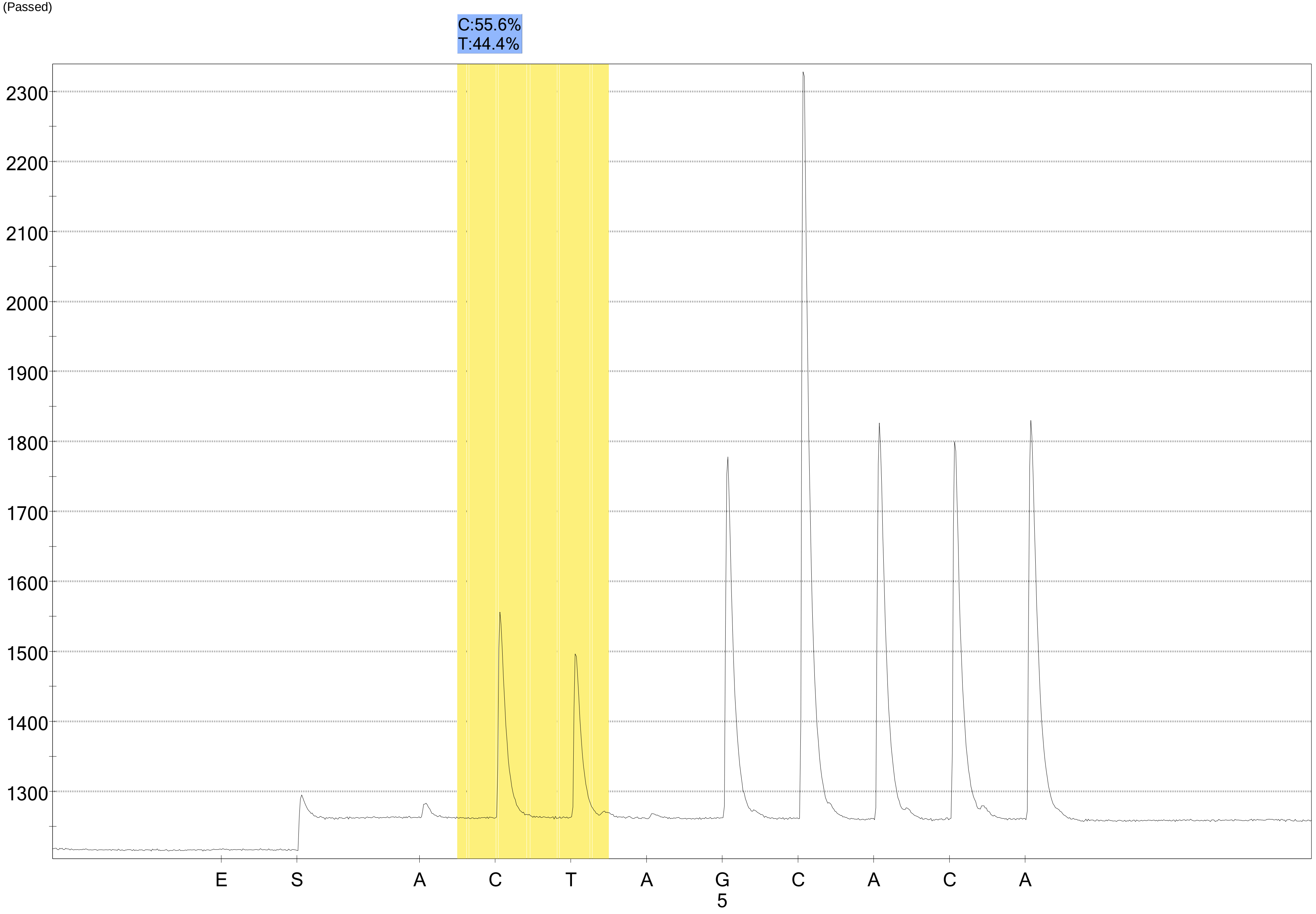

10 uL universal (141+157) - Well H7
Entry: Asb6
1: C: 55.8% / T: 44.2%
(Passed)

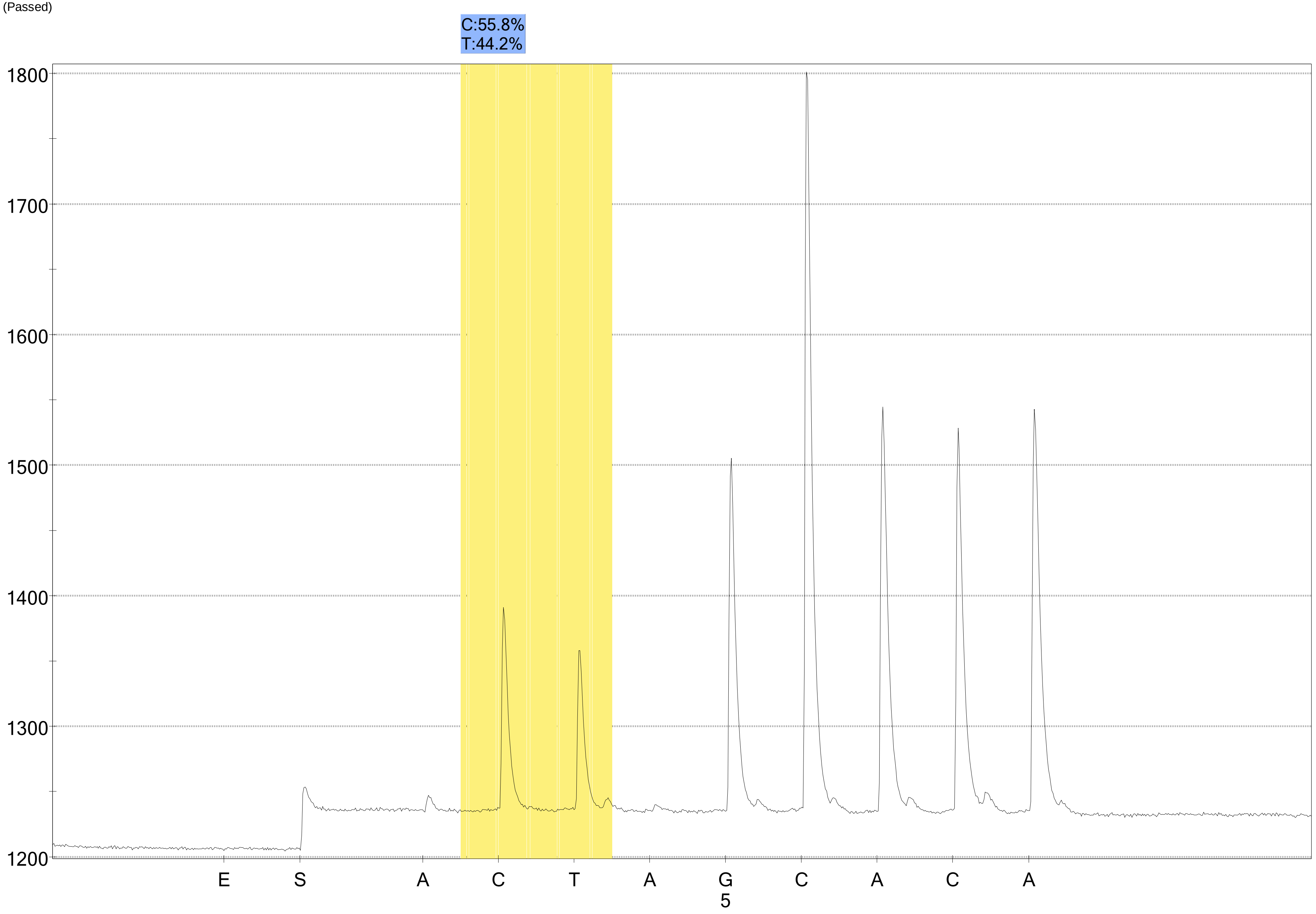

145 - Well H1
Entry: Asb6
1: C: 53.9% / T: 46.1%
(Passed)

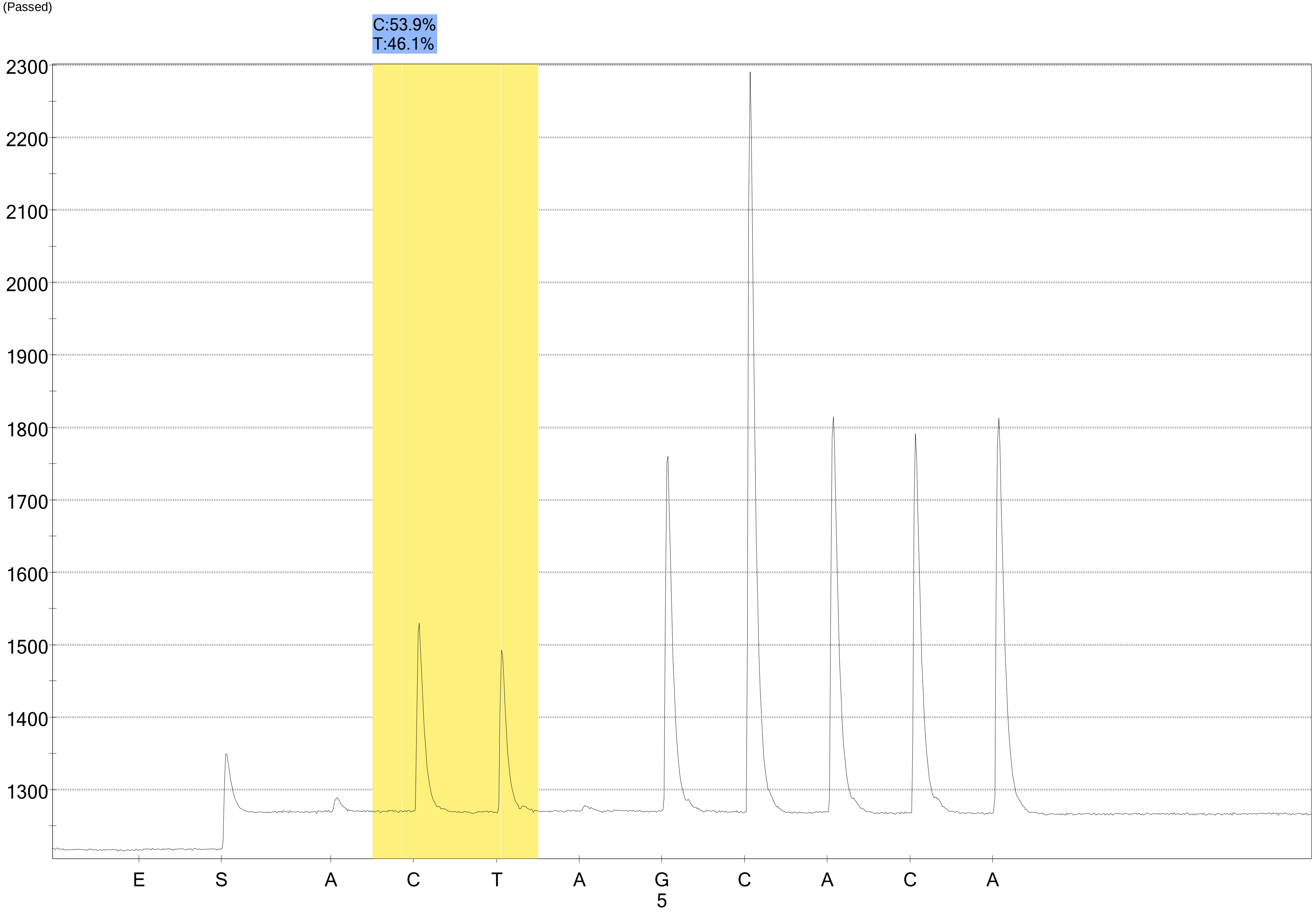

dna - Well H1
Entry: Asb6
1: C: 51.6% / T: 48.4%
(Passed)

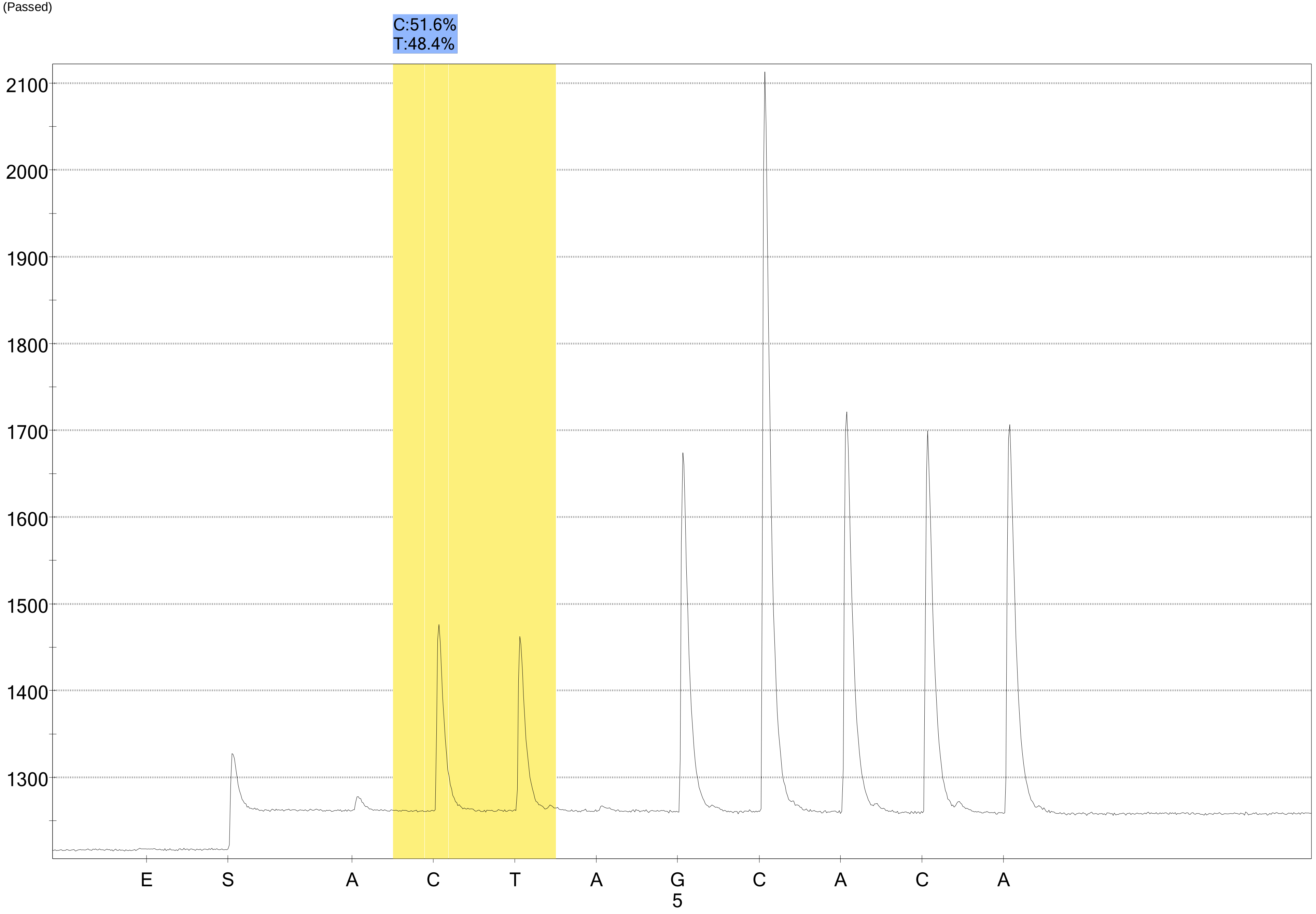

dna - Well H7
Entry: Asb6
1: C: 51.4% / T: 48.6%
(Passed)

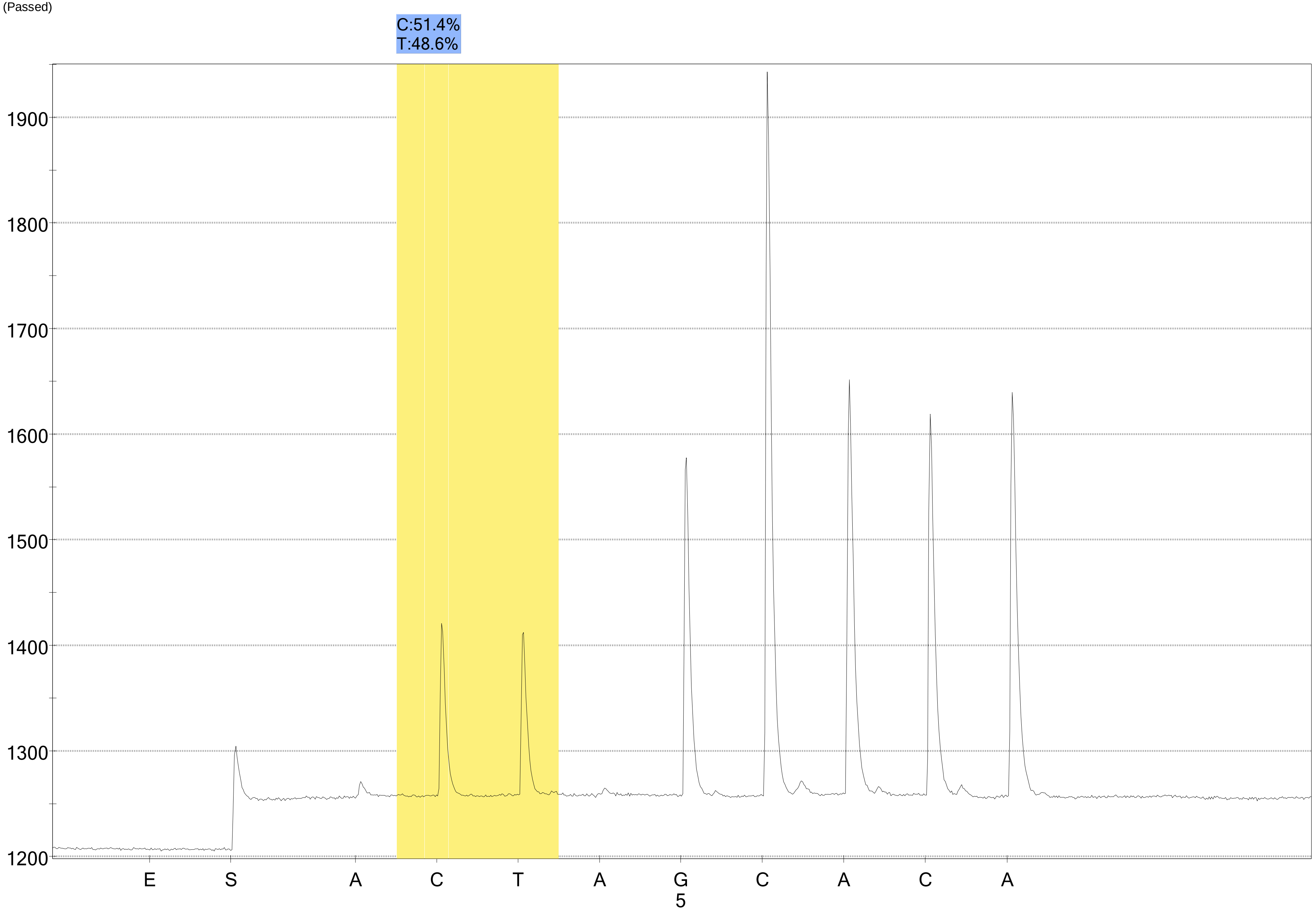

10 uL universal (141+157) - Well H2
Entry: Chmp1a
1: G: 12.2% / T: 87.8%
(Passed)

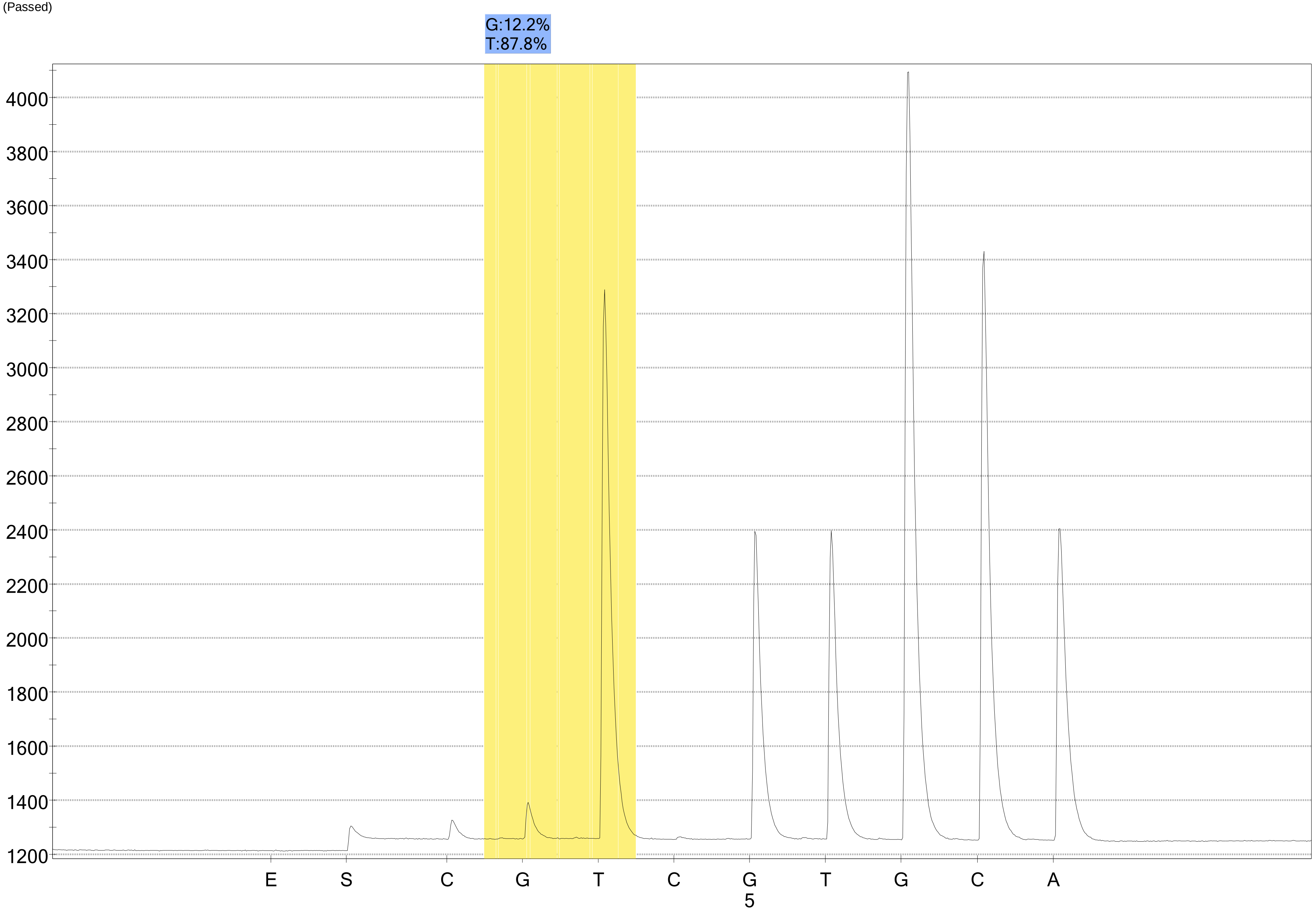

10 uL universal (141+157) - Well H8
Entry: Chmp1a
1: G: 17.9% / T: 82.1%
(Passed)

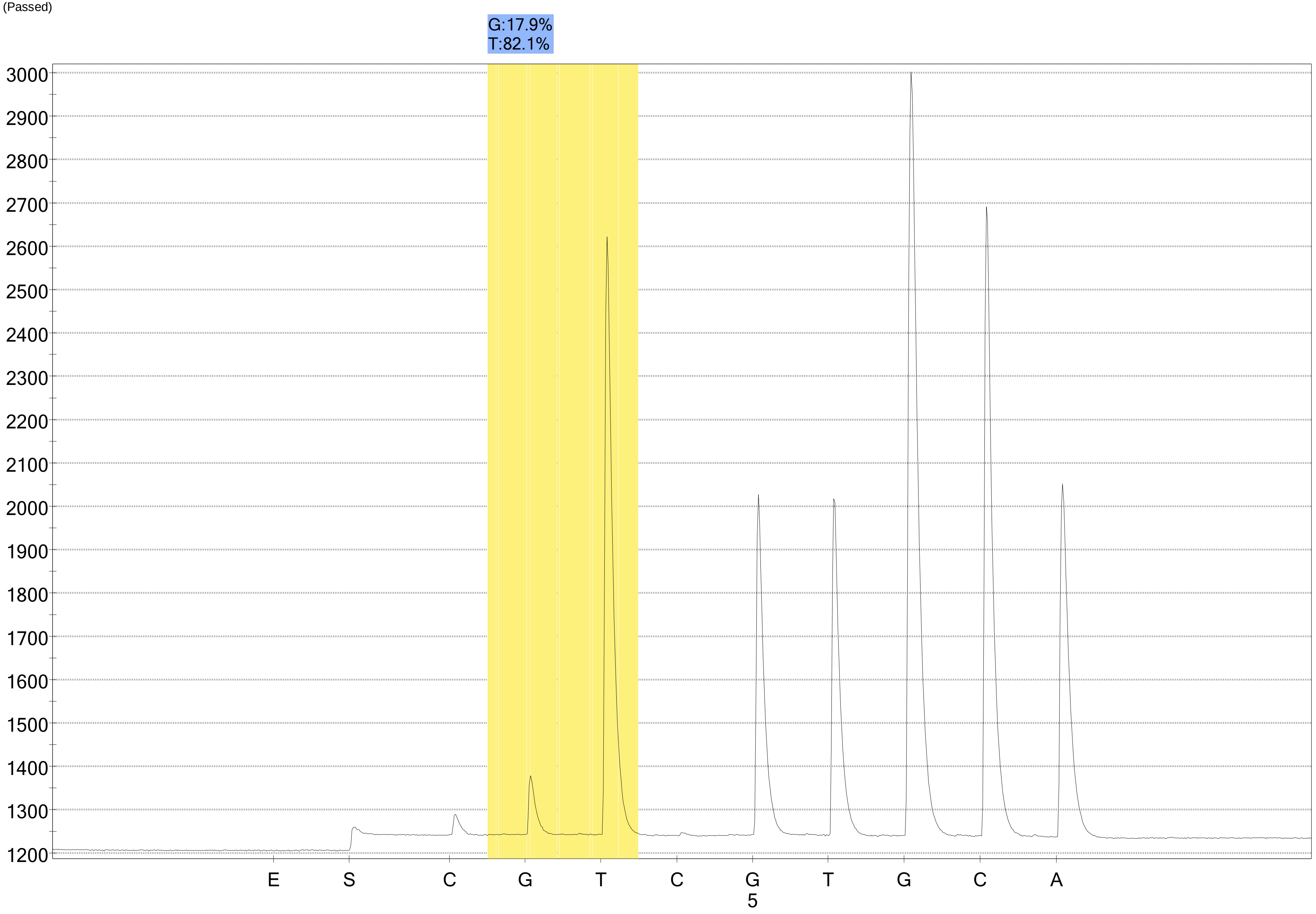

145 - Well H2
Entry: Chmp1a
1: G: 15.4% / T: 84.6%
(Passed)

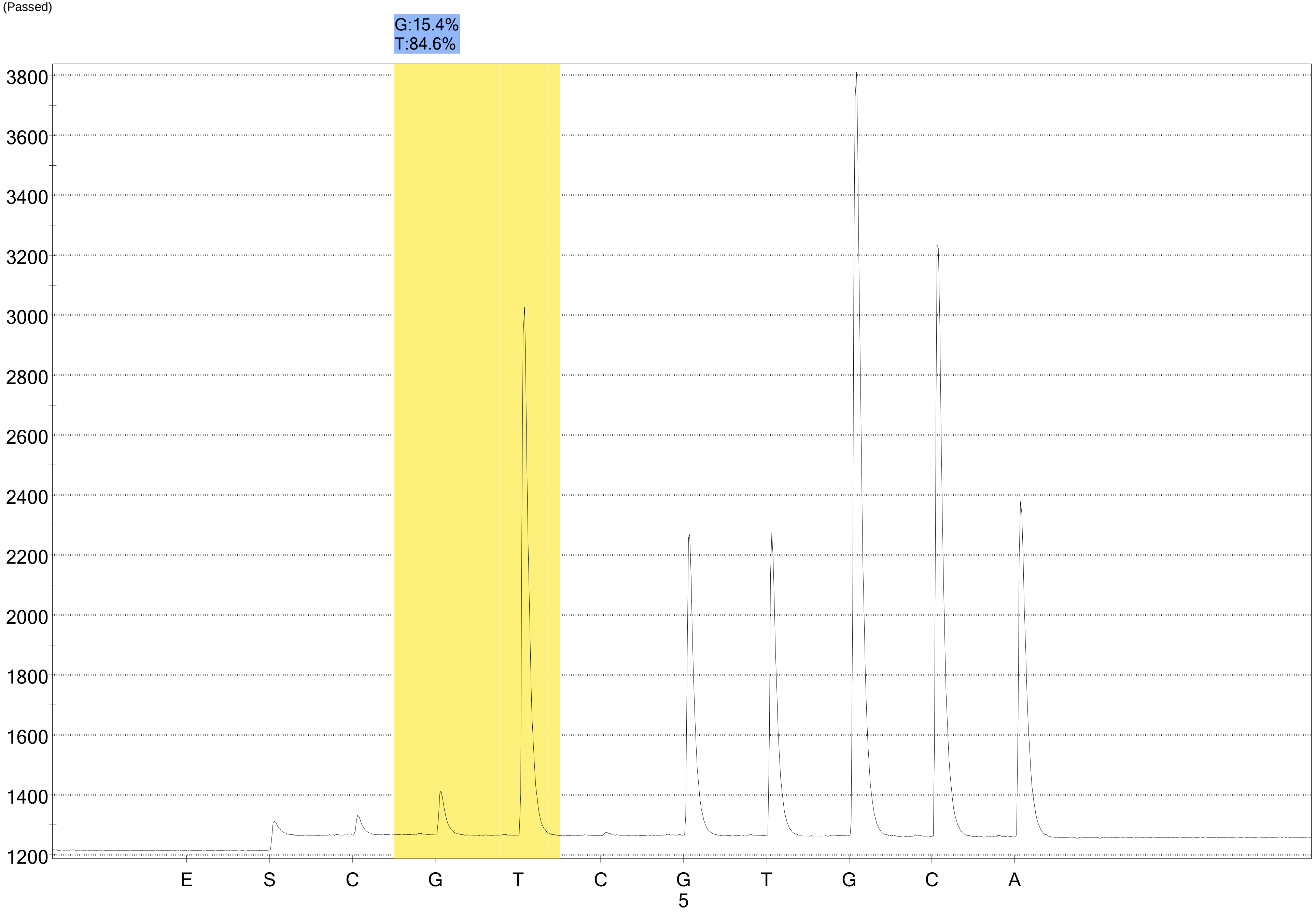

dna - Well H2
Entry: Chmp1a
1: G: 62.9% / T: 37.1%
(Passed)

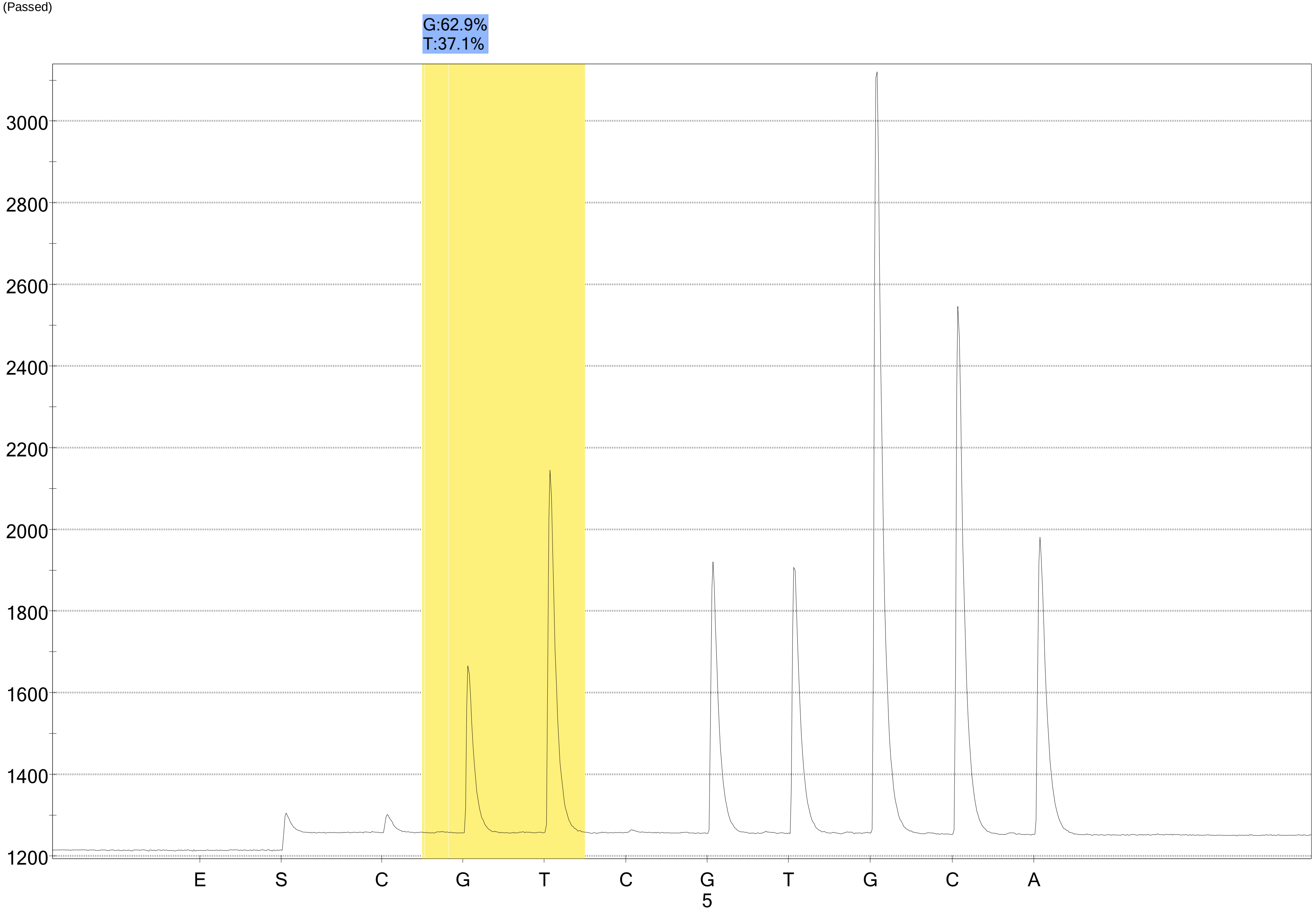

dna - Well H8
Entry: Chmp1a
1: G: 70.5% / T: 29.5%
(Passed)

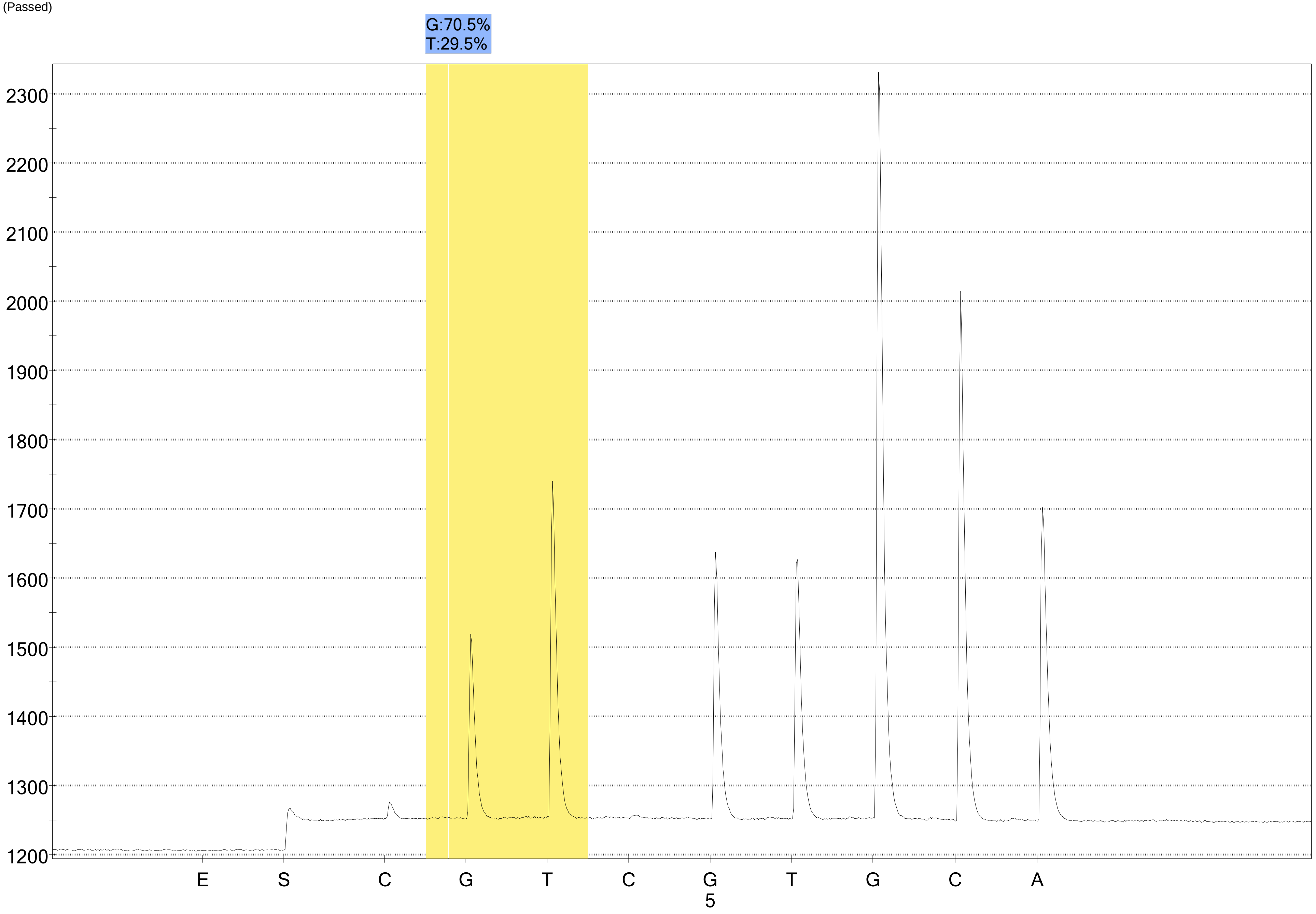

10 uL universal (141+157) - Well E3
Entry: Enpp5
4: G: 28.8% / T: 71.2%
(Passed)

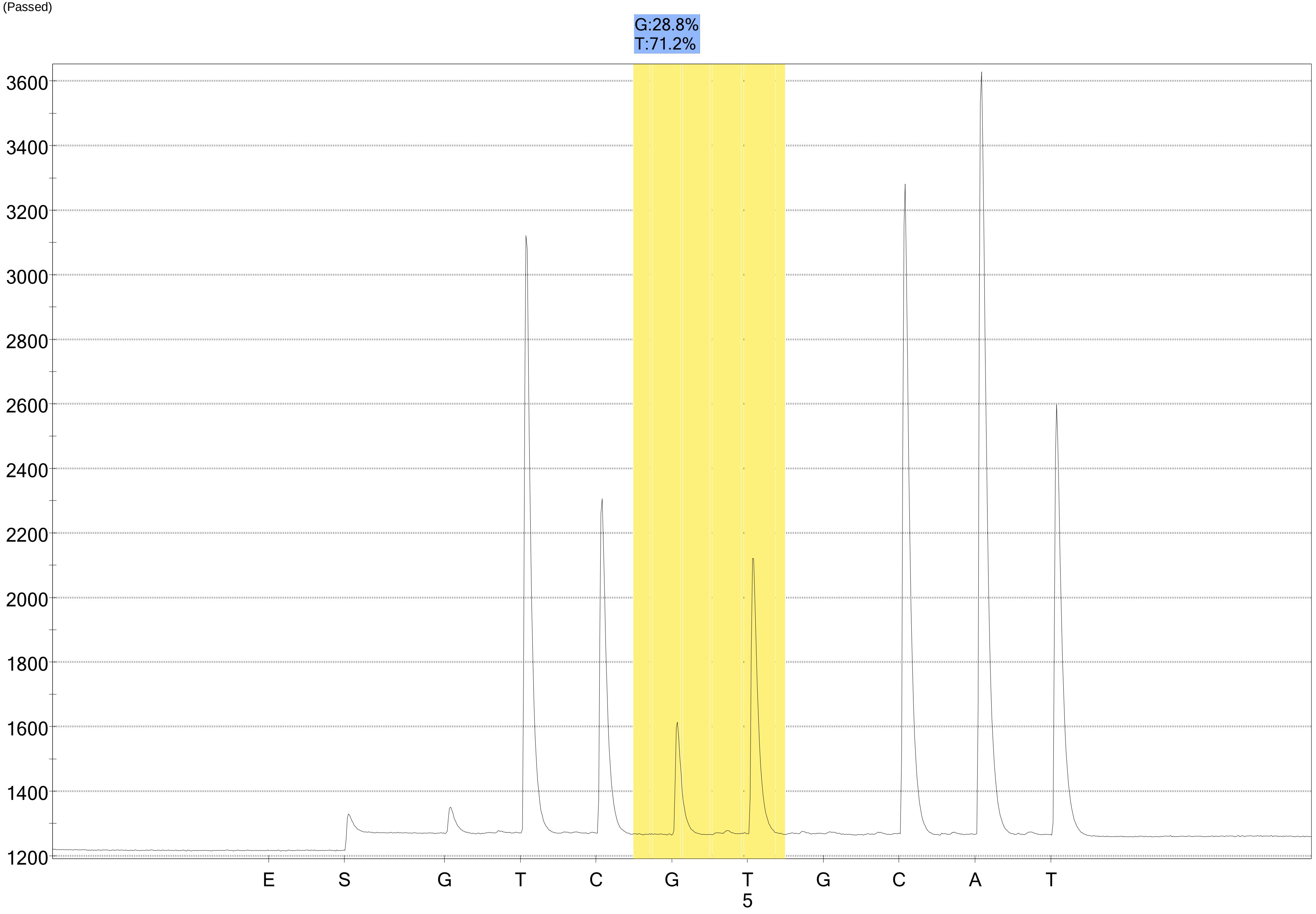

10 uL universal (141+157) - Well E9
Entry: Enpp5
4: G: 39.2% / T: 60.8%
(Passed)

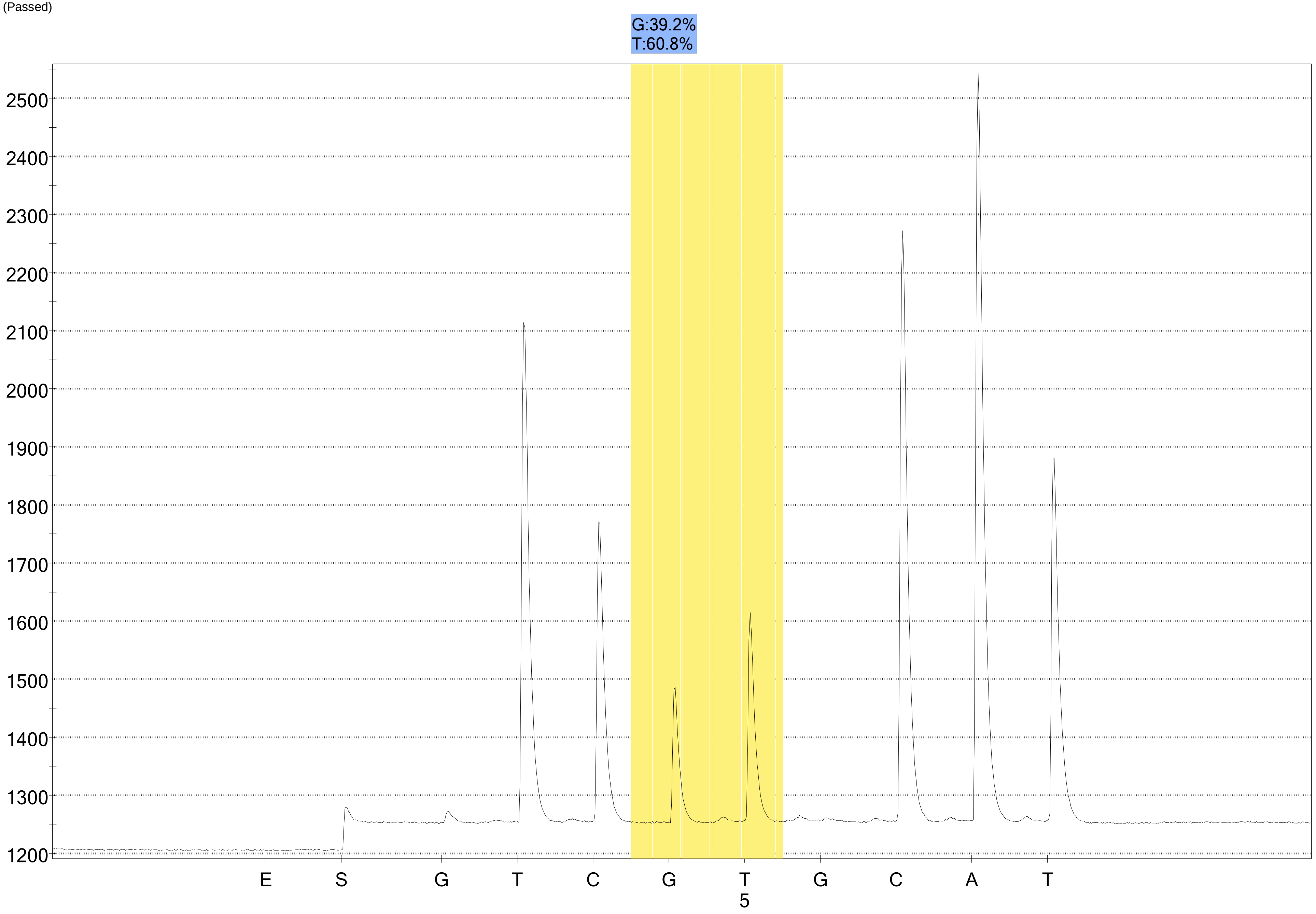

145 - Well E3
Entry: Enpp5
4: G: 28.3% / T: 71.7%
(Passed)

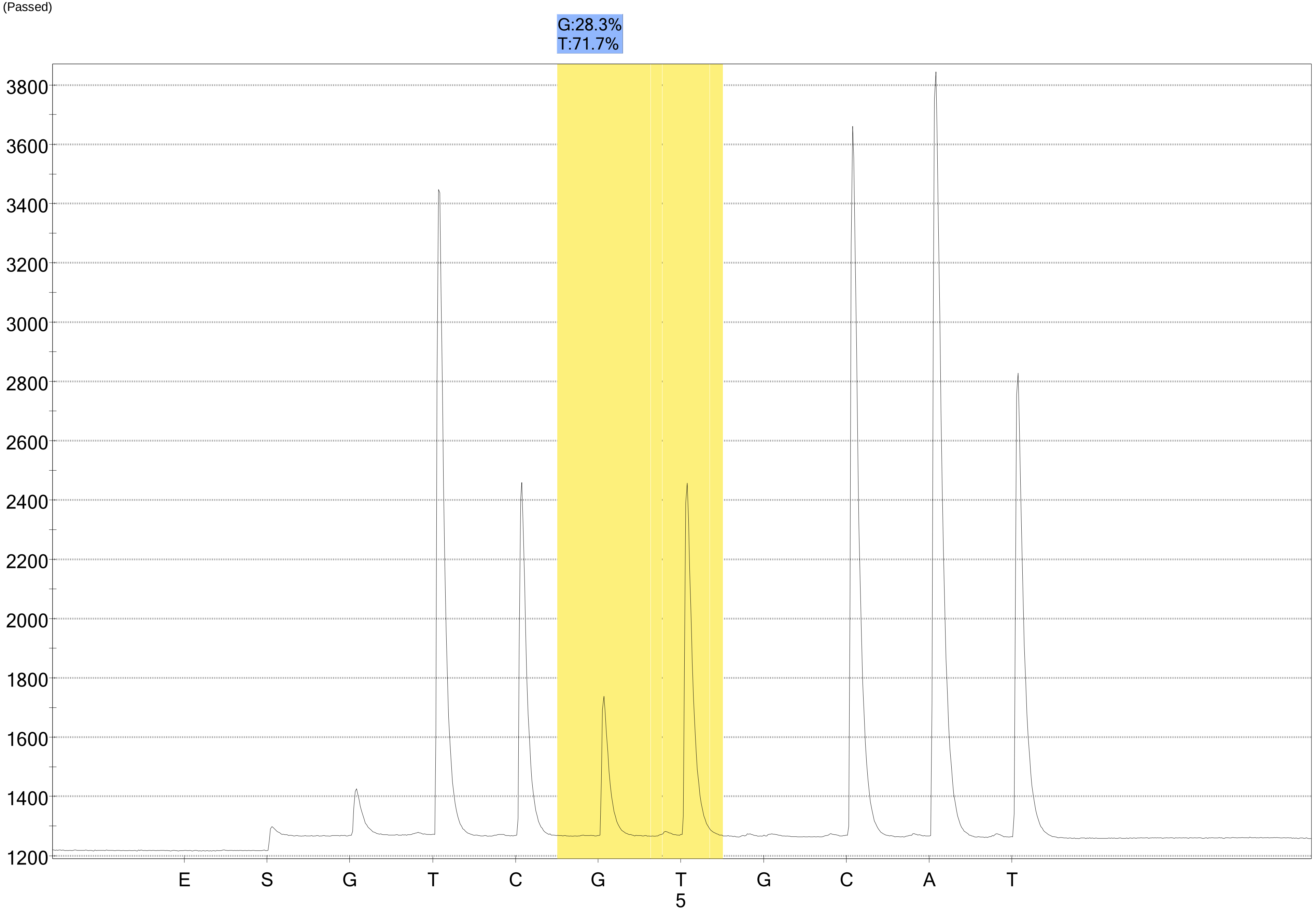

dna - Well E3
Entry: Enpp5
4: G: 26.5% / T: 73.5%
(Passed)

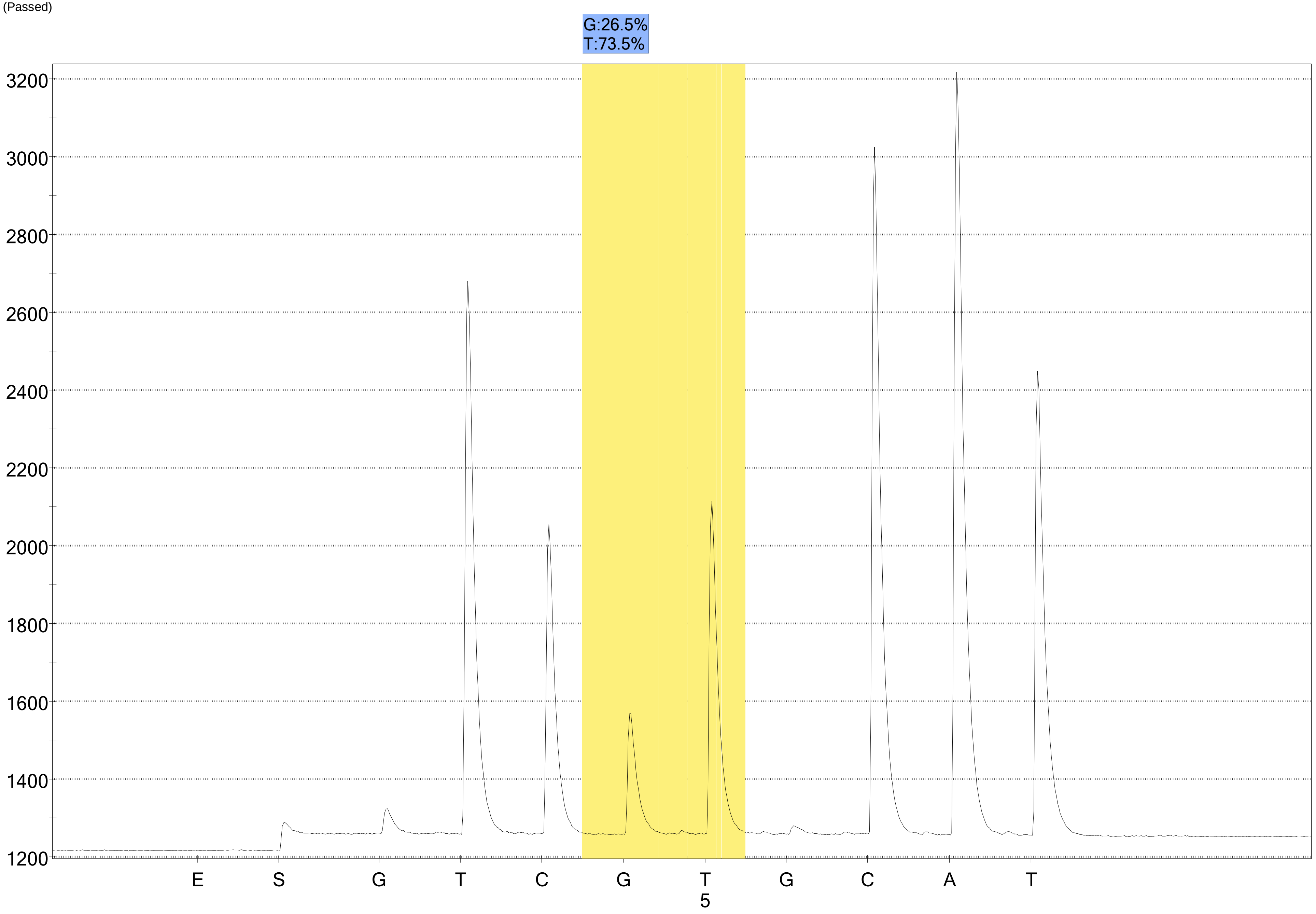

dna - Well E9
Entry: Enpp5
4: G: 22.7% / T: 77.3%
(Passed)

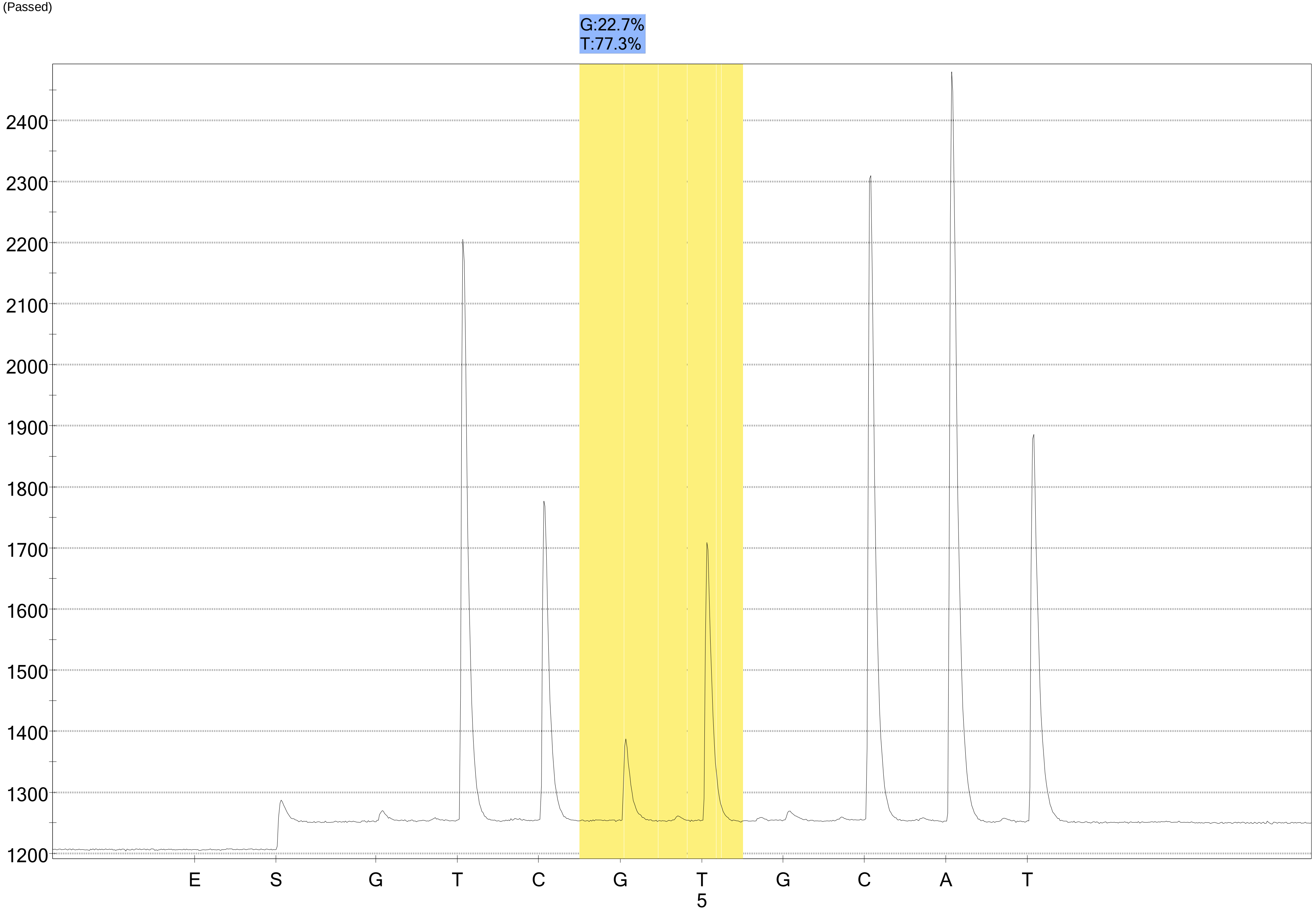

Supplement: Dataset S1 — Pyrosequencing traces analyzed to validate putative novel imprinted genes. Table of contents details page numbers for each assay. In brief, 5 traces were run for each assay that are grouped together. These are in the same order for each: BxC RNA biological replicate 1, CxB RNA biological replicate 1, BxC RNA biological replicate 2, BxC DNA biological replicate 1 and BxC DNA biological replicate 2. (PDF) [file pgen.1002600.s001.pdf]
